# The tree of life of polyamine oxidases

**DOI:** 10.1038/s41598-020-74708-3

**Published:** 2020-10-20

**Authors:** Daniele Salvi, Paraskevi Tavladoraki

**Affiliations:** 1 Department of Health, Life and Environmental Sciences, University of L’Aquila, 67100 L’Aquila, Italy; 2 Department of Science, University ‘Roma Tre’, 00146 Rome, Italy

**Keywords:** Biochemistry, Cell biology, Evolution

## Abstract

Polyamine oxidases (PAOs) are characterized by a broad variability in catalytic properties and subcellular localization, and impact key cellular processes in diverse organisms. In the present study, a comprehensive phylogenetic analysis was performed to understand the evolution of PAOs across the three domains of life and particularly within eukaryotes. Phylogenetic trees show that PAO-like sequences of bacteria, archaea, and eukaryotes form three distinct clades, with the exception of a few procaryotes that probably acquired a *PAO* gene through horizontal transfer from a eukaryotic donor. Results strongly support a common origin for archaeal PAO-like proteins and eukaryotic PAOs, as well as a shared origin between PAOs and monoamine oxidases. Within eukaryotes, four main lineages were identified that likely originated from an ancestral eukaryotic PAO before the split of the main superphyla, followed by specific gene losses in each superphylum. Plant PAOs show the highest diversity within eukaryotes and belong to three distinct clades that underwent to multiple events of gene duplication and gene loss. Peptide deletion along the evolution of plant PAOs of Clade I accounted for further diversification of function and subcellular localization. This study provides a reference for future structure–function studies and emphasizes the importance of extending comparisons among PAO subfamilies across multiple eukaryotic superphyla.

## Introduction

Polyamines are small organic polycations with primary and secondary amino groups. They are widely present in all organisms and are involved in a variety of biological processes. The relative abundance of the different polyamines depends on species and varies in a tissue-specific way. The most common polyamines are the diamine putrescine (Put), triamine spermidine (Spd), and tetraamine spermine (Spm), although a large number of algal, fungal and bacterial species do not contain Spm^1–3^. In some organisms a wider variety of polyamines has been observed, such as 1,3-diaminopropane (Dap), cadaverine, agmatine, thermospermine (T-Spm), norspermine (Nor-Spm), norspermidine, homospermidine, various long-chain and branched polyamines, as well as conjugated forms and acetylated derivatives of polyamines^4–9^.

Polyamine biosynthetic pathway is an ancient and well conserved metabolic pathway. Most eukaryotes synthesize Put from ornithine through ornithine decarboxylase (ODC), whereas in plants and bacteria there is an additional pathway to Put which involves arginine decarboxylase (ADC). It has been suggested that eukaryotic ODC was inherited from an α-proteobacterial ODC progenitor and that the ADC pathway has been acquired by plants from the cyanobacterial precursor of the chloroplast^10^. Transfer of an aminopropyl group to Put by spermidine synthase (SPDS) results to the production of Spd, while spermine synthase (SPMS) and thermospermine synthase (TSPMS) incorporate a new aminopropyl group at the *N*^*8*^-(aminobutyl)- and *N*^1^-(aminopropyl)-end of Spd to synthesize Spm and T-Spm, respectively. Additional triamines, tetramines, as well as long-chain and branched-chain polyamines may be also formed by transfer of aminopropyl or aminobutyl groups to different polyamines^8,11–15^. Phylogenetic studies have suggested that the *SPDS* genes of the various organisms derive from a common ancestor preceding the separation between prokaryotes and eukaryotes and that they have been the origin of SPMS and TSPMS activities through gene duplication and/or neofunctionalization^16^. Moreover, it has been hypothesized that plants acquired *TSPMS* early during evolution by horizontal gene transfer from archaea or bacteria^16–18^.

Polyamine oxidases (PAOs) have an important role in polyamine metabolism and contribute to several physiological processes through regulation of polyamine levels and reaction products. PAOs are characterized by a broad variability in substrate specificity, catalytic mechanism and subcellular localization. They are FAD-dependent enzymes catalyzing the oxidation of the free, and/or acetylated form, of polyamines at the secondary amino groups^19–21^. In mammals, the peroxisomal PAOs (PAOXs) preferentially oxidize *N*^*1*^-acetyl-Spm, *N*^*1*^-acetyl-Spd, and *N*^*1*^,*N*^*1*^^2^-*bis*acetyl-Spm through an *exo-*mode to produce Spd, Put, and *N*^*1*^-acetyl-Spd, respectively, in addition to 3-acetamidopropanal and H_2_O_2_^3,20,22^. Moreover, the mammalian spermine oxidases (SMOXs), which present cytosolic/nuclear localization, preferentially oxidize Spm to produce Spd, 3-aminopropanal and H_2_O_2_^19,23,24^. Unlike PAOXs and SMOXs, *Saccharomyces cerevisiae* PAO (FMS1) catalyzes the oxidation of both acetylated and non-acetylated polyamines^25,26^.

In plants, the intracellular PAOs (e.g., the *Arabidopsis thaliana* AtPAO1 with a putative cytosolic localization and the three peroxisomal AtPAO2, AtPAO3, AtPAO4) preferentially oxidize the free form of Spd, Spm, T-Spm or Nor-Spm to produce 3-aminopropanal, H_2_O_2_ and Put or Spd^27–32^. The cytosolic AtPAO5 (which has a higher activity as dehydrogenase than as oxidase) and its rice orthologue oxidize also *N*^*1*^-acetyl-Spm^9,32–34^. In contrast to the intracellular animal and plant PAOs, the extracellular *Zea mays* PAO (ZmPAO1, previously ZmPAO^35,36^) and its orthologues in *Oryza sativa*, *Avena sativa* and *Hordeum vulgare* oxidize the carbon at the *endo*-side of the *N*^4^-nitrogen of the free forms of Spd and Spm with the production of Dap, H_2_O_2_ and an aminoaldehyde^32,37^.

In some bacterial species, such as *Pseudomonas aeruginosa*, *Citrobacter freundii* and *Serratia marcesens*, Spd is oxidized by spermidine dehydrogenases with FAD and/or heme as prosthetic groups^38–40^. Reaction products of these enzymes with Spd are Dap and 4-aminobutanal, indicating cleavage at the *endo*-side of the *N*^4^-nitrogen. The *P. aerugonosa* enzyme (SpdH) oxidizes also Spm through an *exo-*mode producing Spd and 3-aminopropanal^38–41^. In several bacterial species, an FAD-dependent amine oxidase classified as putrescine oxidase (PuO) is also present. PuO additionally catalyses Spd oxidation, though less efficiently than Put oxidation^42–45^. However, being active at the primary amino group, this enzyme cannot be considered as a PAO. Unlike the well characterized polyamine catabolic pathways in eukaryotes and bacteria, nothing is known about polyamine catabolism in archaea which possess distinct polyamine biosynthetic pathways and produce long-chain and branched polyamines^15,46,47^.

While several studies have been performed on the evolutionary pathways of genes involved in polyamine biosynthesis^10,11,16–18,48^, comparatively less is known about the evolutionary history of genes involved in polyamine catabolism. Most studies have focused on the genomic identification and biochemical characterization of PAO isoforms from single species and therefore they used phylogenetic methods mainly for delimiting clusters and subfamilies to which to assign PAO isoforms^49–51^. Only a few studies considered a wide taxonomic representation of PAO sequences in large groups of organisms and enabled the elucidation of evolutionary relationships among PAO subfamilies and the processes underlying their functional and structural diversity. For example, the phylogenetic analyses of animal PAOs clarified that SMOX and PAOX subfamilies originate from a duplication event preceding the diversification of vertebrates and that subsequently SMOX and PAOX enzymes acquired differences in substrate specificity through divergent evolution and functional specialization^52,53^. Likewise, a recent phylogenetic study on plant PAOs identified four main subfamilies and two main duplication events preceding angiosperm diversification^54^. However, it is still unclear whether all plant *PAO* gene subfamilies originate from a common ancestral gene along the Viridiplantae lineage. The same applies for metazoan *PAO* gene subfamilies, whereas phylogenetic studies on fungal PAO subfamilies have not been performed at all. At a more general level, the phylogenetic origin of the extensive diversity of eukaryotic PAOs and their evolutionary relationships with the few putative PAOs recently identified in bacteria^55^ are still unknown. In this study, a phylogenetic framework was developed to explore the relationships between eukaryotic PAOs and related proteins from bacteria and archaea, and to better understand the evolutionary root of eukaryotic PAO subfamilies, with a special focus on plants that show the highest PAO diversity. Analysis of gene structure and amino acid residues of the putative catalytic sites was also performed to better understand the evolutionary processes that led to functional diversification of *PAO* genes.

## Results and discussion

### Early origin of PAO-like proteins within the three domains of life

To investigate the evolution of PAOs, we assembled, through extensive iterative sequence similarities searches, a set of 428 sequences from bacteria, archaea, and different groups of eukaryotes including alveolates, amoebozoans, cryptista, excavates, fungi, green algae, haptista, land plants, metazoans, red algae, rhizarians, and stramenopiles (Supplementary Table S1). Searches targeting genomic data of Asgard archaea and eukaryote centrohelids, glaucophytes, and metamonads returned no hits (Supplementary Table S2).

**Figure 1:**
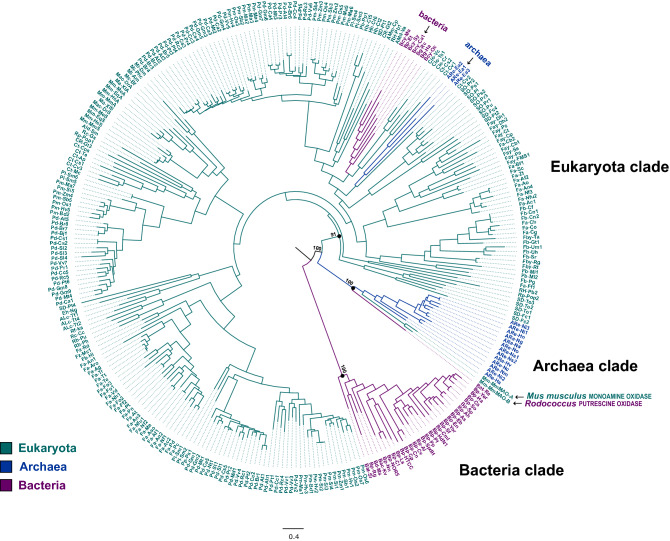
Maximum likelihood tree depicting the phylogenetic relationships between eukaryote PAOs and related PAO-like enzymes in bacteria and archaea; the tree is drawn to scale, with branch lengths measured in number of substitutions per site. Bootstrap support values (ultrafast bootstrap approximation) over 1000 replicates are reported in correspondence of the main nodes. The analysis involved 300 amino acidic sequences (see Supplementary Table S1). Sequences are coloured according to the three main domains of life: eukaryote = green, archaea = blue, and bacteria = red. The three main clades are named as follow: Eukaryota clade, Archaea clade, Bacteria clade; prokaryotic sequences clustering within the Eukaryota clade and non-archaean sequences clustering within the Archaea clade are indicated by black arrows.

Phylogenetic analyses including 300 PAO and PAO-like sequences of bacteria, archaea, and eukaryotes showed three main clades (Fig. 1): the ‘Bacteria clade’ including proteins from bacteria (Bootstrap support, BS = 100), the ‘Archaea clade’ (BS = 100) including 13 archaeal PAO-like proteins, one bacterial putrescine oxidase (*Rhodococcus erythropolis* PuO, coded as Bat-Re) and two eukaryotic monoamine oxidases (*Mus musculus* MAO-A and MAO-B, coded as Mm-MmMAO-A and Mm-MmMAO-B), and the ‘Eukaryota clade’ (BS = 91) including all eukaryotic PAOs plus two small clades of bacterial (N = 7) and archaeal (N = 3) proteins. Therefore, besides a few exceptions, each domain of life has specific PAO-like proteins that evolved from distinct ancestral proteins (Fig. 1).

Proteins of the Archaea clade are recovered as sister to the Eukaryota clade (BS = 100; Fig. 1) using either the midpoint or the MAD rooting methods, thus suggesting a shared evolutionary history between archaeal PAO-like proteins and eukaryotic PAOs. Moreover, Archaeal PAO-like proteins show a close phylogenetic relationship with the mammalian Mm-MmMAO-A and Mm-MmMAO-B^56^ and the bacterial PuO^45^, thus suggesting a common origin between PAOs and MAOs, likely from an oxidase protein carried by the common ancestor of archaea and eukaryotes^57^. A common origin between PAOs and MAOs is further supported by their significant structural similarity^35,58,59^. While biochemical information for archaeal PAO-like sequences is not available, their close evolutionary relationship with eukaryotic MAOs and bacterial PuO raises the question of whether the archaeal PAO-like proteins have catalytic properties more similar to MAOs and PuO than to PAOs.

**Figure 2:**
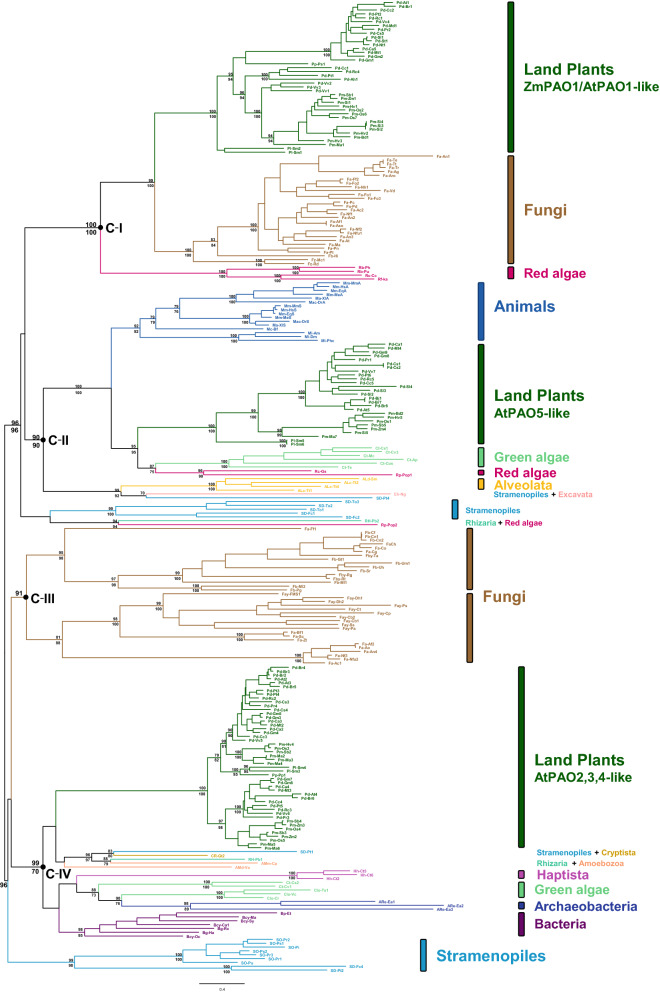
Phylogeny of eukaryote PAOs based on the 253 amino acidic sequences of the Eukaryota clade of Fig. 1. The tree shown was estimated with the Maximum likelihood method under the WAG + I + G model of amino acid replacement. Nodal support (> 70) is reported along the main branches: above, ultrafast bootstrap approximation (1000 replicates); below, SH-like approximate likelihood ratio test (1000 replicates). Main clades and eukaryote groups are indicated.

Most of the bacterial PAOs are included in the well-supported Bacteria clade. This clade includes PAOs from beta- and gamma-proteobacterial species, as well as PAOs from some alpha- and epsilon-proteobacteria, acidobacteria, actinobacteria and Deinococcus-Thermus. These PAOs have a sequence identity ranging between 30 to 75% with *Pseudomonas aeruginosa* spermidine dehydrogenase (SpdH) which oxidizes both Spd and Spm^40^. On the other hand, a few PAOs of cyanobacteria (Bcy-Oc, Bcy-Ma, Bcy-Sy, and Bcy-Ca1), chlorobacteria (Bg-Rc and Bg-Ha), and of the proteobacterium *Edwardsiella tarda* (Bp-Et) form a monophyletic clade nested within the Eukaryota clade (Fig. 1), and in particular within the PAO Clade IV (Fig. 2)*.* This latter clade includes various eukaryotic lineages (amoebozoans, criptista, green algae, haptista, land plants, rhizaria, and stramenopiles), as well as a small clade of three archaeal proteins. This phylogenetic pattern suggests that the PAOs of these prokaryotes have been probably acquired through horizontal transfer from an eukaryotic lineage^60^. However, further data on bacterial and archaeal PAO diversity, including detailed biochemical information, are required to corroborate this preliminary hypothesis.

### Four main clades of eukaryotic PAOs

PAO sequences of eukaryotes exhibit significant diversity. Phylogenetic trees show that all eukaryotic PAOs evolved from a common ancestor (Fig. 1) and represent four main evolutionary lineages referred to as Clade I-IV (Fig. 2). The four main clades of eukaryotic PAOs and their sublineages are strongly supported by bootstrap approximation and/or SH-like approximate likelihood ratio test values ≥ 90 (Fig. 2). The midpoint rooting method recovered a close relationship between Clades I and II (plus a few sequences from stramenopiles, rhizarians and red algae), as well as between Clades III and IV (plus a small clade of stramenopiles); however, the placement of the root is not consistent between the midpoint and the MAD methods (result not shown).

Clade I includes three main subclades, one represented by plant PAOs that have high similarity (33–93%) to *A. thaliana* PAO isoform 1 (AtPAO1) and *Z. mays* ZmPAO1, another one consisting of fungal PAOs (mainly from ascomycetes) and a third one, sister to the other two, consisting of red algae PAOs. Clade II includes three subclades: one composed of animal PAOs (including vertebrate SMOXs and PAOXs) that is sister to an Archaeplastida sub-clade including land plant AtPAO5-like PAOs, green algae and red algae PAOs; a third subclade includes PAOs from alveolates, stramenopiles and excavata. Clade III consists exclusively of fungal PAOs grouped into two main subclades, both including yeast forms: one subclade is composed of ascomycete PAOs including *S. cerevisiae* FMS1^25^ and the other is composed of basidiomycetes PAOs, including *Ustilago maydis* PAO (UmPAO; Fb-Um1 in Fig. 2)^2^. Clade IV includes one subclade of plant PAOs with high sequence similarity to the AtPAO2, AtPAO3 and AtPAO4 (AtPAO2,3,4-like PAOs), as well as PAOs from green algae and various protists (amoebozoans, cryptista, rhizarians and stramenopiles) and a few prokaryotic PAOs that have been discussed above. In addition to these four main clades, putative PAOs of stramenopiles form two small clades, one including diatom proteins and the other one including both diatom and oomycete proteins (Fig. 2). PAO-like sequences of these two stramenopile clades have sequence identity of 21–31% to AtPAO1-AtPAO5, MmSMOX, MmPAOX and FMS1.

The phylogenetic distribution of PAOs of each main clade in multiple eukaryotic superphyla and the lack of monophyly of PAO isoforms of plants (Clades I, II, and IV), fungi (Clades I and III), green algae (Clades II and IV), red algae (Clades I and II) and stramenopiles (various clades), suggest a birth-and-death scenario, with the origin of the main lineages arising from the ancestral eukaryotic PAO before the split of the main superphyla followed by specific gene losses in each superphylum. According to this scenario *PAO* genes of Clade I would have been lost, for example, in animals and *PAO* genes of Clade II in fungi, whereas *PAO* of Clade III would have been retained only in fungi and those of Clade IV only in green plants and some protists. On the other hand, the low number of PAO-like sequences of protists available in the databases suggests caution with the interpretation of the absence of *PAO* genes, of one or more PAO lineages, in these groups.

The four unique groups of homologous PAO sequences identified within the broad phylogenetic framework used in this study provide a crucial reference for future structure–function studies and emphasize the importance of extending the comparisons among PAO subfamilies across multiple eukaryotic superphyla. This is particularly true, for example, for the plant PAO subfamilies ZmPAO1/AtPAO1-like and AtPAO5-like that show a closer relationship to either fungal or animal PAOs rather than to the plant AtPAO2,3,4-like PAOs subfamily. This finding is in agreement with previous studies showing that *Arabidopsis* AtPAO5 is more similar to animal PAOXs/SMOXs in terms of amino acid sequence (including amino acids of the catalytic site) and substrate specificity (specificity for *N*^*1*^-acetyl-Spm), than to plant AtPAO1-4^9^. By analogy, the close phylogenetic relationships between plant ZmPAO1/AtPAO1-like proteins and ascomycete PAOs of Clade I provide directions for future comparative biochemical analyses and suggest that the available ZmPAO1 crystal structure may be a valuable resource for homology modelling and function prediction of related fungal proteins.

In the following subsections, we discuss in detail phylogenetic relationships between and within eukaryotic PAO clades in conjunction with gene structure, subcellular localization, substrate specificity and amino acid residues of the catalytic site.

### Clade I: ZmPAO1/AtPAO1-like PAOs of plants and fungi

**Figure 3:**
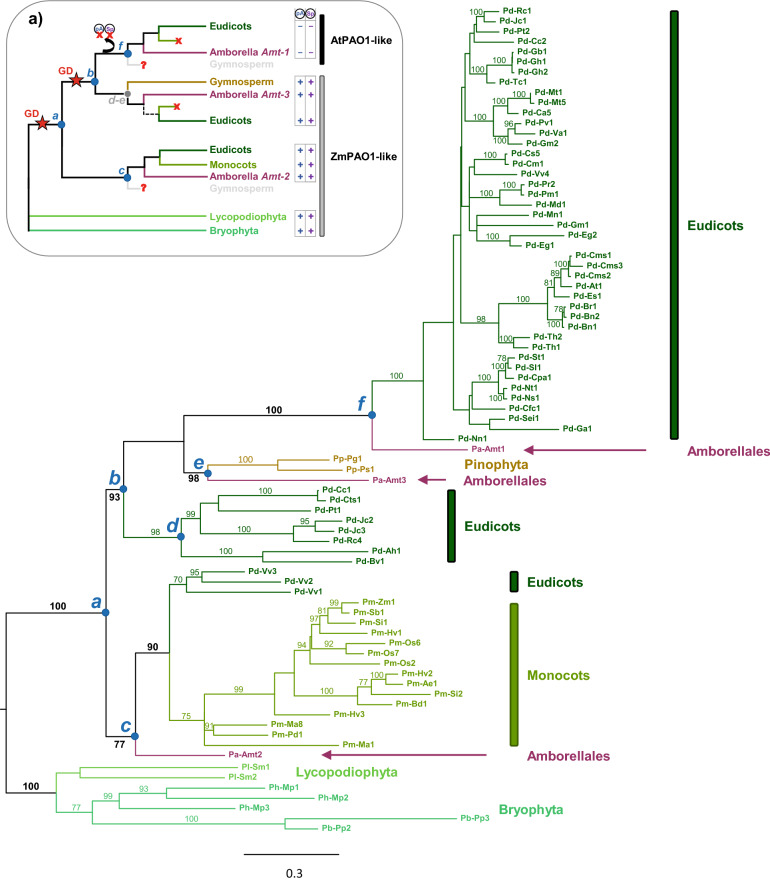
Maximum likelihood phylogeny of plant PAOs of the ZmPAO1/AtPAO1-like clade (see Fig. 2) based on 81 amino acidic sequences. Main clades are labelled from *a* to *f*; bootstrap values of support (BS) are reported along the branches (BS > 70). Box (**a**) illustrates the evolutionary model for the AtPAO1-like proteins with two gene duplications (GD) in correspondence of node *a* and* b* (giving rise to the AtPAO1 subclade)*,* and the loss of peptide A (pA) and of the signal peptide (sP) in correspondence of node *f*. Putative gene extinctions are indicated with ‘x’, whereas ‘?’ denote missing information on the Gymnosperm group.

Plant PAOs of Clade I (Fig. 2) have high amino acid sequence identity to each other (from 40 to 55%). A phylogenetic analysis based on an extended dataset, including 81 ZmPAO1/AtPAO1-like PAOs from bryophytes, lycopodiophytes, pinophytes, angiosperms (eudicots and monocots) and their sister lineage *Amborella trichopoda* (Fig. 3) shows that plant PAOs of Clade I belong to two distinct groups (Fig. 3, box a). One includes ZmPAO1-like PAOs characterized by an extracellular localization (possessing a N-terminal signal peptide for secretion to the apoplast) and an *endo*-mode of substrate oxidation; whereas the second includes AtPAO1-like PAOs characterized by putative cytosolic localization (lacking any known targeting sequence to a specific subcellular compartment) and an *exo-*mode of substrate oxidation. ZmPAO1-like PAOs are widespread across Land Plants lineages (thought in eudicot angiosperms they are only present in a few species), whereas AtPAO1-like PAOs are exclusive of eudicot angiosperms (Fig. 3).

The phylogenetic tree indicates that bryophyte and lycopodiophyte ZmPAO1-like PAOs are sister to seed plant PAOs (BS = 100, node *a*, Fig. 3). Among the latter, two large clades are present, one including ZmPAO1-like isoforms of monocots, Vitales eudicots and the *A. trichopoda* Pa-Amt2 (Fig. 3; BS = 77, node *c*) and the other one being exclusively composed of AtPAO1-like isoforms of eudicots plus the *A. trichopoda* Pa-Amt1 (BS = 100, node *f*). Two smaller clades of extracellular PAOs were also recovered that have a close relationship with the AtPAO1-like clade (BS = 100, node *b*): one is composed of ZmPAO1-like isoforms found in some eudicots (BS = 98, node *d*), and the other of a third isoform of *A. trichopoda* (Pa-Amt3) and two PAOs from gymnosperms (BS = 98, node *e*). Overall, these results are consistent with an origin of the AtPAO1-like clade from a gene duplication event occurring on a ZmPAO1-like gene ancestor before angiosperm diversification^54^. This duplication would have been followed by AtPAO1-like gene extinction in monocots and ZmPAO1-like gene extinction in several eudicots. However, the close relationship between the AtPAO1-like clade and the two newly discovered clades including ZmPAO1-like PAOs found in some eudicots, *Amborella* (Pa-Amt3) and two gymnosperms (BS = 93, node *b*) suggests an additional duplication event, followed by a gene loss in monocots, that preceded the origin of the AtPAO1-like clade (see the scheme in Fig. 3, box a). Indeed, albeit phylogenetic relationships among the AtPAO1-like clade and these two small ZmPAO1-like clades are not well resolved, the latter two have a closer affinity with the AtPAO1-like clade than with the other ZmPAO1-like clades (node *c*), strongly indicating that a single duplication event does not explain well the phylogenetic diversity of plant PAOs of Clade I. Moreover, the addition of representatives of gymnosperm PAOs compared to previous studies suggests that duplication events within this clade might have been even older than previously thought, likely before the diversification of seed plants.

Comparative analysis of the available genomic and amino acid sequences showed three main differences between ZmPAO1-like and AtPAO1-like PAOs of Clade I. All ZmPAO1-like PAOs, including PAOs of the early divergent land plants, pinophytes and two isoforms of *Amborella* (Pa-Amt2 and Pa-Amt3), share a common structure with (i) 8 introns at highly conserved positions (plus an additional intron in the two *Selaginella moellendorffii* PAOs and Pa-Amt3), (ii) a domain of 9 amino acids (peptide A, indicated as pA in Fig. 3, box a) close to Glu170 residue of ZmPAO1 catalytic site^36,58^ (aa174-aa182; numbering of mature ZmPAO1; Supplementary Figure S1), and (iii) the previously mentioned signal peptide for extracellular localization (indicated as Sp in Fig. 3, box a). In contrast, all AtPAO1-like PAOs, including *A. trichopoda* Pa-Amt1, show an additional intron at a position corresponding to the highly conserved amino acid residue Glu173 (numbering of mature ZmPAO1) (Supplementary Figure S1) and lack the peptide A and a signal peptide (given their intracellular localization). Therefore, these two peptides were lost after the gene duplication that gave rise to the AtPAO1-like clade (Fig. 3, box a).

The fungal PAO group of Clade I comprises mainly ascomycete PAOs with the single basidiomycete Fb-Hi PAO and the two zygomycete PAOs as sister to this clade (Fig. 2). Amino acid sequence identity between fungal and plant PAOs of Clade I is high (32–40%), including the amino acids of the catalytic site (see below Table 1). Most fungal PAOs (23 out of 29) have a predicted cleavable signal peptide suggesting extracellular localization; only three of them do not have typical features of the cleavable signal peptide in the N-terminal extension, whereas for the remaining three fungal PAOs only partial sequence data were available. Similarly to the extracellular ZmPAO1-like plant PAOs, the fungal PAOs possess a domain corresponding to the ZmPAO1 region aa174-aa182 (numbering of mature ZmPAO1), though with low sequence similarity. Analysis of the available genomic sequences revealed that the ascomycete PAOs share some common intron positions, which however are different from those of the AtPAO1-like and ZmPAO1-like plant PAO genes, as well from those of Fb-Hi PAO.

Within Clade I, sister to plant and fungal PAOs, there is a sub-clade of four red algae PAOs (Fig. 2), that have amino acid sequence identity of 21–30% with plant and fungal PAOs. Two of the four red algae PAOs have a putative signal peptide for extracellular localization, while for the other two only partial sequence were available thus preventing the identification of a signal peptide. Furthermore, similarly to the fungal PAOs, the four red algae PAOs possess a domain corresponding to the characteristic ZmPAO1 region aa174-aa182.

The identification of a putative signal peptide for extracellular localization in both the red algae and the fungal PAOs, as well as in most groups of plant PAOs of Clade I (except in the derived clade AtPAO1-like proteins), suggests that the ancestral PAO of this clade was extracellular and that it appeared early in the evolution of the eukaryotes.

### Clade II: AtPAO5-like PAOs of plants and animal PAOs

Clade II includes the two reciprocally monophyletic sub-clades of animal PAOXs/SMOXs and of Archaeplastida AtPAO5-like PAOs (Fig. 2) with amino acid sequence identity among them in the range of 25–37%. Clade II also includes PAOs from two alveolates (*Tetrahymena thermophila* and *Symbiodinium microadriaticum*), a stramenopile (*Phaeodactylum tricornutum*) and an excavate heteroloboseans (*Naegleria gruberi*) which present amino acid sequence identity with plant and animal PAOs of Clade II in the range of 18–31%.

In agreement with the detailed study on animal PAOs by Polticelli et al.^52^, vertebrate PAOs consist of two subfamilies, SMOXs and PAOXs, with different substrate specificity (free and acetylated form of Spm, respectively) and subcellular localization (cytosolic/nuclear and peroxisomal localization, respectively), probably derived from a duplication event followed by divergent evolution and functional specialization^52^. A recent study determined that the two PAO proteins of the cephalochordate amphioxus also show the same substrate specificity as vertebrate SMOXs and PAOXs, suggesting that gene duplication and functional specialization predates the diversification of chordates^61^.

**Figure 4:**
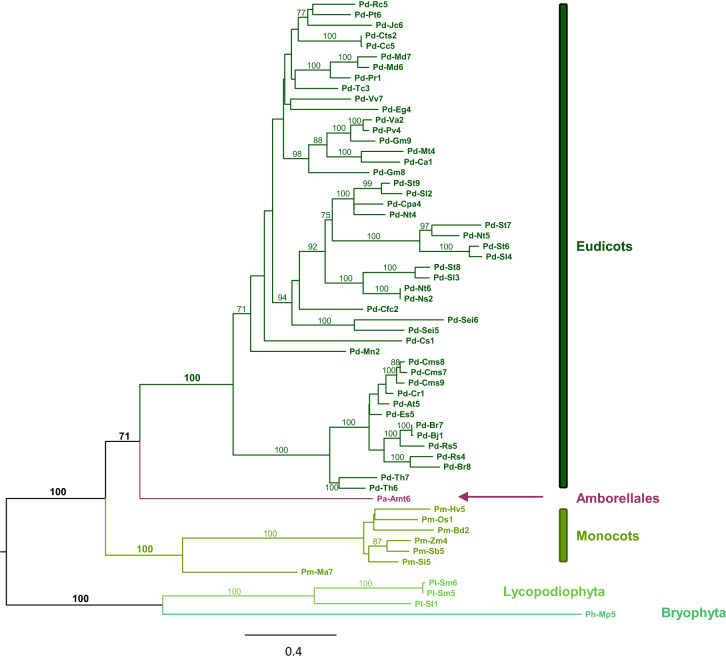
Maximum likelihood phylogeny of plant PAOs of the AtPAO5-like clade (see Fig. 2) based on 59 amino acidic sequences. Bootstrap values of support (BS) are reported along the branches (BS > 70).

As shown by a phylogenetic analysis based on an extended dataset (Fig. 4), phylogenetic relationships among plant PAO group of Clade II mirror the phylogenetic relationships among land plants and include three main sublineages, with the PAOs of *Marchantia polymorpha*, *S. moellendorffii* and *Selaginella lepidophylla* having a sister relationship to the clade formed by *Amborella* Pa-Amt6 and the groups of eudicot and monocot PAOs. Therefore, in contrasts to the vertebrate PAOs, AtPAO5-like PAOs comprise a relatively homogeneous group of proteins that have orthologous relationships, except a few plant species that have multiple copies as a result of recent gene duplications. Plant AtPAO5-like enzymes have cytosolic localization (all lacking a targeting sequence to a specific subcellular compartment) and broad substrate specificity. Indeed, AtPAO5 and the AtPAO5-like enzymes of *O. sativa* and *S. lepidophylla* are able to oxidize the two substrates of the animal SMOXs/PAOXs (Spm and acetylated Spm) in addition to T-Spm and Nor-Spm^9,34,62,63^. Furthermore, plant AtPAO5-like *PAOs* share a very simple gene structure with no intron^9^, with the exceptions of *PAO* genes in *Malus domestica* and *Tarenaya hassleriana* (Pd-Md6 and Pd-Th7, respectively) that have a single intron and in *S. moellendorffii* (Sl-Sm5 and Sl-Sm6) that have two introns. In contrast, animal *PAO* genes consist of 4 to 7 exons interspaced by 3 to 6 introns. Among animals, only *Trichoplax adhaerens* and *Nematostella vectensis*, representing early divergent animal phyla of Placozoa and echinoderms, have intron-less *PAOs.* These data indicate that animal and plant PAOs of Clade II experienced a very different evolutionary history from their common ancestor.

### Clade III: a fungal-specific PAO clade

Clade III PAOs are exclusively from fungi and include one clade of ascomycete PAOs and another of PAOs found predominantly in basidiomycetes and a few ascomycetes (Fig. 2). Both clades include also yeast forms, such as FMS1 (Fay-FMS1) and UmPAO (Fb-Um1). Sequence identity among the PAOs of the ascomycete and basidiomycete PAO clades is relatively low, ranging from 19 to 28%. Moreover, available genomic sequences showed significant differences in gene structure between these two clades of fungal PAOs. In particular, ascomycete *PAO* genes possess from 2 to 4 introns at conserved positions, with the exception of yeast forms that have intron-less genes. Basidiomycete *PAO* genes have 1 to 3 introns at conserved positions, but different to the intron positions of ascomycete *PAOs*. Overall, the intron position of fungal PAOs of Clade III are different to those of PAOs of Clade I, II, and IV. Sequence analyses suggest intracellular localization of all fungal PAOs of Clade III in contrasts to the fungal PAOs of Clade I which have extracellular localization.

### Clade IV: AtPAO2,3,4-like PAOs from plants, green algae and photosynthetic bacteria

Land plant PAOs of Clade IV have high sequence identity (55–93%) to AtPAO2, AtPAO3 and AtPAO4. Furthermore, within the Clade IV (Fig. 2), PAO-like sequences from amoebozoans, cryptista, green algae, haptista, rhizarians, stramenopiles (diatoms; SD-Pt1), and prokaryotes (archaea and bacteria) have sequence identity to AtPAO2, AtPAO3 and AtPAO4 in the range of 25–38%.

**Figure 5:**
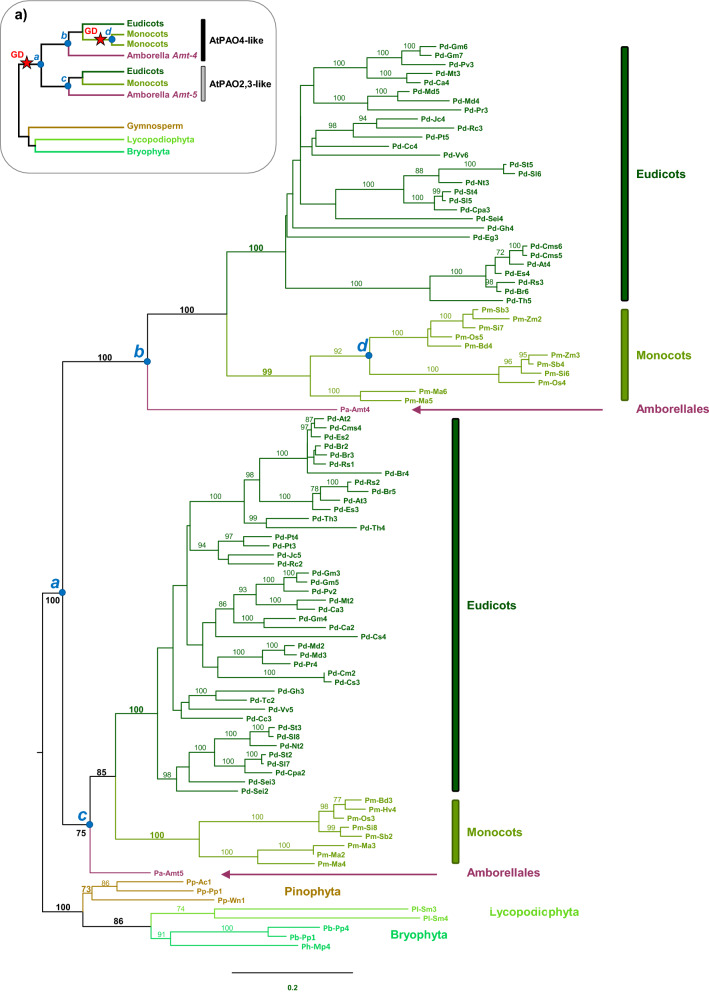
Maximum likelihood phylogeny of plant PAOs of the AtPAO2,3,4-like clade (see Fig. 2) based on 100 amino acidic sequences. Main clades are labelled from *a* to *c*; bootstrap values of support (BS) are reported along the branches (BS > 70). Box (**a**) illustrates the evolutionary model for the AtPAO2,3,4-like proteins with two gene duplications (GD) in correspondence of node *a* (giving rise to the AtPAO2,3 and AtAPO4 subclades) and *d*.

Phylogenetic analysis based on an extended dataset (Fig. 5) showed that plant AtPAO2,3,4-like PAOs are widespread across main lineages of land plants including bryophytes, lycophytes, gymnosperms and angiosperms. The phylogenetic tree further shows that angiosperm isoforms of *Amborella*, monocots and eudicots belong to two sister clades (BS = 100, node *a*; Fig. 5), one including AtPAO2,3-like PAOs (BS = 75, node *c*), and the other including AtPAO4-like PAOs (BS = 100, node *b*). Therefore, in keeping with previous studies^54^, AtPAO2,3-like and AtPAO4-like PAOs arose through a gene duplication before the origin of angiosperms (Fig. 5, box a). Furthermore, within the AtPAO4-like clade, all Poales monocots have two *PAO* copies clustered into two sister clades, suggesting that an additional duplication event took place in the AtPAO4-like *PAO* of this lineage of monocots (Fig. 5, box a).

AtPAO2,3,4-like *PAO* genes of land plants have highly conserved intron positions and upstream untranslated open reading frames^64^ (uORFs). Of note, the *AtPAO2* and *AtPAO3* uORFs are more conserved with each other than with that of *AtPAO4*^64^, which is consistent with phylogenetic results. All plant PAO proteins of Clade IV have a type I peroxisomal targeting signal (PTS1) at the carboxyl terminal and indeed peroxisomal localization has been shown for AtPAO2, AtPAO3, AtPAO4^28,29^, as well as for OsPAO3, OsPAO4, and OsPAO5^31^. Interestingly, AtPAO4-like PAOs of one of the two monocot groups bear a non-canonical, but functional, peroxisomal targeting signal at the carboxyl terminal (CRT)^31^. Despite shared subcellular localization AtPAO4-like PAOs and AtPAO2,3-like PAOs differ in catalytic properties. Indeed, while AtPAO2 is equally active with either Spm or Spd, AtPAO3 has greater activity with Spd and AtPAO4 with Spm^30^. In a similar way, while OsPAO3 of the AtPAO2,3 group is active mainly with Spd, OsPAO4 and OsPAO5 of the AtPAO4 group is mainly active with Spm and T-Spm^31^. Whether these differences reflect distinct physiological roles is still unknown.

Within Clade IV, the AtPAO2,3,4-like clade of land plant PAOs is closely related to the PAOs of green algae (chlorophytes and trebouxiophyceae)*.* The PAOs of green algae *V. carteri* and *C. reinhardtii* have a similar gene structure to each other sharing some intron positions, but different from those of the *PAO* genes of plants and of the other green algae (trebouxiophyceae) for which genomic sequences are available (e.g., Ct-Cv1 of *Chlorella variabilis*). Furthermore, *C. reinhardtii* PAO, but not the *V. carteri* PAO, has a PTS1 signal for peroxisomal localization, though peroxisomal localization has still to be demonstrated. The *PAO* gene of *C. reinhardtii* has also an uORF which however does not exhibit similarity to the uORF of the *AtPAO2,3,4* plant *PAO* genes. The close relationships of PAOs of many protists (amoebozoans, cryptista, haptista, rhizarians, and stramenopiles) with land plant PAOs of the AtPAO2,3,4-like clade allows identification of their common ancestors along the early diversification of eukaryotes, thus much earlier than during the diversification of streptophytes as suggested in previous studies^54^.

### Active site analysis of PAOs

The amino acid residues Glu62, Glu170, Tyr298, Lys300, Phe403 and Tyr439 of ZmPAO1 (Clade I) are key amino acids of the catalytic site as shown by resolution of the crystal structure^58,65^, molecular modelling studies and site-directed mutagenesis^36,66^. Glu62 and Glu170 are located close to the cofactor FAD and residue Tyr298 is found in close proximity to Lys300, the ε-amino group of which is H-bonded through a water molecule with the N5 atom of FAD, an atom which participates in the catalytic mechanism^58^. Moreover, Phe403 and Tyr439 flank the catalytic tunnel on opposite sides and form a kind of ‘aromatic sandwich’^58^. Sequence alignments of PAOs of Clades I to IV, as well as of Archaea, Diatom and Stramenopile clades showed that Lys300 residue of ZmPAO1 is strictly conserved in all PAOs (Table 1). This residue is also present in RePuO and Mm-MAOs (Table 1). Furthermore, except for some fungal PAOs, all PAOs analysed (and also RePuO, Mm-MmMAO-A and Mm-MmMAO-A) have an aromatic amino acid (either Phe or Tyr) at position Phe403 of ZmPAO1 (Table 1). Tyr439 is highly conserved in Clade I (including fungal PAOs) and is also present in the Archaea Clade, while it is substituted mostly by Thr or Ser residues in the PAOs of the other clades, with the exception of the oomycete proteins in which it is substituted by Ala. Tyr439 is also conserved in Mm-MmMAO-A and Mm MmMAO-B and substituted by His in RePuO (Table 1). These observations suggest that the ‘aromatic sandwich’ Phe403/Tyr439 is a particular characteristic of Clade I PAOs, as well as of the archaea PAO/MAO-like sequences. Glu62 is highly conserved in the ZmPAO1-like PAOs of monocots, *Amborella* (Pa-Amt2 and Pa-Amt3), and the dicot *Vitis vinifera* (Pd-Vv1, Pd-Vv2, Pd-Vv3), but highly varies (Asn, Ile; Phe, His) in the extracellular PAOs present in some other dicots (node *d*, Fig. 3). It is also present in several fungal PAOs of Clade I, while it is substituted by an Ala residue in all AtPAO1-like PAOs and by a His residue in most of the other PAOs clades (Clades II, III, and IV, and stramenopile clades). Glu170 is also well conserved in PAO-like sequences of the various clades (including the bacterial and archaeal proteins of Clade IV, and of those of the stramenopile clades), with the exception of the land plant AtPAO5-like PAOs in which it is substituted by Gln. Furthermore, in some PAO-like sequences ZmPAO1 Glu170 residue is not well-defined (Table 1, ND) due to the presence of gaps and/or regions of low sequence homology. Ser402, which in the Mm-MmMAO-A and Mm MmMAO-B is substituted by a Cys residue involved in covalent binding to the isoalloxazine ring of the FAD, is also highly conserved (Table 1). Only the AtPAO4-like PAOs of Clade IV have a Cys residues at this position which, however, is not involved in covalent binding of the FAD^30^. Unlike the other residues of the ZmPAO1 catalytic site, Tyr298 highly varies across the PAOs. In particular, while an aromatic residue is present at this position in the PAOs of Clade I, it is substituted by a Thr residue in vertebrate SMOXs, by a Val residue in the plant and algal PAOs of Clade II and some invertebrate PAOs (such as insect PAOs and the two PAOs from the amphioxus *Branchiostoma floridae*), and by a Asn residue in all vertebrate PAOXs^67^. Further studies are necessary to understand whether these variations in amino acid residues of the PAO catalytic sites correlate to variations in substrate specificity.

**Table 1:** Amino acid residues of the catalytic site of the various PAOs. Amino acid numbering refers to ZmPAO1 mature protein^35^.

	Clade	E62	E170	Y298	K300	T402	F403	Y439
Clade I	Land plant AtPAO1-like (42)	A_34_, Q_1_, V_7_	E	Y	K	S	Y	Y
	Land plant ZmPAO1-like (36)	E_25_, H_1_, I_1_, F_3_, S_1_, N_2_	E_34_, T_2_	Y_35_, F_1_	K	S_23_, T_14_, C_1_, A_1_	F_20_, Y_16_	Y
	Fungi (29)	Q_13_, E_11_, H_4_, S_1_	E_27_, G_1_, D_1_	Y	K	S	F_28_, Y_11_	F_18_, Y_11_
	Red algae (4)	Q	E	Y	K	S_3_, A_1_	Y_3_, F_1_	Y_3_, F_1_
Clade II	Land plant AtPAO5-like (35)	H	E_12_, Q_20_, H_2_, N_1_	V	K	S	Y	T
	Animal SMOX-like (38)	H	E	T	K	S	Y	T
	Animal PAOX-like (24)	H	E_23_, T_1_	N_21_, S_1_, C_2_	K	S	Y	T
	Animal PAOX/SMOX (12)	H	E_11_, A_1_	V_8_, A_1_, I_1_, T_1_, L_1_	K	S_10_, A_2_	Y	T
	Green Algae (6)	H	Q_5_, T_1_	V	K	S	Y	T_5_, C_1_
	Red Algae (2)	H	E	V	K	S	Y	T
	Alveolates, diatoms, Excavates (6)	H	ND	G_4_, V_1_, A_1_	K	S_1_, T_1_, A_1_, N_2_, G_1_	Y	T
Clade III	Fungi (36)	H	ND	L	K	S_21_, A_15_	Y_22_, T_14_	C_15_, T_6,_ S_13_, R_1_, M_1_
Clade IV	Land plant AtPAO2,3,4-like (102)	H	E	E	K	S_64_, C_37_, A_1_	Y	S_83_, T_19_
	Green algae (4)	H	E_2_, Q_2_	Y_2_, L_2_	K	S	Y	T
	Diatoms, amoebozoans cryptista, rhizarians (5)	H_4_, V_1_	E	L	K	S_4_, A_1_	Y_4_, F_1_	T_3_, S_1_, C_1_
	Haptista (3)	H	E	V	K	A	Y	T
	Bacteria (7)	H_6_, Q_1_	E	L	K	S_6_, A_1_	Y	T
	Archaea (3)	H	A_2_, S_1_	A	K_2_, V_1_	A_1_, G_1_, S_1_	Y	T_2_, A_1_
Archaea Clade	Archaea (9)	G	ND	V	K	G_7_, C_1_, A_1_	Y	F_5_, Y_3_, H_1_,
	RePuO (1)	S	ND	V	K	A	Y	H
	Mm-MmMAO (2)	G	ND	V	K	C	Y	Y
Diatom Clade*	Diatoms (5)	Y	ND	M_1_, V_1_, S_1_, F_1_, L_1_	K	A_3_, S_1_, V_1_	Y_4_, H_1_	T_4_, N_1_
Stramenopile Clade	Oomycetes (7)	H	E	Y_4_, C_3_	K	A_4_	Y_4_	A_5_
	Diatoms (2)	H	E	Y	K	S_1_, A_1_	Y	S

Numbers in parentheses indicate the number of PAOs analyzed. Subscript numbers indicate the number of PAOs sequence with each amino acid residue at the corresponding position. ND: not determined, due to the presence of gaps and regions of low sequence homology.

*SD-To1,2,3 and SD-Fc1,2.

## Conclusions

The tree of life of polyamine oxidases suggests a common origin for archaeal PAO-like proteins and eukaryotic PAOs, which probably also involved the evolution of monoamine oxidases. Within eukaryotes, four main clades of PAOs were identified, likely originated from an ancestral eukaryotic PAO before the split of the main supergroups and followed by specific gene losses in each supergroup. As a result, while some eukaryotes present a high diversity of PAO isoforms belonging to multiple clades (e.g. land plants and stramenopiles), some others have PAOs belonging to one (animals) or two clades (e.g. fungi and green algae). Within each of these clades, phylogenetic patterns revealed that PAOs have undergone several diversification events. Evolution of Clade I and Clade IV is shaped by multiple gene duplications. Conversely, only a few gene duplication events occurred within Clade II and Clade III. Clade I PAOs have additionally experienced peptide deletion leading to functional changes and diversification in subcellular localization. The latter has been a pervasive process along eukaryotic PAO evolution, most organisms having PAOs in two or three different subcellular compartments (extracellular space, cytosol and peroxisomes), which suggests different physiological roles. The large variety of PAOs analysed in the present study may facilitate structure–function studies.

## Methods

### Protein sequence homology search and retrieval

The amino acid sequence of PAOs were retrieved by sequence similarity searches using BLASTP^68^ (NCBI, Uniprot and Phytozome databases) and TBLASTN (NCBI TSA database). As query sequences the amino acid sequence of the following PAOs, for which enzymatic activity had been previously verified, were used: *Arabidopsis thaliana* AtPAO1, AtPAO2, AtPAO3, AtPAO4, AtPAO5^9,27,30^, *Zea mays* ZmPAO1^35^, *Pseudomonas aeruginosa* SpdH^40^, *Saccharomyces cerevisiae* FMS1^25^, *Ustilago maydis* UmPAO^2^, *Mus musculus* SMOX^24^ and *M. musculus* PAOX^23^. Following an initial search on the entire databases, several protist lineages were not represented in the dataset. To further assess the presence of PAO-like proteins in these lineages, we repeated the search using the same query sequences and specifically targeting, for each eukaryotic super-group, those species for which genomic resources were available (Supplementary Table S1). Among retrieved sequences, we selected those having a sequence identity with the query sequence ≥ 20%, a coverage ≥ 60% and an E-value ≤ 1e^−6^. Selected sequences were further validated based on sequence length (selecting sequences in the range of 400–650 amino acids), annotation of protein function, and the presence of particular domains. FAD-dependent PAO-like sequences with SWIRM domains, which are involved in histone oxidative demethylation^69^ rather than in polyamine metabolism, were excluded.

### Sequence Analysis

Subcellular localization was inferred based on amino acid sequences using PSORT and SignalP. Genomic exon–intron structure comparison was performed by means of alignment between genomic and cDNA sequences. Amino acid residues of catalytic site were retrieved by multiple sequence alignments performed using Clustal Omega 1.2.1^70^ and based on ZmPAO1 and Fms1 crystal structure^26,58,65,66^.

### Phylogenetic analysis

Multiple amino acid sequence alignments were performed using Clustal Omega 1.2.1. On large data sets, Clustal Omega outperforms other packages in terms of execution time and quality^70^. Multiple sequence alignments were not trimmed. Phylogenetic analyses of the amino acid sequences were performed using the Maximum Likelihood (ML) method on five distinct datasets for a total of 428 PAO-like sequences (see Supplementary Table S1). Multiple sequence alignments and phylogenetic trees are provided in Supplementary Data. The first dataset included 300 sequences of Bacteria (37), Archaea (16), and Eukaryotes (247). Subsequently, ML analyses were performed on the monophyletic group including all eukaryotic PAOs and a few prokaryotic PAOs (253 sequences) based on a new alignment. Additionally, to increase the taxonomic representation, we built, through additional similarity searches, three extended datasets of land plant PAOs for each of the AtPAO1-like (Clade I), AtPAO5-like (Clade II), and AtPAO2,3,4-like (Clade IV) clades, and we made new alignments for each of these clades. Phylogenetic trees were rooted using the midpoint method, which is a valuable method when a proper outgroup is not available or difficult to identify^71^. Additionally, we tested the root position using the Minimal Ancestor Deviation (MAD) method, that has been shown to outperform existing methods^72^. Details on numbers of taxa, sites and informative sites are reported for each alignment in Supplementary Table S3.

For each dataset the best-fit model of amino acid replacement was selected by ModelTest-NG 0.1.5^73^, using an optimize Maximum-Likelihood topology and branch lengths for each model (*-t ml*) and the Akaike Information Criterion (AIC). The WAG model^74^ with gamma distributed rates across site (+ G) and a proportion of invariant sites (+ I) was selected for the eukaryote dataset, and the JTT model^75^ with gamma distributed rates and a proportion of invariant sites (+ I) was selected for each plant PAO dataset. ML tree searches were performed with IQ-tree^76^ (for dataset larger than 200 sequences) and PhyML 3.0^77^ (for dataset smaller than 100 sequences) using the best-fit model and 100 random starting trees. Node support for the resulting phylogenetic tree was evaluated by 1000 bootstrap replicates in IQ-tree (using both the ultrafast bootstrap approximation and the SH-like approximate likelihood ratio test) and by 100 bootstrap replicates in PhyML. Phylogenetic analyses were carried out on the T-REX webserver^78^ and the CIPRES Science Gateway 3.3^79^ (at https://www.phylo.org/).

## Supplementary information

Supplementary information

Supplementary Figure S1

Supplementary Table S1

Supplementary Table S2

Supplementary Table S3

## Data Availability

GenBank accession numbers of all sequences used in this study are reported in Supplementary Table S1.
