# Supplementary material for: The tree of life of polyamine oxidases
Source: Sci Rep. 2020 Oct 20;10:17858. doi: 10.1038/s41598-020-74708-3 (PMC7576179; doi:10.1038/s41598-020-74708-3)
Supplement: Supplementary file 1 — Supplementary information [file 41598_2020_74708_MOESM1_ESM.doc]

Scientific Reports

**The Tree of Life of Polyamine Oxidases**

Daniele Salvi1 and Paraskevi Tavladoraki2,*

1 Department of Health, Life & Environmental Sciences, University of L'Aquila, 67100 L’Aquila, Italy.

2 Department of Science, University ‘Roma Tre’, 00146 Rome, Italy.

*Corresponding author: Paraskevi Tavladoraki, paraskevi.tavladoraki@uniroma3.it

**Supplementary Data**

Data included in this Supplementary file:

**Alignment files generated in this study (fasta format)**

Alignment Three-domains (Fig 1)

Alignment Eukaryota Clade (Fig 2)

Alignment Land Plants Clade I (Fig 3)

Alignment Land Plants Clade II (Fig 4)

Alignment Land Plants Clade IV (Fig 5)

**Tree files generated in this study (Newick format)**

ML phylogenetic Tree Three-domains (Fig 1)

ML phylogenetic Tree Eukaryota Clade (Fig 2)

ML phylogenetic Tree Land Plants Clade I (Fig 3)

ML phylogenetic Tree Land Plants Clade II (Fig 4)

ML phylogenetic Tree Land Plants Clade IV (Fig 5)

**Alignment Three-domains (Fig 1)**

>Pl_Sm4

---------------------------------------------------------------------------------------------------------------------------------------------------------------------MAPTMLSSNDSSSSSPTVLVV-------------------GAGIS---------GLAAARMLHKAA--------FK--------VTVLESR-DRIGGRIYTDFSFG---------------------------FPVDM-GASWLHGVC---------------QDNPLASL--I-GR---LRLPLYRTS-FYQESGLQALASATMTEIYL-----------------PEI--VVVLCYALFDTAGNQIPPQLV-------------TRMGEVFEALLEETK---------------------------------KVREEFAQDMSLKQAFSIILKRRPD-----------------------------LRQEGLGHRVLQWYL-CR--LEGWF--AADADKISL-------QNW--DE----E-----------------------------ELLEGGHGLMVKGYWPVVFSL--AEG---------------------------------LDIKLNHR----VTKISRHP---------------------------------------------------KGVRVAV-------------------------------------------------------ENG---K-----VFNADAIVVAAPLGVLQAKI--------------------------INFEPQLPD---WKVKAIN-ELGVGNENKIAMLFD--N-----VFWPNVEFLGV-----------------------------------------------------V--------------------------------------------------------------------------------------------------ASTTYECSYFL-----------------------------------------------------------------------NL-HKATGHPVLVYMPAGNLANDLEK----LSE------SAAK----NYAF-SQLKKILPNA--------------------------------SLPTKCLVSHWGSDVNS-LGCYSY-DAVGV----------------S--HG-AYD---RLRAPVD--------------------------NLVFFAGEATSS--SFPGTVHGAFATGVLAAAECRKTIEERCKDLE---LFQP--AMAEE------------------IELAIPLQISR------L--------------------------------------------------------------------------------------------------------------------------------------------------------------------------------------------------------------------------------------------------------------------------------------------------------------

>Pl_Sm3

--------------------------------------------------------------------------------------------------------------------------------------------------------------------MAPASQNKGGHVHDGPPSVIVV-------------------GAGVA---------GLAAARALHKAS--------FK--------VTVLESR-GRIGGRVHTDYSFG---------------------------FPVDM-GASWLHGVS---------------EDNPLASV--I-GR---LGLPLYRTS-GDNSV--------------------------------LYD--HDLESYALFDMDGVQVPPELV-------------FRVGESFEALLEMTK---------------------------------KIREEFPEDISVSKAFSVVLERHPE-----------------------------LRQEGLARKVLEWYL-CR--LEGWF--GADADQISV-------RCW--DE----E-----------------------------ELLEGGHGLMVRGYFPVVRHL--AEG---------------------------------IDIRLNHR----VVQVIRHS---------------------------------------------------QGVKIVT-------------------------------------------------------EDS---Q-----VFEADAAVVAVPAGVLKAKI--------------------------IRFEPRLPA---WKEEAFA-GLGLGNENKIALCFD--V-----VFWPNVEFLGV-----------------------------------------------------V--------------------------------------------------------------------------------------------------ASTTYSCSYFL-----------------------------------------------------------------------NL-HKPTGHPVLVYMPAGRLADDIEK----LSD------MEAA----SFAF-GLLQKILPNA--------------------------------AKPVKFLVSRWGSDINS-LGCYTY-DMVGK----------------Q--HE-LYE---QLRAPVD--------------------------T-LFFAGEATSA--SFPGTVHGAFATGALAASECRKGLVERGKCLE---LFQP--AMAA--------------------EDMRPLQISR------L--------------------------------------------------------------------------------------------------------------------------------------------------------------------------------------------------------------------------------------------------------------------------------------------------------------

>Pm_Sb2

-------------------------------------------------------------------------------------------------------------------------------------------------------------------MANNSSFGENARRKPHTPTAIVI-------------------GGGFA---------GLAAADALRNAS--------FQ--------VILLESR-DRIGGRVHTDYSFG---------------------------FPVDL-GASWLHGVC---------------EENPLAPI--I-GR---LGLPLYRTS-GDDSV--------------------------------LFD--HDLESYALYDTNGRQVPQELV-------------EKIGKVFETILEETG---------------------------------KLREGTNEDMSIAKAIAIVMDRNPH-----------------------------FRQEGIAHEVLQWYL-CR--MEGWF--ATDADSISL-------QGW--DQ----E-----------------------------VLLPGGHGLMVRGYRPVINTL--AKG---------------------------------LDIRLNHK----VVEIVRHR---------------------------------------------------NRVEVTV-------------------------------------------------------SSG---K-----TFVADAAVVAVPLGVLKAQT--------------------------IKFEPRLPD---WKEEAIR-ELTVGIENKIVLHFG--Q-----VFWPNVEFLGV-----------------------------------------------------V--------------------------------------------------------------------------------------------------SSSTYGCSYFL-----------------------------------------------------------------------NL-HKATGHPVLVYMPAGRLARDIEK----MSD------EAAA----QFAF-SQLKKILPNA--------------------------------AEPINYLVSHWGSDENS-LGSYTF-DGVNK----------------P--RD-LYE---KLRIPVD--------------------------N-LFFAGEATSL--KYTGTVHGAFSTGVMAAEECKMRVLERFRELDMLEMCHP--AMGED------------------SPVSVPLLISR------L--------------------------------------------------------------------------------------------------------------------------------------------------------------------------------------------------------------------------------------------------------------------------------------------------------------

>Pm_Hv4

------------------------------------------------------------------------------------------------------------------------------------------------------------------MANNNSSYGENVSRKSHTPSAIVI-------------------GGGFA---------GIAAANALRNAS--------FE--------VVLLESR-DRIGGRVHTDYSFG---------------------------FPVDL-GASWLHGVC---------------EENPLAPI--I-GR---LGLPLYRTS-GDDSV--------------------------------LFD--HDLESYALYDTNGSQVPQEFV-------------EEIGKVFEAILEETG---------------------------------KLREEMKEDISIAKAIAIVLERNPH-----------------------------LRREGIAHDVLQWYL-CR--MEGWF--ATDADAISL-------QCW--DQ----E-----------------------------VLLPGGHGLMVRGYRPVINTL--AKG---------------------------------LDIRLGHR----VVEIVRHW---------------------------------------------------NRVEVTV-------------------------------------------------------SNG---K-----TFVADAAVITVPLGVLKSNT--------------------------IKFEPRLPE---WKEEAIR-ELSVGVENKIVLHFS--E-----VFWPNVEFLGV-----------------------------------------------------V--------------------------------------------------------------------------------------------------SSTTYGCSYFL-----------------------------------------------------------------------NL-HKATGHAVLVYMPAGRLACDIEK----MSD------EAAA----QFAF-SQLKKILPNA--------------------------------AEPLNYLVSHWGSDENT-LGSYTF-DGVGK----------------P--RD-LYE---KLRIPVD--------------------------N-LFFAGEATSV--QYTGTVHGAFSTGEMAAEECRMRVLEKFRELDMLEMCHP--MA-EQT-----------------ATVSVPLLISR------L--------------------------------------------------------------------------------------------------------------------------------------------------------------------------------------------------------------------------------------------------------------------------------------------------------------

>Pm_Os3

-------------------------------------------------------------------------------------------------------------------------------------------------------------------MANNSSYGENVRRKSHTPSAIVI-------------------GSGFA---------GIAAANALRNAS--------FE--------VVLLESR-DRIGGRIHTDYSFG---------------------------FPVDL-GASWLHGVC---------------EENPLAPI--I-GR---LGLPLYRTS-GDDSV--------------------------------LFD--HDLESYALYDTKGHQVPQELV-------------EKIGKVFETILEETG---------------------------------KLREETKEDISIAKAIAIVMERNPH-----------------------------LRQEGIAHDVLQWYL-CR--MEGWF--ATDADAISL-------QGW--DQ----E-----------------------------VLLPGGHGLMVRGYRPVINTL--AKG---------------------------------LDIRLGHR----VVEIVRHR---------------------------------------------------NRVEVTV-------------------------------------------------------SSG---K-----TFVADAAVIAVPLGVLKANT--------------------------IKFEPRLPE---WKEEAIR-ELSVGVENKIILHFS--E-----VFWPNVEFLGV-----------------------------------------------------V--------------------------------------------------------------------------------------------------SSTTYGCSYFL-----------------------------------------------------------------------NL-HKATGHPVLVYMPAGRLACDIEK----LSD------EAAA----QFAF-SQLKKILPNA--------------------------------AEPIHYLVSHWGSDENT-LGSYTF-DGVGK----------------P--RD-LYE---KLRIPVD--------------------------N-LFFAGEATSV--QYTGTVHGAFSTGLMAAEECRMRVLERFRELDMLEMCHP--AMGEQT-----------------ATVSVPLLISR------L--------------------------------------------------------------------------------------------------------------------------------------------------------------------------------------------------------------------------------------------------------------------------------------------------------------

>Pm_Ma4

-----------------------------------------------------------------------------------------MCVSYKWNPSSRGKG----------------SVSVRR--VAGAAIA------GLGVME---------NRCDINGVKKASYYLNAEEQRPRSPSAIVI-------------------GGGFA---------GIAAAYALKNAS--------FK--------VVLLESR-DRIGGRVYTDYSFG---------------------------FPVDM-GAAWLHGVC---------------KENPLAPW--I-GR---LGLPIYRTS-GDNSV--------------------------------LFD--HDLESYALFDADGRQVPQELV-------------QKVGE--------TD---------------------------------KLRHETSEDMSIAQAITLVLERHPD-----------------------------LRQEGVANNVLQWYL-CR--MEGWF--ATDANNISV-------KNW--DK----E-----------------------------VLLPGGHGLMVRGYRPVINTL--AKG---------------------------------LDIRLHHQ----VTKIVRGK---------------------------------------------------KGVEVTV-------------------------------------------------------SSG---K-----AFFADAAIITVPLGVLKAKS--------------------------IKFEPRLPE---WKEEAID-GIGVGTENKIVLHFN--K-----VFWPNVEFLGV-----------------------------------------------------V--------------------------------------------------------------------------------------------------SPTSYGCSYFL-----------------------------------------------------------------------NL-HKATGHPVLVYMPAGRLANDIEK----MSD------KAAA----EFAF-SQLKGILPDA--------------------------------SEPIQYLVSHWGTDENS-LGSYTY-DAVGK----------------P--RE-YFE---RLRIPVD--------------------------N-IFFAGEATSI--KYTGTVHGAFSTGLMAAEECRMRVLEKYGDLDTLEMFHP--AMGEEA-----------------ASISVPLLISR------M--------------------------------------------------------------------------------------------------------------------------------------------------------------------------------------------------------------------------------------------------------------------------------------------------------------

>Pm_Ma2

-------------------------------------------------------------------------------------------------------------------------------------------------ME---------NRCDVNGVNKASCYLNSERKQSRSPSAIVI-------------------GGGFA---------GIAAAHALKNAS--------FQ--------VVLLESR-DRIGGRVYTDHSFG---------------------------FPVDM-GAAWLHGVC---------------KENPLATW--I-GR---LGLPIYQTS-GDNSV--------------------------------LFD--HDLESYALFDADGRQVPQELV-------------QKVGE--------AN---------------------------------KVRHETTEDMSVAQAIALVLERDPD-----------------------------LRQEGLANNVLQWYL-CR--MEGWF--ATDADNISL-------KNW--DQ----E-----------------------------VLLPGGHGLMVRGYRPIINTL--AKG---------------------------------LDIRLSHR----VTKIVRGK---------------------------------------------------KGVEVTV-------------------------------------------------------NND---K-----SFFADAAIITVPLGVLKAKS--------------------------IKFEPRLPE---WKEAAID-GIGVGVENKIVLHFD--K-----VFWPNVEFLGV-----------------------------------------------------V--------------------------------------------------------------------------------------------------SSTSYGCSYFL-----------------------------------------------------------------------NL-HKATGHPVLVYMPAGRLAQDIEK----MSD------ESAA----KFAF-SQLKVILPDV--------------------------------TEPIQYLVSRWGRDENS-LGSYSY-DAVGK----------------P--RD-LFE---RLRIPVD--------------------------N-LFFAGEATSI--KYTGTVHGAFSTGLMAAEECRMRVLEKYGDLENLEMFHP--SMDEEA-----------------ASISVPLLISR------M--------------------------------------------------------------------------------------------------------------------------------------------------------------------------------------------------------------------------------------------------------------------------------------------------------------

>Pm_Ma3

-----------------------------------------------------------------------------------------MAVGPELNVLCRSAN----------NSLVLKDWKTCT--VCVTV-----------LQN---------MVGTSKEIDRWASKCFVERKQSRSPSAIVI-------------------GGGFA---------GIAAAHALKNAS--------FQ--------VVLLESR-DRIGGRVYTDHSFG---------------------------FPVDM-GAAWLHGVC---------------KENPLATW--I-GR---LGLPIYQTS-GDNSV--------------------------------LFD--HDLESYALFDADGRQVPQELV-------------QKVGE--------AN---------------------------------KVRHETTEDMSVAQAIALVLERDPD-----------------------------LRQEGLANNVLQWYL-CR--MEGWF--ATDADNISL-------KNW--DQ----E-----------------------------VLLPGGHGLMVRGYRPIINTL--AKG---------------------------------LDIRLSHR----VTKIVRGK---------------------------------------------------KGVEVTV-------------------------------------------------------NND---K-----SFFADAAIITVPLGVLKAKS--------------------------IKFEPRLPE---WKEAAID-GIGVGVENKIVLHFD--K-----VFWPNVEFLGV-----------------------------------------------------V--------------------------------------------------------------------------------------------------SSTSYGCSYFL-----------------------------------------------------------------------NL-HKATGHPVLVYMPAGRLAQDIEK----MSD------ESAA----KFAF-SQLKVILPDV--------------------------------TEPIQYLVSRWGRDENS-LGSYSY-DAVGK----------------P--RD-LFE---RLRIPVD--------------------------N-LFFAGEATSI--KYTGTVHGAFSTGLMAAEECRMRVLEKYGDLENLEMFHP--SMDEEA-----------------ASISVPLLISR------M--------------------------------------------------------------------------------------------------------------------------------------------------------------------------------------------------------------------------------------------------------------------------------------------------------------

>PbPp1

--------------------------------------------------------------------------------------------------------------------------------------------------------------MAPAEQLKTRSESGSSGVRSTTPTVIVI-------------------GAGFG---------GLAAARFLYNSN--------VK--------VVVLESR-ERIGGRVYTDYSFG---------------------------FPVDM-GASWLHGVC---------------KDNPLAPV--I-GK---LRLPLYRTC-GDNSV--------------------------------LYD--HDLESYALFDMDGHQVPQSLV-------------TEVGEVFESLLEETK---------------------------------KLRDEHSDDMSVMKAFTLVLEKRPD-----------------------------LRQEGMAFKVLQWYL-CR--MEGWF--AADADNISV-------QSW--DE----E-----------------------------ELLQGGHGLMVKGYEPVISSL--AEG---------------------------------LDIRFNHRQVAWVTKISRRL---------------------------------------------------HGVRVGT-------------------------------------------------------EDG---K-----VFEADACVVALPLGVLKANV--------------------------VRFEPRLPE---WKEAAIA-DLGVGNENKIALFFE--E-----VCWPNVEFLGV-----------------------------------------------------V--------------------------------------------------------------------------------------------------APTSYGCSYFL-----------------------------------------------------------------------NL-HKATGHPVLVYMPAGRLANDIEQ----LSN------EAAA----NFAI-RQLKRILPNA--------------------------------AEPIKYLVSRWGTDPNS-RGCYSY-DAVGK----------------P--HD-LYE---RLRTPVD--------------------------N-LFWAGEATSE--RFPGTVHGAFHTGVMAGSECLKRFAERCRDLE---MFQP--VMAKE------------------DELITPLLISR------M--------------------------------------------------------------------------------------------------------------------------------------------------------------------------------------------------------------------------------------------------------------------------------------------------------------

>Pd_At3

----------------------------------------------------------------------------------------------------------------------------------------------MESGG----------KTNRQLRKAICVSTDEKMKKKRSPSVIVI-------------------GGGMA---------GISAARTLQDAS--------FQ--------VVVLESR-DRIGGRVHTDYSFG---------------------------FPVDL-GASWLHGVC---------------KENPLAAV--I-GR---LGLPLYRTS-GDNSV--------------------------------LYD--HDLESYALFDKAGNQVSQELV-------------TKVGENFEHILEEIC---------------------------------KVRDEQDEDMSIAQAFSIVFKRNPE-----------------------------LRLEGLAHNVLQWYL-CR--MEGWF--AADAETISA-------KCW--DQ----E-----------------------------ELLPGGHGLMVRGYRPVINTL--SKG---------------------------------LDIRLSHR----ITKISRRY---------------------------------------------------SGVKVTT-------------------------------------------------------EKG---D-----TFVADAAVIALPLGVLKSGM--------------------------ITFEPKLPQ---WKQEAIN-DLGVGIENKIILNFD--N-----VFWPNVEFLGV-----------------------------------------------------V--------------------------------------------------------------------------------------------------AETSYGCSYFL-----------------------------------------------------------------------NL-HKATSHPVLVYMPAGQLARDIEK----KSD------EAAA----NFAF-SQLQKILPDA--------------------------------SSPINYLVSRWGSDINS-LGSYSY-DIVNK----------------P--HD-LYE---RLRVPLD--------------------------N-LFFAGEATSS--SYPGSVHGAYSTGVLAAEDCRMRVLERYGELE---H-----EMEEE------------------APASVPLLISR------M--------------------------------------------------------------------------------------------------------------------------------------------------------------------------------------------------------------------------------------------------------------------------------------------------------------

>Pd_Br5

----------------------------------------------------------------------------------------------------------------------------------------------MASEG---------KTDPPQLNKDTSVSTLERMKKKRTPSVILI-------------------GAGMA---------GIAAARTLQDAS--------FQ--------VVLLESR-DRIGGRVHTDYSFG---------------------------FPVDL-GASWLHGVC---------------KENPLAAV--I-GR---LGLPLYRTS-GDNSV--------------------------------LYD--HDLESYALFDKAGNQVPQELV-------------TKVGETFEHILEEIC---------------------------------KVRDEQFEDMSISQAFSIVFKRSPE-----------------------------LRLEGIAHNVLQWYL-CR--MEGWF--AADAETISA-------KCW--DQ----E-----------------------------ELLPGGHGLMVRGYRPVINTL--SKG---------------------------------LDIRLNHR----VNKIVRRY---------------------------------------------------NGVKVTT-------------------------------------------------------EKG---D-----TFAADAAVIALPLGVLKSGT--------------------------IEFEPKLPD---WKQEAIN-DLGVGIENKIILNFD--N-----VFWPNVEFLGV-----------------------------------------------------V--------------------------------------------------------------------------------------------------AETSYGCSYFL-----------------------------------------------------------------------NL-HKATNHPVLVYMPAGQLARDIEK----MSD------ESAA----KFAF-SQLQKILPDA--------------------------------SSPIHYLVSRWGSDINS-LGSYSY-DIVNK----------------P--HD-LYE---RLRVPLD--------------------------N-LFFAGEATST--SYPGSVHGAYSTGLLAAEDCRMRVLERYGELE---H-----EIEEE------------------APASVPLLISR------M--------------------------------------------------------------------------------------------------------------------------------------------------------------------------------------------------------------------------------------------------------------------------------------------------------------

>Pd_Br4

----------------------------------------------------------------------------------------------------------------------------------------------MESRK----------NS----DHRQMRR-GGESMKTRSPSVIVV-------------------GSGFA---------GISAARTLQDAS--------FQ--------VTVLESR-DRIGGRVHTDYSFG---------------------------FPVDL-GASWLHGVC---------------KENPLAPV--I-GR---LGLPLYRTS-GDNSV--------------------------------LYD--HDLESYALFDMDGNQVPQELV-------------REVGVTFKQILEEVK---------------------------------KVRDEQDADMSISQAFSIVFSKKPE-----------------------------LKLEGLAHNVLQWYL-CR--MEGWF--AADADTISV-------QCW--DQ----E-----------------------------ELLPGGHGLMVRGYRPVINTL--AKG---------------------------------LDIRLNHR----VTKTVRQY---------------------------------------------------NGVIVTT-------------------------------------------------------EDG---K-----TFVADAAVIAVPLGVLKSGT--------------------------ITFEPKLPD---WKQEAIN-QLGVGIMNKIILRFE--K-----VFWPQVELLGV-----------------------------------------------------A--------------------------------------------------------------------------------------------------AETSYGCSYFL-----------------------------------------------------------------------NL-HKATGHPVLVYMSAGQLARDIEK----MSD------EAAA----SFAL-MQLQRIFHNA--------------------------------MAPVQYLVSRWGSDVNS-LGSYSY-DVVGK----------------P--HD-LSE---RLRVPVD--------------------------N-LFFAGEATSS--SFAGSVHGAYSSGLMAAEECMIRVLD---------LFQL--VMGEE------------------GPASVPLLISR------L--------------------------------------------------------------------------------------------------------------------------------------------------------------------------------------------------------------------------------------------------------------------------------------------------------------

>Pd_At2

----------------------------------------------------------------------------------------------------------------------------------------------MESRK----------NSDRQMRRANCFS-AGERMKTRSPSVIVI-------------------GGGFG---------GISAARTLQDAS--------FQ--------VMVLESR-DRIGGRVHTDYSFG---------------------------FPVDL-GASWLHGVC---------------KENPLAPV--I-GR---LGLPLYRTS-GDNSV--------------------------------LYD--HDLESYALFDMDGNQVPQELV-------------TQIGVTFERILEEIN---------------------------------KVRDEQDADISISQAFSIVFSRKPE-----------------------------LRLEGLAHNVLQWYV-CR--MEGWF--AADAETISA-------KCW--DQ----E-----------------------------ELLPGGHGLMVRGYRPVINTL--AKG---------------------------------LDIRVGHR----VTKIVRRY---------------------------------------------------NGVKVTT-------------------------------------------------------ENG---Q-----TFVADAAVIAVPLGVLKSGT--------------------------IKFGPKLPE---WKQEAIN-DLGVGIENKIILHFE--K-----VFWPKVEFLGV-----------------------------------------------------V--------------------------------------------------------------------------------------------------AETSYGCSYFL-----------------------------------------------------------------------NL-HKATGHPVLVYMPAGQLAKDIEK----MSD------EAAA----NFAV-LQLQRILPDA--------------------------------LPPVQYLVSRWGSDVNS-MGSYSY-DIVGK----------------P--HD-LYE---RLRVPVD--------------------------N-LFFAGEATSS--SFPGSVHGAYSTGLMAAEDCRMRVLERYGELD---LFQP--VMGEE------------------GPASVPLLISR------L--------------------------------------------------------------------------------------------------------------------------------------------------------------------------------------------------------------------------------------------------------------------------------------------------------------

>Pd_Br2

----------------------------------------------------------------------------------------------------------------------------------------------MESRK----------NSDRQMRRANCFS-AGGRMKTRSPSVIVI-------------------GGGFA---------GISAARTLQDAS--------FQ--------VMVLESR-DRIGGRVHTDYSFG---------------------------FPVDL-GASWLHGVC---------------KENPLAPV--I-GR---LGLPLYRTS-GDNSV--------------------------------LYD--HDLESYALFDMEGNQVPQELV-------------TNVGITFEQILEEIN---------------------------------KVRDEQDADMSISQAFSIVFSRKPE-----------------------------LRLEGLAHNVLQWYL-CR--MEGWF--AADADTISA-------KCW--DQ----E-----------------------------ELLPGGHGLMVRGYRPVINTL--AKG---------------------------------IDIRLGHR----VTKIVRRY---------------------------------------------------NGVKVTT-------------------------------------------------------ENG---E-----TFVADAAVIAVPLGVLKSGT--------------------------ITFEPKLPE---WKQEAIN-DLGVGIENKIILHFE--K-----VFWPKVEFLGV-----------------------------------------------------V--------------------------------------------------------------------------------------------------AETSYGCSYFL-----------------------------------------------------------------------NL-HKATGHPVLVYMPAGQLAKDIEK----MSD------EAAA----SFAV-LQLQRILPDA--------------------------------LPPVQCLVSRWGSDVNS-LGSYSY-DIVGK----------------P--HD-LYE---RLRVPVD--------------------------N-LFFAGEATSS--SFPGSVHGAYSTGLMAGEDCRMRVLERYGELD---LFQP--VMGEE------------------GPASVPLLISR------L--------------------------------------------------------------------------------------------------------------------------------------------------------------------------------------------------------------------------------------------------------------------------------------------------------------

>Pd_Br3

----------------------------------------------------------------------------------------------------------------------------------------------MESRK----------NSD-----RQIRR-AGGRMKTRSPSVIVI-------------------GGGFG---------GISAARTLQDAS--------FQ--------VVVLESR-DRIGGRVHTDYSFG---------------------------FPVDL-GASWLHGVC---------------KENPLAPV--I-GR---LGLPLYRTS-GDNSV--------------------------------LYD--HDLESYALFDMDGKQVPQELV-------------TEVGITFEQILEEIN---------------------------------KVRDEQEADMSISKAFSIVFSRKPE-----------------------------LRLEGLAHNVLQWYL-CR--MEGWF--AADADTISA-------KCW--DQ----E-----------------------------ELLPGGHGLMVRGYRPVINTL--SKG---------------------------------IDIRLGHR----VTKIVRRY---------------------------------------------------NGVKVTT-------------------------------------------------------ENG---E-----TFVADAAVIAVPLGVLKSGT--------------------------ITFEPKLPE---WKQEAIN-DLGVGIENKIILHFE--K-----VFWPKVEFLGV-----------------------------------------------------V--------------------------------------------------------------------------------------------------AETSYGCSYFL-----------------------------------------------------------------------NL-HKATGHPVLVYMPAGQLAKDIEK----MSD------EAAA----NFAV-LQLQRILPDA--------------------------------LPPVQYLVSRWGSDVNS-LGSYSY-DIVGK----------------P--HD-LYE---RLRVPVD--------------------------N-LFFAGEATSS--SFPGSVHGAYSTGLMAGEDCRMRVLERYGELD---LFQP--VMGEE------------------GPASVPLLISR------L--------------------------------------------------------------------------------------------------------------------------------------------------------------------------------------------------------------------------------------------------------------------------------------------------------------

>Pd_Cs4

----------------------------------------------------------------------------------------------------------------------------------------------MESGA---------K-SNSELRTEICYPKPQTQQVRSSPSVIVI-------------------GGGMA---------GVAAARALHDAS--------FQ--------VTLLESR-DRLGGRIHTDYSFG---------------------------FPVDL-GASWLHGAC---------------EENPLAPL--I-GR---LGLPLYRTS-EDNSV--------------------------------LYD--HDLESYALSDTDGSQVPPELV-------------TKVGITFETILKETE---------------------------------TIREEEIEDMSILRAISIVFERRPE-----------------------------LRLEGLAQKVLQWYL-CR--MEGWF--SADANTISL-------KGW--DQ----E-----------------------------ELLPGGHGLMVRGYLPVIHTL--AKG---------------------------------IDIRLGHR----VTKISRQY---------------------------------------------------TGVKITV-------------------------------------------------------ENG---K-----TFKADAAIIAVPLGVLKANV--------------------------IKFEPKLPD---WKEAAIA-EVGVGLENKIILHFE--T-----AFWPNVEFLGV-----------------------------------------------------V--------------------------------------------------------------------------------------------------ADTSKNCSYFL-----------------------------------------------------------------------NL-HKATSHPVLVYMPSGKLARDIEK----MSD------QEAA----NFAF-MQLKKVVPDA--------------------------------PAPIQYLVSRWGSDVNS-LGSYSY-NIVGK----------------P--HH-LFE---RLRIPVD--------------------------N-LFFAGEATSI--HYPGSVHGAYSTGLMAAEDCRMRFLERYGDVD---LLQA--VMVDE------------------APLSAPLLISR------M--------------------------------------------------------------------------------------------------------------------------------------------------------------------------------------------------------------------------------------------------------------------------------------------------------------

>Pd_Ca2

----------------------------------------------------------------------------------------------------------------------------------------------MESRN---------K-SSPKLTTALCYGNVS-SGQERSPSVIVI-------------------GGGMA---------GIAAARALQDAS--------FQ--------VVLLESR-ERLGGRIHTDYSFG---------------------------FPVDM-GASWLHGVS---------------KENPLASV--I-GR---LGLPLYRTS-GDNSV--------------------------------LYD--HDLESYALFDMDGTQVPQELV-------------TKVGQVFENILLETN---------------------------------KVRNEFSEDMSIQRGLSIVFERKPE-----------------------------LRLEGLAHKVLQWYL-CR--MEGWF--AADSDTISL-------NGW--DQ----E-----------------------------ILLPGGHGLMVRGYLPVIHTL--AKG---------------------------------LDIRLGHR----VTKVVRRY---------------------------------------------------NGVKVTV-------------------------------------------------------ENG---K-----SFTADAAVIAVPLGVLKSKR--------------------------ILFEPKLPD---WKEAAIA-ELGVGLENKIILHFE--N-----VFWPNVEFLGV-----------------------------------------------------V--------------------------------------------------------------------------------------------------ADTSYGCSYFL-----------------------------------------------------------------------NL-HKAARHPVLVYMPSGRLAKDIEK----MSD------EATA----NFAF-EQLKKILPDA--------------------------------SSPIQYLVSRWGSDIDS-LGSYSY-DQVGK----------------P--HE-LYE---MLRVPVD--------------------------N-LFFAGEATSV--NYPGSVHGAFSTGMMAAEDCRMRVLERYGELD---LFQP--VMGEM------------------ASFTIPLQISR------M--------------------------------------------------------------------------------------------------------------------------------------------------------------------------------------------------------------------------------------------------------------------------------------------------------------

>Pd_Gm4

----------------------------------------------------------------------------------------------------------------------------------------------MESRT---------K-SNPQLTRALCYGNDG-KQQGRSPSVIVI-------------------GGGMA---------GIAAARALHNAS--------FQ--------VVLLESR-DRIGGRIHTDYSFG---------------------------FPVDL-GASWLHGVS---------------NENPLASV--I-GR---LGLPLYRTS-GDNSV--------------------------------LYD--HDLESYALFDMDGKQVPPELV-------------TKVGEIFETILQETD---------------------------------KIRQESSEDMSVLRGLSIVFDRKPE-----------------------------LRLEGLAHKVLQWYL-CR--MEGWF--AADSDTISL-------KGW--DQ----E-----------------------------VLLPGGHGLMVRGYLPVINTL--AKG---------------------------------LDILLGHR----VTKVVRRY---------------------------------------------------NGVKVTV-------------------------------------------------------ESG---K-----TFFADAAVIAVPLGVLKAKK--------------------------ILFKPKLPD---WKEAAIA-DLGIGLENKIILHFE--N-----VFWPNVEFLGV-----------------------------------------------------V--------------------------------------------------------------------------------------------------ADTSYGCSYFL-----------------------------------------------------------------------NL-HKAAGHAVLVYMPSGQLAKDVEK----MSD------EAAV----NFAF-MQLKKILPDA--------------------------------SSPIQYLVSRWGSDINS-LGSYSY-DAVGK----------------P--HE-LYE---RLRVPVD--------------------------N-LFFAGEATSM--SYPGSVHGAFSTGMMAAEDCRMRVLERYGEVD---LFQP--VMGEE------------------ASLSIPLQISR------L--------------------------------------------------------------------------------------------------------------------------------------------------------------------------------------------------------------------------------------------------------------------------------------------------------------

>Pd_Gm5

----------------------------------------------------------------------------------------------------------------------------------------------MGFRF---------K-SNPQLRRGLCCANDD-KQQERSPSVIVI-------------------GGGMA---------GIAAARALQDAS--------FQ--------VILLESR-ERLGGRIHTDYSFG---------------------------FPVDL-GASWLHGVC---------------KENPLAPL--I-GK---LGLPLYRTS-EDNSV--------------------------------LYD--HDLESYALFDMDGNQVPQELV-------------TKIGKIFGVILEETN---------------------------------NVREEFSEDMSILRALSIVFERKPE-----------------------------LRLEGLSHKVLQWYL-CR--MEGWF--ATDADTISL-------KCW--DQ----E-----------------------------VLLPGGHGLMVRGYQPVINTL--AKG---------------------------------LDIRQGHR----VTKIVRQY---------------------------------------------------NEVKVAV-------------------------------------------------------ENG---K-----TFVADAAIVAVPLGVLKAKS--------------------------IKFEPKLPD---WKEAAIS-DIGVGIENKIILHFK--N-----VFWPNVEFLGV-----------------------------------------------------V--------------------------------------------------------------------------------------------------AETSYGCSYFL-----------------------------------------------------------------------NL-HKATGRPVLVYMPAGQLAKDIEK----MSD------EAAA----SFAF-MQLKKILPDT--------------------------------SSPIQYLVSRWGTDINT-LGSYSY-DAVGK----------------P--HD-LYE---RLRVPVD--------------------------N-LFFAGEATSM--LYTGSVHGAYSTGMMAAEDCRMRVLERYGELD---LVPP--VMGED------------------A-SVIPLQISR------L--------------------------------------------------------------------------------------------------------------------------------------------------------------------------------------------------------------------------------------------------------------------------------------------------------------

>Pd_Gm6

----------------------------------------------------------------------------------------------------------------------------------------------MESRF---------K-SNPQLRRGLCCANDD-KQQERSPSVIVI-------------------GGGMA---------GIAAARALQDAS--------FQ--------VILLESR-ERPGGRIHTDYSFG---------------------------FPVDL-GASWLHGVC---------------PENPLAPL--I-GK---LGLPLYRTS-EDNSV--------------------------------LYD--HDLESYALFDMDGNQVPQELV-------------TKIGKIFGAILEETN---------------------------------NVREEFSEDMSILRALSIVFERKPE-----------------------------LRLEGLSHKVLQWYL-CR--MEGWF--ATDADTISL-------KCW--DQ----E-----------------------------VLLPGGHGLMVRGYQPVINTL--AKG---------------------------------LDIRLGHR----VTKIVRQY---------------------------------------------------NEVKVTV-------------------------------------------------------ENG---K-----TFVADAAIVAVPLGVLKAKS--------------------------IKFEPKLPD---WKEAAIS-DIGVGIENKIILHFK--N-----VFWPNVEFLGV-----------------------------------------------------V--------------------------------------------------------------------------------------------------AETSYGCSYFL-----------------------------------------------------------------------NL-HKAMGRPVLVYMPAGQLAKDIEK----MSD------EAAA----NFAF-MQLKKILPDA--------------------------------SSPIQYLVSRWGTDINT-LGSYSY-DAVGK----------------P--HD-LYE---KLRVPVD--------------------------N-LFFAGEATSM--LYTGSVHGAYSTGMMAAEDCRMRVLERYGELD---LFPP--VG--D------------------V-SVIPLQISR------L--------------------------------------------------------------------------------------------------------------------------------------------------------------------------------------------------------------------------------------------------------------------------------------------------------------

>Pd_Ca3

----------------------------------------------------------------------------------------------------------------------------------------------MESRI---------K-TNPQSRKGFYYANVD-QQHARSPSVIVI-------------------GGGMA---------GVAAARALHDAS--------FQ--------VVLLESR-DRLGGRIHTDYSFG---------------------------FPVDL-GASWLHGVC---------------KENPLAPL--I-GR---LGLPLYRTC-EDNSV--------------------------------LYD--HDLESYALFDMDGKQVPQELV-------------TEVGKIFEMILQETD---------------------------------NVRQEFSDDMSILHALSIVFERKPE-----------------------------LRLDGLSHKVLQWYL-CR--MEGWF--AADADAISL-------KCW--DQ----E-----------------------------ELLPGGHGLMVRGYLPVIHTL--AKG---------------------------------LDIRLGHR----VTKIARRY---------------------------------------------------NGIKVTV-------------------------------------------------------ENG---K-----TFVADAAIIAVPLGVLKANI--------------------------IKFEPKLPD---WKEAAIA-DIGVGVENKIILHFK--N-----VFWPNVEFLGV-----------------------------------------------------V--------------------------------------------------------------------------------------------------ADTSYGCSYFL-----------------------------------------------------------------------NL-HKAAGHPVLVYMPAGRLAKDIEK----MSD------EAAA----NFAF-TQLKKILPDA--------------------------------SSPIQYLVSRWGTDINS-LGSYSF-DAVGK----------------P--HG-LYE---RLRVPVD--------------------------N-LFFAGEATSV--LYTGSVHGAFSTGTMAAEDCRMRVLERYGELD---LFQP--VLDE-------------------G-SVIPLLISR------F--------------------------------------------------------------------------------------------------------------------------------------------------------------------------------------------------------------------------------------------------------------------------------------------------------------

>Pd_Mt2

----------------------------------------------------------------------------------------------------------------------------------------------MESRI---------K-TNPNSRKGLCYVNVD-QQPRRSPSVIVI-------------------GGGMA---------GIAAARALHDAS--------FQ--------VVLLESR-DRIGGRIHTDYSFG---------------------------FPVDL-GASWLHGVC---------------NENPLAPL--I-GR---LGLPLYRTC-EDNSV--------------------------------LYD--HDLESYALFDMEGNQVPQELV-------------TEVGKTFEMILQETD---------------------------------NVRQEFSEDMSILRALSIVFERKPE-----------------------------LRLEGLSHKVLQWYL-CR--MEGWF--AADSDSISL-------KCW--DQ----E-----------------------------ELLPGGHGLMVRGYLPVIHTL--AKG---------------------------------LDIRLGHR----ATKIVRGY---------------------------------------------------NGVKVTT-------------------------------------------------------ENG---K-----TFVADAAIIAVPLGVLKANV--------------------------IKFEPKLPD---WKEAAIA-DIGVGVENKIILHFK--N-----VFWPNVEFLGV-----------------------------------------------------V--------------------------------------------------------------------------------------------------AETSYGCSYFL-----------------------------------------------------------------------NL-HKAAGHPVLVYMPAGRLAKDIEK----MSD------EAAA----DFAF-TQLKKILPDA--------------------------------SSPIQYLVSRWGTDINS-LGSYSF-DAVGK----------------P--HG-LYE---RLRVPVD--------------------------N-LFFAGEATSV--LYTGSVHGAYSTGTMAAEDCRMRVLERYGELD---IFQP--ELEE-------------------G-SVIPLLISR------I--------------------------------------------------------------------------------------------------------------------------------------------------------------------------------------------------------------------------------------------------------------------------------------------------------------

>Pd_Cs3

----------------------------------------------------------------------------------------------------------------------------------------------MESGS---------RS-NSQLRKAVCHS-GPEKGQVRSPSVIVI-------------------GGGIA---------GVAAARALHDAS--------FQ--------VILLEAR-ERLGGRIYTNYSFG---------------------------FPVDL-GASWLHGVC---------------KENPLAPL--I-GK---LGLPLYRTS-EDNSV--------------------------------LYD--HDLESYALFDMEGKQVPQELV-------------TKVGQVFEAVLEEAD---------------------------------KIRDEYTEDMTITRAFSIIFERRPE-----------------------------LKMDGLAHKVLQWYL-CR--MEGWF--AADANTISL-------KCW--DQ----E-----------------------------ELLPGGHGLMVRGYLPVINTL--AKG---------------------------------LDIRLGHR----VSKVVRRY---------------------------------------------------NEIKVTV-------------------------------------------------------ENG---T-----TFVADAAIVAVPLGVLKANT--------------------------IEFEPKLPD---WKESAIS-DLGVGVENKIILHFE--Q-----VFWPNVEFLGV-----------------------------------------------------V--------------------------------------------------------------------------------------------------AETTYECSYFL-----------------------------------------------------------------------NL-HKATGHSVLVYMPAGQLAEDIEK----LSD------EAAA----NFAF-TQLKKILPDA--------------------------------SDPINFLVSRWGTDVDT-LGSYSY-DIVGK----------------P--HD-LYE---KLRIPID--------------------------N-IFFAGEATST--SFPGSVHGAFATGVMAAEDCRMRVLERYGELN---IFQP--VLAEE-------------------PVSVPLLISR------L--------------------------------------------------------------------------------------------------------------------------------------------------------------------------------------------------------------------------------------------------------------------------------------------------------------

>Pd_Cc3

----------------------------------------------------------------------------------------------------------------------------------------------MDSAS---------R-SNRQLRRALCYSNNAGKGQARSPSVIVI-------------------GAGMA---------GVAAARALHDAS--------FK--------VVLLESR-DRVGGRVHTDYSFG---------------------------FPVDL-GASWLHGVC---------------QENPLAPV--I-SR---LGLPLYRTS-GDNSV--------------------------------LYD--HDLESYALFDMDGNQVPQELV-------------TKVGEAFESILKETD---------------------------------KVREEHDEDMSIQRAISIVFDRRPELRFFEHVSSSLPGISLQRKLLDLLKLVLTCRLEGLAHKVLQWYL-CR--MEGWF--AADAETISL-------KSW--DK----E-----------------------------ELLPGGHGLMVRGYLPVINTL--AKG---------------------------------LDIRLGHR----VTKITRHY---------------------------------------------------IGVKVTV-------------------------------------------------------EGG---K-----TFVADAVVVAVPLGVLKART--------------------------IKFEPRLPD---WKEAAID-DLGVGIENKIIMHFD--K-----VFWPNVEFLGV-----------------------------------------------------V--------------------------------------------------------------------------------------------------SDTSYGCSYFL-----------------------------------------------------------------------NL-HKATGHCVLVYMPAGQLARDIEK----MSD------EAAA----NFAF-TQLKKILPDA--------------------------------SSPIQYLVSHWGTDANS-LGSYSY-DTVGK----------------S--HD-LYE---RLRIPVD--------------------------N-LFFAGEATSM--SYPGSVHGAFSTGLMAAEDCRMRVLERYGELD---LFQP--VMGEE------------------TPISVPFLISR------L--------------------------------------------------------------------------------------------------------------------------------------------------------------------------------------------------------------------------------------------------------------------------------------------------------------

>Pd_Vv5

----------------------------------------------------------------------------------------------------------------------------------------------MESSE---------RS-NRQLRRALCYANIE-RQQATSPSVIVI-------------------GAGMA---------GIAAARALHDAS--------FR--------VVLLESR-DRIGGRVHTDYSFG---------------------------FPVDL-GASWLHGVC---------------KENPLAPL--I-SR---LGLPLYRTS-GDNSV--------------------------------LYD--HDLESYALFDMDGNQVPQELV-------------REIGVAFEKILEETD---------------------------------KVRQEHSEDMPILDAFKIVFERRPD-----------------------------LRLEGLAHKVLQWYL-CR--MEGWF--AADADNISL-------KSW--DQ----E-----------------------------ELLPGGHGLMVRGYIPVINTL--AKG---------------------------------LDIHLNHR----VTKIVRRY---------------------------------------------------NGVKVTV-------------------------------------------------------EDG---R-----SFVADAAIVAVPIGVLKSSR--------------------------IKFEPRLPE---WKEEAIA-DIGVGIENKIALHFD--K-----VFWPNVEFLGV-----------------------------------------------------V--------------------------------------------------------------------------------------------------ADTSYGCSYFL-----------------------------------------------------------------------NL-HKATSHSVLVYMPAGQLAKDIEK----MSD------EAAA----NFAF-MQLKKILPEA--------------------------------SDPIQYLVSRWGTDENS-LGSYTY-DAVGK----------------P--HD-LYE---RLRVPVD--------------------------N-LFFAGEATSV--NYPGSVHGAFSTGTLAAEECRMRVLERYGELD---LFQP--AMGEE------------------TSFSIPLQISR------M--------------------------------------------------------------------------------------------------------------------------------------------------------------------------------------------------------------------------------------------------------------------------------------------------------------

>Pd_Pt3

----------------------------------------------------------------------------------------------------------------------------------------------MDSGF---------KSNRPQLRRGLCYSNEG-RGQARSPSVIVI-------------------GGGIA---------GVAAARALHDAS--------FQ--------VVLLESR-DRLGGRVHTDFSFG---------------------------FPVDL-GASWLHGVC---------------KENPLAPL--I-GR---LGLPLYRTS-GDNSV--------------------------------LYD--HDLESYALFDMDGNQVPQELV-------------TKVGEAFENILKEACISSFLFSPLLSTLPNIITLLDH-----------KVRLENNEDMSILRAFSIVFERRPD-----------------------------LRLEGLALKVLQWYL-CR--MEGWF--AADSETISL-------KCW--DQ----E-----------------------------ELLPGGHGLMVRGYLPVINTL--AKG---------------------------------LDIRLSHR----VKKIVRRY---------------------------------------------------NGVKVTV-------------------------------------------------------EDG---S-----TFMADAAVVAVPLGVLKSKT--------------------------ITFEPELPD---WKEKAIK-DLGVGIENKIVLNFD--H-----VFWPNVEFLGV-----------------------------------------------------V--------------------------------------------------------------------------------------------------AETSYGCSYFL-----------------------------------------------------------------------NL-HKATGHPVLVYMPAGKLARDIEK----MSD------EAAA----NFAF-TQLKKILPDA--------------------------------SAPIKYLVSRWGSDINS-LGSYSY-DTVGK----------------S--HD-LYE---RLRIPID--------------------------N-LFFAGEATSI--SYPGSVHGAFSTGLMAAEACRMRVLERYGELD---IFQP--VMGEE------------------ATVSVPLLISR------M--------------------------------------------------------------------------------------------------------------------------------------------------------------------------------------------------------------------------------------------------------------------------------------------------------------

>Pd_Pt4

------------------------------------------------------------------------------------------------------------------------------------------------------------------MGTGLCYSNEGRGQATRSPSVIVI-------------------GGGIA---------GVAAARALHDAS--------IQ--------VVLLESR-DRLGGRVHTDFSFG---------------------------FPVDL-GASWLHGVC---------------KENPLAPL--I-GR---LGLPLYRTS-GDNSV--------------------------------LYD--HDLESYALYDMDGNQVPQELV-------------TKVGEAFENILKETD---------------------------------KVRLENNEDMSILRAFSIVFERRPD-----------------------------LRLEGLAHKVLQWYL-CR--MEGWF--AADSETISL-------KGW--DQ----E-----------------------------ELLPGGHGLMVRGYLPVINTL--AKG---------------------------------LDIRLGHR----VTKIVRHY---------------------------------------------------NGVKVTV-------------------------------------------------------EDG---R-----TFMADAAVVAIPLGVLKSKT--------------------------IMFEPKLPD---WKEEAIK-DLGVGIENKIVLNFE--Q-----VFWPKVEFLGV-----------------------------------------------------V--------------------------------------------------------------------------------------------------AETSYGCSYFL-----------------------------------------------------------------------NL-HKATGHPVLVYMPAGKLARDIEK----MSD------EAAA----NFAF-MQLKKILPDA--------------------------------FAPIQYLVSRWGSDINS-LGSYSY-DTVGK----------------P--HE-LYE---RLRIPVD--------------------------N-LFFAGEATSV--SYPGSVHGAFSTGLMAAEDCRMRVLERYGELD---LFQP--VMGTEE-----------------APVSVPLLISR------I--------------------------------------------------------------------------------------------------------------------------------------------------------------------------------------------------------------------------------------------------------------------------------------------------------------

>Pd_Pr4

----------------------------------------------------------------------------------------------------------------------------------------------------------------------------------------------------------------MA---------GVSAARALHDAS--------IQ--------VMLLESR-DRLGGRVYTDYSFG---------------------------FPIDL-GASWLHGVC---------------KENPLAPL--I-GR---LGLPLYRTS-GDNSV--------------------------------LYD--HDLESYALFDMDGKQVPQDLV-------------TKVGEVFENLLKETD---------------------------------KVREEFSEDMSITRAFSIVFERNPE-----------------------------LRLEGVAHKVLQWYL-CR--MEGWF--AADADTISL-------KCW--DQ----E-----------------------------ELLPGGHGLMVRGYLPVINTL--AKG---------------------------------LDIRLGHR----VTKIARQY---------------------------------------------------NGVHVTI-------------------------------------------------------EDG---S-----TFVADAAVVAVPLGVLKAKS--------------------------ISFEPKLPN---WKEEAID-DLGVGIENKIVLHFE--K-----VFWPNVEFLGV-----------------------------------------------------V--------------------------------------------------------------------------------------------------AETSYCCSYFL-----------------------------------------------------------------------NL-HKATGHSVLVYMPAGQLAKDIEK----MSD------EEAA----NFAF-MQLKKILPDA--------------------------------SSPIQYLVSRWGSDVNT-LGSYSY-DMVGK----------------P--HD-LYE---KLRVPVD--------------------------N-LFFAGEATSA--DFPGSVHGAFSTGMMAAEDCRMRVLERYGELD---LFEP--VMGEE------------------A-MSIPLLISR------I--------------------------------------------------------------------------------------------------------------------------------------------------------------------------------------------------------------------------------------------------------------------------------------------------------------

>Pd_Rc2

----------------------------------------------------------------------------------------------------------------------------------------------MESGL---------RSNRSQLRRGLCFSDAER-REASSRSVIVI-------------------GGGMA---------GIAAARALYDAS--------FQ--------VVLLESR-DRLGGRVHTNYSFG---------------------------FPVDL-GASWLHGVG---------------PENPLAPL--I-GR---LGLPLYRTS-GDNSV--------------------------------LYD--HDLESYALFDMDGNQVPQELV-------------SEVGETFEIILKETE---------------------------------KVRQEYSEDMSISNAFSIVFERRPE-----------------------------LRLEGLAHKVLQWYL-CR--MEGWF--AADADTISL-------KCW--DQ----E-----------------------------ELLPGGHGLMVRGYLPVINTL--AKG---------------------------------LDIRLGHR----VTKIVRRH---------------------------------------------------NGVKVTT-------------------------------------------------------EDG---R-----TFMADAAVIAVPLGVLKSRT--------------------------ITFEPRLPD---WKEEAIK-DLGVGIENKIVLHFD--K-----VFWPNVEFLGV-----------------------------------------------------V--------------------------------------------------------------------------------------------------SETSYGCSYFL-----------------------------------------------------------------------NL-HKATGHSVLVYMPAGQLAKDIEK----MSD------EAAA----NFAF-MQLKKILPEA--------------------------------SDPIQYLVSRWGSDVNS-LGSYSY-DTVGK----------------P--HD-LYE---RLRVPVD--------------------------N-LFFAGEATSA--SYPGSVHGAFSTGLMAAEDCRMRVLERYGELD---LFQP--VMGEE------------------AAVSVPLLISR------M--------------------------------------------------------------------------------------------------------------------------------------------------------------------------------------------------------------------------------------------------------------------------------------------------------------

>Pm_Os4

------------------------------------------------------------------------------------------------------------------------------------------------MDP-------------NSLKTGGLLLPTIERQCASPPSVIVI-------------------GGGIS---------GVAAARALSNAS--------FE--------VTVLESR-DRVGGRVHTDYSFG---------------------------CPIDM-GASWLHGVC---------------NENSLAPL--I-GY---LGLKLYRTS-GDNSV--------------------------------LYD--HDLESYALFDKAGHQVSKETV-------------AKVEETFERILDETV---------------------------------KVRDEQEHDMPLLQAISLVLERHPH-----------------------------LKLQGIDDQVLQWCV-CR--LEAWF--AADADEISL-------KNW--DQ----E-----------------------------HVLTGGHGLMVNGYYPIIQAL--AQG---------------------------------LDIRLNQR----VTKIARQF---------------------------------------------------NGVTVTT-------------------------------------------------------EDG---T-----SYSADACIITVPLGVLKANI--------------------------IKFEPELPS---WKSSAIA-DLGVGIENKIAMHFD--T-----VFWPNVEVLGM-----------------------------------------------------V--------------------------------------------------------------------------------------------------GPTPKACGYFL-----------------------------------------------------------------------NL-HKATGNPVLVYMAAGRFAQEVEK----LSD------KEAV----DLVM-SHLKKMLPDA--------------------------------TEPTKYLVSRWGSDPNS-LGSYSC-DLVGK----------------P--AD-VSA---RFAAPVE--------------------------N-LYFAGEAASA--DHSGSVHGAYSSGIAAADECRKRILMQKGIPDLVQV-----KAYEEM-----------------AGVIAPLQICR------T--------------------------------------------------------------------------------------------------------------------------------------------------------------------------------------------------------------------------------------------------------------------------------------------------------------

>Pm_Sb4

------------------------------------------------------------------------------------------------------------------------------------------------MDP-------------NGLKTGGLLLPTIERRCTSPPSVIVI-------------------GGGIS---------GVAAARALSNSS--------FK--------VTVLESR-DRIGGRVHTDYSFG---------------------------CPIDM-GASWLHGVC---------------NENSLAPL--I-GY---LGLRLYRTS-DDNSV--------------------------------LYD--HDLESYALFDKDGNQVPKETV-------------DKVGETFERILEETV---------------------------------KVRDEQEHDMPLLQAISIVFERHPH-----------------------------LKLEGLDDQVLQWCV-CR--LEAWF--AADADEISL-------KNW--DQ----E-----------------------------RVLTGGHGLMVNGYYPVIEAL--AQG---------------------------------LDIRLNQR----VTEITRQY---------------------------------------------------NGVKVTT-------------------------------------------------------EDG---T-----SYFADACIISVPLGVLKANV--------------------------IKFEPELPS---WKSSAIA-DLGVGVENKIAMHFD--R-----VFWPNVEVLGM-----------------------------------------------------V--------------------------------------------------------------------------------------------------GPTPKACGYFL-----------------------------------------------------------------------NL-HKATGNPVLVYMAAGRFAQEVEK----LSD------KEAV----SLVV-SHLKKMLPDA--------------------------------SEPTQYLVSRWGSDPNS-LGSYSC-DLVGK----------------P--AD-VCA---RFSAPVD--------------------------N-LYFAGEAASA--EHSGSVHGAYSSGIAAAEECRKRLLTLKGIPDLVQV-----AAWEEM-----------------AGAVAPLQICR------T--------------------------------------------------------------------------------------------------------------------------------------------------------------------------------------------------------------------------------------------------------------------------------------------------------------

>Pm_Zm3

------------------------------------------------------------------------------------------------------------------------------------------------MDP-------------NGLKTGGLLLPTIERRCTSPPSVIVI-------------------GGGIS---------GVAAARALSNSS--------FK--------VTVLESR-DRIGGRIHTDNSFG---------------------------CPIDM-GASWLHGVC---------------NENPLAPL--I-SY---LGLRLYRTS-DDNSV--------------------------------LYD--HDLESYALFDKDGNQVPKETV-------------DKVGETFERILEETV---------------------------------KVRDEQEHDMPLLQAISIVFERHPH-----------------------------LKLEGLDDQVLQWCV-CR--LEAWF--AADADEISL-------KNW--DQ----E-----------------------------RVLTGGHGLMVNGYYPVIEAL--AQG---------------------------------LDIRLNQR----VTEITRQH---------------------------------------------------NGVKVTT-------------------------------------------------------EDG---T-----SYLADACIISVPLGVLKANV--------------------------IKFEPELPQ---WKSSAIA-DLGVGTENKIAMHFD--R-----VFWPNVEVLGM-----------------------------------------------------V--------------------------------------------------------------------------------------------------GPTPKACGYFL-----------------------------------------------------------------------NL-HKATGNPVLVYMAAGRFAQEVEK----LSD------KEAV----GLVV-SHLKKMLPDA--------------------------------TEPTQYLVSRWGSDPNS-LGSYSC-DLVAK----------------P--AD-VCA---RFAAPVE--------------------------N-LHFAGEAASA--EHSGSVHGAYSSGIAAAEECRKRLLALKGIPDLVQV-----AAWEEM-----------------AGAVAPLQICR------T--------------------------------------------------------------------------------------------------------------------------------------------------------------------------------------------------------------------------------------------------------------------------------------------------------------

>Pm_Os5

----------------------------------------------------------------------------------------------------------------------------------------------MDQPS-------------NGFAAGGLFLRHIDGQNASPPSVIVI-------------------GGGIS---------GIAAARALSNAS--------FK--------VTLLESR-DRLGGRVHTDYSFG---------------------------CPIDM-GASWLHGVC---------------NENSLAPL--I-RL---LGLRLYRTS-GDNSV--------------------------------LYD--HDLESYALFDKDGRQVPQEIV-------------TKVGETFEKILKETV---------------------------------KVRAEHEDDMPLIQAISIVLDRNPH-----------------------------LKLDGLQYEVLQWCI-CR--LEAWF--ATDVDNISL-------KNW--DQ----E-----------------------------HVLTGGHGLMVHGYDPVIKAL--AQD---------------------------------LDIHLNHR----VTKIIQRY---------------------------------------------------NKTIVCV-------------------------------------------------------EDG---T-----SFVADAAIITVPLGVLKANI--------------------------IKFEPELPD---WKLSSIS-DLGIGIENKIALRFN--S-----VFWPNVEVLGR-----------------------------------------------------V--------------------------------------------------------------------------------------------------APTSNACGYFL-----------------------------------------------------------------------NL-HKATGHPVLVCMVAGRFAYEFEK----LSD------EESV----NFVM-SQLKKMLPGA--------------------------------TEPVQYLVSRWGTDPNS-LGSYSC-DLVGK----------------P--AD-LYE---RFCAPVG--------------------------N-LFFAGEAACI--DHSGSVHGAYSSGIVAAEDCRRHLSTQLGISDLFQVGK--IIMREEM-----------------TEVMVPFQISR------L--------------------------------------------------------------------------------------------------------------------------------------------------------------------------------------------------------------------------------------------------------------------------------------------------------------

>Pm_Sb3

----------------------------------------------------------------------------------------------------------------------------------------------MDQPP-------------NGFAAGG-FFTHIDGQNRSPPSVIVI-------------------GGGIS---------GIAAARALSNAS--------FK--------VTLLESR-DRVGGRVHTDYSFG---------------------------CPIDM-GASWLHGVC---------------NENSLAPL--I-RM---LGLRLYRTS-GDNSV--------------------------------LYD--HDLESYALFDKHGQQVPQEIV-------------SKVGETFEKILKETV---------------------------------KVRDEHANDMPLIQAMAIVLNRNPH-----------------------------MKLEGLEYEVLQWCI-CR--LEAWF--ATDMDNISL-------KNW--DQ----E-----------------------------HVLTGGHGLMVNGYDPVIKAL--AQG---------------------------------LDIHLNHR----VTKIIQRY---------------------------------------------------NKVIVCV-------------------------------------------------------EDG---A-----SFVADAAIITVPLGVLKANI--------------------------IKFEPELPR---EKLSAIA-DLGVGIENKIALKFN--T-----VFWPNVEVLGR-----------------------------------------------------I--------------------------------------------------------------------------------------------------APTSNACGYFL-----------------------------------------------------------------------NL-HKATGNPVLVCMVAGRFAYEIEK----LSD------EESV----NFVM-SQLRKMLPQA--------------------------------TEPVQYLVSRWGSDPNS-LGSYSC-DLVGK----------------P--AD-LYE---RFCAPVG--------------------------N-LFFAGEAACI--DHSGSVHGAYSSGIAAAEDCRRRLSTQLGISDLFQVAK--VVMREEM-----------------NEVMVPFQISR------L--------------------------------------------------------------------------------------------------------------------------------------------------------------------------------------------------------------------------------------------------------------------------------------------------------------

>Pm_Zm2

----------------------------------------------------------------------------------------------------------------------------------------------MDQPP-------------NGFAAGG-FFTHIDGQNRSPPSVIVI-------------------GGGIS---------GIAAARALSTAS--------FK--------VTLLESR-DRPGGRVHTDYSFG---------------------------CPIDM-GASWLHGVC---------------NENSLAPL--I-RM---LGLRLYRTS-GDNSV--------------------------------LYD--HDLESYALFDKHGQQVPQEIV-------------SKVGETFERILKETV---------------------------------IVRDEHANDMPLFQAIAIVLDRNPH-----------------------------MKLQGLEYEVLQWCI-CR--LEAWF--ATDMDNISL-------KTW--DQ----E-----------------------------HVLTGGHGLMVNGYDPVIRAL--AQG---------------------------------LDIHLNHR----VTKIIQRY---------------------------------------------------NKVIVCV-------------------------------------------------------EDG---A-----SFVADAAIVTVPLGVLKANI--------------------------IKFEPELPK---EKLSAIA-DLGVGIENKIALKFD--T-----VFWPDVEVIGR-----------------------------------------------------V--------------------------------------------------------------------------------------------------APTSNACGYFL-----------------------------------------------------------------------NL-NKATGNPVLMCMVAGRFAYEIEK----LSD------EESV----NFVM-SQLRNMLPQA--------------------------------TDPVQYLVSRWGSDPNS-LGSYSC-DLVGK----------------P--AD-LYE---RFCAPVG--------------------------S-LFFAGEAACI--DHSGSVHGAYSSGIAAAEDCRRRLSAQLGISAGLFQVGKAAMREEMT-----------------AEAMVPFQISR------L--------------------------------------------------------------------------------------------------------------------------------------------------------------------------------------------------------------------------------------------------------------------------------------------------------------

>Pm_Ma5

---------------------------------------------------------------------------------------------------------------------------------------------MASKGF-------------SGRKNFGLLISHIERQQPSPPSVIVI-------------------GGGIS---------GIAAAHALSNAS--------FKALLSITRFVILLESR-NRLGGRVHTDFSFG---------------------------CPVDM-GASWLHGVC---------------NENSLAPL--I-RG---LGLRLYRTS-GDNSV--------------------------------LYD--HDLESYALFDKDGNQVPQQVV-------------MRVGETFERILKETE---------------------------------RVRDENTADMSLLQAISIVLDRNPQ-----------------------------LRQEGLAYEVLQWYI-CR--LEAWF--AADADTISL-------KNW--DQ----E-----------------------------HVLSGGHGLMVQGYYPVIQAL--SKG---------------------------------LDVRLNHR----VAKIAQRS---------------------------------------------------NRVIITM-------------------------------------------------------EDG---N-----TFVADAAIITVPIGVLKANL--------------------------IEFEPRLPA---WKLSAIS-DIGVGIENKIALRFN--T-----VFWPNVEVLGL-----------------------------------------------------V--------------------------------------------------------------------------------------------------AQTSYACGYFL-----------------------------------------------------------------------NL-HKATGHPVLVYMAAGRFAYDIEK----LSD------EEAI----NFVM-LQLKKMIPEA--------------------------------TNPIQHLVSRWGTDPDS-LGSYSC-DLVGK----------------P--AD-LYE---RLCAPVD--------------------------N-LYFAGEAASA--DHSGSVHGAYTSGITAAEVCRSRLSVQHGISDLFHL-----VMTEEF-----------------AEVMVPLQISR------M--------------------------------------------------------------------------------------------------------------------------------------------------------------------------------------------------------------------------------------------------------------------------------------------------------------

>Pm_Ma6

---------------------------------------------------------------------------------------------------------------------------------------------MTSKGS-------------SSRKNFGLL---VSHVERPQPSVIVI-------------------GGGIS---------GIAAARALSNAS--------FN--------VILLESR-NRLGGRVHTDHSFG---------------------------CPVDM-GASWLHGVC---------------NENSLAPL--I-RG---LGLRLYRTS-GDNSV--------------------------------LYD--HDLESYALFDKDGNQVPQQVV-------------TKVGEAFERILKETE---------------------------------KVRNEHVVDMSLSQAISFVLERYPQ-----------------------------LRQEGMAHEVLQWFI-CR--LEAWF--AADADTISL-------KNW--DQ----E-----------------------------HVLSGGHGLMLQGYYPVIQAL--SED---------------------------------LDIRLNHR----VKKIAQCN---------------------------------------------------NRVIVTV-------------------------------------------------------EDG---N-----TFVADAAIITVPLGVLKANL--------------------------IEFEPKLPE---WKLSAIS-DIGVGIENKIALRFS--N-----VFWPNVEFLGL-----------------------------------------------------V--------------------------------------------------------------------------------------------------AQKSYACGYFL-----------------------------------------------------------------------NL-HKATGHPILVYMAAGRFAYDMEK----LSD------EEAV----NFVM-LQLKKMIPDA--------------------------------TDPIQHLVSHWGTDGDS-LGSYSC-DLVGK----------------P--AD-IYE---RFCAPVD--------------------------N-LYFAGEAASA--DHSGSVHGAYTSGVMAAEVCQRHLSVQHGISDLFQL-----VMREEL-----------------SEAMVPLQISR------M--------------------------------------------------------------------------------------------------------------------------------------------------------------------------------------------------------------------------------------------------------------------------------------------------------------

>Pd_At4

----------------------------------------------------------------------------------------------------------------------------------------------MDKKK---------NSFPDNLPE-GTISELMQKQNNVQPSVIVI-------------------GSGIS---------GLAAARNLSEAS--------FK--------VTVLESR-DRIGGRIHTDYSFG---------------------------CPVDM-GASWLHGVS---------------DENPLAPI--I-RR---LGLTLYRTS-GDDSI--------------------------------LYD--HDLESYGLFDMHGNKIPPQLV-------------TKVGDAFKRILEETE---------------------------------KIRDETANDMSVLQGISIVLDRNPE-----------------------------LRQEGMAYEVLQWYL-CR--MEAWF--AVDANLISL-------KCW--DQ----D-----------------------------ECLSGGHGLMVQGYEPVIRTI--AKD---------------------------------LDIRLNHR----VTKVVRTSN--------------------------------------------------NKVIVAV-------------------------------------------------------EGG---T-----NFVADAVIITVPIGVLKANL--------------------------IQFEPELPQ---WKTSAIS-GLGVGNENKIALRFD--R-----AFWPNVEFLGM-----------------------------------------------------V--------------------------------------------------------------------------------------------------APTSYACGYFL-----------------------------------------------------------------------NL-HKATGHPVLVYMAAGNLAQDLEK----LSD------EATA----NFVM-LQLKKMFPDA--------------------------------PDPAQYLVTRWGTDPNT-LGCYAY-DVVGM----------------P--ED-LYP---RLGEPVD--------------------------N-IFFGGEAVNV--EHQGSAHGAFLAGVSASQNCQRYIFERLGAWEKLKLVSLM-GNSDIL-----------------ETATVPLQISR------M--------------------------------------------------------------------------------------------------------------------------------------------------------------------------------------------------------------------------------------------------------------------------------------------------------------

>Pd_Br6

----------------------------------------------------------------------------------------------------------------------------------------------MDKK----------VSFTDELPDVTISALLQKQNNVVQPCVIVI-------------------GSGIS---------GLAAARSLSEAS--------FN--------VTVLESR-DRIGGRIHTDYSFG---------------------------CPVDM-GASWLHGVS---------------NDNPLAPI--I-RR---LGLTLYRTS-GDDSI--------------------------------LYD--HDLESYGLYDMHGNKIPPQLV-------------TQVGDAFKRILEETE---------------------------------KIRDETANDMSVLQGISIVLDRHPE-----------------------------LRQEGIAYEVLQWYI-CR--MEAWF--AVDANLISL-------KCW--DQ----D-----------------------------ECLSGGHGLMVQGYEPVIRTI--AKD---------------------------------IDIRLNHR----VTKVSRTSN--------------------------------------------------NKVIVEV-------------------------------------------------------EGG---T-----NFVADAVIITVPIGVLKANL--------------------------IQFEPELPQ---WKTSAIS-DLGVGNENKIALRFE--N-----VFWPNVEFLGM-----------------------------------------------------V--------------------------------------------------------------------------------------------------APTSYSCGYFL-----------------------------------------------------------------------NL-HKATGHPVLVYMAAGNLAKDLEK----LSD------EATA----NFVM-LQLKKMFPDA--------------------------------PDPAQYLVTRWGTDPNT-LGCYAY-DVVGM----------------P--ED-LYA---RLGEPVD--------------------------N-IFFGGEAVNV--EHQGSAHGAFLAGVSASQNCQRYIFERLGAWEKLKLVSLM-RNSDIL-----------------ETGTVPLQISR------M--------------------------------------------------------------------------------------------------------------------------------------------------------------------------------------------------------------------------------------------------------------------------------------------------------------

>Pd_Pr3

-----------------------------------------------------------------------------------------------------------------------------------------------------------------------MH-----ALLLLSLVVVYQ-------------------GLQLH---------VF----SMMHLL--------RS--------VILLESR-DRLGGRIHTDYSFG---------------------------CPVDM-GASWLHGVC---------------NENPLAPL--I-RR---LGLTLYRTS-GDDSV--------------------------------LYD--HDLESYALFDMDGSQVPQEMV-------------IEVGNTFKQILKETE---------------------------------KVRNENTDDMSVSQAISIVMDRHPE-----------------------------LRQNGLAHEVLQWYI-CR--MEAWF--AADADVISL-------KNW--DQ----E-----------------------------HVLSGGHGLMVQGYDPIIRAL--AED---------------------------------IDVRLNHR----VTKILNGH---------------------------------------------------NKMMVTI-------------------------------------------------------EDG---R-----NFVADAAIITVPHGILKAKM--------------------------IEFEPKLPE---WKVDAIS-DLGVGNENKIALRFE--K-----VFWPNVELLGV-----------------------------------------------------V--------------------------------------------------------------------------------------------------APTSYACGYFL-----------------------------------------------------------------------NL-HKTTGHPVLVYMAAGRFAYDLEK----LTD------DGAV----SFVM-LQLKKMLPDA--------------------------------TDPVQYLVSRWGTDLNS-LGCYSL-DLVGK----------------P--GD-IYD---RLRAPLG--------------------------S-LFFGGEAVSM--DHQGSVHGAYSAGVIAAEDCQRHLLNKFGRLEKLQHAYIT-D---EV-----------------LEATVPLQISR------M--------------------------------------------------------------------------------------------------------------------------------------------------------------------------------------------------------------------------------------------------------------------------------------------------------------

>Pd_Vv6

----------------------------------------------------------------------------------------------------------------------------------------------MDSKQ-------------SLQGAFVSFIERRRRSLSSIPSVIVI-------------------GGGIS---------GIAAAHTLHNAS--------FQ--------VVLLESR-DRLGGRIHTDYSLG---------------------------CPVDM-GASWLHGAC---------------NENPLAPL--I-CR---LGLTLYRTS-GDDSV--------------------------------LYD--HDLESCTLFDMDGHQVPQKMV-------------VEVGETFKKILKETE---------------------------------NVRIEHCDDMSVLQAISIVLDRHPE-----------------------------LRQEGLANEVLQWYI-CR--MEAWF--AVDADMISL-------KSW--DQ----E-----------------------------HILSGGHGLMVQGYDPIIKTL--SKD---------------------------------LDIRLNHR----VTNISYGC---------------------------------------------------KKVVVTV-------------------------------------------------------EGG---R-----NFVADAAIITVPIGILKANL--------------------------IEFKPKLPD---WKVNAIS-DIGVGNENKIALRFD--D-----VFWPNVELLGI-----------------------------------------------------V--------------------------------------------------------------------------------------------------APTSYACGYFL-----------------------------------------------------------------------NL-HKATGYPILVYMTAGSSACGLEK----LSD------ECAV----NFVM-LQLKKMFPDA--------------------------------TKPVQYLVSRWGTDPNS-LGCYAH-DVVGK----------------P--ED-SYE---RLLEPLD--------------------------N-LFFGGEAVSL--DHQGSVHGAYSAGIMAAENCQRYILERRGNLEKLQLVSLR-S---AI-----------------HEAAVPLQISR------M--------------------------------------------------------------------------------------------------------------------------------------------------------------------------------------------------------------------------------------------------------------------------------------------------------------

>Pd_Gm7

----------------------------------------------------------------------------------------------------------------------------------------------MDPN----------HLFFTHFRDGAIASRIEGQHRGALPSVIVI-------------------GAGIS---------GLAAARSLYDAS--------FK--------VTVLESR-DRLGGRIHTDFSFG---------------------------CPVDM-GASWLHGVC---------------NENPLAPL--I-RG---LGLSLYRTS-GDNSV--------------------------------LYD--HDLESYMLFNIDGKQVPQQMV-------------IEVGDTFKKILEETG---------------------------------KVRDEHTEDISVSQAISIVLDKHPD-----------------------------LRQQGLAHEVLQWFI-CR--MEAWF--AADADMISL-------KTW--DQ----E-----------------------------HVLSGGHGLMVQGYDPVIKVL--AKD---------------------------------IDIRLNHR----VKKISSGY---------------------------------------------------NKVMVTV-------------------------------------------------------EDG---R-----NFVADAAIITVPIGILKANL--------------------------IEFEPKLPD---WKVSAIS-DLGVGNENKIALRFD--K-----VFWPNVELLGT-----------------------------------------------------V--------------------------------------------------------------------------------------------------APTSYTCGYFL-----------------------------------------------------------------------NL-HKATGHPVLVYMVAGRFAYDIEK----LSD------EAAA----NFVM-QQLKKMFPNA--------------------------------SKPVQYLVSRWGTDPNS-LGCYSY-DLVGK----------------P--TD-VYD---KLRAPLG--------------------------N-LFFGGEAVSL--DNQGSVHGAYSAGVMAAENCESYMLEKLGHAEKLSLASVR-H---EM-----------------LETLIPLQISR------M--------------------------------------------------------------------------------------------------------------------------------------------------------------------------------------------------------------------------------------------------------------------------------------------------------------

>Pd_Gm8

----------------------------------------------------------------------------------------------------------------------------------------------MEPN----------QLFSTHFRDGTIASCIEGQHRGAIPSVIVI-------------------GAGIS---------GLAAARSLHDAS--------FK--------VTVLESR-DRLGGRIHTDFSFG---------------------------CPVDM-GASWLHGVC---------------NENPLAPL--I-RG---LGLSLYRTS-GDNSV--------------------------------LYD--HDLESYMLFNIDGKQVPQQMV-------------IEVGDIFKKILEETG---------------------------------KVRDEHTEDISVSQAISIVLDRHPE-----------------------------LRQQGLAHEVLQWFI-CR--MEAWF--AADADMISL-------KTW--DQ----E-----------------------------HVLSGGHGLMVQGYDPIIKVL--AKD---------------------------------IDICLNQR----VKMISSGY---------------------------------------------------NKVMVTV-------------------------------------------------------EDG---R-----NFVADAAIITVPIGILKANL--------------------------IQFEPKLPD---WKVSAIS-DLGVGNENKIALRFD--K-----VFWPNVELLGT-----------------------------------------------------V--------------------------------------------------------------------------------------------------APTSYTCGYFL-----------------------------------------------------------------------NL-HKATGHPVLVYMVAGRFAYDIEK----LSD------EAAA----NFVM-QQLKKMFPNS--------------------------------SKPVQYLVSRWGTDPNS-LGCYSY-DLVGK----------------P--LD-VYD---KLRAPLG--------------------------N-LFFGGEAVSL--DNQGSVHGAYSAGVMAAENCESYLLEKLGHVEKLSLASVR-H---EM-----------------LETLIPLQISR------M--------------------------------------------------------------------------------------------------------------------------------------------------------------------------------------------------------------------------------------------------------------------------------------------------------------

>Pd_Ca4

----------------------------------------------------------------------------------------------------------------------------------------------MDPK----------LFFSNNYLDGTITSRIE-SQQRPLPSVIVI-------------------GAGIS---------GVAAARILHDAS--------FK--------VTLLESR-DRLGGRIHTDYSFG---------------------------CPVDM-GASWLHGVC---------------NENPLAPL--I-CG---LGLTLYRTS-GDNSV--------------------------------LYD--HDLESCMLFSIDGKQVPQQTV-------------IEVGETFKSILEETG---------------------------------KVRDEHPEDISVSEAISIVLDRHPQ-----------------------------LRQQGLAHEVLQWYI-CR--MEAWF--AADADMISL-------QTW--DQ----E-----------------------------HVLSGGHGLMVQGYNPVINAL--AKD---------------------------------VDIRLNHR----VTKISSGY---------------------------------------------------NKVMVTV-------------------------------------------------------EDG---R-----NFVADAAIITVPIGILKANL--------------------------IEFEPRLPD---WKVSAIS-DLGVGNENKIALRFD--K-----VFWPDVELMGV-----------------------------------------------------V--------------------------------------------------------------------------------------------------APTSYSCGYFL-----------------------------------------------------------------------NL-HKATGHPVLVYMAAGRFAYDLEK----LSD------ESAA----NFVM-LQLKKMFPDA--------------------------------SEPVQYLVSHWGTDPNS-LGCYSY-DLVGK----------------S--MD-VYD---KLRAPLG--------------------------N-LFFGGEAMSL--DNQGSVHGAYSAGVMAAENCQRYLWEKQGNLESLAQVSVR-N---ET-----------------LGTTIPLQISR------I--------------------------------------------------------------------------------------------------------------------------------------------------------------------------------------------------------------------------------------------------------------------------------------------------------------

>Pd_Mt3

----------------------------------------------------------------------------------------------------------------------------------------------MDPK----------LFFSNNFLDGTITSLID-SQQRPAPSVIVV-------------------GAGIS---------GIAAARILHDAS--------FK--------VTLLESR-DRLGGRIHTDYSFG---------------------------CPVDM-GASWLHGVC---------------NENPLAPL--I-RC---LGLTLYRTS-GDDSV--------------------------------LYD--HDLESCMLFDIDGHQVPQQTV-------------IEVGETFKRILEETG---------------------------------KVRDEHPEDISVSEAISIVLDRHPQ-----------------------------LRQQGLSHEVLQWYI-CR--MEAWF--AADADMISL-------KTW--DQ----E-----------------------------HVLSGGHGLMVQGYKPVINAL--AKD---------------------------------IDIRLNHR----VTKISSGY---------------------------------------------------NKVMVTL-------------------------------------------------------EDG---R-----NFVADAAIITVPIGILKANL--------------------------IEFEPRLPD---WKVSAIS-DLGVGNENKIALKFD--K-----VFWPDVELMGV-----------------------------------------------------V--------------------------------------------------------------------------------------------------APTSYACGYFL-----------------------------------------------------------------------NL-HKATGNPVLVYMAAGRFAYDLEK----LSD------ESAA----NFVM-LQLKKMFPDA--------------------------------CEPVQYLVSHWGTDPNS-LGCYSY-DLVGK----------------S--MD-VYD---KLRAPLG--------------------------N-IFFGGEAMSL--DNQGSVHGAYSAGVMAAENCQRYLWEKQGNLESLSQVSAR-H---ET-----------------LGTNFPLQISR------I--------------------------------------------------------------------------------------------------------------------------------------------------------------------------------------------------------------------------------------------------------------------------------------------------------------

>Pd_Cc4

----------------------------------------------------------------------------------------------------------------------------------------------MDSN----------QSFSNNLLDDTVASLIERAQIGSLPSVIVI-------------------GGGIS---------GLAAARILYDAS--------FK--------VVLLESR-DRLGGRIHTDYSFG---------------------------CPVDM-GASWLHGVC---------------NENPLAPL--I-RR---LGLTLYRTS-GDNSV--------------------------------LYD--HDLESYALYDMDGNKVEKEMA-------------IKVGEIFKRILNESQ---------------------------------KVRDEHTNDMSVLQAISIVLDRHPE-----------------------------LRQEGLAYEVLQWYI-CR--MEAWF--AVDADMISL-------KCW--DQ----E-----------------------------QVLSGGHGLMVQGYDPVIKAL--SKD---------------------------------IDIHLNQR----VTKISNGC---------------------------------------------------NKVMVTV-------------------------------------------------------EDG---R-----NFVADAAIVTVPLGILKANL--------------------------IQFEPKLPE---WKLSAIA-DIGVGNENKIALRFD--N-----VFWPNVELLGV-----------------------------------------------------V--------------------------------------------------------------------------------------------------APTSYACGYFL-----------------------------------------------------------------------NL-HKATGHPVLVYMAAGRFAYDLEK----LSD------ESAA----NFVM-MQLKKMFPDA--------------------------------TEPVQYLVSRWGTDPNT-LGCYSY-DVVGM----------------P--GD-LYE---RLRAPLG--------------------------N-LFFGGEAVSM--EHQGSVHGAYSAGVMAAQNCQKYLLKQPGNLEKLQLVTLS-H---EI-----------------LGTAFPLQISR------M--------------------------------------------------------------------------------------------------------------------------------------------------------------------------------------------------------------------------------------------------------------------------------------------------------------

>Pd_Pt5

---------------------------------------------------------------------------------------------------------------------------------------------------------------MENLIENGTFISQVERPNSSLPTVIVI-------------------GGGIS---------GLAAARRLHDAS--------FK--------VILLESR-DRLGGRIHTDHSFG---------------------------YPVDL-GASWLHGVC---------------NENPLAPL--I-RG---LGLKLYRTS-GDNSV--------------------------------LYD--HDLESYTLFDKEGHKIPQQMV-------------IEVGDAFKRILDETE---------------------------------KVRDEHTDDMSVLQAIWIVLDRHPE-----------------------------LRQEGLAYEVLQWYI-CR--MEAWF--AADADMISL-------KSW--DQ----E-----------------------------QVLSGGHGLMVQGYDPIIKAL--AKD---------------------------------IDIRLNHR----VAKISNGP---------------------------------------------------NKVMVTV-------------------------------------------------------EDG---T-----GFIADAAIITVPLGILKANL--------------------------IHFEPKLPQ---WKVDAIS-DLGFGSENKIAMQFD--R-----VFWPDVELLGV-----------------------------------------------------V--------------------------------------------------------------------------------------------------APTSYACGYFL-----------------------------------------------------------------------NL-HKATGHPVLVYMAAGRFACDLEK----LSD------ESAA----NFVM-LQLKKMFPNA--------------------------------TEPVQYLVTRWGTDPNS-LGCYSY-DLVGK----------------P--GD-SYE---RLRAPLG--------------------------N-LFFGGEAVSME-DHQGSVHGAYSAGIMAAENCQGHILERLGYFDKLQLVPSR-G---EI-----------------HDAAFPLQISR------M--------------------------------------------------------------------------------------------------------------------------------------------------------------------------------------------------------------------------------------------------------------------------------------------------------------

>Pd_Rc3

---------------------------------------------------------------------------------------------------------------------------------------------MDGTDV---------SSSLRDLLLDGTFASHIERQNSSPPSVIVI-------------------GAGIS---------GLAAARVLYDAS--------FK--------VILLESR-DRLGGRIHTDYSFG---------------------------YPVDL-GASWLHGVC---------------NENPLAPL--I-RS---LRLTLYKTS-GDNSV--------------------------------LYD--HDLESCTLFDMNGHQVPKELV-------------IEVGDIFKRILKETE---------------------------------RIRDEHPDDMSILQAIKLVLDRHSE-----------------------------LRQEGIANEVFQWYI-CR--MEAWF--AVDADMISL-------KMW--DQ----A--------------------------SEENVLCGGHGLMVQGYDPIIKAL--AKD---------------------------------IDIRLNHK----VTKICNAL---------------------------------------------------NKAMVVV-------------------------------------------------------EDG---R-----NFIADAVIVTVPLGILKANL--------------------------IQFEPKLPD---WKVAAIS-DLGVGSENKIALQFD--E-----VFWPNVELLGI-----------------------------------------------------V--------------------------------------------------------------------------------------------------APTSYACGYFL-----------------------------------------------------------------------NL-HKATGHPVLVYMAAGRFAYDLEK----LSD------ESAA----TFVM-LQLKKMFPHA--------------------------------TDPVRYLVTRWGTDPNS-LGCYTY-DVVGK----------------P--DD-LYD---RLRAPLG--------------------------N-LFFGGEAVSM--DHQGSVHGAYASGLMAAENCQRHVLEKLGTMEKLQLVPFR-T---AI-----------------HEAAIPLQISR------M--------------------------------------------------------------------------------------------------------------------------------------------------------------------------------------------------------------------------------------------------------------------------------------------------------------

>Bat_Sr

------------------------------------------MDRPISRRDFFDGVATVAGVAALGSLAGCAGPGAGRPNTIGTKNLK-------------------------VPSETNY----------PPVLT------GMQGNT-S----NALSVPHALRDGRFWDHAGAPEPTGEHYDLVVV-------------------GAGIS---------GVAAAHRWLERA------PDAR--------ILILDNH-DEVGGHARRNEFHPAG----------------------RKGPLIGYGGSQSIDAPS---------------AWTPEGKD--LLDK---IGVRVRRFEE-YFDQTLY-TGMRMYGSVFCDRETFP--------------R-----EKLVVFTPGR------------------KAPEWIAEL-PVAEQAREDLLML------YGHPPDWFPGLSDG--------LKKERLAGLTYAEFLRDVCKVHPDV--------------------------------------LRY-V-QTM-PNDEW--GYGSDAFGA------IDAW--GSAGEHDYPGFQGL-ELDRA--TPSEYNSPSMIKGWGADDPYVYHFPEGNQAIVRMM--IGRMIPGF---ATSTEMEEISTATFDYGRLDLPRNQIRIRLSCP----VVSAANDGDPATA----------------------------------------------TTATVGYFD-----------------------------------------------------------G-DRVRTVRAANVIMACWNMVIPY------------------------------LVGELPA---DQKDALS------KAVKVPLLYA--M--VQLRGWEAWKRAG----------------------------------------ISRTRFTG-AYWCGAELDYPVSIG------------------------------------------------------------------------------------EYLRPKTPDQPINVHMISTPAL----------------------------------------------------------------PGMSPA-SGSVAGRHELIKTPYEYLE----------------------YSIR-DQLTRLLGPSG---------------------------FDPARDIQAITVNRWGH-------GYAP-EYATPW---N-------LDFYPHGPF-PAT---VARRRA---------------------------GRIAIANSDS----VPAAYADAAITAAYRAVGELQA--------------------------------------------------------------------------------------------------------------------------------------------------------------------------------------------------------------------------------------------------------------------------------------------------------------------------------------------------------------------------

>BdMs

-----------------MLY--------------SKEDKDLGMDRKITRRDFLNGVAITVGAAAISSRA--------------------------------------------FAQGAPD----------PARLT------GLRGQY-E----GVQSVMHAVRDGTFRLEPGSVRPTKERYDLVVV-------------------GAGIS---------GLAAAFHYRQLS------PGAK--------VLLLDPL-DDFGGHAKRNEFRVG------------------------GRTLIGYGGSQSMQTPS---------------YFSPAVNK--LLAD---IGIEPKKFE-GYYDGEWFDKRGLGE-GMFFAREVFG--------------S-----N--QLVRVGE------------------KAADWVPKT-PLNPKAKQDLIRLL----DN--PPDYLEGLSRA--------EKFERLSRITYKEFLLETVKADPQL--------------------------------------VTI-F-QSS-TEPYF--GVGIDATTC------LDAW--AN----HNPGFDGM-DLGE---KIYKTMSPSGRLARTDPDDYIYHFPDGNAGIARAL--VRRLIPRA---LKGRSMEDLVTQRVDYAQLDVPNRPVRLRLGAT----VVRVRHLGDPATA----------------------------------------------QEVEVTYVG-----------------------------------------------------------K-GGLQSVRAGHVILACWHKVIPY------------------------------ITDELPA---EQVEALK------DQVKVPLLYT--N--VAIRNWRAFDRLK----------------------------------------IGNFSAPGTFFANDVGIDFPVSMG------------------------------------------------------------------------------------AYRFAQRPDEPVLLHLGKVMTS----------------------------------------------------------------PGK-PSREQALEGRRRLQTLSFRDLE----------------------RTIR-DLLGRALSGGG---------------------------FDPARDIAAITVNRWAH-------GYAY-EYMRPW---D--------AFWPDGPL-PIE---TARRKW---------------------------GRVAIANSDS----GAYAYAHSAIDQGVRAVRELVYVRPDAPAYAKFPGPPPEKL-----------------EFIE----------------------------------------------------------------------------------------------------------------------------------------------------------------------------------------------------------------------------------------------------------------------------------------------------------------------------------

>Bp_Rg

--------------------------------------------MTITRRDFVHGAALAALPGAAAA-------------------------------------------------QDDD----------PSLAT------GLQGQT-E----ADNAVAHVWRDRPGRWDDQAAELDADEPDLLVV-------------------GAGIS---------GLAGAWWFREHAG-----RPVT--------MRLLDAA-AEVGGHARRNTFISR-----------------------SGHTLIGYAGSQSLDSPS---------------LFSPASKG--LLRG---VGVDLARFEQGAFDHDWQRRHGLDGHALHFGAAGWG--------------R-----ACTVFRGDGK------------------APADWLART-PLGPQARQVLARLL----DEGSRHDPLPGVRTR-------AARAAVLARWTYAEFLRRGWGADDEA--------------------------------------QRW-H-RGA-TRGYF--GVGSDATSA------LDAW--AL----GLPGFQAL-DLGE---RPFAANSPSGRQLRLGQDDYVYHFPDGNAGVVRAL--LRSLLPQA--LPGAEGLDGLVDAPLRHARLDEPGQPLRISLRST----VVGLRHLGPPERA----------------------------------------------ERVEVRYVD-----------------------------------------------------------AAGRLRAVRARQVLLACWHRVVAR------------------------------LCDELPA---AQRRALD------DQVKVPLVYA--N--VLLSNWRAWKAAG----------------------------------------VSSLHPVD-GFWDDVALDFPVRLG------------------------------------------------------------------------------------RQRPPESADEPILLHLGKVVVP----------------------------------------------------------------GGGAPAREQSAAGRKALLDWDFGRFE----------------------HEIV-TLLGEALGPHG---------------------------FDAARDLEAITANRWPH-------GYAY-EYMRPW---D--------PFWPRAPL-PCE---TARRGW---------------------------GRVAIANSDA----GAYAYANGAIDQAARAVQELLPRARLPAWQR-FPGPAMG---------------------------------------------------------------------------------------------------------------------------------------------------------------------------------------------------------------------------------------------------------------------------------------------------------------------------------------------------------

>Bp_HTCC

-------------------------------------------MKKMTRRDFINGSAVALGATTLPSPLAWALAAEG----------------------------------------EAY----------PPALT------GLRGNH-P----GSNTMAHARAWNAD-FDLDQIVDTQESYDLVVV-------------------GAGLS---------GLAAAFFYRQKFG-----PDKT--------ILILDNH-DDFGGHAKRNEHTID------------------------DHQLITYGGSQTLVEPR---------------EAPEAVKS--LFKG---VGIDLNRFD-TAFNQEFYSDHGLGA-TTYFNAKQFG--------------R-----DTVV-RHPFGNYYNYIEGLHGAAI----SDEEAVAQT-PLSKRGQRQLLHIL----KS--GLHSLDV-SED--------DLPEYIKTHNYFDYLTQTLGVDDPQ--------------------------------------VLHMA-RHS-AIDWS--DASAELLTI------KKAK--EA----GALGFAPV-------------------KVYDADHPYIHHFPDGNAGVARAI--VKYLIPSI--AE-GATAESLVGAKFDYGQLDGSNHPSRIRLNST----VVDVHHSPDKAGT----------------------------------------------ERVSVHYIR------------------------------------------------------------GEKAHKVSAGHVVMACHNAMVPH------------------------------IVSDLPE---KQATALS------EQLKSPLVYT--S--VGLRNWRAFKEQG----------------------------------------IGLAMSPGNMH-QTVFIDFPVSLG------------------------------------------------------------------------------------GYQHTEGPDAPCVLQMISCPYS--------------------------------------------------------------EQSGV-PAKEQYREARYRMLGTPFSVYE----------------------AEVR-EHLSGMLSSKY---------------------------FNFDRDVASLTVNRWAH-------GYTV-AGREDP------------AAGP--GG-SVT---IGRQPH---------------------------RRITIANSDS----APEADAIAAMEMGYRAITEL----------------------------------------------------------------------------------------------------------------------------------------------------------------------------------------------------------------------------------------------------------------------------------------------------------------------------------------------------------------------------

>Bp_Nn

----MTTSQRTEKGRKDMKS--------------SDRDLGMGMGMPIARRDFLNGALMSGAALSGLVPGTSARAADP----------------------------------------MPY----------PPALT------GMRGSGYP----TAYSTGHALRDHVF-MPTGAPQETADSYDLIVV-------------------GGGIS---------GLAAAWFYQQRHG-----GSKR--------VLILDNH-DDFGGHAKRNEFGEG------------------------AS--MRLSNAGSFNIFA------------EDGETGGAHAD--LYRA---LGIDIGKLAAETVDPGFYHRHGMGQ-GVFFDRETFG--------------R-----DVLLPDPAPWTDFTYLYA-PTTPADAEARWARFMADA-PLSPRARADVHRLY----HA--ATDYMAGMTVE--------EKVRRLDTMSYADYLTGPAGCDPMV--------------------------------------VTY-L-RDR-TFGSGRGLHSTTALS--------AHQ--RF----GLPGFAGL-GLPA----------A----YETDEAGTSYHFPEGNATVARLL--VGRLIPGA--LK-GRTAEGVMLERVDYSRLDDAANATRLRLNST----AVHVANVP---------------------------------------------------GGVEVHYTR------------------------------------------------------GG---VAGQFLKTRARQCVLACWNYVIPY------------------------------ICPELPA---EQREALS------YNVKTPNLWV--N--VWLNNWRAFHKAG----------------------------------------TCYMNAPGSYY-ASLILEQPVSIG------------------------------------------------------------------------------------GYRHSQTPDDPTVLTMLHGFET----------------------------------------------------------------PGL-PIKDQFRIGRAELYATSFETFE----------------------RNTR-EQLGRALGPYG---------------------------FDPARDIAGLTVNRWGH-------GYSY-WYSALY---DDFL---KTGA----PP-PHL---LARRPC---------------------------GAIAIANTDS----GGTDTTELAIDMAARAIAELG---------------------------------------------------------------------------------------------------------------------------------------------------------------------------------------------------------------------------------------------------------------------------------------------------------------------------------------------------------------------------

>Bp_Ls

--------------------------------------MGKKSLSGITRRDILHGLGALGATA-LVPGKALADAVLA---------ME-------------------------TSGASGY----------PPLRT------GLRGNH-V----GSFEVAHSLARHGQTDWSPVQSLDTIEYDLVVV-------------------GAGIS---------GLAAAYFFQQK-N-----PNAR--------VLLLDNH-DDFGGHAKRNEFDVG------------------------KQKIIGYGGSQTMENPS---------------SYPRVTQA--LLEE---LGVNLNAFD-TAYDQNFYKRHGLTG-GTFFNQEAWG--------------E-----NRLIPVDVANLSYYLPLADS------PISIEAAVGRM-PMSDAAKAQMRHLL----TT--DTDQLADIPAD--------KKIDYLYSMTYLDFVAKHLGVTEPE--------------------------------------VIA-A-LQG-LITES--GVGIESTSAG-----DAIM--YS----GLPGANAC-GLPE----------Y-------DGDPYIHHFPDGNASIARAL--VQRLIPEV--SS-APTLEALLTQPFDYSALDKADAKVQLRLSST----VVNVAHRGKSEKG----------------------------------------------NGVSVTYVK------------------------------------------------------------YGQAFNVKARATILACYNTIIPA------------------------------LCPELPE---KQREALS------LQVKSPILYT--S--VALTNWRAWAELG----------------------------------------IGGARCPGSYH-NTAILDFPVSLG------------------------------------------------------------------------------------SYQFSDNPDQPIMVHMERFPHP--------------------------------------------------------------INRDL-DKRGKLRWGRHQLLSTPYETIE----------------------RNVV-QQLSGMLGDGG---------------------------FDPEKDISAITVNRWAH-------GYSY-DYDFLE---EPYY---DDWNDP--RY-PHV---RGRQTF---------------------------GSIAIANSDA----RGSAYLDAAIGQAHRAVEALS---------------------------------------------------------------------------------------------------------------------------------------------------------------------------------------------------------------------------------------------------------------------------------------------------------------------------------------------------------------------------

>Bp_Cl

----------------------------------------MPSRLNITRRDFLGGIALGTTAGALTPLELLAQSA-A----------------------------------------APY----------PPALT------GMRGSQ-P----GSFDVAHAMAWGGKEFSV-PREQTDDDYDLVVV-------------------GGGIS---------GLAAAFYWQQQKG-----TDSR--------ILILDNH-DDFGGHARRNEFTVD------------------------GKKLVGYGGSQSIDTPG---------------SYSPAAAQ--LLRD---VGIITDRFY-DYYDQDFFAGAKLGR-GSYFPEEVYG--------------K-----DLTLPGVKSWRSSDDAA-----------LTENDIRDY-PIPLEARTALLSLM----NS--TINYAAHIPRE--------QRAEWLRGQTYREFLLDVVGVPEDV--------------------------------------YYL-F-RDS-ARGWW--GVGWDALST------LAAA--SM----GMPGTAYL-DLPE----------GSSDE-PVRDEPYIFHFPDGNAGVARAI--LRKLIPDA--VP-GSTMEDLVLAKTDYGKLDRKESPCRLRLNST----AVNVHHTP---DG----------------------------------------------SHVDVSYVR------------------------------------------------------------DGKSHRVRARHAVLACYNAIIPH------------------------------IAPELPD---DQRDAIN------YATKVPLIYI--S--VAVRNWRPFAELG----------------------------------------MHSISVMQSDFMHSFGMDFPVSMG------------------------------------------------------------------------------------GVKYPENPDEAAILHGSVVPCA--------------------------------------------------------------PDQGL-TAKEQHVIGRQAMYQMSYNDME----------------------SRIL-RQLSGGLRGTS---------------------------FDPERDIAAITVNRWPH-------GYAY-EYNDYS----------DPPEYGPGYG-PHL---LGARQM---------------------------GRISVANSDA----SAYAFVDGAIDAAYRATREQLS--------------------------------------------------------------------------------------------------------------------------------------------------------------------------------------------------------------------------------------------------------------------------------------------------------------------------------------------------------------------------

>Bp_NOR5

----------------------------------------MPSRLKISRRDFLGGIALGTTAGALTPLELLAQSGSS----------------------------------------TSY----------PPALT------GLRGSQ-P----GSFDAAHAMAWGGKKFAV-PKEQTDDDYDLVVV-------------------GGGIS---------GLAAAFYWQQEKG-----PNSR--------ILILDNH-DDFGGHARRNEFTVD------------------------GKTLVGYGGSQSIDTPG---------------SYSPAASK--LLRD---LGIFTDRFY-EYFDQDFFARHNLGR-GSYFPKEAYG--------------K-----DLTLPGLRSWRSNDASP-----------VSDDMVKDY-PMAPEARAALLELM----NS--EKNYAAHIPRE--------DRAEWLRKQSYRDFLLKTVGVPEDV--------------------------------------YFL-F-RDS-ARGWW--GTGWDAISS------LSAA--GM----GMPGTLHL-DLPE----------YRSSE-PVRDEPYIFHFPDGNAGVARAL--LRKLIPVA--VA-GSTMEDLVLANTDYAKLDLRSSPCRLRLNST----AVDVRHTA---DE----------------------------------------------SMVNVTYQR------------------------------------------------------------DGKSYRVRAKHAVLACYNAIIPH------------------------------ICPELPE---NQREAID------YATKVPLIYV--S--VAVRNWRPFAQLR----------------------------------------MHSINIMQTDFMHSFGMDFPVSMG------------------------------------------------------------------------------------GVNYPQSPDQPTILHGSVTPCA--------------------------------------------------------------PDQGL-SAREQHVIGRRAIYEMSWDDME----------------------GHIL-RQMTGALSATE---------------------------FDPQRDIAAITANRWPH-------GYAY-EYNDYS----------DPPEYGPDNG-PHV---LGARQL---------------------------GRISIANSDA----SAYAFVDGAIDAAYRATREQLA--------------------------------------------------------------------------------------------------------------------------------------------------------------------------------------------------------------------------------------------------------------------------------------------------------------------------------------------------------------------------

>Bp_Cn

--------------------------------------------MTITRRDFLNGTALTIAAG-LAPVQLLHAQQGG--------------------------------------AGVAY----------PPALT------GLRGNH-A----GTYTLAHSLAREGAKYP---VVLAREAFDLVVV-------------------GGGLS---------GLAAAWFYRRRFG-----ANRR--------ILILDNH-DDFGGHAKRNEFTVR------------------------GKRLVTYGGSAEMPAGA---------------ASDAAVRE--LLAG---IGLDATRPP-GTVPADPYGALGMGR-AVFFDAEHFG--------------R-----DQWVAGDPFGELIDVRSG-QQAGAPDAAALSRFLDNT-PLPVADRAALARLA----AG--TTDYLPALSGT--------ERAAALRSMRYASFLRDKAGIGLAG--------------------------------------QRF-L-RSR-SNDAY--ALDVDGISV------ADAI--AI----GLPAGANM---PA----------PAGNVRLGKSPASRLWFPDGNASLARLL--VQSLIPGV--AS-IARPADVVSAAFDYSRLDRDGQPVRLRLNST----AIAVEPDG---------------------------------------------------NITRVTYGF------------------------------------------------------------SGNLHRVEARHVVLAGYNMMIPY------------------------------LLPSLPA---KQKEVLH------NEAKAPLVYT--K--VALDHWQAFAALR----------------------------------------TRRIHAPAMAY-TDLWLEPPAG-------------------------------------------------------------------------------------------RQPSDPAVLHMLYVPTV--------------------------------------------------------------PDSGM-QARDRFRAGRALLLGTPFDTLE----------------------RDIR-AQLDRMLGHKG---------------------------FASKDVIRGITVNRWAH-------GYSY-MPDSLA----------AENVAP-DA---VW---PGLAGV---------------------------GNISIANSDN----AGSPSLTAAVGQGKRAVERLPG--------------------------------------------------------------------------------------------------------------------------------------------------------------------------------------------------------------------------------------------------------------------------------------------------------------------------------------------------------------------------

>Bp_Cp

--------------------------------------------MTISRRDFLNGTALAIAAG-MAPAQLLAAAQGQ--------------------------------------PGAAY----------PPALT------GLRGNH-A----GTFTLAHSLAREGAKYP---TVMARESFDLVVV-------------------GGGLS---------GLAAAWFYQRRFG-----TGKR--------ILVLDNH-DDFGGHAKRNEFTVR------------------------GKRLVTYGGSAEVPPSA---------------ATDAAVRE--LFTG---LGVDAARTP-PAEQADPYAALGMGR-AVFFDADHFG--------------R-----DQWLAGDPFGEYVDARAGRQGAAAPDAAALSRFLDAA-PLPLQDRAALARLV----DG--TTDYLTGMPAG--------ERAAYLRSTRYAAFLRDKAGIGLAG--------------------------------------QRF-L-RSR-SNDTY--ALDADGISV------ADAI--GI----GLPAGAGM---PA----------PSPDPRLGKSPASRLWFPDGNASLARLL--VQRMIPGV--AK-LTRPSDIMNAAFDYGRLDQEGAPVRLRLEAT----AIAVDPDG---------------------------------------------------PITRVTYGW------------------------------------------------------------RGNLHRIEARHVVLAGWNMMVPY------------------------------LMPSLPA---TQRDTMR------AEVKAPLVYT--K--VALDHWRAFAALQ----------------------------------------TRRIHAPAMAY-TDLWLEPPAG-------------------------------------------------------------------------------------------TSPADPAVLHMLYVPTV--------------------------------------------------------------PDSGM-SARERFRAGRAFLLGTPFSELE----------------------RGIR-TQLDRMLGTAG---------------------------FASKDVIRAITVNRWAH-------GYSY-RPDSLA----------GEDA----A---TW---ASPAGV---------------------------GNVSFAGSDS----AGSPSLTGAIARGRSAVDRLPV--------------------------------------------------------------------------------------------------------------------------------------------------------------------------------------------------------------------------------------------------------------------------------------------------------------------------------------------------------------------------

>BacKv

-----------------MND--------------KKRDCALGMDRSITRRDFLNGVALTVGGA-LVAPNLLNATEKG-------------------------------------SSSEYY----------PPALM------GLRGNH-E----GTYTYAHELRDGVFQESA-QPLKTDEDYDLVIV-------------------GGGIS---------GLAAAHLYRKKAG-----KNAK--------ILILDNH-DDFGGHAKRNEFRAA------------------------NRMLLGYGGTQSIESPS---------------EYSPAAKQ--VLKD---LGIETKRFY-KDYDQKLYSHL--GT-ANFFDKETFG--------------Q-----DKLVTA--------------MFET----PWQEWVKQT-PLSEAAKRDIARVY----TE--KVDYLPGLSPK--------EKRAKLAKISYADYLTKYAKCTPEV--------------------------------------LPF-F-QSR-TNDLF--CVNIDAVPTLAILEAGDDY--GI----PYAGLDGL-GFGN----------QSQGRSEHKQEPYIFHFPDGNASVARLL--VRALMPGA--IP-GDSMEDVVTAKADYSTLDRADSPVRIRLNST----VVRAKHVGDVATS----------------------------------------------KQVEVQYMR------------------------------------------------------------DGKLQSVTGKACIMACYNMMVPY------------------------------LCPELPQ---VQKDALA------EGVKAPLVYT--H--VAIRNWDIFDKLK----------------------------------------MWQVCCPGSYH-VYVALDFPVSIG------------------------------------------------------------------------------------EYKFPSKPSEPMVLFMLRTPCK----------------------------------------------------------------PGL-SQKDQYRAGRMELFTTPYETFE----------------------RNIR-EQLSRMFGPYG---------------------------FDSARDIEGITVNRWAH-------GYAY-GYNSLF----------DPDVPE-DQR-PHI---IGRKQF---------------------------GRISIANSDA----AATAYTDAAIDMADRAVKEVLALKS-----------------------------------------------------------------------------------------------------------------------------------------------------------------------------------------------------------------------------------------------------------------------------------------------------------------------------------------------------------------------

>Bp_Cml

----------------------------------------------MQRRDFLNGMALTILAG-MTPLQVLYGKEAK---------IE-------------------------DFTKEYY----------PPKWL------GLRGSN-N----ASYEFAHML-RDGEKFDF-SAIKPKQEYDLVVV-------------------GAGIS---------GLAAACFYQNKFG-----KDKK--------ILILDNH-DDFGGHARRNEIDLE------------------------DGTILSYGGSETFQSPK------------A--LYSKEVVD--LLSS---LGVDIDELA-KRFDVNFYPDLNLSR-GVYFSKTEFG--------------V-----DKVVSGNPRKVICDDIPEGRHNGR----SVEAFIGDF-PLNEKDKKDLIALF----KS--EKDYLKGLTKE--------QRDEYVAKTSYKKFLEDKVKLSPQA--------------------------------------VKF-F-EGM-TDDFL--ALGIDAVSC------EDAR--AS----FLPGFDKL-GLDP----------IEGEALAEMEEPYIHHCADGNATVARLM--VRRLIPDV--SKKGKDMDEVTLAHFDYSKLDLAKNKVRLRLNST----VINVENTK---------------------------------------------------DGALVTYVN------------------------------------------------------------KGKNYRVKAKKVVMANYNSMIPY------------------------------IVPSMPQ---DQKDALS------KNVKTSLLHT--N--VIISNWEPFIKLG----------------------------------------VHEIYSPKMPY-ARTKLDYPVDMG------------------------------------------------------------------------------------GYHHPRDPKKPICVHMVCSPLAFASMQ-------------------------------------------------------GIDLEGM-DARDRARVGRNLLFTMSFEEHE----------------------KIVR-DQLQGMLGSAG---------------------------FDHEKDIKAIVVNRWGH-------CYSY-TENSLF----------DDSEEA-QK--TIE---LARKPF---------------------------GNIVIANSDA----DWDAYMHAAIDQAYRAVNEL----------------------------------------------------------------------------------------------------------------------------------------------------------------------------------------------------------------------------------------------------------------------------------------------------------------------------------------------------------------------------

>Bp_HdN1

--------------------------------------------MPVTRRDFLNGMALTIAAG-MTPFKQLAFADEVK-------LQA-------------------------LMSGDYY----------PPKLT------GLRGSH-D----GSFESAHQLAREHFKFSS--PRKVEESYDLVVV-------------------GAGIS---------GLAAALYYRDQFG-----PDKK--------ILVLDNH-DDFGGHAKRNEFATK------------------------EGTLLSYGGSESLQSPR------------S--VYSPAASE--FIKR---IGIELDPLE-QHFLVDLYPGMGLSR-GVFFDRKHFG--------------E-----SKLVAGDPGHQVADDIPRGKENGR----SYEAFIGDF-PLSKDDQTKLIELH----QK--PRDYLAGMAQK--------DKIHWLETHSYNDFLRQKVGLSELG--------------------------------------VLF-F-QQQ-THDFL--AMGTDVAAA------LDAR--EA----ALPGFDAL-GLPP----------LEAEYQAEIDDPYIHHFPDGNASIARLT--VRQLIPEV--AP-GASMSDIVLARFDYSQLDCLERNCRIRLNST----VIRAQHQR---DN----------------------------------------------GPVELTYVQ------------------------------------------------------------NGQLHKVLGNHCIMACYNMMIPY------------------------------LVPEMGQ---AQQDALK------RNVKSPLVYT--K--VALSNWRAFKSKG----------------------------------------VHSVYCPSAPY-CKVKLDFPSNLG------------------------------------------------------------------------------------GYRFPTSPDQPMVVHMIYVPTV--------------------------------------------------------------QGEGL-SPARQAVGGRAKLLEMSFADHE----------------------QMIR-SQLQEMFGDAG---------------------------FK-QEDILGITVNRWSH-------GYTY-IGSSLF----------DDWDEL-EK--IGE---IARQPF---------------------------GNIHIANADS----QWDAYAHAAIDAAERSVAEIRAKSQTQAS-------------------------------------------------------------------------------------------------------------------------------------------------------------------------------------------------------------------------------------------------------------------------------------------------------------------------------------------------------------------

>Bp_Al

--------------------------------------------MTITRRDFLNGAALTIAAG-FTPLQALRAAP-Q----------------------------------------LYY----------PPILT------GMRGNH-P----GSFEAAHKVGRQGHRFPL-GGLSVSEQYDLVVV-------------------GGGIS---------GLAAAYFYRQQ-H-----PKAT--------VLILDNH-DDFGGHAKRNEFHEG------------------------GHTLLTYGGSESFQSPK------------A--LFSERVNG--LLRE---LGVDADKFM-RYFDRDFYFRQGLSR-GAFFDKAHFG--------------V-----DKVIGGSPEMGIADDLRPDLLNSR----SLEAYINDF-PLPEADRRALITLH----TD--PPDYLPDMSLE--------EKEEYLATISYSTFLKQHVGLSDDA--------------------------------------MLY-F-RSM-SNEFY--GYGVDAIAA------IDAY--ET----RYPGFDAM-HLPP----------ISEEVQAELDEPYIYHFPDGNASVARLL--VRALIPNV--AP-GADMDDIVMAMFDYSQLDQPGNAVRLRLNST----AVQIDNPQ---------------------------------------------------GPVDIGYLR------------------------------------------------------------DGVLHRVQGKQCILACYNMMIPS------------------------------IMPSLPD---EQKQALH------RNVKAPLVYT--K--VLLKNWHAFKKLG----------------------------------------VHSLYAPTAPY-SLVKLDYPVNMG------------------------------------------------------------------------------------GYTHARSPDDPIIVHMVQVPTV--------------------------------------------------------------PNTGL-DVRQQLRMGRSKLLGMSFDEME----------------------AEIR-QQLGAILATVN---------------------------EPLDPLIKAITINRWSH-------GYSY-EETSLF----------DDSDQA-EQ--TIN---LARQAH---------------------------GQVAIANSDS----GWSPYMHAAIDEAWRAVNELNGEG------------------------------------------------------------------------------------------------------------------------------------------------------------------------------------------------------------------------------------------------------------------------------------------------------------------------------------------------------------------------

>Bp_SpdH

--------------------------------------------MTISRRDFLNGVALTIAAG-LTPAEILRAAPGG----------------------------------------RYY----------PPALT------GLRGSH-P----GAFEVAHQMGWEKKTFDV-DHLPIEEEYDLVVV-------------------GGGIS---------GLAAAWFYRER-H-----PAAR--------ILVIENH-DDFGGHAKRNEFQAG------------------------GRTILGYGGSESLQSPN------------A--LYSEDAKH--LLKR---LGVELKRFE-TAFDTDFYPGLGLSR-AVFFDKASFG--------------V-----DKLVSGDPTPMVADEVPRDRLNAR----SWRAFIGDF-PLSREDREALIALY----ES--PRDYLAGKSVE--------EKETYLAKTSYRDYLLKNVGLSETS--------------------------------------VKY-F-QGR-SNDFS--ALGADALPA------ADAY--AA----GFPGFDAL-GLPQ----------PSEEAQAEMDEPYIYHFPDGNASLARLM--VRDLIPAV--AP-GRGMEDIVMARFDYSKLDLAGHPVRLRLNST----AVSVRNRA---------------------------------------------------GGVDVGYSR------------------------------------------------------------AGRLHRVRGKHCVMACYNMMVPY------------------------------LLRDLSE---EQAHALS------QNVKFPLVYT--K--VLLRNWQAWKTLG----------------------------------------IHEIYAPTLPY-SRIKLDFPVDLG------------------------------------------------------------------------------------SYRHPRDPRQPIGVHMVYVPTT--------------------------------------------------------------PNAGM-DARTQARVGRSKLYAMSFEQLE----------------------KDIR-DQLQAMLGPAG---------------------------FDHRRDITGITVNRWSH-------GYSY-FMNTLY----------DDEAES-EA--LME---LARSKV---------------------------GNVAIANSDA----AWDAYAHAAIDQAVRAVRELG---------------------------------------------------------------------------------------------------------------------------------------------------------------------------------------------------------------------------------------------------------------------------------------------------------------------------------------------------------------------------

>Bp_Pf

--------------------------------------------MDITRRDFLNGVAITIAAG-MTPLQILQAAPDG----------------------------------------RYY----------PPALT------GLRGSH-V----GSFEVAHQMGWEKKAFDT-DKLPITEDYDLVVV-------------------GGGLS---------GLSAAWFYREK-H-----PKAK--------ILILENH-DDFGGHAKRNEFQAG------------------------GRLIIGYGGSEAFQSPN------------H--LYSKEVNG--LLKK---LGVNIKRFE-TAFDRQFYPGLGLSR-GVFFDKENFG--------------E-----DKLVTGDPTPMVADDIAPDQLHAR----SISDFINDF-PLPETDRQALIALH----VA--PKDYLPGKTAE--------EKAEYLAATSYRDFLLKNVGLSEGA--------------------------------------VKY-F-QSR-TNDFM--ALSIDAVAS------ADAY--SV----GFPGFGGM-NLAP----------ISEEAAAEMEEPYIYHFPDGNASLARLL--VRSLIPAV--AP-GHTMDDIVLAPFDYARLDQPRTPVRVRLNST----AVSVRNVG---------------------------------------------------DGVHIGYSR------------------------------------------------------------GGQLAQVRGKRCILACYNMMIPH------------------------------LLRDLPA---EQAQALS------QNVKYPLVYT--K--VVVRNWTSFQKLG----------------------------------------VHEIYAATQPY-SRIKLDYPVSMG------------------------------------------------------------------------------------GYEHPRDPTQPIGLHMVYVPTS--------------------------------------------------------------PNSGM-NGRDQARAGRGKLYGQTFEQLE----------------------AQLR-DQLQRMLGPGG---------------------------FNHQTDILAITVNRWSH-------GYAT-FSNSLF----------DDADES-EK--LKD---MARQPV---------------------------GHVSIANSDA----AWSAYAHAAIDEAYRAVGEVG---------------------------------------------------------------------------------------------------------------------------------------------------------------------------------------------------------------------------------------------------------------------------------------------------------------------------------------------------------------------------

>Bp_Pp

--------------------------------------------MTITRRDFLNGVALTIGAG-LTPLQILQAAPSG----------------------------------------RYY----------PPALT------GLRGSH-P----GAFEVAHQMGWEKKVFDT-SSLKIEEQYDLVVV-------------------GAGIS---------GLSAAWFYRQQ-H-----PTAR--------VLIIENH-DDFGGHAKRNEFQAG------------------------SQMILGYGGSEAFQSPK------------H--LYSATVNA--LLKR---LNVDVDRFQ-TAFDRNFYPNLGLSR-GVFFDKAGFG--------------E-----AKLVSGDPTPMVADDIAPDKLNAR----SWRAFIGDF-PLPEADRQALIDRH----EA--PRDYLAGKTLE--------QKEAYLAKTSYQDFLRKDVGLSEAA--------------------------------------ARY-F-LSR-TNDFS--ALSIDAVAA------ADAY--GV----GFPGFAAM-GLAP----------VSEALKAEMEEPYIYHFPDGNASLARLL--VRSLIPAV--AP-GNDMNDIVLAPFDYARLDVAQSPVRLRLNST----AVSVGNRN---------------------------------------------------GGVDIGYSR------------------------------------------------------------AGQLHRVHGKHCILACYNMMIPY------------------------------LLRDLTA---EQAHALA------QNVKFPLVYT--K--VVIRNWHSFMKLG----------------------------------------VHEIYAPNQPY-SRVKLDYPVDIG------------------------------------------------------------------------------------GYSHPRDPDQPIGLHMVYVPTT--------------------------------------------------------------PNASM-DARSQARVGRSKLYAMNFEQME----------------------AMVR-DQLQAMLGPAG---------------------------FDYHKDVQAITINRWPH-------GYSY-FANSLF----------DDEEES-EK--LMN---LARQKV---------------------------GNVAIANSDA----AWEAYAHAAIDEASRAVNELFA--------------------------------------------------------------------------------------------------------------------------------------------------------------------------------------------------------------------------------------------------------------------------------------------------------------------------------------------------------------------------

>Bp_Cv

--------------------------------------------MSITRRDFLNGFALTVAAG-LTPLEILRAAPRQ-------------------------------------ISASYY----------PPSLT------GLRGNH-D----GSFENAHKLGRAHHAVNL-GGAKVEEHYDLVVV-------------------GGGIS---------GLAAAHYYRKK-H-----PQAK--------VLILDNH-DDFGGHAKRNEFKVG------------------------NRLMLGYGGTESLQSPK------------H--VFSKEALA--LMKE---LTIDIDGLA-SHFHRNFYPDLKLSR-GVFFNKEDFG--------------V-----DKTVGGDPYLQVADDIDPKRLNAR----PIRDFINDF-PMSKKDRDALIALH----TS--TRDYLGDMPKN--------KREAYLAKISYSTYLSRHVGLSATA--------------------------------------IRF-F-QSR-SNDFQ--AVGIDGLPA------LDAR--NL----DLPGFHGVAGLGK----------ISAEAEAELRDPYIYHFPDGNASIARLL--VRKMIPQA--AP-GKDMNDIVLARFDYSKLDLPENPVRLRLNST----VVRVENAK---------------------------------------------------GPVDVGYLD-----------------------------------------------------------KGGRLRRVQGRHVVMACYNMMIPH------------------------------IMPELEN---DQKQALA------MNVKAPLVYT--K--VVLKHWDSFMKLG----------------------------------------VHELYCPSMPY-SRIKMDYPVDLG------------------------------------------------------------------------------------GYQHPRKPSEPMCLHMVYVPTI--------------------------------------------------------------AGSNL-DPRTQWKMGRAKLLAMPFSEHE----------------------NMIR-SQLQRILGPVG---------------------------FDHNRDIAAITVNRWSH-------GYSY-FLNSMY----------DDEEES-KR--IIA---AARRPA---------------------------GKVAIANSDS----DWNPYTHAAIDQAWRAVNELS---------------------------------------------------------------------------------------------------------------------------------------------------------------------------------------------------------------------------------------------------------------------------------------------------------------------------------------------------------------------------

>Bp_Sf

--------------------------------------------MAITRRDFLNGVAVTIAAG-LTPLDLVRAAGKG---------HE-------------------------FIDGSYY----------PPALT------GLRGNH-P----GSFEMAHALGREHKHFDL-STLPIEEEYDLVIV-------------------GGGIS---------GLAAGCFWHELAG-----KNSK--------VLILDNH-DDFGGHAKRNEFTSA------------------------GKKLIGYGGSEAFQSPA------------H--NFSPEVNK--LMET---VGVSVTRLK-KSFDVNFYPDWQLSR-GVFFDKKNFG--------------E-----TKIVSGDPGRAVSDDIPPDRLNGR----NIEDFINDF-PLSEEDRKALIDLH----VH--PVDYLAGMTVD--------EKTEWLDTHSYSEFLSTRVGLSKMA--------------------------------------LLY-F-QQR-SNDFF--AIGIEGISG------SDAR--AC----ALPGMEAL-GLPP----------LDGESLADLEEPYVYHFPDGNAGLARLL--VRHMLPDA--LP-GNTMEDSVLARLDYEKLDLPENTTRLRLNST----VINAANVE---------------------------------------------------GALPSPGYA-------------------------------------------------------------------MAKCIACAVRTPLWRA------------------------------IT---------------------------------------------------------------------------------------------------------------------------------------------------------------------------------------------------------------------------------------------------------------------------------------------------------------------------------------------------------------------------------------------------------------------------------------------------------------------------------------------------------------------------------------------------------------------------------------------------------------------------------------------------------------------------------------------------------------------------------------------------------------------------------------------------------------------------------------------------------------------------------------------------

>Bp_Eb

--------------------------------------------MSITRRDFLNGVALTVVAG-LTPWDLARAEENG---------IA-------------------------EKAGEYY----------PPSLM------GLRGNH-P----GSFEMAHALGREHQKFDF-DSLTVEEEYDLVIV-------------------GGGIS---------GLASACFWQQVAG-----KDKK--------ILILDNH-DDFGGHAKRNEFTAG------------------------KSTLIGYGGSESFQSPK------------H--NFSPEVNK--LLAD---VGISVVKMK-AGFDQNFYPDLNLSR-GVFFDKKNFG--------------E-----TRIVSGDPGRAVSDDIPADRLNGR----KIEDFINDF-PLSETDKAALIELH----VR--PKDYLPGMKVD--------EKLAWMDKHSYSEFLSEKVGLSKIA--------------------------------------LLY-F-QQR-TNDFF--AIGIEGISC------SDAR--VC----ALPGMEAM-HLPP----------LDEESLADLEEPYTYHFPDGNAGLTRML--VRHLIPDA--LP-GESMADAVTSTLDYSRLDTKATSTRLRLNSI----VINAKNTD---------------------------------------------------NGVIVTYIH------------------------------------------------------------QGHPYRVRGRNTIMAGYNMMIPF------------------------------LVPETPE---EQKAYLR------MNVKAPLVYT--N--VAIRNWRSFTDLG----------------------------------------IHEFYSPAAPY-SRVKLDYPVSMG------------------------------------------------------------------------------------KYHHPRNADDPMLIHMVYVPTY--------------------------------------------------------------PGSNL-TAREQFQRGRAWLLGTTFETHE----------------------QMIR-EQLQEMLGSTG---------------------------FDHQRDIAGITVNRWAH-------GYAY-YASSLF----------DDMDKM-PE--IIK---EARKPI---------------------------GRIAIANSDS----DWSAYAHAAIDQAWRAVNELKNLG------------------------------------------------------------------------------------------------------------------------------------------------------------------------------------------------------------------------------------------------------------------------------------------------------------------------------------------------------------------------

>Bp_En

--------------------------------------------MSITRRDFLNGMAIAIVSG-LTPLELVRANGKAS-------GSA-------------------------VINADYY----------PPGLT------GLRGNH-P----GSFEMAHALGREHEKFDF-GKLPVEEEYDLVVV-------------------GGGIS---------GLAAACFWREKMG-----KEAK--------ILILDNH-DDFGGHAKRNQFNVG------------------------GKTLLGYGGSESFQSPN------------S--NFSPVVHG--LMDS---LGVSITRMK-SSFDATFYPDQNLSR-GVFFDKKNFG--------------E-----TKIVSGDPGRAVSDDIPPDRLNGR----SIEAFINDF-PLSEADRKALLDLH----VN--PADYLPGMTVE--------QKSEWLDVHSYHEFLEQKVGLSKMA--------------------------------------LMY-F-QQR-TNDFF--AIGIEGVSC------SDAR--AC----ALPGMEAM-GLPP----------LDGEALADLEEPYTYHFPDGNAGLTRLM--VRKLIPDA--LP-GSTMEDSVTARLHYELLDRPENGTRIRLNSS----VINAANAD---------------------------------------------------NGVVVSYIN------------------------------------------------------N----QSGKLHRVKSRNAIMAGYNMMIPY------------------------------IVPEAPE---QQKEDLR------LNVKAPLVYT--N--VVVNNWRAFKNTG----------------------------------------VHEFYSPAAPY-SRVKLDYPVSMG------------------------------------------------------------------------------------DYHHPQTPDDPMCIHMVYVPTY--------------------------------------------------------------PGSNM-SPREQFRRGRAFLLGSSFEAHE----------------------QMIR-SQLQEMLGHTG---------------------------FDHERDIAAITVNRWAH-------GYAY-YANALS----------DDMEKM-PE--IIN---RARQPI---------------------------GRITIANSDS----DWSAYAHAAIDQAWRAVNELVAMG------------------------------------------------------------------------------------------------------------------------------------------------------------------------------------------------------------------------------------------------------------------------------------------------------------------------------------------------------------------------

>Bp_Yor

--------------------------------------------MAITRRDFLNGVAITIAAG-LTPLDLVRAAGKA---------GA-------------------------VIHGEYY----------PPALT------GLRGNH-P----GSFEMAHALGREHENFAL-DGLPVEEEYDLVIV-------------------GGGIS---------GLAAACFWQQLAG-----KESK--------ILVLDNH-DDFGGHAKRNEFTVE------------------------GKTLIGYGGSEAFQSPA------------N--NFSPVVNK--LMAD---LGISVSRMK-AGFDVNFYPDHQLSR-GVFFDKKHFG--------------E-----TKIVSGDPGRAVSDDIPHDRLNGR----PIEAFINDF-PLSEEDRKALITLH----VN--PSDYLPGMTVE--------EKSAWLDGHSYHEFLSEKVGLSKMA--------------------------------------LLY-F-QQR-SNDFF--AIGIEGISC------ADAR--AC----ALPGMEGM-GLPP----------LDGEALADLEEPYVYHFPDGNAGLTRLM--IRHLIPQA--LP-GSTMEDSITSRLDYSTLDKPQNPARIRLNSV----VVNAANTD---------------------------------------------------DGVVVTYLN------------------------------------------------------------DGKPHRVRGRNAVMAGYNMMVPY------------------------------LVPETPE---EQKDDLR------LNVKAPLVYT--N--VVVKNWQPFMKLG----------------------------------------IHEFYSPAAPY-SRVKLDYPVSLG------------------------------------------------------------------------------------SYQHPQSPDEPMVLHMVYVPTY--------------------------------------------------------------PGSNM-SAREQFRKGRAFLLGTSFDAHE----------------------KMIR-DQLQEMLGSAG---------------------------FDHERDIAAITVNRWAH-------GYAY-YANALF----------DDMDKQ-AE--IVE---RARKPV---------------------------GRIAIANSDS----DWSAYAHAAIDQAWRAVNELKAMG------------------------------------------------------------------------------------------------------------------------------------------------------------------------------------------------------------------------------------------------------------------------------------------------------------------------------------------------------------------------

>Bp_Ah

--------------------------------------------MSITRRDFLNGVAIAIAAG-VAPIQLLQAAEGGK-------AL--------------------------AEKTLVY----------PPALT------GLRGNH-P----GSFEPAHSIARDGKQYDF-AHLPLEGEYDLVIV-------------------GAGIS---------GLSAACFYQQLLG-----ADKK--------ILLLDNH-DDFGGHAKRNEFTTP------------------------DGLRLGYGGSESLQSPR------------S--VYSPVALG--LLKA---LEVNIDELA-KGFQQTFYPDLGLSR-GVFFDEKHFG--------------V-----NKIVSGDPGHSVADDIPRDRLNGR----PLAEFIGDF-PFDDTDKAALLALH----EE--KTDYLHGMTRE--------QKDAWMESNSYTTFLRDKVGLSERA--------------------------------------ITF-F-QQR-TDDFQ--AVGIDATSC------ADAR--LC----ALPGFAGM-DLTP----------LDAESQAELDDPYIFHFPDGNAGLTRLM--VRKLIPQV--AP-GHTMQDVVLARFDYSQLDRPEHKVQLRLGST----ALQARNVPLKDGK----------------------------------------------QGVDVTYIK------------------------------------------------------------EGKLHRVQARQCIMAGYNMMIPY------------------------------LVPEMPE---PQKEALR------QNVKAPLVYT--K--VVIRNWQPFVKLG----------------------------------------VHEVYAPAAPY-SRVKLDYPVDLG------------------------------------------------------------------------------------GYEHPKNPDQPMCLHMVYVPTL--------------------------------------------------------------PGSGL-SAREQSRKGRAMLLGMPFEQHE----------------------QMIR-EQLQAMLGEAG---------------------------FNHEQDILAITVNRWSH-------GYSY-IANSLF----------DDEEQS-ER--FIE---RGRQPV---------------------------GNITIANSDA----GWSPYAHAAIDEAWRAVNELVALTKGGAK-------------------------------------------------------------------------------------------------------------------------------------------------------------------------------------------------------------------------------------------------------------------------------------------------------------------------------------------------------------------

>Bp_As

--------------------------------------------MSITRRDFLNGVAITIAAG-IAPINLLQAAEGGK-------AL--------------------------ADKTLAY----------PPALT------GLRGNH-P----GSFEPAHSIARDGKQYDF-ANVPLEGEYDLVIV-------------------GAGIS---------GLAAACFYQQLLG-----ADKK--------ILLLDNH-DDFGGHAKRNEFTTP------------------------DGLRLGYGGSESLQSPR------------S--VYSPIALG--LLKT---LEVNIDELA-AGFQQTFYPDLGLSR-GVYFDEKHFG--------------V-----NKVVSGDPGHNVADDIPRDRLNGR----PLAEFIGDF-PLDERDRAALLALH----EE--KIDYLSGMSRE--------EKDLWMTRNSYTSFLRDKVGLSERA--------------------------------------ITY-F-QQR-TNDFQ--AVGIDATAC------ADAR--LC----ALPGFDGL-DLTP----------LDAEEQAELDDPYIFHFPDGNAGLTRLM--VRKLIPQV--AP-GHTMQDVVLAKFDYGKLDLPEHKVQLRLGST----ALQAKNIRRTDGS----------------------------------------------TGVDVTYIK------------------------------------------------------------EGKLHRVRAKQSVMAGYNMMIPY------------------------------MVPEMAE---PQKEALR------QNAKAPLVYT--K--VVIKNWQSFVKLG----------------------------------------VHEVYSPAAPY-SRVKLDYPVNLG------------------------------------------------------------------------------------GYEHPKNPDQPMCLHMVYVPTL--------------------------------------------------------------PGSGL-SAREQSRKGRAMILGMPFEQHE----------------------QMIR-EQLQGMLGSAG---------------------------FNHEQDILAITVNRWSH-------GYSY-ITNTLF----------DDEAQC-EK--WIE---LGRQPI---------------------------GNITIANSDS----DWSPYAHSAIDQAWRAVNELVAMQKGGAQ-------------------------------------------------------------------------------------------------------------------------------------------------------------------------------------------------------------------------------------------------------------------------------------------------------------------------------------------------------------------

>Bp_So

--------------------------------------------MGITRRDFLNGVAITIAAG-LTPLQILRASPQT------------------------------------ANQTLYY----------PPALT------GLRGNH-P----GSFEHAHQLGRDGKAFDF-GSVPATEEFDLIVV-------------------GAGIS---------GLAAACFWQQMKG-----TQQR--------ILLIDNH-DDFGGHAKRNEFSSE------------------------NGTILGYGGSESLQSPR------------S--NFSPVAMG--LLQK---LGISIDNLE-KAFDKTFYPDLNLSR-GVYFDRKNFG--------------V-----DKVVNGDPGRMVADDIPRDRLNGR----SYEAFIGDF-PLPESDRQALIALH----TV--DKDYLPDLTQE--------QKSEWLDKHSYTQFLREKVGLSEMA--------------------------------------IRY-F-QQT-TSDFQ--AVGIDATSC------SDAR--IC----DLPGLNGM-NLPP----------LDEESQADLDDPYVFHFPDGNATLARLM--VRHLIPAV--APGGKDMNDIVLAKFDYSRLDRPESPVKLRLNST----GLHAANVG---------------------------------------------------DKVEVTYMT------------------------------------------------------------GEKMTKVRAGQVVMAGYNMMIPY------------------------------LVPEMSH---EQQEALK------QNVKSPLVYS--K--VVIRNWQPFIKLG----------------------------------------VHEVYSPTAPY-CRVKLDYPVSMG------------------------------------------------------------------------------------GYEHPRDPNQPIGLHMVYVPTL--------------------------------------------------------------AGSGL-SPREQSRKGRALLLGTPFEVHE----------------------QMIR-EQLQGMLGSAG---------------------------FDHQRDIQAITVNRWSH-------GYSY-FLNGLF----------DDEEEA-KK--IIE---TARKPI---------------------------GKIVIANSDS----DWSPYANSAIDQAWRAVNELAFGQAAVKEGA-----------------------------------------------------------------------------------------------------------------------------------------------------------------------------------------------------------------------------------------------------------------------------------------------------------------------------------------------------------------

>Bp_Sp

--------------------------------------------MSITRRDFLNGVAITIAAG-LTPMQILRASPQT------------------------------------ANQTLYY----------PPTLT------GLRGNH-P----GSFEHAHQLGRDGKAFDF-ASIPATEEFDLVVV-------------------GAGIS---------GLAAACFWQQMKG-----QQQR--------ILLIDNH-DDFGGHAKRNEFSSE------------------------NGTILGYGGSESLQSPR------------S--NFSPVAMR--LLQK---LGVSIDNLE-KAFDKTFYPDLNLSR-GVYFDRKNFG--------------V-----DKVVNGDPGRMVADDIPHDRLNGR----SYEAFIGDF-PLPESDRQALIALH----TV--DKDYLPEMSQE--------QKSEWLDKHSYTEFLRDKVGLSEMA--------------------------------------IRY-F-QQT-TSDFQ--AVGIDATSC------SDAR--IC----DLPGLNGM-NLPP----------LDEESQADLDDPYVFHFPDGNATLTRLM--VRHLIPAV--APGGKDMNDIVLAKFDYSQLDRAESPVKLRLNST----GLHAANVG---------------------------------------------------DKVEVTYMT------------------------------------------------------------GEKMTKVRAGQVVMAGYNMMIPY------------------------------LVPEMSP---EQQLALK------QNVKSPLVYS--K--VVIRNWQSFIKLG----------------------------------------VHEVYSPTAPY-CRVKLDYPVSMG------------------------------------------------------------------------------------GYQHPRDPNQPIGLHMVYVPTL--------------------------------------------------------------AGSGL-SPREQSRKGRALLLGTPFEVHE----------------------QMIR-EQLQGMLGSAG---------------------------FDHQRDIEAITVNRWSH-------GYSY-FLNGLF----------DDEDEA-KK--IIE---TARKPI---------------------------GKIVIANSDS----DWSPYANSAIDQAWRAVNELAFGQVAAKEGA-----------------------------------------------------------------------------------------------------------------------------------------------------------------------------------------------------------------------------------------------------------------------------------------------------------------------------------------------------------------

>Bp_Yer

--------------------------------------------MGITRRDFLNGVAIAVTAG-MTPFEALKASPQT------------------------------------AAQTLYY----------PPSLT------GLRGNH-Q----GSYEAAHILGREDKKVDP-SSLPVEDEFDLVIV-------------------GAGIS---------GLAAACFWQEQHG-----KQQR--------ILLLDNH-DDFGGHAKRNEFHVE------------------------DKTLLGYGGSESFQSPR------------T--NFSPVAMG--LLKT---LNVDIETMA-KDFNQPFYPDLHLSR-GVYFDRKNFG--------------V-----DKLVSGDPGRAVADDIPPDRLNGR----DIRDFINDF-PLPESDRKALIALH----TE--EKDYLHGMSQE--------EKVAWVDRHSYTQFLREKVGLSGMA--------------------------------------IRY-F-QQR-TNDFQ--AVGIDATSC------SDAR--IC----ALPGLESM-GLPP----------LDAESLEDLEQPYIFHFPDGNAGLARLM--VRHLIPPV--AP-GKTMEDIVLAKFDYSQLDKSGQPVRLRLNST----CMHVANVDH-EGK----------------------------------------------PAVEVTYMT------------------------------------------------------------GTHLHRIRAGQAVMAGYNMMIPY------------------------------LVPEIAE---EQKAALK------ENVKAPLLYT--K--VVIRNWQPFIKLG----------------------------------------VHEIYSPGAPY-SRVKLDYPVDMG------------------------------------------------------------------------------------GYQHPRDPNQPIGLHMVYVPTL--------------------------------------------------------------PGSGL-SPREQSRKGRALLLGTSFDAHE----------------------KMIR-NQLQGMFGEVG---------------------------FDHQHDIMAITINRWSH-------GYSY-FLSGMF----------DDEEQS-QK--TIQ---LARQPV---------------------------GRITIANSDS----DWSPYANSAIDQAWRAVNELTAMKKVNA--------------------------------------------------------------------------------------------------------------------------------------------------------------------------------------------------------------------------------------------------------------------------------------------------------------------------------------------------------------------

>Bp_Cs

--------------------------------------------MAITRRDFLNGVAITVAAG-MTPWQILRASPQT------------------------------------AAQSLYY----------PPTLT------GLRGNH-P----GSFEQAHALGREGKHFDP-ASVPVEEEYDLVIV-------------------GAGIS---------GLAAACFWQELRG-----KQQR--------ILLLDNH-DDFGGHAKRNEFHVD------------------------GKTLLGYGGSESFQSPA------------N--NFSETAMG--LLKT---LNVSIERMA-KSFDQTFYPDLNLSR-GVYFDKTNFG--------------V-----DKIVSGDPGRAVADDIPPDRMNAR----DIRAFINDF-PLPQADRDALIALH----TE--KKDYLAGMRVE--------DKVAWLDSHSYSQFLRDKVGLSDSA--------------------------------------IRY-F-QQR-TNDFQ--AIGIDGTSA------SDAR--IC----ALPGLDGM-DLPP----------LDAESLADLEEPYIYHFPDGNAGLARLM--VRHLIPAV--AP-GNSMDDIVLAPFDYSQLDKPEHPVRLRLNST----GVHAANVA---------------------------------------------------GGVEVTYLR------------------------------------------------------------DGKLHKVKAGQTVMAGYNMMIPY------------------------------LVPEIPH---DQQEALK------QNVKAPLVYS--K--VVIRNWQPFMKLG----------------------------------------VHEIYSPAAPY-SRVKLDYPVDMG------------------------------------------------------------------------------------GYQHPRDPNAPIGLHMVYVPTF--------------------------------------------------------------PGSGL-SAREQFRKGRAFLLGTPFEVHE----------------------KMIR-DQLQGMFGAAG---------------------------FDHERDIAAITVNRWSH-------GYSY-FFSGLF----------DDEEGS-QK--IIE---KARQPV---------------------------GRITIANSDA----DWSPYANSAIDQGYRAVKELHEMAKEGA--------------------------------------------------------------------------------------------------------------------------------------------------------------------------------------------------------------------------------------------------------------------------------------------------------------------------------------------------------------------

>Bp_Ha

--------------------------------------------MSITRRDFLNGMAITIAAG-LTPWQALRASPQA------------------------------------LTQSLYY----------PPTLT------GLRGNH-P----GSFEAAHLLGREGKHFDP-KSVPVEEQFDLVVV-------------------GAGIS---------GLAAACFWQQLQG-----KNQR--------ILLLDNH-DDFGGHAKRNEFNVE------------------------GKTILGYGGSESFQSPR------------T--NFSPVAMG--LLKT---LNVDIEQMA-KDFDQNFYPDLKLSR-GVYFDRKNFG--------------V-----DKIVNGDPGRAVADDIAPDRLNGR----DITAFINDF-PLSESDRKALIALH----TE--QKDYLPELNTD--------EKVAWLDSHSYSQFLREKVGLSEIA--------------------------------------IRY-F-QQR-TNDFQ--AVGIDATSC------SDAR--IC----ALPGLDGM-NLPP----------LDAESLADLDEPYIFHFPDGNAGLARLM--VRHLIPAV--AP-GDSMESIVLAKFDYSQLDKKDSPVRLRLNST----GVHVANVSE-QGK----------------------------------------------QAVDVTYLT------------------------------------------------------------GDKLHRVRAGQVVMAGYNMMIPY------------------------------LVPEMPE---KQAAALK------ENVKSPLVYS--K--VVIRNWQPFMKLG----------------------------------------VHEIYSPAAPY-SRVKLDYPVNMG------------------------------------------------------------------------------------GYEHPRDPNQPIGLHMVYVPTL--------------------------------------------------------------PGSGL-SPREQSRKGRALLLGTPFEVHE----------------------KMIR-EQLQGMFGAAG---------------------------FDHQRDIQAITVNRWSH-------GYSY-FLNGLF----------DDEKEA-EQ--IIH---TARQPI---------------------------GRITIANSDS----DWSPYANSAVDQAWRAVNELTAMNKENV--------------------------------------------------------------------------------------------------------------------------------------------------------------------------------------------------------------------------------------------------------------------------------------------------------------------------------------------------------------------

>Pd_Gm1

------------------------------------------------------------------------------------------------------------------------------------------------------------------------------MESPSRSFVIIV-------------------GAGVS---------GISAAKLLAENG-------VKD--------LVILEAS-NCIGGRIRKENFGGV---------------------------SVEL-GAGWIVGVG-------------GKESNPIWEL--V-AE---YGLRTCFSD-YT-NV-----------------------------------------PYNIYDRSGKIFSSGI-------------AADSYKKAVD--SAIRNLTNQEE---------------------------ADREGNS-----------SKTTEPP-------------------------------SSPLELAIDFIL-----HDFEM--AE----------------AV-------PISTFT------AF----------------GER-EFLVADERGFDYLVYKM--AEDFLLT----------------------SEGKILDTRLKLNHV----VREIEHRGS---------------------------------------------------GVRVITED-------------------------------------------------------D---C-----IYEANYVLVSVSIGVLQS--------------------------NLVAFHPPLPR---WKLEAIE-KCDVTVYTKIFLKFP--Y-----QFWPSG-PG---------------------------------------------------NEFFIYAHD-Q---------------------------------------------------------------------------------------------RGYYTFWQQM---------------------------------------------------------------------ENAY-P---GSDILVVTLTNGESKRVEA----QSD------EDTL----REAM-EVLKDMFG-PN------------------------------IPDATDILVPRWWNNRFQ-RGSYSN-YPVIS----------------N--LQ-VVR---DVKAPV---------------------------GRIFFTGEHTSE--RFSGYVHGAYLAGINSSKELLEEMRKDNKRKNKS--Q-SRVL----------------EPLSALTECNIPRQ------------------------------------------------------------------------------------------------------------------------------------------------------------------------------------------------------------------------------------------------------------------------------------------------------------------------

>Pd_At1

---------------------------------------------------------------------------------------------------------------------------------------------------------------------------------MSTASVIII-------------------GAGIS---------GISAAKVLVENG-------VED--------VLILEAT-DRIGGRIHKQNFGDV---------------------------PVEL-GAGWIAGVG-------------GKESNPVWEL--A-SR---FNLRTCFSD-YT-NA-----------------------------------------RFNIYDRSGKIFPTGI-------------ASDSYKKAVD--SAILKLKSLEA---------------------------QCSG-----------QVAEEAPSSP-------------------------------KTPIELAIDFIL-----HDFEM--AE----------------VE-------PISTYV------DF----------------GER-EFLVADERGYECLLYKM--AEEFLVT----------------------SHGNILDYRLKLNQV----VREVQQSRN---------------------------------------------------GVVVKTED-------------------------------------------------------G---S-----VYEANYVIVSASIGVLQS--------------------------DLLSFQPLLPR---WKTEAIQ-KCDVMVYTKIFLKFP--Q-----CFWPCG-PG---------------------------------------------------QEFFIYAHE-Q---------------------------------------------------------------------------------------------RGYFTFWQHM---------------------------------------------------------------------ENAY-P---G----------SNIKRVEA----QSD------QETM----KEAM-SVLRDMFG-AT------------------------------IPYATDILVPRWWNNRFQ-RGSYSN-YPMIS----------------D--NQ-LLQ---NIKAPV---------------------------GRIFFTGEHTSE--KFSGYVHGGYLAGIDTSKSLLEEMKQ------------SLLLQPLLAFTESLTLT---------HQKPNNSQIYTN---V-------K---FIS------GTS-----------------------------------------------------------------------------------------------------------------------------------------------------------------------------------------------------------------------------------------------------------------------------------------

>Pd_Br1

---------------------------------------------------------------------------------------------------------------------------------------------------------------------------------MTTASVIII-------------------GAGIS---------GITAAKELAEKG-------VED--------VLILEAT-ERIGGRIQKQSFGDV---------------------------SVEL-GAGWIAGVG-------------GKESNPVWEL--A-SR---LNLRTCFSD-YT-NA-----------------------------------------RYNIYDQSGEIFPTGF-------------AADSYKKAVD--SAILKLKSLEA---------------------------ECDG-----------QEAEEAPSSP-------------------------------KTPIELAIDFIL-----HDFEM--AE----------------VE-------PISTYV------DF----------------GER-EYLVADERGYESLLYKM--AEGFLYT----------------------SDGNILDNRLKLNKV----VREVQQSRN---------------------------------------------------GVVVKTED-------------------------------------------------------G---S-----EYEANYVIVSASIGVLQS--------------------------NLISFQPPLPK---WKTEAIQ-KCDVMVYTKIFLKFP--R-----CFWPCG-PG---------------------------------------------------QEFFIYAHE-Q---------------------------------------------------------------------------------------------RGYFTFWQHM---------------------------------------------------------------------ENAY-P---GSNILVVTLTNEQSKRVES----QSD------EETL----KEAM-SVLRDMFG-PT------------------------------IPYATDILVPRWWNNRFQ-RGSYSN-YPMIS----------------D--NQ-LQR---NVKAPF---------------------------GRIFFTGEHTSE--KFSGYVHGGYLAGMDTSKTLLEEMKQ------------SLLLQPLLAFTESLTQT---------DQRSNPQMY-SN---V-------N---LIS------GRS-----------------------------------------------------------------------------------------------------------------------------------------------------------------------------------------------------------------------------------------------------------------------------------------

>Pd_Md1

------------------------------------------------------------------------------------------------------------------------------------------------------------------------------MDSPSSSSVIIV-------------------GAGVS---------GLSAAKVLIENG-------VED--------VVILEAS-DRIGGRIRKQDFGGV---------------------------SVEL-GAGWIVGVG-------------GRELNPVLDL--A-LK---SNLRTIFSD-YS-NA-----------------------------------------RYNIYDSGKI-FPR-G-------------LEETYKKEVE--SAVQKLKKLE-------------------------------AGGG------DFSNVTEPPTTQ-------------------------------KTPIELAIDFTL-----HDFEM--PE----------------VE-------PISTFL------DY----------------GER-EFLVADERGYEHMLYKM--AEDVLFT----------------------SEGKLLDSRLKFNKV----VRELQHSRN---------------------------------------------------GVTVMTED-------------------------------------------------------G---C-----VFQANYMILSVSIGVLQS--------------------------NLIAFNPPLPR---WKTEAIQ-KCDVIVYTKIFLKFP--Y-----KFWPCG-PG---------------------------------------------------QEFFLYAHE-R---------------------------------------------------------------------------------------------RGYYTFWQHM---------------------------------------------------------------------ENAY-P---GSNMLVVTLTNGESKRVEA----QSD------KETL----NEAM-AALKDMFG-PD------------------------------IPEATDILVPRWWNNRFQ-RGSYSN-YPMIS----------------D--NQ-FVH---DIKNPV---------------------------GRLFFTGEHTSE--KFSGYVHGGHLAGIETGKALLEEMEKEKERTSESENQ-AFLLEPLLALTGSLSLTQN-DAVSS-LKCDIPKRSYLS-----------G---KVG------IAELCYD-------------------------------------------------------------------------------------------------------------------------------------------------------------------------------------------------------------------------------------------------------------------------------------

>Pd_Pr2

------------------------------------------------------------------------------------------------------------------------------------------------------------------------------MESPSRSSVIIV-------------------GAGVS---------GLSAAKVLIENG-------VED--------VVILEAS-DRIGGRIRKQDFGGL---------------------------SVEL-GAGWIVGVG-------------GRESNPVWEL--A-QK---SNLRTFFSD-YS-NA-----------------------------------------RFNIYDQSGKIFPSGI-------------AADSYKKAVE--SAMQKLKKSEA---------------------------DSCYGGG------DVTKAAESSSTP-------------------------------KTPIELAIDFIL-----HDFEM--PE----------------GE-------PISTFQ------DF----------------GER-EFLVADERGYEHLLYKM--AGEFLFT----------------------SEGKLLDNRLKFNKV----VRELQHSRN---------------------------------------------------GVTVMTED-------------------------------------------------------G---C-----VYEASYVILSVSIGVLQS--------------------------ELIAFNPPLPR---WKTEAIQ-KCDVIVYTKIFLKFP--Y-----KFWPCG-PG---------------------------------------------------KEFFIYAHE-R---------------------------------------------------------------------------------------------RGYYTFWQHM---------------------------------------------------------------------ENAY-P---GSNILVVTLTNEESKRVEA----QSD------KETL----KEAM-GALRDMFG-LN------------------------------IPEATDIFVPRWWNNRFQ-RGSYSN-YPVIS----------------N--GQ-VVR---DIKAPL---------------------------GCIFFSGEHTSE--RYSGYVHGGYLAGIETGKALLEEIEKEKERSIESENQ-TFLLEPLLALTGSLSLTQN-DAVSS-LKCDIPRQLYLS-----------G---KVG------APELYYDYVDL---------------------------------------------------------------------------------------------------------------------------------------------------------------------------------------------------------------------------------------------------------------------------------

>Pd_Gm3

------------------------------------------------------------------------------------------------------------------------------------------------------------------------------MDSPSRSSVIIV-------------------GAGIS---------GIAAAKVLAENG-------VED--------LVILEAS-DRVGGRICKESFGGV---------------------------TVEL-GAGWIAGVG-------------GQQPNPIWEL--A-AQ---FELRTCFSD-YS-NA-----------------------------------------RYNIYDRSGNIIPSEI-------------AADSYKKAVD--SAIQKLRNQEE---------------------------EEEAYGDDHCNNNIKNSETKLPSTP-------------------------------ETPIELAIDFIL-----HDFEM--AE----------------VE-------PISTYV------DF----------------GER-EFLVADERGYDYLLYKM--AEEFLFT----------------------SEGRILDNRLKLNKV----VRELQYSKS---------------------------------------------------GVTVKTED-------------------------------------------------------G---F-----VYEANYVILSVSIGVLQS--------------------------DLLAFNPTLPR---WKLDAIE-KCDVMVYTKIFLKFP--Y-----KFWPSG-PD---------------------------------------------------KEFFIYAHE-R---------------------------------------------------------------------------------------------RGYYTFWQHM---------------------------------------------------------------------ENAY-P---GSNMLVVTLTNEESKRVEA----QAD------EETL----REAM-AVLRDMFG-PN------------------------------IPNAIDILVPRWWNNRFQ-RGSYSN-YPIIS----------------N--HK-LFH---NIKAPV---------------------------GRIFFTGEHTSE--RFNGYVHGGYLAGIDTSKALLEEMRKEKESQT-L-L--------LEPLLASLTMSKP-ETVSNIHKCDIPTQLYLS-----------G---KLG------VPEAIL--------------------------------------------------------------------------------------------------------------------------------------------------------------------------------------------------------------------------------------------------------------------------------------

>Pd_Ca5

------------------------------------------------------------------------------------------------------------------------------------------------------------------------------MDSSTRSSVIVV-------------------GAGIS---------GISAAKVLAENG-------VED--------IVILEAS-DRIGGRIRKECFGGV---------------------------SVEL-GAGWIAGVG-------------GRESNPVWEL--A-VE---QNLRTCYSD-YS-NA-----------------------------------------RYNIYDQSGKIIPSGI-------------AADSYKKAVD--SAIQKLRNEEE---------------------------AEANDDANINGGNDSNKITKPPSTP-------------------------------KTPVELAIDFIL-----HDFEM--AE----------------VE-------PISTYV------DF----------------GER-EYLVADERGYDYLLYKM--AEDFLFT----------------------SEGRILDDRLKLNKV----VRELQHSRN---------------------------------------------------GVTVITED-------------------------------------------------------G---C-----VYEANYVILSVSIGVLQS--------------------------DLIAFNPPLPR---WKFEAIE-KCDVMVYTKIFLKFP--Y-----RFWPSG-PE---------------------------------------------------KEFFIYAHE-R---------------------------------------------------------------------------------------------RGYYTFWQHM---------------------------------------------------------------------ENAY-P---GSNILVVTLTNEESKRVEA----QSD------EETL----RESM-AVLRDMFG-PN------------------------------IPDAIDILVPCWWNNRFQ-RGSYSN-YPIIS----------------N--RK-VFH---NIKAPV---------------------------GHIFFTGEHTSE--KFNGYVHGGYLAGIDTGKALVEEIRKEKEREK-E-SQ-TLLLEPLLALTGSLTMSKS-ETVSNIHKCDIPTQLYLS-----------G---KLG------IPEAIL--------------------------------------------------------------------------------------------------------------------------------------------------------------------------------------------------------------------------------------------------------------------------------------

>Pd_Mt1

------------------------------------------------------------------------------------------------------------------------------------------------------------------------------MDSTNRSSVIII-------------------GAGIS---------GISAAKVLSENG-------VED--------IVMLEAS-DRIGGRIRKECFGGV---------------------------SVEL-GAGWIAGVG-------------GREANPVWEL--A-VQ---HNLKTCFSD-YS-NA-----------------------------------------RFNIYDQSGKLIPSGI-------------ADDSYKKAVE--SAIQKLRDEEV---------------------------EEDDEEA----NDDGNKVTKPSLTP-------------------------------KTPVELAIDFIL-----HDFEM--AE----------------VE-------PISTYV------DF----------------GER-EFLVADERGYDHLLYKM--AEGFLFT----------------------SEGRILDDRLKLNKV----VRELQHSRN---------------------------------------------------GVTVITED-------------------------------------------------------G---C-----VYEANYVILSVSIGVLQS--------------------------DLLAFNPPLPR---WKLEAIE-KCDVMVYTKIFLKFP--Y-----RFWPCG-PE---------------------------------------------------KEFFMYAHE-Q---------------------------------------------------------------------------------------------RGYYTFWQHM---------------------------------------------------------------------DNAY-P---GSNILVVTLTNGESKRVEA----QTN------EETL----REAM-AVLRDMFG-PN------------------------------IPDAIDILVPCWWNNRFQ-RGSYSN-FPIIS----------------N--GK-VFY---NIKAPV---------------------------GRIFFTGEHTSE--RFNGYVHGGYLAGIDTGKALVEEIRKENERES-E-SQ-TLLLEPLLALTGSLTKSKP-ETVSNIHKCDIPTQLYLS-----------G---KLG------IPEAIL--------------------------------------------------------------------------------------------------------------------------------------------------------------------------------------------------------------------------------------------------------------------------------------

>Pd_Nt1

------------------------------------------------------------------------------------------------------------------------------------------------------------------------------MATPRRCSVVIV-------------------GAGIS---------GLTAAKVLSENG-------VDD--------VMILEAS-DKIGGRIRKEEFGGV---------------------------TVEL-GAGWIAGVG-------------GKQSNPVWEL--A-LQ---SNLRTCFSD-YS-NA-----------------------------------------RYNIYDPSGKIFPSGI-------------AADSYKKAVD--SAIQKLRSQEG---------------------------NNNHE----S-------FAETTSTP-------------------------------KTPIELAIDFIL-----HDFEM--AE----------------VE-------PISTYV------DF----------------GER-EFLVADERGYEHLLYKM--AENFLFT----------------------SEGKITDSRLELNTV----VREVQHSRN---------------------------------------------------GVLVSTED-------------------------------------------------------G---S-----LYEANYVILSVSIGVLQS--------------------------DLISFTPPLPR---WKMEAIR-NLDVMVYTKIFLKFP--Y-----KFWPCE-PE---------------------------------------------------KEFFIYAHE-R---------------------------------------------------------------------------------------------RGYYTFWQHM---------------------------------------------------------------------ENAY-P---GSNILVVTLTNGESKRVES----QSD------QETL----REAM-QVLRNMFG-PD------------------------------IPDATDILVPRWWNNRFQ-RGSYSN-YPIYV----------------N--HQ-LVH---DIKEPV---------------------------GRIFFTGEHTSE--KFSGYVHGGYLSGIDTTNALLEEMRKDDGRKN-E-SQ-AFLLEPLLALTGSLTLTQA-ETVSSLHKCDIPRQLFLS---N-------S---KLG------LPEAIL--------------------------------------------------------------------------------------------------------------------------------------------------------------------------------------------------------------------------------------------------------------------------------------

>Pd_Sl1

------------------------------------------------------------------------------------------------------------------------------------------------------------------------------METPRRSSVIIV-------------------GAGIS---------GLTAAKVLSENG-------VDD--------VVILEAA-DKIGGRIRKEEFGGV---------------------------AAEL-GAGWIAGVG-------------GKQSNPVWEL--A-LQ---SNLRTCFSD-YS-NA-----------------------------------------RYNIYDHSGKIFPSGI-------------AADSYKKAVD--SAIQKLRSQEG---------------------------NHNEDTD--D-------AAETPSTP-------------------------------KTPIELAIDFIL-----HDFEM--AE----------------VE-------PISTYV------DF----------------GER-EFLVADERGYEHLLYKM--AENFLFT----------------------CEGKIMDSRLKLNTV----VREVQHSRN---------------------------------------------------GVLVTTED-------------------------------------------------------G---S-----LYEANYVILSVSIGVLQS--------------------------DLISFSPSLPR---WKMEAVR-NLDVMVYTKIFLKFP--N-----KFWPCE-PE---------------------------------------------------KEFFIYAHE-R---------------------------------------------------------------------------------------------RGYYTFWQHM---------------------------------------------------------------------ENAY-P---GSNMLVVTLTNGESKRVEA----QSD------QDTL----REAM-EVLRNMFG-PD------------------------------IPDATDILVPRWWNNRFQ-RGSYSN-YPIYA----------------N--HQ-LVH---DIKEPV---------------------------GRIFFTGEHTSE--KFSGYVHGGYLSGIDTSNALLEKMRRDDGRKN-E-SQ-AFLLEPLLALTGSLTLTQA-ETVSSLHKCDIPRQLFLS---N-------S---KLG------LPEAIL--------------------------------------------------------------------------------------------------------------------------------------------------------------------------------------------------------------------------------------------------------------------------------------

>Pd_St1

------------------------------------------------------------------------------------------------------------------------------------------------------------------------------MEIPRRSSVIII-------------------GAGIS---------GLTAAKVLSENG-------VDD--------VVILEAA-DKIGGRIRKEEFGGV---------------------------AAEL-GAGWIAGVG-------------GKQSNPVWEL--A-LQ---ANLRTCFSD-YS-NA-----------------------------------------RYNIYDHSGKIFPSGI-------------AADSYKKAVD--SAIQKLRSQEG---------------------------NHNENTE--D-------AAETPSTP-------------------------------KTPIELAIDFFL-----HDFEM--AE----------------VE-------PISTYV------DF----------------GER-EFLVADERGYEHLLYKM--AENFLFT----------------------SEGKIMDSRLKLNTV----VREVQHSRN---------------------------------------------------GVLVTTED-------------------------------------------------------G---P-----LYEANYVILSVSIGVLQS--------------------------DLISFSPPLPR---WKMEAVR-NLDVMIYTKIFLKFP--Y-----KFWPCE-PE---------------------------------------------------KEFFIYAHE-R---------------------------------------------------------------------------------------------RGYYTFWQHM---------------------------------------------------------------------ENAY-P---GSNMLVVTLTNGESKRVEA----QSD------QDTL----REAM-EVLRNMFG-PD------------------------------IPNATDILVPRWWNNRFQ-RGSYSN-YPIYA----------------N--HQ-LVH---DIKEPV---------------------------GRIFFTGEHTSE--KFSGYVHGGYLSGIDSSNALLEKMRRDDGWKN-E-SQ-AFLLEPLLALTGSLTLTQA-ETVSSLHKCDIPRQLFLS---N-------S---KLA--------EAIL--------------------------------------------------------------------------------------------------------------------------------------------------------------------------------------------------------------------------------------------------------------------------------------

>Pd_Vv4

------------------------------------------------------------------------------------------------------------------------------------------------------------------------------MDSITRCSVIVV-------------------GAGVS---------GISAAKVLAEKG-------VED--------LVILEAS-DRIGGRVRKEDFGGV---------------------------SVEL-GAGWVAGVG-------------GKESNPVWEL--A-RK---SGLRTCFSD-YS-NA-----------------------------------------RYNIYDRSGKLFPSGV-------------AADSYKKAVE--SAIQMIRHQEA---------------------------NHHGGGG--IGGADLSKLSEQLPDP-------------------------------KTPIELAIDFIL-----HDFEM--AE----------------VE-------PISTFL------EF----------------GER-EYLVADERGYEYILYKM--AETFLFS----------------------SEGKILDSRLKLNKV----VRELQHSRN---------------------------------------------------GIMVKTED-------------------------------------------------------G---C-----VYEADYVILSVSIGVLQS--------------------------DLITFRPPLPR---WKTEAIE-KCDVMVYTKIFLKFP--Y-----KFWPCG-PG---------------------------------------------------KEFFIYAHE-R---------------------------------------------------------------------------------------------RGYFTFWQHM---------------------------------------------------------------------ENAY-P---GSNILVVTLTNGESKRVEA----QSD------EETL----KEAM-GVLRDMFG-PD------------------------------IPNATDILVPCWWNNRFQ-RGSYSN-YPIIS----------------N--PQ-VVN---NIKAPL---------------------------GRIFFSGEHTSE--KFSGYVHGGYLAGIDTADSLLEEMRKEAERKA-E-NQ-TFMLEPLLALTGSLTLSQT-DAVSALNTFDIPRQLFLT-----------S---KLG------MPEAIL--------------------------------------------------------------------------------------------------------------------------------------------------------------------------------------------------------------------------------------------------------------------------------------

>Pd_Cc2

------------------------------------------------------------------------------------------------------------------------------------------------------------------------------MDSTSRSSVIII-------------------GAGVS---------GISAGKILAENG-------IED--------ILILEAS-DRIGGRVRNEKFGGV---------------------------SVEL-GAGWIAGVG-------------GKESNPVWEL--A-SK---SGLRTCFSD-YT-NA-----------------------------------------RYNIYDRSGKIIPSGV-------------AADSYKKAVE--SAIANLKNLEA---------------------------TN----S--NIGEVIKAATELPSSP-------------------------------KTPLELAIDFIL-----HDFEM--AE----------------VE-------PISTYV------DF----------------GER-EFLVADERGYAHLLYKM--AEEFLST----------------------SDGKILDNRLKLNKV----VRELQHSRN---------------------------------------------------GVTVKTED-------------------------------------------------------G---C-----VYEANYVILSASIGVLQS--------------------------DLISFKPPLPK---WKTEAIE-KCDVMVYTKIFLKFP--C-----KFWPCS-PE---------------------------------------------------KEFFIYAHE-R---------------------------------------------------------------------------------------------RGYYTFWQHM---------------------------------------------------------------------ENAY-P---GSNILVVTLTNGESKRVEA----QPD------EETL----KEAM-EVLQDMFG-PD------------------------------IPNATDILVPRWWNNRFQ-RGSYSN-YPIIS----------------D--NQ-LVN---SIRAPV---------------------------GGIFFTGEHTSE--RFNGYVHGGYLAGIDTGKAVVEKIRKDNERNN-SETQ-NFLLEPLLA----LTLTQT-EAMSSLHKCDIPKQLYLS-----------G---KLG------IPEAIL--------------------------------------------------------------------------------------------------------------------------------------------------------------------------------------------------------------------------------------------------------------------------------------

>Pd_Cs5

------------------------------------------------------------------------------------------------------------------------------------------------------------------------------MDSPSRSSVIVI-------------------GAGVS---------GLSAAKVLVDNG-------VDD--------FVILEAS-DRIGGRVCKENFGGV---------------------------SVEL-GAGWIVGVG-------------GKEPNPVWEL--A-LK---SSLRTCFSD-YS-NA-----------------------------------------RYNIYDRSGKIFPSGV-------------AADSYKKAVD--SAIQKLRNQGE---------------------------DA----D--DLSIV----SEPLCTP-------------------------------KTPMELAIDFIL-----HDFEM--PE----------------VE-------PISTYL------DF----------------GER-EFLVADERGYECLLYKM--AEDFLFT----------------------SEGKILDSRLKLNKV----VREIQHSRN---------------------------------------------------GVTVTTED-------------------------------------------------------G---C-----IYEANYVVLSVSIGVLQS--------------------------DLISFTPPLPR---WKTEAIE-KCDVMVYTKIFLKFP--Y-----KFWPCG-PG---------------------------------------------------KEFFIYAHE-R---------------------------------------------------------------------------------------------RGYYTFWQNM---------------------------------------------------------------------ENAY-P---GSNILVVTVTNGESKRVEA----QSD------EETM----KESM-EVLRDMFG-PD------------------------------IPDATDILVPRWWSNRFQ-RGSYSN-YPIIS----------------D--CQ-VVQ---NIKEPI---------------------------GRIYFTGEHTSE--RFNGYVHGGYLAGIDTSNALLEEMRKDKERKS-D-GQ-SFLLEPLLALTGSLSLSQT-EAVSGLHKCDLPTQLYLN-----------G---KLG------IQEAIL--------------------------------------------------------------------------------------------------------------------------------------------------------------------------------------------------------------------------------------------------------------------------------------

>Pd_Pt2

------------------------------------------------------------------------------------------------------------------------------------------------------------------------------MDSPPRSSVIII-------------------GAGIS---------GVSAGKVLAENG-------IED--------MVILEAS-DRIGGRIRKDNFGGV---------------------------SVEL-GAGWIAGVG-------------GKESNPVWEL--A-SQ---SGLRTCFSD-YS-NA-----------------------------------------RYNIYDRSGKIYPSGV-------------AADSYKKAVD--LAIENLKSLEA---------------------------N--------LVGEV----NEPPSSP-------------------------------KTPIELAIDFIL-----HDFEM--AE----------------VE-------PISTFV------DF----------------GER-EFLVADERGYEHLLYKM--AENFLLI----------------------SEGKILDNRLKLNKV----VRELQHSRN---------------------------------------------------GVVVKTED-------------------------------------------------------G---C-----IYEANYVILSVSIGVLQS--------------------------DLISFRPPLPR---WKTEAIE-KCDVMVYTKIFLNFP--Y-----KFWPCG-PG---------------------------------------------------KEFFIYAHE-R---------------------------------------------------------------------------------------------RGYYTFWQHM---------------------------------------------------------------------ENAY-P---GSNILVVTLTNGESKRVEA----QSD------KETL----EEAM-GVLRDMFG-PH------------------------------IPNATDILVPRWWNNRFQ-RGSYSN-YPIIS----------------D--NQ-DVH---DIKAPV---------------------------GRIFFTGEHTSE--RFSGYVHGGYLAGIDTSNSLVEEMRKEKERKS-E-SQ-TFLLEPLLALTGSLTLTQT-EAVPSLHKCDIPTHLYLS-----------G---KVG------LQEAIL--------------------------------------------------------------------------------------------------------------------------------------------------------------------------------------------------------------------------------------------------------------------------------------

>Pd_Rc1

------------------------------------------------------------------------------------------------------------------------------------------------------------------------------MDSPPLSSVIVI-------------------GAGIS---------GLSAAKVLAENG-------IED--------VVILEAS-DRIGGRIKKESFGGV---------------------------SVEL-GAGWIAGVG-------------GKESNPVWEL--A-NQ---SGLRTCFSD-YS-NA-----------------------------------------RYNIYDRSGKIFPSGV-------------AADSYKKAVD--SAIMKLRSQEA---------------------------N--------LVGEV----IEPPCSP-------------------------------KTPIELAIDFIL-----HDFEM--AE----------------VE-------PISTYV------DF----------------GER-EFLVADERGYEYLLYKI--AEDFLFT----------------------SEGKILDTRLKLNKV----VREIQHSRN---------------------------------------------------GVTVKTED-------------------------------------------------------G---C-----IYEANYVILSASIGVLQS--------------------------DLISFRPPLPS---WKTEAIE-KCDVMVYTKIFIKFP--Y-----KFWPCC-PE---------------------------------------------------KEFFIYAHE-R---------------------------------------------------------------------------------------------RGYYTFWQHM---------------------------------------------------------------------ENAY-P---GSNILVVTLTNGESKRVEA----QSD------EETL----EEAM-EVLRDMFG-PN------------------------------IPNATDILVPRWWNNRFQ-RGSYSN-YPIIS----------------N--NQ-VLH---DIRAPV---------------------------GRILFTGEHTSE--RFNGYVHGGYLSGIDTSKTLLEEMIQEKERKN-E-NQ-TFLLEPLLALTESLTLTQT-EAVSTLHKCDIPTQLYLS-----------G---KLS------IPEAIL--------------------------------------------------------------------------------------------------------------------------------------------------------------------------------------------------------------------------------------------------------------------------------------

>Pd_Ah1

------------------------------------------------------------------------------------------------------------------MRKINKVEAMKF------------------------------------LLFLVMGLLVSLISASSYPSVIVI-------------------GAGMS---------GISAAKTLHDNN-------IKD--------FIILEAT-NRISGRIHKTEFAGY---------------------------TVEK-GANWLHGAE-------------GPEKNPMYEI--A-EK---INLKNFYSD-FS-NV-----------------------------------------SLNTYKQNGEKYSMEE-------------VEAAIALADDNEEFGTKLAEQFS---------------------------ANTKEDDDMSLLAAQRLNKK-E--P-------------------------------KTILERMVDFYF-----NDGEQ--AEAPRVSSL-------KHIL-------PRPEFS------LY----------------GDG-EYFVADPRGFEGITHTI--AKSFLSY----------------------TNHTVTDPRLMFNQV----VTEIEYKRR---------------------------------------------------SVTVKTED-------------------------------------------------------G---N-----VYKAKYVIVSPSLGVLQS--------------------------DLITFTPELPL---WKRRAIS-EFSIGIYTKIFLKFP--Y-----KFWPTG-PG---------------------------------------------------TEFFFYVHA-R---------------------------------------------------------------------------------------------RGYYAIWQQL---------------------------------------------------------------------ENEY-P---GSNILFVTVADEESKRVEQ----QPD------EVTK----AEAM-EVLRKIFG-ED------------------------------IPEATDIMIPRWYSDRFY-RGTFTN-WPVGY----------------T--NK-KHK---NLRAPV---------------------------GRVFFTGEHTHP--ELFGYADGAYFAGITTANDILARLKGGILPWHNQDMK-LMKI------------------------------------------------------------------------------------------------------------------------------------------------------------------------------------------------------------------------------------------------------------------------------------------------------------------------------------------------------

>Pd_Rc4

------------------------------------------------------------------------------------------------------------------MKKLFY----MV------------------------------------PLILFHLFLVAAASASSSPTAVVI-------------------GAGIS---------GIAAAKTLHEAG-------IQD--------ILILEAT-PRIGGRLMKTQFSGY---------------------------TVEM-GCNWLFTG--------------GPVANPLIDM--A-KK---LKLRTFYSD-FE-NI-----------------------------------------TSNTYKQEGGLYPKKQ-------------VEEVSGVATARDDFCVKFSQKLS---------------------------AKKK-DVDVSILAAQRIYNKRP--P-------------------------------TSPLEMVIDFFY-----NDFED--AEPPKVTSL-------KHTY-------PRNEMV------DH----------------GED-EYFVADPRGVEVLVQYL--AKQFLSS-------------------------VTKDPRLKLNKV----VRDISYSDS---------------------------------------------------GVIIKTED-------------------------------------------------------G---S-----TYNSKYVIVSVSLGVLQS--------------------------DLIEFQPKLPV---WKRIAIS-DFSMTIYTKIFMKFP--Y-----KFWPTG-PG---------------------------------------------------TEFFLYSHV-R---------------------------------------------------------------------------------------------RGYYPAWQHL---------------------------------------------------------------------ENEY-P---GSNILFATVTADESRRIEQ----LSD------EAVE----AELM-EILKKLFG-DH------------------------------IPKPESILVPRWGLNKFY-KGSYSN-WPANY----------------N--QK-RKD---QLADPV---------------------------GPVYFTGEHTSN--KYIGYATGAYLAGIDTANDLIECIKNKSCKGSHHKN------------------------------------------------------------------------------------------------------------------------------------------------------------------------------------------------------------------------------------------------------------------------------------------------------------------------------------------------------------

>Pd_Cc1

------------------------------------------------------------------------------------------------------------------MKVSAVV---LA------------------------------------LALLLPFTLVIAPTSPPSNSVIIV-------------------GAGMS---------GFMAAKTLEEAG-------YKD--------FIILEAS-SRVGGRLHKGNIGGH---------------------------TIEL-GANWVNSG--------------GPKSSPSLQI--A-KK---IKLKTFYSD-YA-NL-----------------------------------------TSNIYKQDGGLYQKHV-------------VESAVRIAKTRDAFCTNLSKILS---------------------------SETTRDDDTSILGSQRLLKE-V--P-------------------------------MTPLEMAIDYFF-----NDYED--AEPPRITSL-------KTTY-------PRNQLV------DF----------------GED-SYFVADPRGFESVVHSV--AKQFLSH----------------------GHQVIRDPRLKLNKV----VRNISYSKD---------------------------------------------------KVTVKTED-------------------------------------------------------G---S-----VYQANYAIVSVSIGVLQS--------------------------DFIEFTPNLPL---WKKLAIN-NFNMAIYTKIFMKFP--Y-----KFWPTG-PG---------------------------------------------------TEFFIYAHE-R---------------------------------------------------------------------------------------------RGYFPIWQHL---------------------------------------------------------------------ENEM-P---GSNILFVTVTDEESRRVER----QSD------EKTK----AEIMNNVLRKLFG-NK------------------------------IPEPQSIFVPRWWSNRFF-NGSYSN-WPNGF----------------T--QQ-SYK---ELKEPI---------------------------GPIYFTGEHTNS--TYLGYIDGAYFAGINTANDLIKCFKQFSDGHHI---------------------------------------------------------------------------------------------------------------------------------------------------------------------------------------------------------------------------------------------------------------------------------------------------------------------------------------------------------------

>Pd_Pt1

----------------------------------------------------------------------------------------------------------------------ELL---SS------------------------------------VLVLAILFILTMTSASPSPTVIII-------------------GAGMS---------GILAAKTLHDSG-------IQD--------ILILEAN-SKIGGRIHSVQFRGH---------------------------TVEL-GANWVIGG--------------GPRSNHLYEI--A-SK---LNLKTYLSD-YG-NI-----------------------------------------SANIYKQEGGLYPKHI-------------VSAALEVAETRDQFCTSFSTRLS---------------------------APGHDRDDVSILVSQRLFKE-V--P-------------------------------TTPLDMVIDYFY-----NDYED--AEPPRVTSL-------KNTI-------PRYEFL------DF----------------GDQ-TYFLADSRGFESILIYI--AKQFLSH----------------------KHEVIRDQRLKLNKV----VREINYSKS---------------------------------------------------GVQVKTED-------------------------------------------------------G---S-----VYQAKYVIVSVSVGVLQS--------------------------DLIVFKPHLPQ---WKTQAIY-EFDMAVYTKIFLRFP--Y-----KFWPSG-PE---------------------------------------------------TEFFLYAHE-K---------------------------------------------------------------------------------------------RGYYPIWQHL---------------------------------------------------------------------ETEM-P---GSNILFVTVTDEEAKRIEQ----QQD------IKIQ----EEIM-DVLKKMFG-ND------------------------------IPEPDEILIPRWWSNRFF-KGSFSN-WPIGY----------------S--QR-RHM---QLKEPV---------------------------GRIYFSGEHTYS--RYLGYADAAYFAG-----------------------------------------------------------------------------------------------------------------------------------------------------------------------------------------------------------------------------------------------------------------------------------------------------------------------------------------------------------------------------------

>Pl_Sm2

--------------------------------------------------------------------------------------------------------------------MATS--LLIL------------------------------------LVFNAWVAIVASDGHTKTDSVLIV-------------------GAGIS---------GIMAAKTLSQNG-------IND--------FVILEAT-ERIGGRMREEAFAGG---------------------------IVEI-GANWVEGVH-------------GSKVNPIWTL--A-NK---YNLTSFYTD-FS-NQ-----------------------------------------SSNIYTKNGYVDPSTV-------------T-NETKMAEAEKEYVTNLAISKS---------------------------KNG--EQDISILTGQRLFGSVP----------------------------------QTPIEMCLEYQN-----YDFEF--AEPPRVTSL-------ENTH-------PNPTFR------DF----------------GDD-EYFVADPRGYSHIVHQL--AGDFLQT----------------------RNGKITDPRLLLNKV----VRKIKYSKD---------------------------------------------------GVKLLTED-------------------------------------------------------G---S-----TYFGKFAIVTASLGVLQS--------------------------SLIKFQPVLPD---WKVEALF-QFDMAIYTKIFLRFP--Y-----TFWPIY-PG---------------------------------------------------AQFLIYCDE-R---------------------------------------------------------------------------------------------RGYYSTWQSLVSFQHL---------------------------------------------------------------AKEF-P---GKNMIFVTVTDEESRRIEQ----LPD------KEIK----AEIM-SVLRKMFG-PN------------------------------IPEIEEMLVPRWGSMKYF-KGSYSN-WPIGV----------------S--DS-EFE---AIQAPV---------------------------ETLYFAGEHTSQ--KYSGYVHGAYLTGIEAGKDLVACIKHKKCRKFSQEKH-KDLK---NS---------T-CKEEIAKEREATRQAWTK---KMDAVLASH---KNG------M-------------------------------------------------------------------------------------------------------------------------------------------------------------------------------------------------------------------------------------------------------------------------------------------

>Pp_PBp_Ah

---------------------------------------------------------------------------------------------------------------------MAY--TSDH------------------------------------FCLVFFICTVLFSLSVGATTTIIV-------------------GAGMS---------GIMAAKTLTDNG-------VKD--------FVILEAT-NRIGGRMHKETVGGY---------------------------TIEI-GANWVEGVG-------------GKIMNPIWPL--A-KK---YKLRTFYSD-WS-NL-----------------------------------------SYNIYHQEGGILPQSL-------------VARPYALATSSSDFSSKLSESFH---------------------------KSG--EEDVSILASQRTFGHVP----------------------------------VTPLEMAIDFYF-----YDFEI--AEPPRVTSL-------KNVL-------PNPTFD------DF----------------GED-EYFVADSRGYEYIVHKI--AQEFLDS----------------------HNGDISDNRLKLNQV----VREIQYTDK---------------------------------------------------GVKVVTEN-------------------------------------------------------G---S-----AYTAENVIVSVSVGVLQT--------------------------KLIKFKPDLPL---WKLLSIY-RWDMVIYCKIFMKFP--S-----KFWPTG-PG---------------------------------------------------TEFFIYAHE-Q---------------------------------------------------------------------------------------------RGYYNFWQHL---------------------------------------------------------------------ENEY-P---GGNLLMVTVTDDEARRIEQ----QPD------HETK----IEIM-GVLRKMFG-SD------------------------------IPEMEAILIPRWGRDRFF-KGTYSN-WPIGV----------------S--TH-DFD---NIKAPVG--------------------------PIISLENIQVRN--IMV-----MYTVLILQASTLPICCWTA---------------------------------------------------------------------------------------------------------------------------------------------------------------------------------------------------------------------------------------------------------------------------------------------------------------------------------------------------------------------

>Pl_Sm1

--------------------------------------------------------------------------------MRSFSSFFLSSFVFFFFLASVPLD--RSR----------------C------------------------------------VAAARKIAAEDALDAKYSFDVIIV-------------------GAGMA---------GIMAANTLSEAG-------IDD--------FVILEAT-DRIGGRMREADFAGK---------------------------RIEL-GANWVEGVN-------------ETTTNPIWEL--A-NK---HKLRMFYSN-FD-NL-----------------------------------------SSNIYTQDGHFANKL--------------GDIYMKKLDDSSEWIESLGIKKS---------------------------QSN--SADISVLTAQRIYGKVP----------------------------------STPVEMVLDYYN-----YDYEF--AEPPRVTSL-------KNTQ-------PNPTFH------NF----------------GDS-NFLVADQRGYSYLVQKL--AEEFLDS----------------------KDGVITDPRLKLNTV----VNNIRYSKN---------------------------------------------------GVTVGTEG-------------------------------------------------------G---K-----SYKAKYVIVTVSLGVLQS--------------------------GLIKFIPPFPD---WKIEALS-EFDMAVYTKIFLKFP--Y-----KFWPSNGPL---------------------------------------------------TEFMLYADE-H---------------------------------------------------------------------------------------------RGYYPVWQHL---------------------------------------------------------------------ENEY-P---GANVMFVTVTDDESRRIEQ----QPR------NETI----EEVH-EVLKNMFG-PS------------------------------VPKPIDILVPKWFSNRFF-VGSFSN-WPIGV----------------E--SY-EFE---RIQAPLK--------------------------GALYFSGEHTHE--HYNGYVHGAYYSGIDAANRLLACKKEGKCIDEVPQAP-PHRR---GS--KKRRNVDE-AEMEAIKERDAARQAWKK---SAM--------------------------------------------------------------------------------------------------------------------------------------------------------------------------------------------------------------------------------------------------------------------------------------------------------------

>Pd_Vv1

-------------------------------------------------------------------------------------------------MGIR------------RTRL-ML--ASLI------------------------------------VIMGTLLGCPGAATAKRAPTVIIV-------------------GAGMS---------GISAAKTLSDAG-------IKR--------ILILEAT-NRIGGRMYKANFSGV---------------------------SVEL-GANWVSGVG-------------GPQVNPVWIM--A-NK---LRLKSFLSN-FL-NL-----------------------------------------SSNTYKPEGGVYEESV-------------ARKAFEVAEQVVEFGTKVSKDLA---------------------------ARK--QPDISILTSQRLKNYFP----------------------------------KTPLEMVIDYYL-----CDFES--AEPPRATSL-------LNSE-------PSSTYS------NF----------------GED-SYFVSDPRGYESVVHYV--AQQFLTT---------------------NAAGQITDPRLQLKKV----VTEISRSPR---------------------------------------------------GVAVKTED-------------------------------------------------------G---L-----VHRADYVIVSVSLGVLQN--------------------------DLIKFHPSLPQ---WKILALD-QFNMAIYTKIFLKFP--Y-----KFWPSG-NG---------------------------------------------------TEFFLYAHE-K---------------------------------------------------------------------------------------------RGYYPFWQHL---------------------------------------------------------------------EREF-P---GENVLLVTVTDDESRRLEQ----QSD------SETK----AEIM-AVLRNMFG-KQ------------------------------IPEATDILVPRWLSNRFF-KGSYSN-WPIGV----------------S--HH-QFN---QIKAPV---------------------------GKVYFTGEHTSA--AYYGYVHGAYFAGIDTAKLMTSCIKRGACSYNI---------------------------------------------------------------------------------------------------------------------------------------------------------------------------------------------------------------------------------------------------------------------------------------------------------------------------------------------------------------

>Pd_Vv2

--------------------------------------------------------------------------------------MELQIELRTGGMLIT------------KTRVLL---ASLT------------------------------------AIMAVVFSASSAIAAEEVHAVVIV-------------------GAGMS---------GISAANKLSEAG-------IEN--------ILILEAT-NRIGGRIQKMNFAGL---------------------------SVEI-GASWVEGVG-------------GPRLNPIWDM--V-NR---LKLTTFYSN-YD-NI-----------------------------------------SSNAYKQKGGLYEKSE-------------AQNAFYAAQELSEFIKNVSKYLK---------------------------AHR--QDDISILASQRLKNQVP----------------------------------STPLDMAIDYIA-----YDYEF--SEPPRVTSL-------KNSI-------PLHTFS------KF----------------GED-AYFVADPKGYESVVYFV--AKQFLTT---------------------NESGEITDPRLLFNKV----VNEISYTKN---------------------------------------------------GVTVKTED-------------------------------------------------------G---S-----VYRAEYVMVSASIGVLQS--------------------------GLINFKPDLPP---WKILAIY-QFDMAVYTKIFLKFP--D-----KFWPTG-NG---------------------------------------------------TEFFFYAHE-K---------------------------------------------------------------------------------------------RGYYTIWQQL---------------------------------------------------------------------EEEY-P---GANFLLVTVTDDESRRIEQ----QPD------SDTK----AEIM-GVLRAMFG-KN------------------------------ISEATDVLVPRWWSDKFY-RGSYSN-WPIGV----------------S--RL-EYD---RIRAPV---------------------------GRVYFTGEHTSE--YFNGYVHGAYLAGIDSAKMLIRCVKHGDCCYSIPPEG-S---------------------------------------------------------------------------------------------------------------------------------------------------------------------------------------------------------------------------------------------------------------------------------------------------------------------------------------------------------

>Pd_Vv3

-------------------------------------------------------------------------------------------------MVNN------------KETSMVA--SLLA------------------------------------VMAVVLSFFSIAQAAAKVPTVIVV-------------------GAGMS---------GISAAKTLSDAG-------IKN--------ILILEAT-DRIGGRIHKTNFAGL---------------------------SVEM-GANWVEGVG-------------GSEMNPIWEM--V-NK---IKLKTFFSD-YD-NV-----------------------------------------SSNTYKQVGGLYAESV-------------AQHLLDSLDNVVEFSENLSTLLT---------------------------AKK--QEDISVLTAQRLKNRVP----------------------------------STPLEMAIDYYN-----YDYEF--AEPPRVTSL-------QNTA-------PLPTFA------NF----------------GED-LYFVGDSRGYESVVHYV--AKQFLTT---------------------NKDGQITDPRLLLNKA----VVQITYSPS---------------------------------------------------GVIIKTED-------------------------------------------------------G---S-----VYRAEYVMLSPSIGVLQS--------------------------TLIDFKPDLPP---WKILAIY-QFDMAVYTKIFLKFP--Y-----KFWPAG-NG---------------------------------------------------TEFFLYAHE-K---------------------------------------------------------------------------------------------RGYYTIWQQL---------------------------------------------------------------------EREY-P---GSNVLLVTVTDDESKRIEQ----QPD------SDTK----AEVM-GVLRAMFG-KN------------------------------IPEATDILVPRWWSNKFY-KGTFSN-WPIGV----------------S--RF-EFD---QIRAPV---------------------------GRVYFTGEHTSQ--HYNGYVHGAYLAGIDSANILIPCIKKGACTYHVQPKG-T---------------------------------------------------------------------------------------------------------------------------------------------------------------------------------------------------------------------------------------------------------------------------------------------------------------------------------------------------------

>Pm_Ma1

---------------------------------------------------------------------------------------------------------------------------------------------------------------------------------MDSASVIII-------------------GAGMS---------GISAAKTLSDAG-------VKE--------ILILEAT-NRIGGRIRNTYFADL---------------------------SVET-GANWIEGVH-------------GEKQNPIWEM--A-QQ---LGLRTFRSD-YS-NL-----------------------------------------SSNTYKQDGGRYEKAA-------------VEAAIDESEKIHSLGEEYSKTLP---------------------------PSG--RNDISISTFQRLENKIP----------------------------------STPLEMIVDYYS-----YDYEF--AEPPRVTSL-------QNTV-------PLPTFD------DF----------------GDN-VYFVADERGYEILVYNL--ASMFLET---------------------NSHGVIVDPRLQLGKV----ATEIQYSGD---------------------------------------------------GATVSTDD-------------------------------------------------------R---S-----SYRADFVLVSVSIGVLQS--------------------------DLIKFNPVLPK---WKILALY-EFDMAVYTKIFLKFP--F-----KFWPDG-DD---------------------------------------------------TEFFLYASK-R---------------------------------------------------------------------------------------------RGYYPLWQHF---------------------------------------------------------------------EKQY-P---GANILMVTVTDEESRRIEQ----QDE------STTK----KEAM-EVLRSMFG-KD------------------------------IPEATDILVPRWWSNKFF-RGSFSN-WPLGV----------------N--RH-EFD---SIKAPI---------------------------KRLYFTGEHTSE--HYNGYVHGAYLAGLHHLHDQSHHHS-----------------------------------------------------------------------------------------------------------------------------------------------------------------------------------------------------------------------------------------------------------------------------------------------------------------------------------------------------------------------

>Pm_Si4

------------------------------------------------------------------------------------------------------------------MKPSVV--IALA------------------------------------AV--LVAAQYASLADAYGPRVIIV-------------------GAGMSAERNIALRAGISAGKRLWESG-------VRE--------LLFLEAT-ERVGGRMHKHNFGGL---------------------------NVEI-GANWVEGIG-------------GERVNPILPI--V-NDT--LKLRNFYSD-FD-SV-----------------------------------------VGNFYRENGGLYDKDY-------------VQKRMDRADEVEDLGANLTKMMD---------------------------PSG--RDDISILAMQRLFNHQPNGP-------------------------------STPVDMALDYYK-----FDYEF--AEPPRVTSL-------QNTE-------PTPTNA------DF----------------GED-SNFVADQRGFESIIHYI--GSSYLST---------------------NANGKISDRRVLLNKV----VRQIAYNNR---------------------------------------------------GVVVKTED-------------------------------------------------------G---S-----SYAADFVVVSTSLGVLQS--------------------------DLIQFKPQLPF---WKIFSIY-RFDMAVYTKIFLKFP--R-----RFWPVG-DG---------------------------------------------------KQFFVYASR-R---------------------------------------------------------------------------------------------RGYYGMWQSF---------------------------------------------------------------------EREY-P---GVPVLLVTVTDDESRRIEQ----QPD------DVTK----AEAV-AVLRKMFPDVD------------------------------VPNATDIYVPRWWSNRFF-KGSYSN-WPIGV----------------N--RY-EYD---QLRAPV---------------------------GRVYFTGEHTSE--RYNGYVHGAYLAGIDSADILINRIFKNE-EYKVRGKY-EDQA---AE--VNGQVAEA-K-------------------------------------------------------------------------------------------------------------------------------------------------------------------------------------------------------------------------------------------------------------------------------------------------------------------------------------

>Pm_Si2

------------------------------------------------------------------------------------------------------------------MKPSVV--IALA------------------------------------AV--LVAAQYASLADAYGPRVIIV-------------------GAGMS---------GISAGKRLWESG-------VRE--------LLFLEAT-ERVGGRMHKHNFGGL---------------------------NVEI-GANWVEGIG-------------GERVNPILPI--V-NDT--LKLRNFYSD-FD-SV-----------------------------------------VGNFYRENGGLYDKDY-------------VQKRMDRADEVEDLGANLTKMMD---------------------------PSG--RDDISILAMQRLFNHQPNGP-------------------------------STPVDMALDYYK-----FDYEF--AEPPRVTSL-------QNTE-------PTPTNA------DF----------------GED-SNFVADQRGFESIIHYI--GSSYLST---------------------NANGKISDRRVLLNKV----VRQIAYNNR---------------------------------------------------GVVVKTED-------------------------------------------------------G---S-----SYAADFVVVSTSLGVLQS--------------------------DLIQFKPQLPF---WKIFSIY-RFDMAVYTKIFLKFP--R-----RFWPVG-DG---------------------------------------------------KQFFVYASR-R---------------------------------------------------------------------------------------------RGYYGMWQSF---------------------------------------------------------------------EREY-P---GVPVLLVTVTDDESRRIEQ----QPD------DVTK----AEAV-AVLRKMFPDVD------------------------------VPNATDIYVPRWWSNRFF-KGSYSN-WPIGV----------------N--RY-EYD---QLRAPV---------------------------GRVYFTGEHTSE--RYNGYVHGAYLAGIDSADILINRIFKNE-EYKVRGKY-EDQA---AE--AK---------------------------------------------------------------------------------------------------------------------------------------------------------------------------------------------------------------------------------------------------------------------------------------------------------------------------------------------

>Pm_Si3

------------------------------------------------------------------------------------------------------------------MKPSVV--IALA------------------------------------AV--LVAAQYASLADAYGPRVIIV-------------------GAGMS---------GISAGKRLWESG-------VRE--------LLFLEAT-ERVGGRMHKHNFGGL---------------------------NVEI-GANWVEGIG-------------GERVNPILPI--V-NDT--LKLRNFYSD-FD-SV-----------------------------------------VGNFYRENGGLYDKDY-------------VQKRMDRADEVEDLGANLTKMMD---------------------------PSG--RDDISILAMQRLFNHQPNGP-------------------------------STPVDMALDYYK-----FDYEF--AEPPRVTSL-------QNTE-------PTPTNA------DF----------------GED-SNFVADQRGFESIIHYI--GSSYLST---------------------NANGKISDRRVLLNKV----VRQIAYNNR---------------------------------------------------GVVVKTED-------------------------------------------------------G---S-----SYAADFVVVSTSLGVLQS--------------------------DLIQFKPQLPF---WKIFSIY-RFDMAVYTKIFLKFP--R-----RFWPVG-DG---------------------------------------------------KQFFVYASR-R---------------------------------------------------------------------------------------------RGYYGMWQSF---------------------------------------------------------------------EREY-P---GVPVLLVTVTDDESRRIEQ----QPD------DVTK----AEAV-AVLRKMFPDVD------------------------------VPNATDIYVPRWWSNRFF-KGSYSN-WPIGV----------------N--RY-EYD---QLRAPV---------------------------GRVYFTGEHTSE--RYNGYVHGAYLAGIDSADILINRIFKNE-EYKVRGKY-EDQA---AE--VNGQVAER-KKF-------HRLNEW----------------------------------------------------------------------------------------------------------------------------------------------------------------------------------------------------------------------------------------------------------------------------------------------------------------------

>Pm_Bd1

-----------------------------------------------------------------------------------------------------------------MMKPSTF--SALA------------------------------------AVILLVVTQHASVAAGRGPRVIIV-------------------GAGMS---------GISAGKRLADAG-------VRD--------VLILEAT-GRVGGRMHKHNFGGI---------------------------NVEI-GANWVEGVE-------------GKKVNPIWPM--V-NAT--LNLRNFLSD-FD-SV-----------------------------------------VSNVYKEKSTCR-------------------REWDRADEVEELGGKLASQMD---------------------------PSG--RDDISILAMQRLFNHQPNGP-------------------------------TTPVDMALDYFR-----YDYEF--AEPPRATSL-------QNTE-------PLPTAA------DF----------------GED-NHFVADQRGFEAIIYHI--ARQYLSSD--------------------RKSGNIVDPRLKLNKV----VREISYNRK---------------------------------------------------GVVVRTED-------------------------------------------------------N---S-----AYSGDYVIVSTSLGVLQS--------------------------DLIQFKPQLPA---WKIIAIY-RFDMAVYTKIFLKFP--T-----KFWPVG-EG---------------------------------------------------KQFFVYASS-R---------------------------------------------------------------------------------------------RGYYGMWQSF---------------------------------------------------------------------EKEY-P---GANVLMVTVTDQESRRIEQ----QPD------NQTK----AEAV-AVLRKMFPDRH------------------------------VPDATDIYVPRWWSDRFF-KGSYSN-WPIGV----------------N--RY-EYD---QLRAPV---------------------------GRVFFTGEHTSE--HYNGYVHGAYLAGMDSADILMNSIFNKV-GFKVRPKD-DHQI---IA--EAK--------------------------------------------------------------------------------------------------------------------------------------------------------------------------------------------------------------------------------------------------------------------------------------------------------------------------------------------

>Pm_Hv2

------------------------------------------------------------------------------------------------------------------MKPSFV--TAIA------------------------------------ALLLIAAQHASIVAAGKGPRVIIV-------------------GAGMS---------GISAGKRLWDAG-------VRD--------LLILEAT-DRVGGRMHKHNFGGL---------------------------NVEI-GANWVEGLN-------------GDKTNPIWPM--V-NST--LKLRNFYSD-FD-GV-----------------------------------------VANVYKESGGLYDEEF-------------VQKRMDRADEVEELGGKFAAKLD---------------------------PSG--RDDISILAMQRLFNHQPNGP-------------------------------TTPVDMALDYYK-----YDYEF--AEPPRVTSL-------QGTE-------PTATFA------DF----------------GDD-ANFVADQRGFETIIYHI--AGQYLRS---------------------DKSGNIIDPRVKLNKV----VRQISYNDK---------------------------------------------------GVVVTTED-------------------------------------------------------N---S-----AYSADYVMVSTSLGVLQS--------------------------DLIQFKPQLPA---WKIMAIY-RFDMAVYTKIFLKFP--K-----KFWPTG-PG---------------------------------------------------KQFFVYASS-R---------------------------------------------------------------------------------------------RGYYGMWQSF---------------------------------------------------------------------EKEY-P---GANVLLVTVTDVESRRIEQ----QPD------NVTM----AEAV-GVLRNMFPDRD------------------------------VPDATDIYVLRWWSNRFF-KGSYSN-WPIGV----------------N--RY-EYD---QLRAPVG--------------------------GRVYFTGEHTSE--HYNGYVHGAYLAGIHSADILMNKASNNV-DFKVRPKY-DDEL---KA--EAK--------------------------------------------------------------------------------------------------------------------------------------------------------------------------------------------------------------------------------------------------------------------------------------------------------------------------------------------

>Pd_OBp_As

-----------------------------------------------------------------------------------------------------------------------------------------------------------------------------------------------------------------------------------------------------------------------------------------------------------------------------------------------------------------------------------------------------------------------------------------------------------------------------------------------------------------MDSLPNGP-------------------------------SSPVDMVVDYYL-----YDYEY--AEPPRVTSL-------QNAV-------PQRTFS------DF----------------GDD-VYFVADKRGYESVVHYL--AGQYLNT---------------------DDSGNVADPRLQLNKV----VREISYSSS---------------------------------------------------GVTVKTED-------------------------------------------------------G---S-----VYQADYRHGLCQLGSPAERSYTVQATAACKFRVLCPARFSRIASDRCVLHVFDQK---WKILAIY-EFDMAVYTKIFVKFP--K-----RFWPEG-EG---------------------------------------------------REFFLYAST-R---------------------------------------------------------------------------------------------RGYYGIWQEF---------------------------------------------------------------------EKQY-P---DSNVLLVTVTDKESRRIEQ----QSD------NQTK----AEIM-EVLRNMFPDQD------------------------------VPDATDILVPRWWSNRFY-KGTFSN-WPIGV----------------N--RY-EYD---QLRAPI---------------------------ERVYFTGEHTSE--YYNGYVHGGYLAGIDSAEILIDCAQNQMCKYHVQGKY-D---------------------------------------------------------------------------------------------------------------------------------------------------------------------------------------------------------------------------------------------------------------------------------------------------------------------------------------------------------

>Pm_Hv3

------------------------------------------------------------------------------------------------------------------MLEMKA--CTAI------------------------------------ALVLVVAAQCAALATAAGPRVIIV-------------------GAGMS---------GISAGKRLSEAG-------ITD--------LVILEAT-DRIGGRIHKTKFAGV---------------------------NVEM-GANWVEGVN-------------GDEMNPIWTM--A-NGTGGLNLRTFRSD-FD-HL-----------------------------------------ASNTYKQDGGLYDEKV-------------VENIIERMDEVEESGSKLSGTLH---------------------------HSG--QQDMSVMAMQRLNDHMPSGP-------------------------------ARPVDMVIDYYQ-----HDFEF--AEPPRVTSL-------QNTQ-------PLPTFS------DF----------------GDD-VYFVADQRGYESVVYHV--AGQYLKTD--------------------RKSGAIVDQRLKLNTV----AREITYFPS---------------------------------------------------GVAVRTED-------------------------------------------------------N---K-----VYRADYVVVSASLGVLQT--------------------------DLIRFKPQLPS---WKIVSIY-QFDMAVYTKIFLRFP--K-----RFWPEG-PG---------------------------------------------------KEFFLYASG-R---------------------------------------------------------------------------------------------RGYFPVWQQF---------------------------------------------------------------------ETQY-P---GSNVLLVTVTDDESRRIEQ----QSD------NQTM----AEAV-AVLRKMFPGKD------------------------------VPDATEILVPRWWSNRFF-KGSFSN-WPIGV----------------N--RY-EYD---LIRAPV---------------------------GRVYFTGEHTSE--KYNGYVHGAYLAGIDSADILINCAKNKMCKYDVKGKH-D---------------------------------------------------------------------------------------------------------------------------------------------------------------------------------------------------------------------------------------------------------------------------------------------------------------------------------------------------------

>Pm_Os6

----------MEENKVSMLLLDGWWHMVLMNV------------------------------MLIN-----------VFLDMGRYNFFGSMEPHRFVWLKNEWI--EKRN-----------------------------------------------------VLKVHRYRLFMTLKLNLRSEIRK-------------------GKHLT---------CISAGKRIWEAG-------IAD--------VLILEAT-DRIGGRMHKQSFAGV---------------------------NVEI-GANWVEGVN-------------GEKKNPIWPI--V-NST--LKLRSFRSD-FD-SL-----------------------------------------AQNVYKD-GGLCDEAY-------------VQKRMDRADEVDKSGENLSATLH---------------------------PSG--RDDMSILSMQRLNDHLPNGP-------------------------------SSPVDMAVDYFT-----YDYEF--AEPPRVTSL-------QNTV-------PLPTFT------DF----------------GDD-TYFVADQRGYESVVHHL--AGQYLNA---------------------DKSGNIADARLKLNKV----VREISYSST---------------------------------------------------GVTVKTED-------------------------------------------------------N---S-----TYQADYVMVSASLGVLQS--------------------------DLIQFKPQLPS---WKILAIY-QFDMAVYTKIFVKFP--K-----KFWPEG-AG---------------------------------------------------REFFLYAST-R---------------------------------------------------------------------------------------------RGYYGVWQEF---------------------------------------------------------------------EKQY-P---DANVLLVTVTDEESRRIEQ----QPD------SQTK----AEIM-EVVRCMFPDED------------------------------VPDATDILVPRWWSDRFF-RGSFSN-WPIGV----------------S--RY-EYD---QLRAPV---------------------------GRVYFTGEHTSE--RYNGYVHGAYLAGIDSAEILINCAQKKMCKYNVGGKH-G---------------------------------------------------------------------------------------------------------------------------------------------------------------------------------------------------------------------------------------------------------------------------------------------------------------------------------------------------------

>Pm_Os7

---------------------------------------------------------------------------------MTKPTTMAIFL---SIVLLSMAQ--LPSL-----------------------------------------------------VAGTGRPRV-----------III-------------------GAGIS---------GISAGKRLSEAG-------ITD--------ILILEAT-DHIGGRMHKQRFAGV---------------------------NVEI-GANWVEGVN-------------GEKMNPIWPI--V-NST--LKLRNFLSD-FD-SL-----------------------------------------AQNVYKD-GGLCDAAY-------------VQKRIDLADEADKSGENLSATLH---------------------------PSG--RDDMSILSMQRLNNHLPNGP-------------------------------SSPVDMVVDYFT-----YDYEF--AEPPRVTSL-------RNTV-------PLPTFT------DF----------------GDD-NYFVADQRGYEAVVYYL--AGQYLEA---------------------DKSGNIVDARLQLNKV----VREISYSST---------------------------------------------------GVTVKTED-------------------------------------------------------N---S-----TYQADYVMVSASLGVLQS--------------------------DLIQFKPQLPS---WKILAIY-QFDMAVYTKIFVKFP--K-----KFWPEG-AG---------------------------------------------------REFFLYAST-R---------------------------------------------------------------------------------------------RGYYGVWQEF---------------------------------------------------------------------EKQY-P---DANVLLVTVTDEESRRIEQ----QPD------SQTK----AEIM-EVVRSMFPDED------------------------------VPDATDILVPRWWSDRFF-QGSFSN-WPIGV----------------S--RY-EHD---QLRAPV---------------------------GRVYFTGEHTSE--RYNGYVHGAYLAGIYA--------------------------------------------------------------------------------------------------------------------------------------------------------------------------------------------------------------------------------------------------------------------------------------------------------------------------------------------------------------------------------

>Pm_Hv1

-------------------------------------------------------------------------------------------------------------------MKPTT--ATAA------------------------------------LVLALTLAHHASIAAAAGPRVIIV-------------------GAGMS---------GISAGKRLSEAG-------ITD--------LVILEAT-DHVGGRMHKQSFGGI---------------------------NVEV-GANWVEGVNG------------AGRMNPIWPL--V-NST--LKLKNFRSD-FD-GL-----------------------------------------ADNVYKENGGVYERAY-------------VQKRLDRWGEVEEGGEKLSAKLR---------------------------PSG--QDDMSILAMQRLNDHLPNGP-------------------------------TSPVDMVLDYFK-----HDYEF--AEPPRVTSL-------QNVV-------PLATFT------DF----------------GDD-VYFVADQRGYEAVVYYL--AGQYLKA---------------------DKSGNIVDPRLQLNKV----VTEISHSGG---------------------------------------------------GVTVRTED-------------------------------------------------------A---K-----VYKADYVMVSTSVGVLQS--------------------------DLIQFKPRLPT---WKVLSIY-QFDMAVYTKIFVKFP--R-----KFWPQG-KG---------------------------------------------------REFFLYASS-R---------------------------------------------------------------------------------------------RGYYGVWQEF---------------------------------------------------------------------EAQY-P---DANVLLVTVTDDESRRIEQ----QSD------NQTK----AEIV-EVLRSMFPGED------------------------------VPDATDILVPRWWSDRFY-RGTFSN-WPIGV----------------N--RY-EYD---QLRAPV---------------------------GRVYFTGEHTSE--HYNGYVHGAYLSGIDSADILIKCAQKRMCKYHSPGKF-D---------------------------------------------------------------------------------------------------------------------------------------------------------------------------------------------------------------------------------------------------------------------------------------------------------------------------------------------------------

>Pm_Si1

------------------------------------------------------------------------------------------------------------------MIPSTA--TIAL------------------------------------VLAVLSLAQYAFLATAAGPRVIIV-------------------GAGMS---------GISAGKRLSEAG-------ITD--------LLILEAT-DHVGGRMHKQNFAGI---------------------------NVEV-GANWVEGVN-------------GGKMNPIWPI--V-NST--LKLRNFRSD-FD-YL-----------------------------------------AQNVYKENGGLYDQDY-------------VQKRLDQADKAEESGKKLSGTLP---------------------------ASG--SNDMSILAMQRLYDHQPNGP-------------------------------EMPVDMALDYYK-----FDYEL--AEPPRVSSM-------QNCV-------PLPTFS------DF----------------GDD-TYFVADQRGYESVVYHL--AGQYLKT---------------------DKSGKIVDPRLKLNKV----VREISYSPN---------------------------------------------------GVTVKTED-------------------------------------------------------N---S-----VYQADYVMVSASLGVLQS--------------------------DLIQFKPQLPA---WKVVAIY-KFDMAVYTKIFVKFP--K-----KFWPEG-KG---------------------------------------------------REFFLYASS-R---------------------------------------------------------------------------------------------RGYYVVWQEF---------------------------------------------------------------------EKQY-P---DANVLLVTVTDEESRRIEQ----QSD------NQTK----AEIM-EVLRKMFPGKD------------------------------VPDATDILVPRWWSDRFY-KGTFSN-WPIGV----------------S--RY-EYD---QLRAPV---------------------------GRVYFTGEHTSE--HYNGYVHGAYLAGIDSAEILINCAQKKMCKYHVQGKY-D---------------------------------------------------------------------------------------------------------------------------------------------------------------------------------------------------------------------------------------------------------------------------------------------------------------------------------------------------------

>Pm_Sb1

-------------------MLREYYTRTNTHT------------------------------STYTSHYIYVWVTLCAYIVRSASIYRGQCIHFKLLYRERSTS--VREMSSSTSSALVV--VVAA------------------------------------VLLALTLAQHGSLAATVGPRVIVV-------------------GAGMS---------GISAAKRLSDAG-------ITD--------LLILEAT-DHIGGRMHKKNFAGI---------------------------NVEV-GANWVEGVNS-----------NRGKMNPIWPI--V-NST--LKLRNFRSD-FD-YL-----------------------------------------AQNVYKEDGGLYDEDY-------------VQKRIDRADSVEELGEKLSGTLH---------------------------ASG--RDDMSILAMQRLYDHQPNGP-------------------------------ATPVDMVVDYYK-----YDYEF--AEPPRVTSL-------QNVV-------PLPTFS------DF----------------GDD-VYFVADQRGYEAVVYYL--AGQFLKT---------------------DRSGKIVDPRLQLNKV----VREINYSPG---------------------------------------------------GVTVKTED-------------------------------------------------------N---S-----VYRADYVMVSASLGVLQS--------------------------ALIQFKPQLPA---WKVTAIY-QFDMAVYTKIFLKFP--K-----KFWPEG-KG---------------------------------------------------REFFLYASS-R---------------------------------------------------------------------------------------------RGYYGVWQEF---------------------------------------------------------------------EKQY-P---GANVLLVTVTDEESRRIEQ----QSD------NQTK----AEIM-QVLRKMFPGKD------------------------------VPDATDILVPRWWSDRFY-KGTFSN-WPIGV----------------N--RY-EYD---QLRAPV---------------------------GRVYFTGEHTSE--HYNGYVHGAYLSGIDSAEILINCAQKKMCKYHVQGKY-D---------------------------------------------------------------------------------------------------------------------------------------------------------------------------------------------------------------------------------------------------------------------------------------------------------------------------------------------------------

>Pm_Zm1

--------------------------------------------------------------------------------------------------------------MSSSPSFGLL--AVAA------------------------------------LLLALSLAQHGSLAATVGPRVIVV-------------------GAGMS---------GISAAKRLSEAG-------ITD--------LLILEAT-DHIGGRMHKTNFAGI---------------------------NVEL-GANWVEGVN-------------GGKMNPIWPI--V-NST--LKLRNFRSD-FD-YL-----------------------------------------AQNVYKEDGGVYDEDY-------------VQKRIELADSVEEMGEKLSATLH---------------------------ASG--RDDMSILAMQRLNEHQPNGP-------------------------------ATPVDMVVDYYK-----FDYEF--AEPPRVTSL-------QNTV-------PLATFS------DF----------------GDD-VYFVADQRGYEAVVYYL--AGQYLKTD--------------------DKSGKIVDPRLQLNKV----VREIKYSPG---------------------------------------------------GVTVKTED-------------------------------------------------------N---S-----VYSADYVMVSASLGVLQS--------------------------DLIQFKPKLPT---WKVRAIY-QFDMAVYTKIFLKFP--R-----KFWPEG-KG---------------------------------------------------REFFLYASS-R---------------------------------------------------------------------------------------------RGYYGVWQEF---------------------------------------------------------------------EKQY-P---DANVLLVTVTDEESRRIEQ----QSD------EQTK----AEIM-QVLRKMFPGKD------------------------------VPDATDILVPRWWSDRFY-KGTFSN-WPVGV----------------N--RY-EYD---QLRAPV---------------------------GRVYFTGEHTSE--HYNGYVHGAYLSGIDSAEILINCAQKKMCKYHVQGKY-D---------------------------------------------------------------------------------------------------------------------------------------------------------------------------------------------------------------------------------------------------------------------------------------------------------------------------------------------------------

>Fz_Mc1

------------------------------------------------------------------------------------------------------------------MRK--------SI----------------------------------VLSTLLAFGCYLTEAKSYDTKVVIL-------------------GGGVS---------GISAALNLTANG-------IDD--------IIMVEAR-DTLGGKAQDVPFADV---------------------------NVEL-GCNWVQGLGT----------------NPINQL--A-LK---YKLRTAVTD-GD-DV-------------------------------------------VFYGEKGKINATER-------------YNEMNKYYEIM---SEDAIKRVK---------------------------K---DQVDLAGNVGLDIAGWYA-K---------------------------------DAIDEAIEYYV-----WDWEM--GESPELSST-------IFTVVN-----DQWTYGET-SFGPG----------------SDG-DSFVIDPRGFKHIFQEE--AKKVFV---------------------------PNDKRLLLDTT----VTKIEYNDK---------------------------------------------------GVTVHTKN-------------------------------------------------------G---D-----TIHAEYAITTFSVGVLQH--------------------------KDIEWSPKLPA---WKLEGIY-GFHMATYTKIFMNFP--T-----QFWDD-------------------------------------------------------NQFTVWVDPDQ---------------------------------------------------------------------------------------------RGYYNAWQNL----NA---------------------------------------------------------------EGFL-PKNSSSNIFFVTATQDLSYRIEA----MTD------EEVK----DEIM-VVLRRMYGN-D------------------------------IPEPTDFMFPRWHSNPLF-RGSYSN-WPIGE----------------M--DE-HHV---NMKAPLN--------------------------NRVFFAGEATSA--EYYGFLQGAWFSGAEAGLKVSKCVKGKCPVSEYYPEITNAKLHASAIR----------------------STLSKK--------------------------------------------------------------------------------------------------------------------------------------------------------------------------------------------------------------------------------------------------------------------------------------------------------------------

>Fz_Rd

------------------------------------------------------------------------------------------------------------------MKT--------GL----------------------------------LTGLATSFFFCLANAKVYNTKVAIL-------------------GGGVS---------GMSAALKLTEEG-------IHD--------FIMVEAR-HELGGRAQNAKFGDI---------------------------NVEL-GCNWVQGLGT----------------NPVNEL--A-KK---YKLHTVPTD-GD-DV-------------------------------------------LFYDEHGKVNGTDT-------------YKKFNDYYDEM---SDNAMKRIK---------------------------N---NQADLSGRTALNLVGWEA-Q---------------------------------TPLEEAIEYYV-----WDWEM--GENPEVSST-------MYAVLN-----DNWTY--T-GFGPG----------------SDG-DNMVIDNRGFKYIFVQE--SKRAFR---------------------------HKNSRLLLNSL----VTKVDYSEE---------------------------------------------------GVRVHLKN-------------------------------------------------------G---D-----MIHAEYAISTFSVGVMKH--------------------------KDVQWSPPLPE---WKMEGIY-AFDMATYTKIFMNFP--R-----KFWDD-------------------------------------------------------SQFVVWADPDR---------------------------------------------------------------------------------------------RGYFNTWQNL----NA---------------------------------------------------------------KGYL-PQNTTTNIFFVTVTQDMSFQVEK----MTD------DEVK----EAAM-DVLRQMYGD-D------------------------------IPEPDHFLFPRWHSDPLF-RGSYSN-WPIGE----------------L--DQ-HHQ---NMKAPLH--------------------------NRLFFAGEALSA--RYYGFLQGAWFTGIDAASDIVQCIRGSCSEAEYFPEITLAQIRPDYIT----------------------KRNFAF--------------------------------------------------------------------------------------------------------------------------------------------------------------------------------------------------------------------------------------------------------------------------------------------------------------------

>Fb_Hi

------------------------------------------------------------------------------------------------------------------MRL-------L---------P-------------------LLGFAFSPFAIGLQVPLTVDRTSEHHAQVLIL-------------------GGGMT---------GIIAARTLHQQG-------IHD--------FKIVEAR-DEIGGRMKSTLFGSP-----------------------SMSYRVEV-GANWIHGTQS-----------GDGPANPIFEL--A-RK---YNISLQANH-YHSSM-------------------------------------------TTFDYTGQVDYLDV-------------FNASVDAFTRL---CIAGGN-----------------------------------LPDATSRVGYSTIGTTP-L---------------------------------THQELAAEYYQ-----FDWEY--AQTPEMTSW-------AAAAWA-----ANLTFNI--DQGGF----------------SSE-NLLSIDQRGFASILQAE--AAEFLT-----------------------------DNQILLSTI----VKTIQYSDD---------------------------------------------------GVSVVLED-------------------------------------------------------G---S-----VLSADHVLVTFSLGVLQN--------------------------DDVIFEPTLPK---WKTEAIH-GMSMGTYTKVYLQFP--E-----KFWFD-------------------------------------------------------TEFALFADK-K---------------------------------------------------------------------------------------------RGWYPVWQDL----DT---------------------------------------------------------------VEFF-P---GSGVVFVTVTGEFSKRIEA----LSD------DQVE----GEVL-SVLQLMYPEMT------------------------------IPEPEAFYFPRWFSDPLY-RGSYSN-WPGNL----------------T--IE-DHE---NIKASIG--------------------------NRLWFAGEATSR--KYFGYLHGAYYEGQETAGLIARCIKSRSCHSPSHSVRRNPWD------------------------------------------------------------------------------------------------------------------------------------------------------------------------------------------------------------------------------------------------------------------------------------------------------------------------------------------------------

>Fa_An1

------------------------------------------------------------------------------------------------------------------MRLA----------------P-------------------AAA---VALPVLAAHAAPLAEPVCENTDVVIL-------------------GAGVA---------GLTAAQTLQDNG-------VNN--------FLVLEAR-GETGGRLYSHEFAGH---------------------------TVEL-GANWVHGPGK-----------ADGNINPMWTM--V-QK---ANLNTVETN-NE-EH-------------------------------------------VLYPADNVKNIAAA-------------LEAAGNATDKV---FVDAINLLQ---------------------------N---NLEDRTYRAGQRLYGWDP-RK-------------------------------TDPAEQLADWWY-----WDWGA--ASPPEMH--------------------------------------------------SEE-DRFVCDEPGFVSALRNT--VSSVLD-------------------------------RVRVNNK----VTSIKHDLS---------------------------------------------------GVTVTSNN-----------------------------------------------------------G-----CVNAKYAIVTFSLGVLQK--------------------------GDVKFDPPLPD---WKAQGIA-GFEMATYTKIFLKFP--T-----SFWDK-------------------------------------------------------EKFILWADPHV---------------------------------------------------------------------------------------------RGNYPVFQPL----DL---------------------------------------------------------------DGLY-E---GSNILVATVTGERAYRVES----QDP------EVTK----QEIY-DILRKMYFDRD------------------------------VTYPEDIYFANWSKWDWA-YGSYSY-WPAST----------------S--LQ-EHQ---NLRANV---------------------------DSVFFAGEATSQ--EFFGYLHGAYYEGKHVAEFLAPCIKVNQA---------GCTEKKYDVLTGVT-----------------PYDLYNP---------------DNG------WYIKGNGVPGN---------------------------------------------------------------------------------------------------------------------------------------------------------------------------------------------------------------------------------------------------------------------------------

>Fa_Aro

------------------------------------------------------------------------------------------------------------------MKG--KL---VFLA------T-------------------CLG--FSAIHGRI----IPEENACRKTKVAIL-------------------GAGVT---------GITAAQTLANQS-------MTD--------FLIIEYQ-DRIGGRLHEVNFGRK---------------------KDGSPYVVEA-GANWVEGLGG----------S-GKPENPIYTL--A-KK---YDIRALKTD-YE-NK-------------------------------------------TTYDKTGKKDFSSV-------------IANAAAAMQKV---VVQAGSLLK---------------------------E---NVQDKTLRAALRFVDWNP-AP-------------------------------NNAHAQFADWFS-----SDFES--SFTPEENSA-------IFSSVA-----DNATFS------HF----------------SDD-NLFVYDQRGYSTIIRGE--AATFLR---------------------------PNDPRLLLNTV----VTVVNYTHD---------------------------------------------------GVTVLTND-------------------------------------------------------G---A-----CIEADYAVSTFSLGVLQR--------------------------DAVQFYPPFPS---WKKSAIA-SFEIGTYTKIFLQFD--R-----AFWPN-------------------------------------------------------SQYLMWADPHE---------------------------------------------------------------------------------------------RGYYPLFQPL----DL---------------------------------------------------------------PGVL-P---GSGILMGTVVNRQARRVES----QTN------QETQ----KEIM-KVLRTMYGN-D------------------------------IPDPIAIYYPRWNQEPWS-YGSYSN-WPPST----------------S--LQ-VHQ---NLRANV---------------------------GRLFFAGEATSQ--EFYGYLHGAYYEGRAVGEMLARCIEGPANC------TDQNGQPRYQVLTGVT-----------------PFDLYNE---------------KHG------WSVDTTS-------------------------------------------------------------------------------------------------------------------------------------------------------------------------------------------------------------------------------------------------------------------------------------

>Fa_Ag

------------------------------------------------------------------------------------------------------------------MKL--DL---LFLT------A-------------------SLG--VSTIHGK---AIQQKEPACRKTKVAIL-------------------GAGVA---------GITAAQTLANRS-------MTD--------FVIVEYQ-GRIGGRLHDVKFGKK---------------------KDGSPYTVEA-GANWVEGLGG----------TSGHPENPIYTL--A-KK---YKIQALVTD-YD-SK-------------------------------------------TTYDKTGRNDFSKI-------------IANAASAMDKV---VAHAGSLLK---------------------------N---NIQDKTVRAALRFMGWNP-AP-------------------------------NNAHAQFADWFS-----SDFES--SFSPEENSA-------IFSSVA-----DNATFA------HF----------------SDD-NLFVYDQRGYSAFIRGE--AATFLE---------------------------PNDHRLLLNTV----VKLVNYTDD---------------------------------------------------GVTVVTDN-------------------------------------------------------G---G-----CIQADYAVSTFSLGVLQR--------------------------DVVQFYPPFPS---WKKSAIS-SFEVGTYTKIFLQFD--K-----AFWPN-------------------------------------------------------SQYLMYADPHE---------------------------------------------------------------------------------------------RGYYPLFQPL----DL---------------------------------------------------------------PGAL-Q---GSGILVGTVVGKQARKVEA----QTD------QETK----TEIM-KVLRTMFGK-N------------------------------IPDATAIWYPRWNQEPWA-YGSYSN-WPPST----------------S--LQ-AHQ---NLRANV---------------------------GRLFFAGEATSQ--EFYGYLHGALYEGRAVGEMLATCIGGPKQC------TDENGQPRYPVLTGVT-----------------PFDLYNK---------------ENG------WFVDTMV-------------------------------------------------------------------------------------------------------------------------------------------------------------------------------------------------------------------------------------------------------------------------------------

>Fa_Tr

------------------------------------------------------------------------------------------------------------------MRL--NF---VFLA------P-------------------CLG--IPIIHGKPI-EQGQKEPACRKTKVAILYVSLTIFVILFAGILTEYRGAGVA---------GITTAQTLANRS-------MTD--------FIIVEYQ-DRIGGRLHNVKFGKK---------------------RDGSPYTVEA-GANWVEGLGG----------G-NRPENPIFTL--A-EK---YKLQALATD-YD-NK-------------------------------------------TTYDRTGKNDFSKI-------------IANAASAMEKV---VTHAGSMLK---------------------------N---NIQDKTVRAALRFMGWNP-AA-------------------------------NNAHAQFADWFS-----SDFES--SFTPEENSA-------VFSSVA-----DNATFK------HF----------------SDD-NLFVYDQRGYSTFIRGE--AATFLQ---------------------------PNDPRLLLNTV----VQVVNYTDN---------------------------------------------------GVTVVTND-------------------------------------------------------G---G-----CIQADYAVATFSLGVLQR--------------------------DVVQFYPPFPS---WKKSAIS-SFEIGTYTKIFLQFD--K-----AFWPN-------------------------------------------------------SQYLMYADPRE---------------------------------------------------------------------------------------------RGYYPLFQPL----DL---------------------------------------------------------------PGAL-R---GSGILVGTVVGKQARRVEA----QTN------QETQ----DEIM-KVLRMMFGE-N------------------------------IPDPTAIWYPRWNQEPWA-YGSYSN-WPPST----------------S--LQ-AHQ---NLRANV---------------------------GRLFFAGEATSQ--EFYGYLHGALFEGRAVGQMLATCINDPVRC------TDKYGQPRYPILTGVT-----------------PYDLYNE---------------KNG------WFVSTIA-------------------------------------------------------------------------------------------------------------------------------------------------------------------------------------------------------------------------------------------------------------------------------------

>Fa_Te

----------------------------------------------------------------------------------------------------------------------------------------------------------------------------------------------------------------------------------------------MTD--------FIIVEYQ-DRIGGRLHNVKFGKK---------------------KDGSPYTVEA-GANWVEGLGG----------G-DQPENPIFTL--A-KK---YKLQALKTD-YD-NK-------------------------------------------TTYDKTGKYDFSKI-------------IENAQSAMEKV---VTHAGSLLK---------------------------N---NIQDKTVRAALRFMGWNP-AA-------------------------------NNAHAQFADWFG-----SDFES--SFTPEENSA-------VFSSVA-----DNATFK------HF----------------SDD-NLFVYDQRGYSTFIRGE--AATFLQ---------------------------PNDPRLLLNTV----VQVVNYTDN---------------------------------------------------GVTVVTND-------------------------------------------------------G---G-----CIQADYAVATFSLGVLQR--------------------------DVVQFYPPFPS---WKKSAIS-SFEIGTYTKIFLQFD--K-----AFWPN-------------------------------------------------------SQYLMYADPHE---------------------------------------------------------------------------------------------RGYYPLFQPL----DL---------------------------------------------------------------PGAL-Q---GSGILVGTVVGKQARRVEA----QTN------EETQ----EEIM-KVLRTMFGE-S------------------------------IPDPTAIWYPRWNQEPWA-YGSYSN-WPPST----------------S--LQ-AHQ---NLRANV---------------------------GRLFFAGEATSQ--EFYGYLHGALSEGRAVGQMLATCIGDPVQC------TDKNGQPRYPVLTGVT-----------------PYDLYNE---------------KNG------WFVTTVA-------------------------------------------------------------------------------------------------------------------------------------------------------------------------------------------------------------------------------------------------------------------------------------

>Fa_Tt

------------------------------------------------------------------------------------------------------------------MRL--NF---VFLA------A-------------------CLG--MPIIHGKPIVDYGQKEPACRKTKVAIL-------------------GAGVA---------GITTAQTLANQS-------MTD--------FIIVEYQ-DRIGGRLHNVKFGKK---------------------KDGSPYTVEA-GANWVEGLGG----------G-DQPENPIFTL--A-KK---YKLQALKTD-YD-NK-------------------------------------------TTYDKTGKYDFSKI-------------IENAQSAMEKV---VTHAGSLLK---------------------------N---NIQDKTVRAALRFMGWNP-AA-------------------------------NNAHAQFADWFG-----SDFES--SFTPEENSA-------VFSSVA-----DNATFK------HF----------------SDD-NLFVYDQRGYSTFIRGE--AATFLQ---------------------------PNDPRLLLNTV----VQVVNYTDN---------------------------------------------------GVTVVTND-------------------------------------------------------G---G-----CIQADYAVATFSLGVLQR--------------------------DVVQFYPPFPS---WKKSAIS-SFEIGTYTKIFLQFD--K-----AFWPN-------------------------------------------------------SQYLMYADPHE---------------------------------------------------------------------------------------------RGYYPLFQPL----DL---------------------------------------------------------------PGAL-Q---GSGILVGTVVGKQARRVEA----QTN------EETQ----EEIM-KVLRTMFGE-S------------------------------IPDPTAIWYPRWNQEPWA-YGSYSN-WPPST----------------S--LQ-AHQ---NLRANV---------------------------GRLFFAGEATSQ--EFYGYLHGALSEGRAVGQMLATCIGDPVQC------TDKNGQPRYPVLTGVT-----------------PYDLYNE---------------KNG------WFVTTVA-------------------------------------------------------------------------------------------------------------------------------------------------------------------------------------------------------------------------------------------------------------------------------------

>Fa_Vd

------------------------------------------------------------------------------------------------------------------MAS--KLLAFLALA------A-------------------ELR---PTLATVLPPRANAKPQACRKTKVAVL-------------------GAGIS---------GITAAQALSGAG-------VDD--------FLILEHN-DYIGGRVHHTTFGAK---------------------PDGSPYTVEL-GANWIEGVGG-----------TGPVKNPILEA--T-DK---AKIKSVFSN-YS-AI-------------------------------------------VSYDHTGANDYLHL-------------LDEYDGNFTLA---TQDAGSILE---------------------------N---DLQDSSMRAGLSVAGWKP-GR--------------------------------DMRAQAAEWWS-----WDFGV--SWPPDESGF-------QFGITG-----DNETFN------RF----------------GDE-RYLATEARGLNAFVREA--ALIFLDG--------------------------LEDPRLLLNTT----VEAVEHSTK---------------------------------------------------GIVVRDRD-------------------------------------------------------G---G-----CVEAEYAICTFSVGVLQN--------------------------DVVEFQPRLPV---WKREAIE-QFQMGTYTKIFLQFN--E-----SFWPQD------------------------------------------------------AQFLLYADEDE---------------------------------------------------------------------------------------------RGWYPVFQNL----GA---------------------------------------------------------------PGFL-E---GSNILFGTVVGHQAFRAEQ----QTD------EETK----GQIL-TVLRKMFPDAT------------------------------VPEPTAFMYPRWGQEEWA-FGSYSN-WPVGM----------------T--LT-KHQ---NLRANV---------------------------GRLWFAGEANSA--KYYGFMHGAYYEGKDAGERVAAMVRGEPIINE--DTAPDGQLKRYEKLYGPI-----------------NMNEYNE---------------GNG------WPDDE---------------------------------------------------------------------------------------------------------------------------------------------------------------------------------------------------------------------------------------------------------------------------------------

>Fa_Fo1

------------------------------------------------------------------------------------------------------------------MKQ--SPAQLLTVL------T-------------------AFF-----TAADAVSLPPRDKGTCRKTKVAIL-------------------GAGVA---------GIAAAQNLTQAK-------ITD--------FLIVEHN-DYIGGRLRSQQFGRN--------------------TKTGKPYTIEL-GANWVEGIGS-----------LETHENPIWRL--A-QK---HGLKTTYAD-YD-AL-------------------------------------------KTFDHKGAKNWTDK-------------IAELDAAFENA---SGDSGHILL---------------------------D---NLQDLSARAGLRTGGWRP-DK-------------------------------NDMYAQAADWWG-----WDFEA--AWTPDESGL-------VFGVAG-----DNATFG------YF----------------SDV-SNLVIDQRGYNYFLKQE--AKTFLKE---------------------------NDLRLLLKTT----VEGIEYNKK---------------------------------------------------GVKVTTKD-------------------------------------------------------G---G-----CIEANYAICTFSLGVLQK--------------------------DVVEFKPKLPH---WKQSAID-QFAMGTYTKIFMQFN--E-----SFWDTD------------------------------------------------------AQYQLYADPIE---------------------------------------------------------------------------------------------RGRYPLFQPL----NG---------------------------------------------------------------KGFL-E---GSNIIFATVTGEQAYQVER----QTD------EETE----AQVV-EVLQSMYPDKK------------------------------VHKPTAFTYPRWSTEPWA-YGSYSN-WPVGM----------------T--LE-KHQ---NIRANL---------------------------ERLWFAGEANSA--EFFGFVHGGYTEGREIGHRIGRIINGEAG-------DDEFDMERYEVLHGTT-----------------HKDEYND---------------ENG------WLFPYDVDGDN---------------------------------------------------------------------------------------------------------------------------------------------------------------------------------------------------------------------------------------------------------------------------------

>Fa_Fo3

------------------------------------------------------------------------------------------------------------------MRH--SITQLTAVF------T-------------------ALS-----TTAHSAVLQRDKQGSCTKTKVAIL-------------------GAGVA---------GIAAAQNLTKAG-------IDQ--------FIIVEHN-DYIGGRMRKQSFGKN---------------------ADGQPYTIEF-GANWVEGIGS-----------EATHENPIWQL--A-KK---YDLKSHESD-YD-NY-------------------------------------------LTFDHKGQTNWSST-------------IKSLEKIYSKA---EAEAGRLLL---------------------------G---NLQDTSVRAAIRSAGWRP-DK-------------------------------DDMHAQAADWWK-----WDFES--AWTPDESGL-------IFGVAG-----GNATFG------YF----------------SDV-SNLVVDQRGFSTIIQEE--AKTFLKK---------------------------GDARLRLKTT----VEGIKYGKD---------------------------------------------------GVTITTDK-------------------------------------------------------G---D-----CIQADYAICTFSLGVLQS--------------------------NTTEFSPPLPD---WKQSAID-QFAMGTYTKIFMQFE--E-----AFWDNQ------------------------------------------------------TQFFLYADPLE---------------------------------------------------------------------------------------------RGRYPLFQSL----NP---------------------------------------------------------------EGFA-P---GSNILFGTVTGQQAWRVER----QTN------NETM----EQIL-DVLRLMFPDKN------------------------------VTTPTAFAYPRWSTEPWA-YGSYSN-WPVGM----------------T--LE-KHQ---NMRANV---------------------------ERLWFAGEANSA--EFFGFLHGAYTEGQDIANKIGNIINGKAG-------DDEFDMERYENLHGTT-----------------FVDEYDE---------------DNG------WLFPYDVEGDEEEEEEE---------------------------------------------------------------------------------------------------------------------------------------------------------------------------------------------------------------------------------------------------------------------------

>Fa_Nh1

------------------------------------------------------------------------------------------------------------------MRH--SYSQLLAVF------C-------------------GVL-----LGTSQ-AVAVPRGGTCKKTKVAIL-------------------GAGVA---------GITAAQTLSNAS-------IHD--------FLIVEHN-DYVGGRLRKTSFGEG---------------------PDGKPLTVEL-GANWVEGLES-----------EKGNTNPIWRL--A-QK---HGIKNTQSN-YT-KL-------------------------------------------LTYDEKGPADFSEE-------------IDEFDEKLEIA---MADAGLLMK---------------------------N---NLQDTSTRAGLGLAGWRP-GW--------------------------------DMKKQAAEWFG-----WDFEM--VYPPEQCGF-------LYTIAV-----QNATFD------HF----------------SDE-TNLVIDQRGFSAWLLGE--ADEFLEK---------------------------NDPRLLLNTT----VDKIAYDKN---------------------------------------------------GVKIITKD-------------------------------------------------------G---D-----CIEADYAICTFSVGVLQN--------------------------DVITFEPELPR---WKQEPIQ-QFQMGTYTKIFMQFN--E-----SFWPKD------------------------------------------------------TEFFLYADPKE---------------------------------------------------------------------------------------------RGYYPLFQAL----DA---------------------------------------------------------------PGFV-E---GSNVLFGTVTGQQSYHAEQ----QSD------EETL----EEIM-EVLHTIFPDTK------------------------------IPKPTSFMYPRWSQEEWA-FGSFSN-WPPGM----------------T--LE-KHQ---NMRANV---------------------------DRLWFAGEANSA--QFFGYLQGAYFEGQEIGDRIARIIGGKET-------EAALQMRRYEVLEGTT-----------------TPDEYNE---------------SNG------WSVPSE--------------------------------------------------------------------------------------------------------------------------------------------------------------------------------------------------------------------------------------------------------------------------------------

>Fa_Ff2

------------------------------------------------------------------------------------------------------------------MRH--STSQLLSLS------L-------------------GIL-----LGSSE-AASIPRKETCTKTKVAIL-------------------GAGVA---------GITAAQTLHNAS-------IHD--------FIILEHN-DHVGGRMKHTTFGKS---------------------SDGTPFTVEL-GANWIEGLQN-----------PSGEINPIWRL--A-QK---HKVKNTYSN-DS-AI-------------------------------------------ITYDETGASDYTDL-------------IDLFDEKFEIA---SQDAGYIFT---------------------------E---NLQDTSTRAGLSLAGWKP-KK--------------------------------DMKMAAADWWG-----WDFET--AYSPEESGF-------VYGVAG-----NNATFK------HF----------------SDE-TNLVIDQRGYNAWLVGE--ANDFLKK---------------------------NDPRLRLKTT----VKKIEYTTK---------------------------------------------------GVKIDTNN-----------------------------------------------------------G-----CVEADYAICTFSVGVLQN--------------------------DAVDFEPTLPR---WKREAIQ-VFQMGTYTKIFMQFN--E-----TFWPED------------------------------------------------------TQYFLYADPEQ---------------------------------------------------------------------------------------------RGYYPLFQSL----ST---------------------------------------------------------------PGFL-P---GSNILFGTVVQQQAYEVEQ----QSD------EKTK----KEIM-EVLRSMFPDKD------------------------------VPEPTAFMYPRWSMEEWS-YGSYSN-WPVGM----------------T--LE-KHQ---NLRANV---------------------------DRLWFAGEANSA--EFFGYLHGAYFEGQEIAERITRILKGEES-------EQSQQMKRYKTLRGTT-----------------ELEEHDS---------------ANG------WSTPLDD-------------------------------------------------------------------------------------------------------------------------------------------------------------------------------------------------------------------------------------------------------------------------------------

>Fa_Fo2

------------------------------------------------------------------------------------------------------------------MRH--STSQLLSIS------L-------------------GLL-----LGSSE-AASIPRKETCTKTKVAIL-------------------GAGVA---------GITAAQTLHNAS-------IHD--------FIILEHN-DYVGGRMKHTTFGKS---------------------SDGKPLTVEL-GANWIEGLQN-----------PSGEINPIWRL--A-QK---HKVKNTYSN-DS-AI-------------------------------------------ITYDETGASDYTEL-------------IDLFDEKFEIA---SQEAGYIFT---------------------------E---NLQDTSTRAGLSLAGWKP-KR--------------------------------DMKMAAADWWG-----WDFET--AYSPEESGF-------VYGVAG-----NNATFK------HF----------------SDE-TNLVIDQRGYNAWLVGE--ANEFLKK---------------------------NDPRLRLKTT----VKKIEYTTK---------------------------------------------------GVKIDTNN-----------------------------------------------------------G-----CVEADYAICTFSVGVLQN--------------------------NAVDFKPTLPR---WKRQAIE-QFQMGTYTKIFMQFN--E-----TFWPED------------------------------------------------------TQYFLYADPEQ---------------------------------------------------------------------------------------------RGYYPLFQSL----ST---------------------------------------------------------------PGFL-P---GSNILFGTVVQQQAYEVEQ----QSD------EKTK----KEIM-EVLRSMFPDKH------------------------------IPEPTAFMYPRWSMEEWS-YGSYSN-WPVGM----------------T--LE-KHQ---NLRANV---------------------------DRLWFAGEANSA--EFFGYLQGAYFEGQEIGERITRILKGEES-------EQSQQMKRYKTLRGTT-----------------EPEEHDA---------------ANG------WSTPLDD-------------------------------------------------------------------------------------------------------------------------------------------------------------------------------------------------------------------------------------------------------------------------------------

>Fa_Pn

-----------------------------------------------------------------------------------------------------------------------------------------------------------------------------------------------------------------------------------------------------------------------MQSFKFGSD---------------------ANGDPYTLEL-GANWVQGTGT-----------EGGPENPIWTF--A-KQ---ANLSNTNSN-LS-SI-------------------------------------------LTYDETGANDFLDL-------------IDEFEEKYAVA---EQNAGTILT---------------------------K---SLQDRSMRAGLWQGGWRP-KD--------------------------------A-HRKAIEWWE-----WDWEM--SQTPEESSF-------VFGITV-----YNFTFY------RY----------------SED-DNMSVDPRGFSIWLYAQ--AAKFLK---------------------------ANDPRLLLNTV----VKDIEYCDT---------------------------------------------------HVTITNED-------------------------------------------------------G---T-----CVEADYAINTVSLGVLQN--------------------------EVIKYTPELPS---WKQDSIA-TFAMGTYTKIFYQFN--E-----TFWPED------------------------------------------------------TQFFLYAHPTT---------------------------------------------------------------------------------------------RGYYTAWQSL----ST---------------------------------------------------------------EGFF-P---GSNILFVTVVDEQSYRIEA----QDD------EVTK----QEGL-AVLRQMFPDIN------------------------------IPEPVAFHYPRWTNTPWS-YGSFTN-WPSGT----------------T--LE-MHQ---NLRANV---------------------------GRLYFAGEATST--EHFGYLQGAWFEGQEAGLKIAGMLTQECRNA----ESGCGQHVKYEVLHGTT-----------------EAWEYNA---------------YNG------MGESPFFKPDASAES-----------------------------------------------------------------------------------------------------------------------------------------------------------------------------------------------------------------------------------------------------------------------------

>Fa_Pt

------------------------------------------------------------------------------------------------------------------MRLAKGWLLLVGLV------P-------------------------SLTFAAPAFHGRDYNSTCKRTKVAII-------------------GGGVA---------GITAAQALANQS-------VTD--------FLILEYQ-DHIGGRMRNTKFGSD---------------------PDGNPYTVEL-GANWISGLGQ----------DTDGPENPVWTF--S-KQ---VNLTSPNSD-AF-SI-------------------------------------------ATYNETGAVDYTDI-------------LDEFEDYWSKF---EQSAGTILS---------------------------E---NLQDRSFRAGLWQSGWRP-KS--------------------------------DPTRKAVEYYL-----WDWET--AQSPEGSSF-------VYGIAG-----YNFTYY------GF----------------SEM-SNFCTDQRGFSTWLKYQ--AAEFLQ---------------------------PNDPRVLLNTV----VTNIIYSDT---------------------------------------------------GVHIATSD-------------------------------------------------------G---S-----CVEADYAISTVSLGVLQN--------------------------DAITFEPELPE---WKQSAIA-NFHFGTYTKIFFQFN--E-----TFWPED------------------------------------------------------KQFFLYADPTT---------------------------------------------------------------------------------------------RGYYTVWQSL----ST---------------------------------------------------------------EGFL-P---GSNIIFATVVGDQSYRIEA----QDD------ETTK----AEGM-AVLRKMFPSII------------------------------VPEPIAFTYPRWTQTPWA-RGSYSN-WPAGT----------------T--LE-MHQ---NLRANV---------------------------GRLYFAGEAQSA--QYFGFLHGAWFEGQEVGERIAGQITTECVNR----PSGCGAYNRYEVLHGTT-----------------EFWEVNA---------------FNG------MGTSPFFVANSVVDAEGGDA------------------------------------------------------------------------------------------------------------------------------------------------------------------------------------------------------------------------------------------------------------------------

>Fa_At

----------------------------------------------------------------------------------------------------------------------MHIPSKYGLL------A-------------------ASAIQACSGYVAQPRANDATCKSTTKTTVAIL-------------------GGGMA---------GVTAAQALTNAS-------VTD--------FLILEYR-DTLGGRMWHTDFGKD---------------------ENGHPYTIEL-GANWVQGIGS-----------N-KTENPIWRL--A-KK---YNLKNHYSN-YD-SI-------------------------------------------LTYDEHGYVDFQNV-------------LDEYSEASEKA---TQEAGRLLV---------------------------Q---NAQDMTARSGFALAGWNP-GH-------------------------------DDMKAQAVEWWN-----WDWED--AWTPETSSF-------IFGMAG-----ENLTFN------QF----------------GED-NNLCIDQRGFNVLVTEE--AKTFLK-----------------------------PEQVRFNTQ----VTQVDYSSD---------------------------------------------------GVTIHTKN-------------------------------------------------------G---D-----CVRAAYAICTFSVGVLQR--------------------------DVIKWEPELPL---WKRTAIQ-KFEMGTYTKIFLQFN--E-----TFWPED------------------------------------------------------KQFFLYASSTT---------------------------------------------------------------------------------------------RGYYPVWQSL----ST---------------------------------------------------------------EGFF-P---GSNIIFVTVVQDQAYRAEL----QSD------EETK----EEVM-EVLRQMFPDKD------------------------------IPEPIAFMYPRWTSVPWA-YGSYSN-WPAGT----------------T--LE-VHQ---NLRANV---------------------------DRVWFAGEAISA--EYFGFLQGAWFEGREAGMQVAGLLQDRCVNIY--GDRVCGQRVHYDPLQGTT-----------------PIDAYTL---------------ING------WPVDSINLE-----------------------------------------------------------------------------------------------------------------------------------------------------------------------------------------------------------------------------------------------------------------------------------

>Fa_An3

------------------------------------------------------------------------------------------------------------------MHTIPITVSLYAFL------A-------------------TTFLFSCSAYVA--QAP-LSDDKCTETTVAIL-------------------GGGMA---------GIAAAQALSNAS-------IDD--------FIILEYR-DTLGGRVWHTDFGKD---------------------KQGKPYVIEL-GANWLQGLGS-----------E-AIENPVWAL--A-KK---YRLKNTYSN-YS-SI-------------------------------------------RTYNETGYTDYRYL-------------LDEYAQAYHIA---ARDAGRILT---------------------------Q---NLQDQTARTGLALAGWRP-RK-------------------------------NDMAAQAVEWWS-----WDWED--AHTPETSSL-------VFGIAG-----ENLTFN------QF----------------GKA-NHLVLDPRGYSTIIQNE--ALGFLPN--------------------------PSDGRLRLNTR----VTRIEYSPR---------------------------------------------------GVTIHTTNDNNK-------------------------------------------------NSN---T-----CIRAAYAICTFSLGVLQN--------------------------KAVTFDPPLPS---WKQTAIE-KFNMGTYTKIFMQFP--E-----TFWPTD------------------------------------------------------TQFFLYASPTT---------------------------------------------------------------------------------------------RGYYPVFQSL----ST---------------------------------------------------------------ENFL-P---ESNILFATVVDEQAYRVER----QSL------TQTK----DQIL-NVLREMFPDKH------------------------------IPEPTAFTYPRWTNEPWV-YGSYSN-WPAGT----------------T--LE-MHQ---NLRANT---------------------------GRLWFAGEATSA--AYFGFLHGAWYEGRDAGENVAALLQGRCVEDK---EEACGERVFYEQLNGTT-----------------PLDAYSR---------------LNG------WPAVSYY-------------------------------------------------------------------------------------------------------------------------------------------------------------------------------------------------------------------------------------------------------------------------------------

>Fa_Nf2

------------------------------------------------------------------------------------------------------------------MRLRYVLP----LV------A-------------------DWILQTCEGYVAQQVPL-RDNGRCARTTVAIL-------------------GGGVT---------GITAAQALANAS-------IDD--------FLILEYR-DRLGGRLRHEEFGED---------------------ENGNPYVVEL-GANWIHGVGM-----------G-VRENPIWQL--A-RK---HNLTVTHSN-YS-SI-------------------------------------------RTYNETGFIDYRHL-------------QREYAEANRAA---SREAGRIMT---------------------------E---NLQDQTARTGLALAGWRP-RK-------------------------------DDSAAQAVEWWN-----WDWES--AQTPDTSSL-------VFGLAA-----ENITFQ------QF----------------GAR-NELVIDPRGYSAIIIGE--AATFLYS---------------------ENGAPRMDHRVWLQTQ----VIEIEYSDK---------------------------------------------------GVTIRNSD-------------------------------------------------------G---S-----CVEAAYAICTFSLGVLQN--------------------------DAVTFRPALPG---WKQTAIH-KYTMGTYTKIFMQFE--K-----MFWPND------------------------------------------------------TQFFLYASPTT---------------------------------------------------------------------------------------------RGYFPVFQSL----SM---------------------------------------------------------------EGFL-P---GSNILFVTVVDAEAYRVER----QSD------PETQ----AEIL-DVLRQMFPDKH------------------------------VPEPKAFFYPRWSEEPWA-YGSYSN-WPVGT----------------T--LE-IHQ---NLRANV---------------------------QRLWFAGEATSS--AYFGFAHGAWYEGKEVGEHVAALLQGKCVTLQ--GQKACGERRHYEVLHGTT-----------------PLEAYNA---------------ING------WPMSSVEL------------------------------------------------------------------------------------------------------------------------------------------------------------------------------------------------------------------------------------------------------------------------------------

>Fa_Nfu1

------------------------------------------------------------------------------------------------------------------MRLRDALP----FV------A-------------------GWILQTCEGYVAQQVPL-GDHGRCARTTVAIL-------------------GGGMT---------GITAAQALANAS-------IDD--------FLILEYR-DRLGGRLRHAEFGED---------------------ENGNPYVVEL-GANWIHGVGM-----------G-VRENPIWQL--A-RK---HNLTVTCSN-YS-SI-------------------------------------------RTYNETGYTDYRHL-------------QREYAEAYRIA---SREAGRIMT---------------------------E---NLQDQTARTGLALAGWRP-RK-------------------------------DDSAAQAVEWWN-----WDWES--AQTPDTSSL-------VFGLAA-----ENITFQ------QF----------------GAR-NELVIDPRGYSAIINGE--AATFLAS---------------------ENGEPSMDPRVRLQTQ----VTQIEYSDK---------------------------------------------------GATIRNRD-------------------------------------------------------G---S-----CVEAAYAICTFSLGVLQN--------------------------DAVIFRPALPG---WKQTAIY-KYTMGTYTKIFMQFE--E-----MFWPND------------------------------------------------------TQFFLYASPTA---------------------------------------------------------------------------------------------RGYFPVFQSL----SM---------------------------------------------------------------EGFL-P---GSNILFVTVVDAEAYRVER----QSD------PETQ----AEIL-HVLRQMFPDKH------------------------------IPEPKAFFYPRWSEEPWA-YGSYSN-WPVGT----------------T--LE-THQ---NLRANV---------------------------QRLWFAGEATSS--AYFGFAHGAWYEGKEVGEHVAALLQGKCVTLQ--GQKACGERRHYEVLHGTT-----------------PLEAYNA---------------ING------WPMSSVDL------------------------------------------------------------------------------------------------------------------------------------------------------------------------------------------------------------------------------------------------------------------------------------

>Fa_Ma

------------------------------------------------------------------------------------------------------------------MHL--QHSTALFAI------A-------------------AVGIQSARAHV---ARSAPKDATCRKTSVAIL-------------------GGGMA---------GITAAQALSNNS-------ITD--------FVIIEYN-DRVGGRATQTNFGKK---------------------EDGSPYVVEL-GPNWIQGLGR-----------PGGPENPIWTL--A-KK---YNLKNTFSD-YT-SM-------------------------------------------LTYNETGYTDYSDI-------------LDEYDEAWTKA---SVRAGRMLA---------------------------E---NAQDETTRAGLAMAGWNP-KH-------------------------------TDMKRQAVEWWN-----WDWDA--ALTPEESSL-------IFGAAS-----DNLTFH------QF----------------SDH-NNLVIDPRGYRHIIEEE--SNTFLN---------------------------RNDNRLLLKTQ----ITNVTYSDD---------------------------------------------------GVTIHNSD-------------------------------------------------------G---S-----CISAAYAICTFSLGVLQN--------------------------NAVAFEPQLPE---WKRVAIQ-KFSMGTYTKIFMQFN--E-----TFWPTD------------------------------------------------------SQYFLYASPTT---------------------------------------------------------------------------------------------RGYYPVWQSL----ST---------------------------------------------------------------EGFM-P---GSNIIFATVTEEGSYRVEQ----QTD------EQTK----DEAL-EVLRQMFPNVT------------------------------VPEPLAFMYPRWTKAPWC-FGSYSN-WPIGT----------------T--LE-MHQ---NLRANT---------------------------GRLWFAGEATSA--ENFGFLHGAWFEGMEAGSQVAALLKGECAHVY--NGAECGGRVHYETLRGTS-----------------PLENYNV---------------LNG------WAASSF--------------------------------------------------------------------------------------------------------------------------------------------------------------------------------------------------------------------------------------------------------------------------------------

>Fa_An2

------------------------------------------------------------------------------------------------------------------MQL--SLLALLGAL-------------------------------ALPSAA---VPHARDEGQCKQTTVAIL-------------------GGGMA---------GIAAAQTLHNAS-------MDD--------FMILEYR-DTIGGRAWHKPFGQD---------------------KDGNPYIIEM-GCNWVQGLGT-----------PGGPQNPVWTL--A-QV---YNLSTIYSN-YS-NV-------------------------------------------STYNQHGYKDYSHL-------------IDTWDDIYDTA---AAQAGVMLL---------------------------D---NLQDQTAQTGLALAGWRP-KV-------------------------------DDMEAQAVDWWS-----WDFED--AYTPLESSF-------IFGVAG-----QNLTVN------GF----------------SDE-DNFVIDQRGYSHIIHGM--ASTFLK---------------------------PNDTRLLLNNH----ITNISYSDS---------------------------------------------------GVTVHSSD-------------------------------------------------------G---S-----CVRASYAICTFSLGVLQH--------------------------DAVTFTPSLPE---WKKEAIE-GFTMATYTKIFLQFN--E-----TFWPED------------------------------------------------------TQYFLYADPYM---------------------------------------------------------------------------------------------RGYYPVFQSL----ST---------------------------------------------------------------EGFF-P---GSNIIFVTVTEQFAWRAER----QSD------EKTK----AEVM-EVLRKMFPEKD------------------------------IPDPIAFMYPRWTLEPWA-YGSYSN-WPPST----------------T--LE-MHE---NLRANA---------------------------GRLWFAGEATSP--TYFGFLHGAWFEGQAAGLHLSSILNGTCK-----ANTTCKGRKHYETLHGTT-----------------PLSDYSF---------------VDG------WNGNSFYDFNDD--------------------------------------------------------------------------------------------------------------------------------------------------------------------------------------------------------------------------------------------------------------------------------

>Fa_Pc

------------------------------------------------------------------------------------------------------------------MHL--HHLSLLAAI------A-------------------GT-TL-PVIGA---PHQARTEGTCKKTKVAIL-------------------GGGVA---------GITAAQALTNAS-------VHD--------FVILEYR-DTIGGRAWHKPFGKD---------------------KDGKPYNIEM-GANWVQGIGS-----------EGGPQNPIWLL--A-QK---YGLKTEFSN-YD-NV-------------------------------------------STYNKDGYFDYSHL-------------IDAYDEAYEIA---NAKAGEILT---------------------------Q---NLQDQNAKSGLALAGWTP-KV-------------------------------HDMEAQAVDWWS-----WDFEA--AYSPIESSF-------VFGCAG-----DNLTFN------YF----------------SDH-DNLVIDQRGLNFIIKRI--ASTFLR---------------------------DNDPRLHLNTE----VTNITYSDH---------------------------------------------------GVRVHNKD-------------------------------------------------------G---S-----CVEADYAITTFSLGVLQR--------------------------GAVNFSPELPD---WKLEAIQ-KFNMGTYTKIFFQFN--E-----TFWPSE------------------------------------------------------TQYHLYADPVT---------------------------------------------------------------------------------------------RGWYPIWQSL----ST---------------------------------------------------------------PGFL-P---DSNIIFVTVTNEFAYRVER----QSD------EQTK----KEAM-DVLRKMFPDKD------------------------------IPEPTAFMYPRWTSEPWA-YGSYSN-WPPAT----------------S--LE-MHQ---NLRANA---------------------------GRLWFAGEATSP--TFFGFLHGAYFEGLDAGRQIAAIMQHRCINADSAKLRECGPRKHYETLHGTS-----------------PYSDYTM---------------LNG------WAVDSSIDNNPE--------------------------------------------------------------------------------------------------------------------------------------------------------------------------------------------------------------------------------------------------------------------------------

>Fa_Pd

------------------------------------------------------------------------------------------------------------------MHL--QHLSLWAAI------A-------------------GI-VQ-PTIGV---PHQTRTEGTCKKTKVAIL-------------------GGGVA---------GITAAQALTNAS-------VHD--------FVILEYR-DTIGGRAWHKPFGKD---------------------KDGKPYNVEM-GANWVQGIGS-----------KGGPQNPIWVL--A-QK---YGLNTEFSN-YD-NL-------------------------------------------LTYNKDGYSDYSNL-------------LDAYDEAYDIA---NQKAGEILT---------------------------Q---NLQDRNFKSGMALAGWNP-KV-------------------------------HDMEAQAVDWWS-----WDFEA--AYSPIESSF-------AFGCAG-----DNLTSN------FF----------------SDQ-DNFVIDQRGFNVILKGL--ASTFLI---------------------------DNDPRLHLNTE----VTNITYSDR---------------------------------------------------GVTVHNKD-------------------------------------------------------G---S-----CVEADYAITTFSLGVLQN--------------------------GAINFSPELPD---WKQESIQ-KFTMGTYTKIFFQFN--E-----TFWPSE------------------------------------------------------TQYHLYADPVT---------------------------------------------------------------------------------------------RGWYPIWQSL----ST---------------------------------------------------------------PGFL-P---DSNIIFVTVTNELAYRAER----QTD------EQTK----KEAM-EVLRKMFPEKD------------------------------IPEPTAFMYPRWTTEPWA-YGSYSN-WPPAT----------------S--LE-MHQ---NFRANV---------------------------GRLWFAGEATSP--TFFGFLHGAYYEGQDAGRQIAAIMQQRCVNADSAKLRECGPRKHYKTLHGTS-----------------PYSDYTM---------------LNG------WAVDSSIDNNPE--------------------------------------------------------------------------------------------------------------------------------------------------------------------------------------------------------------------------------------------------------------------------------

>Fa_Af1

------------------------------------------------------------------------------------------------------------------MFL--GHLSLIAFG--------------------------AWAVHPVVGYV---SQPRDQEGTCQKATVAIL-------------------GAGIS---------GISAAQTLSKAS-------VDD--------FLILEYR-DRIGGRAWHENFGQD---------------------KDGNPYVVEM-GANWVQGLGN-----------PGGPENPIWTL--A-KE---FGLQTTYSN-YS-NV-------------------------------------------STYNQDGYKDYSHL-------------LDECDEAYDIA---NQAAGKILV---------------------------E---NLQDQTAKAGLALAGWKP-KS-------------------------------HDMEAQAVDWWT-----WDFEA--SFTPLESSL-------VFGMAS-----DNLTSN------QF----------------SDH-DNFVTDQRGFNTIIKGM--ASKFLT---------------------------EDDPRLLLNTK----VTNITYGPE---------------------------------------------------GVTVYSSD-------------------------------------------------------G---N-----CVQAAYAICTFSLGVLQN--------------------------DVVTFTPELPE---WKKTAIQ-MFTMGTYTKIFLQFN--E-----TFWPTD------------------------------------------------------TQYFLYADPAT---------------------------------------------------------------------------------------------RGYYPLFQSL----SM---------------------------------------------------------------DGFH-P---GSNIIFVTVTDELAQRAER----QSD------EETK----QEIM-EVLRKMFPDVD------------------------------VPEPTAFLYPRWNTEPWS-YGSYSN-WPMGT----------------T--LE-MHE---NLRANT---------------------------DRLWFSGEATSP--SYFGFLHGAWFEGRDAGRRIAGLLNGCKE----GNSTTCVPRKHYEVLHGTS-----------------PLADYSS---------------VNG------WEVSSFYDSNDD--------------------------------------------------------------------------------------------------------------------------------------------------------------------------------------------------------------------------------------------------------------------------------

>Fa_Aso

------------------------------------------------------------------------------------------------------------------MFL--GHLSLIAFG--------------------------AWAVHPVVGYV---SQPRDQEGTCQKTTVAIL-------------------GAGIS---------GISAAQTLSKAS-------VDD--------FLILEYR-DRIGGRAWHENFGQD---------------------KDGNPYVVEM-GANWVQGLGN-----------PGGPENPIWTL--A-KE---FGLQTTYSN-YS-NV-------------------------------------------STYNQDGYKDYSHL-------------LDECDEAYDIA---NQAAGKILV---------------------------E---NLQDQTAKAGLALAGWKP-KS-------------------------------HDMEAQAVDWWT-----WDFEA--SFTPLESSL-------VFGMAS-----DNLTSN------QF----------------SDH-DNFVTDQRGFNTIIKGM--ASKFLT---------------------------EDDPRLLLNTK----VTNITYGPE---------------------------------------------------GVTVYSSD-------------------------------------------------------G---N-----CVQAAYAICTFSLGVLQN--------------------------DVVTFTPELPE---WKKTAIQ-MFTMGTYTKIFLQFN--E-----TFWPTD------------------------------------------------------TQYFLYADPAT---------------------------------------------------------------------------------------------RGYYPLFQSL----SM---------------------------------------------------------------DGFH-P---GSNIIFVTVTDELAQRAER----QSD------EETK----QEIM-EVLRKMFPDVD------------------------------VPEPTAFLYPRWNTEPWS-YGSYSN-WPMGT----------------T--LE-MHE---NLRANT---------------------------DRLWFSGEATSP--SYFGFLHGAWFEGRDAGRRIAGLLNGCKE----GNSTTCVPRKHYEVLHGTS-----------------PLADYSS---------------VNG------WEVSSFYDSNDD--------------------------------------------------------------------------------------------------------------------------------------------------------------------------------------------------------------------------------------------------------------------------------

>Fa_Ac2

-------------------------------------------------------------------------------------------------------------------MH--LHLTLTAIA------G-------------------CVLQSATAYVA---QEPRSAPPTCQKTTVAIL-------------------GAGMA---------GIKAAETLTNAS-------IHD--------FVILEYR-DTIGGRVWHTEFGKD---------------------ENGDPYLVEM-GANWIQGIGT-----------EDGPQNPIWTL--A-KE---YKLNNTFSD-YA-NV-------------------------------------------STYNHHGYSNYSHL-------------IAEFDAVEGIA---SAAAGTILT---------------------------E---NLLDQTAQTGLALAGWKP-KK-------------------------------TDMEAQAVDWWS-----WDFET--AYPSLESSL-------VFGYAG-----SNLTWN------GF----------------SDE-DNLVWDQRGYNTIIKGM--ASKFLP---------------------------ADDPRLRLNTQ----VANITYSDK---------------------------------------------------GVTVHNRD-------------------------------------------------------G---T-----CVQAQYALCTFSLGVLQN--------------------------DAVTFTPQLPL---WKRTAIE-KFTMGTYTKIFLQFN--E-----TFWPAD------------------------------------------------------TQYMLYADPKL---------------------------------------------------------------------------------------------RGRYPIWQSL----ST---------------------------------------------------------------PGFL-P---GSNIIFATVTNDFAYRVET----QSD------DETK----AELM-HVLRSMFPDKA------------------------------LPEPTAIMYPRWSTEPWA-YGSYSN-WPPAT----------------S--LE-EHE---NLRANT---------------------------GRLWFAGEHTSA--SYFGFLHGAWFEGRDAGRQIAALLQKRCVYYNSTRERLCGPRKHYETLHGFT-----------------PLADYSA---------------VNG------WTGNSFYDYNHD--------------------------------------------------------------------------------------------------------------------------------------------------------------------------------------------------------------------------------------------------------------------------------

>Fa_Nf1

----------------------------------------------------------------------------------------------------------------------------------------------------------------------------------------------------------------MA---------GIKAAETLSNAS-------IHD--------FVILEYR-DTIGGRAWHTNFGKD---------------------ENGDPYVVEL-GANWIQGIGT-----------PDGPQNPIWTL--A-KE---FNLKNTFSD-YD-NV-------------------------------------------STYNENGYSDYSHL-------------FNEFDAADEIA---NAAAGTILL---------------------------E---NLLDQTARTGLALAGWKP-KK-------------------------------TDMEAQAVEWWN-----WDFED--AYSPLESSL-------VFGYAG-----SNLTWN------GF----------------SDE-DNFVLDQRGYNTIIKGM--AAKFLK---------------------------ANDTRLRLNTQ----ITNITYSDK---------------------------------------------------GVTVYSSD-------------------------------------------------------G---T-----CVQAQYALCTFSLGVLQN--------------------------DAVTFTPELPY---WKQTAIQ-KFTMGTYTKIFLQFN--E-----TFWPSN------------------------------------------------------TQYFLYADPKL---------------------------------------------------------------------------------------------RGWYPIWQSL----ST---------------------------------------------------------------PGFL-P---GSNILFVTVTNEFSYHVEN----QSD------EETK----AEVM-AVLRKMFPDKD------------------------------IPEPTAFMYPRWSTEPWS-YGSYSN-WPAST----------------G--LE-EHQ---NLRANT---------------------------GRLWFAGEHTSP--SYFGFLHGAYFEGLDAGRQIAALLQGRCVYYNSTMERLCGPRRHYERLHGIT-----------------PLADYSA---------------VNG------WISNSFYDYNDE--------------------------------------------------------------------------------------------------------------------------------------------------------------------------------------------------------------------------------------------------------------------------------

>Pd_Sl4

-------------------------------------------------------------------------------------------------------------------------------------------------------------------------------MVSQKPKIVII-------------------GAGIA---------GLTAAKKLYTTQN---TNELFE--------VCVVEGG-NRIGGRIFTTEFCG---------------------------DRVEM-GATWIHGIE----------------GNPIYKI--A-QE---INGFETD-------------------------KPWD------SMG--GKVD-K----KLTITEEGHEVH-S------------SFVNSISNFFNNLLEFSSGEGDFDGG---------------VGRKIVESLNLEENGRVDKISMGSYLRKGLEFYWESFNLE---------------DENVEVFENWSRKALEEGIFAMFE-NIH-RHYSS--AGDLGTLDF-------NGE--SE----Y----------------------------CNFP-GDEITIAKGYSSIVESL--ASVLPP------------------------------GLIQLGRK----VSKIEWQLETSD---------------------------------------------GNKPVKLHF-------------------------------------------------------SDG---S-----VMYADHVIVTVSLGVLKQGIRED----------------------SSLFSPPLPK---FKTEAIS-RLGFGVVDKVFLQLTPTH-----H-----------------------------------------------------DGMNFPNMMMVFHQSNAK------------------------------------------------------------------------------------------LKNPKIPLWIRRTTLTH---------------------------------------------------------------------PVYPESRVVVSWFAGEEALKVET----LDD------DEII----EGVS-ITMSEFLSNTK--------------------------HYKNSIKFSKVLKCKWGTDPLF-LGSYTH-IAVGS----------------S--GD-DLD---AMAEPLPKEISD------------DKNSKKSPRLQVLFAGEATSR--NYYSTTHGAYLTGLREANRLLEYFQCVDV-------------------------------------------------------------------------------------------------------------------------------------------------------------------------------------------------------------------------------------------------------------------------------------------------------------------------------------------------------------------

>Pd_Br8

--------------------------------------------------------------------------------------------------------------------------------------------------------------------------------MLKKPRVVII-------------------GAGMA---------GLTAANKLYTHSN----NTTFD--------LSVFEGG-SRIGGRINTFEFSS---------------------------ERIEM-GATWIHGIG----------------GSPVYKI--A-EE---AGSLVSD-------------------------EPWE------CMD--STAD-K----ARTFAEGGFEVD-P------------SVVEPVSGLFNALMELAQGKKKEKE----------------KKITQNDVVSGLGSIYENATSVGSFLKSGFDAYRDATGNG--------------G-----------NGSLEEAIFTMFS-NTQ-RTYTS--ADDLWTLDY-------AAE--SE----Y----------------------------QMFP-GEEITIAKGYVSVIHHL--ASVLPH------------------------------GVIQLNRT----VTKIEWESC------------------------------------------------EECPVRLHF-------------------------------------------------------SDG---S-----VVFADHVIVTVSLGVLKAGIESD----------------------DELFSPPLPE---FKSDAIK-RLGYGVVNKLFVEVSE---------------------------------------------------------------RNFPSLQLVFGREDSD------------------------------------------------------------------------------------------SRFVKIPWWMRRTATIA---------------------------------------------------------------------PIHSNSKVLLSWFAGKEAIELEK----LTD------EEII----DGVL-TTISCLTGKQVKN-------------GYVNG-N-----DNEVLRITKVLKSKWGGDPLF-RGSYSY-VAVGS----------------S--GD-DLD---ALAEPLPKINKK-------SGQGKCHDQTKVHELQVMFAGEATHR--THYSTTHGAYYSGMREANRLLKHYKCDF--------------------------------------------------------------------------------------------------------------------------------------------------------------------------------------------------------------------------------------------------------------------------------------------------------------------------------------------------------------------

>Pd_At5

--------------------------------------------------------------------------------------------------------------------------------------------------------------------------------MAKKARIVII-------------------GAGMA---------GLTAANKLYTSSN-----NTFE--------LSVVEGG-SRIGGRINTSEFSS---------------------------EKIEM-GATWIHGIG----------------GSPVYRI--A-KE---TGSLVSD-------------------------EPWE------CMD--STID-K----AKTFAEGGFEIE-P------------SIVESISGLFTALMELAQGKEISQSDAD------------LSRLAHIYETATRVCSKGSSTSVGSFLKSGFDAYWDSISNG--------------GEEGVKGYGKWSRKSLEEAIFTMFS-NTQ-RTYTS--ADELSTLDF-------AAE--SE----Y----------------------------QMFP-GEEITIAKGYLSVIHHL--ASVLPQ------------------------------GVIQLNRK----VTKIEWQSN---------------------------------------------------EVKLHF-------------------------------------------------------SDG---S-----VVFADHVIVTVSLGVLKAGIETD----------------------AELFSPPLPD---FKSDAIR-RLGYGVVNKLFVEMSQ---------------------------------------------------------------RKFPSLQLVFDREDSE------------------------------------------------------------------------------------------FRFVKIPWWMRRTATIT---------------------------------------------------------------------PIHSNSKVLLSWFAGKEALELEK----LTD------EEIK----DAVM-TTISCLTGKEVKN-----DTAKPLTNGSLNDDD-------EAMKITKVLKSKWGSDPLF-RGSYSY-VAVGS----------------S--GD-DLD---AMAEPLPKINKK-------VGQVNGHDQAKVHELQVMFAGEATHR--THYSTTHGAYYSGLREANRLLKHYKCNF--------------------------------------------------------------------------------------------------------------------------------------------------------------------------------------------------------------------------------------------------------------------------------------------------------------------------------------------------------------------

>Pd_Bj1

--------------------------------------------------------------------------------------------------------------------------------------------------------------------------------MAKKPRIVII-------------------GAGMA---------GLPAANKLYTASN-----NSFD--------LSVVEGG-SRIGGRINTSEFSS---------------------------EKIEM-GATWIHGIG----------------GSPIYKI--A-EE---TGSLVSE-------------------------EPWE------CMD--STVD-K----ARTFAEGGFEIE-P------------PIVEPVSGLFNALMELAQGKDIENDDGGD-----------LGEIYEIATRFYSSVNGLNGSSVGSFLRSGFEAYWASVSKG---------------GNGVKEYGTWSRRSLEEAIFTMFS-NTQ-RTYTS--ADDLYTLDY-------AAE--SE----Y----------------------------QMFP-GEEITIAKGYLSVIHHL--ASVLPQ------------------------------GVVELNRR----VTKIEWESN------------------------------------------------EEDPVKLHF-------------------------------------------------------SDG---S-----VVFADHVIVTVSLGVLKAGIESD----------------------GGLFSPPLPE---FKSDAIK-RLGYGVVNKLFVEVSQ---------------------------------------------------------------RRFPSLQLVFEKEDSE------------------------------------------------------------------------------------------YRFVKIPWWMRRTATMA---------------------------------------------------------------------PIHSNSKVLLSWFAGKEALELEK----LPD------EEII----DGVL-TTVSCLTGKKVKK--DNGKAPKTLANGSLREDD-----GEELVKITKVLTSKWGGDPLF-RGSYSY-VAVGS----------------S--GD-DLD---AMAEPLPQINKK-------SGQVNGHGQAKVRELQVMFAGEATHR--THYSTTHGAYYSGLREANRLLKHYKCDF--------------------------------------------------------------------------------------------------------------------------------------------------------------------------------------------------------------------------------------------------------------------------------------------------------------------------------------------------------------------

>Pd_Br7

--------------------------------------------------------------------------------------------------------------------------------------------------------------------------------MAKKPRIVII-------------------GAGMA---------GLTAANKLYTASN-----NSFD--------LSVVEGG-SRIGGRINTSEFSS---------------------------EKIEM-GATWIHGIG----------------GSPIYKI--A-EE---TGSLVSE-------------------------EPWE------CMD--STVD-K----ARTFAEGGFEIE-P------------PIVEPVSGLFNALMELAQGKDIENDDGGD-----------LGEIYEIATRFYSSVNGLNGSSVGSFLRSGFEAYWASVSKG---------------GNGVKEYGTWSRRSLEEAIFTMFS-NTQ-RTYTS--ADDLYTLDY-------AAE--SE----Y----------------------------QMFP-GEEITIAKGYLSVIHHL--ASVLPQ------------------------------GVVELNRR----VTKIEWESN------------------------------------------------EEDPVKLHF-------------------------------------------------------SDG---S-----VVFADHVIVTVSLGVLKAGIESD----------------------GGLFSPPLPE---FKSDAIK-RLGYGVVNKLFVEVSQ---------------------------------------------------------------RRFPSLQLVFEKEDSE------------------------------------------------------------------------------------------YRFVKIPWWMRRTATMA---------------------------------------------------------------------PIHSNSKVLLSWFAGKEALELEK----LPD------EEII----DGVL-TTVSCLTGKKVKK--DNGKAPKTLANGSLREDD-----GEEVVKITKVLTSKWGGDPLF-RGSYSY-VAVGS----------------S--GD-DLD---AMAEPLPQINKK-------SGQVNGHGQAKVRELQVMFAGEATHR--THYSTTHGAYYSGLREANRLLKHYKCD---------------------------------------------------------------------------------------------------------------------------------------------------------------------------------------------------------------------------------------------------------------------------------------------------------------------------------------------------------------------

>Pd_CBp_Ah

-------------------------------------------------------------------------------------------------------------------------------------------------------------------------------MVVKKAKIVII-------------------GAGMA---------GLTAANKLYTAEG---SKDLFD--------ISVVEGG-GRIGGRINTAEFMG---------------------------ERIEM-GATWIHGIG----------------GSPIYKI--A-EQ---IGALHSD-------------------------QSWE------CMD--GYSG-Q----STTVAEGGIELS-P------------ATVDPISTLFQMLMDFAQGKITGDSD-------------------------ILQQANYDKRSIGEFLQQGIDSYWVSKNGE----------------TEVNGCKEWSQKSLEEAIFAMYE-NNQ-RTYTS--AGDLSTLDF-------ISE--SE----Y----------------------------QMFP-GEEITIAKGYLSVIESI--ASVLPP------------------------------GLVQLGKK----VTKIEWHPELDPP-------------------------------------------NIPTPVTLHF-------------------------------------------------------ADG---S-----HISADHVIVTVSLGVLKAGTQPD--------------------SPSPLFHPPLPS---FKTEAIS-RLGFGVVNKLFLRLAPVT-----ENGLNL-----------------------------------------------KRTHQFPCLNFVFHQPDXE------------------------------------------------------------------------------------------VPAEKIPWWMRKTTSLR---------------------------------------------------------------------PIYQNSSLLLSWLAGEEALHLEK----LKD------DEII----NGVS-TTISNFLIQN-------------------------------EFSFSQVLKSQWGSDPLF-LGSYSY-VAVGS----------------S--GE-DLD---AMAEPLPRTEE----------------SSKSPLLQILFAGEATHR--THYSTTHGAYFSGLREANRLLHHYNCT---------------------------------------------------------------------------------------------------------------------------------------------------------------------------------------------------------------------------------------------------------------------------------------------------------------------------------------------------------------------

>Pd_CBp_As

-------------------------------------------------------------------------------------------------------------------------------------------------------------------------------MVVKKAKIVII-------------------GAGMA---------GLTAANKLYTAEG---SKDLFD--------ISVVEGG-GRIGGRINTAEFMG---------------------------ERIEM-GATWIHGIG----------------GSPIYKI--A-EQ---IGALHSD-------------------------QSWE------CMD--GYSG-Q----STTVAEGGIELS-P------------ATVDPISTLFQMLMDFAQGKITGDSD-------------------------ILQQANYDKRSIGEFLQQGIDSYWVSKNGE----------------TEVNGCKEWSQKSLEEAIFAMYE-NNQ-RTYTS--AGDLSTLDF-------ISE--SE----Y----------------------------QMFP-GEEITIAKGYLSVIESI--ASVLPP------------------------------GLVQLGKK----VTKIEWHPELDPP-------------------------------------------NIPTPVTLHF-------------------------------------------------------ADG---S-----HISADHVIVTVSLGVLKAGTQPD--------------------SPSPLFHPPLPS---FKTEAIS-RLGFGVVNKLFLRLAPVT-----ENGLNL-----------------------------------------------KRTHQFPCLNFVFHQPDXE------------------------------------------------------------------------------------------VPAEKIPWWMRKTTSLR---------------------------------------------------------------------PIYQNSSLLLSWLAGEEALHLEK----LKD------DEII----NGVS-TTISNFLIQN-------------------------------EFSFSQVLKSQWGSDPLF-LGSYSY-VAVGS----------------S--GE-DLD---AMAEPLPRTEE----------------SSKSPLLQILFAGEATHR--THYSTTHGAYFSGLREANRLLHHYNCT---------------------------------------------------------------------------------------------------------------------------------------------------------------------------------------------------------------------------------------------------------------------------------------------------------------------------------------------------------------------

>Pd_Sl3

-------------------------------------------------------------------------------------------------------------------------------------------------------------------------------MVSKKAKVVII-------------------GAGMA---------GLTAANKLYTTAG---CKDLLD--------LCVVEGG-NRIGGRINTSEFGG---------------------------DRIEL-GATWIHGIG----------------GSPVHEI--A-QQ---INSLQSE-------------------------QPWE------CMD--GLLE-T---EAITIAEDGYVLD-S------------SFVEPISNLFNKLMDISQSKLVTQN------------------------------EIPNVMSVGSFLRRGIDAYWDEHV------------------DELEGLDKRRKRSLEQGIFAMFE-SIQ-RTYTS--ANDLEMLDF-------NAE--KE----Y----------------------------CMFP-GEEITIAKGYLSVIESL--ASVLPP------------------------------GLIQLGRK----VTKIEWQPDELLEIE---------------------------------------NGTNKPPVKLHF-------------------------------------------------------VDG---S-----IMYADHVIVTVSLGVLKQGIRED----------------------SGMFNPPLAS---FKTEAIT-RLGFGVVNKVFLKLTSIP-----D------------------------------------------------------GINFPYLQMVFHNES--------------------------------------------------------------------------------------------EQNPKIPWWMRRTANLC---------------------------------------------------------------------PIYGNSNVLLSWFVGKEALEVES----LSD------EEII----DGFS-KTISSFLINSH-------------------------SDAESMFKVEKVLKSQWGNDPLF-LGSYSY-VAIES----------------S--GD-DLD---AMAEPLPKKITS-------L----HDSNVSPPPLQILFAGEATHR--THYSTTHGAYFSGIREANRLLQHYHCIDI-------------------------------------------------------------------------------------------------------------------------------------------------------------------------------------------------------------------------------------------------------------------------------------------------------------------------------------------------------------------

>Pd_Gm10

-------------------------------------------------------------------------------------------------------------------------------------------------------------------------------MVAKKPLIVII-------------------GAGMA---------GLTAANKLHSVSA---SKDLFE--------VCVVEGG-NRIGGRINTSEFGG---------------------------DRIEM-GATWIHGIG----------------GSPIHKI--A-QQ---IHALDSE-------------------------QPWE------CMD--GNEN-K----ATTIAEGGFVLNPS------------SHVDPITKLFNNLMDHAQRKMPTTT-----------------------------KGDCGNLSVGSFLKQGLDAYCGSSK-E---------------EEELKGFGKWSKKLLDEAIFAVHE-NTQ-RTYTS--AADLFNLDY-------AAE--SE----Y----------------------------QMFP-GEEITIAKGYLSIIESL--ASVLPP------------------------------GLVQLGRK----VTRIEWQPERHEAMN----------------------------L-------ENGRPCSSRPVMLHF-------------------------------------------------------CDG---S-----IMSADHVIVTVSLGVLKASIRDD-------------------DSGMLMFNPPLPS---FKAEAIS-RLGFGVVNKLFMQLSEPP-----H----------------------------------------------------EHSKGFPFLQMVFHSPQSE------------------------------------------------------------------------------------------LRHKKIPWWMRRTATLC---------------------------------------------------------------------PIYNNSSVLLSWFAGEEALALES----LKD------EEII----EGVS-DTISCFLSN-----------SLEFCNGNVNSEK---YSHEYKVKFSKVLKSKWGTDPLF-LGSYSH-VAVGS----------------S--GD-DLD---TMAEPLPKCLT-----------------CASPPLQILFAGEATHR--THYSTTHGAYFSGLREANRLLQHYSLC---------------------------------------------------------------------------------------------------------------------------------------------------------------------------------------------------------------------------------------------------------------------------------------------------------------------------------------------------------------------

>Pd_Gm9

-------------------------------------------------------------------------------------------------------------------------------------------------------------------------------MVVKKPRIVII-------------------GAGMA---------GLTAANKLYTATA---SKDLFE--------LCVVEGG-TRIGGRINTSEFGG---------------------------DRIEM-GATWIHGIG----------------GSPIHKI--A-QE---IHSLHSD-------------------------QPWE------CMD--GNTV-TDDATTITIAEGGFHLHHP------------SIVDPITKLFNTLMEYSQGKLNDTT---SKGGSELESYQKLAAKVASV-SASSNNNNKNNLSVGSFLRQGLEAYQVSKE-E---------------QEEVKGCGNWSRKLLEEAIFAMHE-NNQ-RTYTS--ADDLFTLDY-------GAE--SE----Y----------------------------IMFP-GEEITIAKGYLSIIEYL--ASVLPP------------------------------GLVQLGKK----VTRIEWQLDDEKRKK----------------------------GGAVEN--NGCCSSSSRPVKLHF-------------------------------------------------------CDG---S-----VMYADHVIVTVSLGVLKAAILDD------------------DDDDSGMFYPPLPP---SKTEAIS-RLGFGVVNKLFMQLSPTH-----GGLKQHEN-------------------E-------------------------QSDKGFPFLQMAFHSPQSE------------------------------------------------------------------------------------------MRNKKIPWWMRRTATLF---------------------------------------------------------------------PIYNNSSVLLSWFVGEEALALES----LKD------EEII----NGVS-STVSFFLQQ-----------NE--------------V------KFSKVLKSKWGTDPLF-LGSYSY-VAVGS----------------S--GD-DLD---IMAEPLPKDNSS-------CQ---ASSAASSSPLQILFAGEATHR--THYSTTHGAYFSGLREANRLLQHYHCVGIYNN----------------------------------------------------------------------------------------------------------------------------------------------------------------------------------------------------------------------------------------------------------------------------------------------------------------------------------------------------------------

>Pd_Ca1

-------------------------------------------------------------------------------------------------------------------------------------------------------------------------------MVVKKPRIVII-------------------GAGMA---------GLTAANKLYTSTS---SKDLFE--------LCVVEGG-TRIGGRINTSEFGG---------------------------DKIEM-GATWIHGIG----------------NSPIHKI--A-QQ---TNSLHSQ-------------------------QPWE------CMD--GNNN---DESIITIAEGGFHLQ-P------------SIVDPVTKLFKSLMDYSQGKLNEET---SKKCELFSYYNMAAK------AS---NFGSKNLSIGSFLRKGLDAYFESVKDE---------------EEVKGYGENWNRKLLEEAIFAMHE-NTQ-RTYTS--AGDLEFLDY-------EAE--SE----Y----------------------------RMFP-GEEITIAKGYLSIIEYI--ASVLPP------------------------------GLIQLGRK----VKRIEWKSDDDDVVD----------------------------DDD------EKNGCCFRPVKIHF-------------------------------------------------------CDG---S-----VMYADHVIVTVSLGVLKASICNH--------------D-DGDGDSGMFFCPPLPN---SKVEAIS-RLGFGVVNKLFMQLSPTT-----NEKYS--K-------------------------------------------------GFPFLEMVFHSTT-K------------------------------------------------------------------------------------------NEKKIIPWWMRRTATLF---------------------------------------------------------------------PIYNNSSVLLSWFAGEEALALES----LND------EEII----NGVS-STISSFLPQ-----------LQ--------------QKKDSSLLFSKVLKSKWGTDPLF-LGSYSY-VKVGS----------------S--GE-DLD---TMAEPLPMMKK---------------D--SNFPLQILFAGEATHR--THYSTTHGAYFSGLREANRLLQHYHFVGIFNN----------------------------------------------------------------------------------------------------------------------------------------------------------------------------------------------------------------------------------------------------------------------------------------------------------------------------------------------------------------

>Pd_Mt4

-------------------------------------------------------------------------------------------------------------------------------------------------------------------------------MVVKKPKIVII-------------------GAGMA---------GLTAANKLYTSTA---SKDLFE--------LIVVEGG-TRIGGRINTSEFGG---------------------------DKIEM-GATWIHGIG----------------NSPIHKI--A-QQ---IHSLHSD-------------------------QPWE------CMD--GNNS-N-DESLTTISEGGFNLQ-P------------SIVDPVSKLFKYLMEYSQGKLTKET---AKGEEVLSYYNMAVK------AASSNFASKKNLSIGSFLRQGLDAYFESLKDE---------------EEEVKGYGDWNKKLLEEAVFAMYE-NTE-RTYTS--AGDLECLDY-------EAE--SE----Y----------------------------RMFP-GEEITIAKGYLSIIEYI--ASVLPP------------------------------GLIQLGKK----VKKIEWQSQKKS-----------------------------------------YDDNCFRPVKLHF-------------------------------------------------------CDG---S-----IMYADHVIVTVSLGILKASISHH----------------DDDDDKGMLFSPNLPS---FKVEAIS-RLGFGVVNKLFMQLSTQK-----TTNLD--D-------------------E-------------------------NSEGLFPFLQMVFHSPQNE------------------------------------------------------------------------------------------TKDKKIPWWMRKTATLF---------------------------------------------------------------------PIYNNSSVLLSWFAGEEALALES----LKD------EEII----NGVT-STVSSFLPQ-----------NE--------------V------KFDKVLKSQWGTDPLF-LGSYSY-VQVGS----------------S--GE-DLD---TMAEPLPMMKD---------------NSNFSYPLQILFAGEATHR--THYSTTHGAYFSGLREANRLLQHYHCVGIFNN----------------------------------------------------------------------------------------------------------------------------------------------------------------------------------------------------------------------------------------------------------------------------------------------------------------------------------------------------------------

>Pd_Sl2

-------------------------------------------------------------------------------------------------------------------------------------------------------------------------------MVTKKPKVVII-------------------GAGMA---------GLTAANKLYTSAG---CKEFIE--------VCVVEGG-DRIGGRIITSEFVG---------------------------SRIEM-GATWIHGIG----------------GSPVYKI--A-QE---INSLHSD-------------------------KPWE------CMN--EFLD-E----PLTIAEGGYHLN-S------------SLVDPICNLFKKLMDFAQGKPHVQF-----------------------------PSSNGNLSIGSFLRKGLDAYWGSIKEQ----------------EETLGFDNWTRKSLEEGIFAMLE-NTQ-RTYTS--AGDLETLDF-------NAE--SE----Y----------------------------RMFP-GKEITIAKGYSTIVESL--ASVLPD------------------------------GMIQLGRK----VTKIEWQPDDGEN------------------------------------------CDVIKPVKIHF-------------------------------------------------------CDG---S-----IMYADHVIVTVSLGVLKQGIHNN----------------------TGMFSPPLPR---FKTEAIS-RLGFGVVNKLFLQLSPNR-----D--------------------------------------------------------EFPNLQMVFHQSDSK------------------------------------------------------------------------------------------LRHRRIPWWMRRTASLC---------------------------------------------------------------------PIYGTSNVLLSWFAGKEALEVES----LSD------EEII----DGFS-KVISDLLEN-----------SKNKCNGHA----------DSAIKFDKVLKTKWATDPLF-LGSYSY-VAVGS----------------S--GD-DLD---SMAEPLPKTNN---------------HDLNSLQLQILFAGEATHR--THYSTTHGAYFSGLREANRLLQHYKYIDI-------------------------------------------------------------------------------------------------------------------------------------------------------------------------------------------------------------------------------------------------------------------------------------------------------------------------------------------------------------------

>Pd_Cc5

--------------------------------------------------------------------------------------------------------------------------------------------------------------------------------MAKKPRIVII-------------------GAGMA---------GLTAANKLYTTPS---SKDLFE--------LCVVEAG-TRIGGRINTSEFGG---------------------------DRIEM-GATWIHGIG----------------GSPIYKI--S-QE---INSLESH-------------------------QPWE------CMD--GFSS-Q----RTTVAEGGFEVN-P------------SIVEPVSSLFDSLMDYAQGKLS----EESTSCEKAEFCKLTCKAFKICSSSNG--DSSGKLSVGSFLRQGLNSYWDFLKEQ----------------DELKGYGTWSRKLLEESIFAMNE-NTQ-RTYTS--AGDLMTLDF-------TAE--SE----Y----------------------------QMFP-DEEITIAKGYMSIIEHL--ASVLPP------------------------------GFIQLGRK----VTNIEWKPAVEIENG----------------------------YG-------NGASGTGRSVKLHF-------------------------------------------------------SDG---S-----TVLADHVIVTVSLGVLKAGINHD----------------------SGMFSPPLPS---FKTEAIS-RLGYGVVNKLFVQLSPST-----SHDTPKG--------------------D--------------------------SLSKFPFLHLAFHRPDSE------------------------------------------------------------------------------------------FRNKKIPWWMRRTAALC---------------------------------------------------------------------PIYKKSSVLLSWFAGEEALELES----LSD------EDII----NGVS-TTISSFLSQ---PQKKVESNSHELWNGNVNHH--VESSKGSEVKFTKVLKSKWGNDPLF-LGSYSY-VAVGS----------------S--GD-DLD---TMAEPLPKIG-P------------NFESSAIPPLQILFAGEATHR--THYSTTHGAYFSGLREANRLLQHYHSVGA-------------------------------------------------------------------------------------------------------------------------------------------------------------------------------------------------------------------------------------------------------------------------------------------------------------------------------------------------------------------

>Pd_Pt6

-------------------------------------------------------------------------------------------------------------------------------------------------------------------------------MVAKKPRIVII-------------------GAGMA---------GLTAANKLYTSSS---SNDMFE--------LCVVEGG-SRIGGRINTSEFGG---------------------------DRIEM-GATWIHGIG----------------GSPVHKI--A-QE---IHSLESE-------------------------QPWE------CMD--GLLD-E----PKTVAEGGFELS-P------------SLVESISTVFKNLMDYAQGKLI----EREESSEEVDFCKLADKI---CKICPSNGGGPGKLSVGSFLRQALNVYWDSVKEQ----------------EQIEGCGNWSRKLIEEAIFAMHE-NIQ-RTYTS--AGDLLTLDF-------DAE--SE----Y----------------------------RMFP-GEEITIAKGYLSVIESL--ASVLPH------------------------------GLIQLGRK----VARIEWQPEAHQSSG---------------------------------------HGCAGRPVKIHF-------------------------------------------------------CDG---S-----IMSADHVIVTVSLGVLKAGIGPD----------------------SGMFNPPLPT---FKTEAIS-RLGFGVVNKLFLQLSSRH-----DGRDG-------------------------------------------------DYSKFPFLQMAFHRPDSE------------------------------------------------------------------------------------------WRHKKIPWWMRRTASLS---------------------------------------------------------------------PIYKNSGVLLSWFAGKEALELET----LSD------EEII----DGVS-TTLSSFLSQ---PHKQLNSNSHGVCNGKEKSVD------GNRVRFANVLKSKWGNDPLF-LGSYSY-VAVGS----------------S--GD-DLD---TLAEPLPNTD-----------------TLGSAPLQILFAGEATHR--THYSTTHGAYFSGLREASRLLQHYHCVGV-------------------------------------------------------------------------------------------------------------------------------------------------------------------------------------------------------------------------------------------------------------------------------------------------------------------------------------------------------------------

>Pd_Rc5

-------------------------------------------------------------------------------------------------------------------------------------------------------------------------------MVAKKPRIVII-------------------GAGMA---------GLTAANKLYTSST--SSKDMFE--------LCVVEGG-TRIGGRINTSEFGG---------------------------DRIEM-GATWIHGIG----------------GSPVHKI--A-QE---INSLESK-------------------------QPWE------CMD--GFWN-E----PKTIAEGGFELN-P------------SLVESISTLFKNLMDFAQGKLVQA--SESSSGDGVDFYNLAAKASKICTSNGGGVNVAGKRSIGSFLRQGLDAYWDSVKDQ----------------EQIKGYCTWSRKLLEEAMFTVHE-NIQ-RTYTS--AGDLLTLDF-------DAE--SE----Y----------------------------QMFS-GEEITIAKGYLSIIESL--ASVLPK------------------------------GLIQLGRT----VARIEWQPEANHSME---------------------------------------NGHGHKPVQLHF-------------------------------------------------------NDG---S-----VMCADHVIVTFSLGVLKAGIGQD----------------------SGMFSPPLPS---FKTEAIS-RLGYGVVNKLFLQLSPKN-----DAVTKGG--------------------D-------------------------DVKAKFPFLQMAFHRQDSQ------------------------------------------------------------------------------------------LRHKRIPWWMRRTASIS---------------------------------------------------------------------PIHKNSSVLLSWFAGKEALELES----LSD------EEII----NGVS-TTISSFLQQQTQPNKIVSSKAHELCNGSVSSENCVESSKGSEIKFSKVLKSRWGNDPLF-LGSYSY-VAVGS----------------S--GD-DMD---KLAEPLPRIG--------------NFETDGCPQLQILFAGEATHR--THYSTTHGAYFSGLREANRLLQHYHCVGI-------------------------------------------------------------------------------------------------------------------------------------------------------------------------------------------------------------------------------------------------------------------------------------------------------------------------------------------------------------------

>Pd_Pr1

-------------------------------------------------------------------------------------------------------------------------------------------------------------------------------MVAKKSRIVII-------------------GAGMA---------GVTAANKLFTATG---SKDLFE--------LCVVEGG-SRIGGRINTSEFGG---------------------------DRIEM-GATWIHGIG----------------GSPVHKI--A-QE---IHALESE-------------------------QPWE------CMD--GSSD-E----PKTIAECGFELT-P------------TLVDPISSLFKNLMDYAQGKKVFDESTETESNGDVEYSKLGDKASEVCTSNG----GLGKLSVGSFLRQGLDAYWVSKKNQ-------------DEELKLNGYGNWSRRLLEEAIFAMHE-NTQ-RTYTS--AGDLFTLDY-------SAE--SE----Y----------------------------RMFP-GEEITIAKGYLSIVQSL--ASVLPP------------------------------GLIQLGRK----VTKIEWQQPETHINN---------------------------------------GYDDTRPVKLHF-------------------------------------------------------SDG---S-----VMLADHVIVTVSLGVLKASIQQD----------------------SGMFNPPLPS---FKTEAIS-RLGFGVVNKLFLQLSSTH-----ATKGQ-------------------------------------------------DFSKFPFLQMVFHRADSE------------------------------------------------------------------------------------------FRHKKIPWWMRKTASLC---------------------------------------------------------------------PLYHNSSVLLSWFAGKEALELES----LKD------EEII----NGVS-ATVSSFLSKPQ-KKERENCHSHELCNGNVNSQE------NSEVKIAKVLKSQWGNDPLF-LGSYSY-VAVGS----------------S--GE-DLD---TMAEPLPKLNSC------------GDEPATSPPLQILFAGEATHR--THYSTTHGAYFSGLREANRLLQHYHHVGV-------------------------------------------------------------------------------------------------------------------------------------------------------------------------------------------------------------------------------------------------------------------------------------------------------------------------------------------------------------------

>Pd_Vv7

-------------------------------------------------------------------------------------------------------------------------------------------------------------------------------MVAKKPRIVII-------------------GAGMA---------GLTAANKLYTSTG---FKDLFE--------LCVVEGG-TRIGGRINTSQFGG---------------------------DRIEM-GATWIHGIV----------------GSPIHKM--A-QE---LHSLESD-------------------------QPWE------CMD--GYLD-S----PTTMAEGGFELG-P------------STVDPVSTLFKKLMDFSQGKLIEDS-----------------------------------------------------------------------------VCSEELSLLEEAIFAMHE-STQ-RTYTS--AGDLSTLDY-------DAE--SE----Y----------------------------IMFP-GEEVTIAKGYLSIIEAL--ASVLPA------------------------------GLIQLGRE----VTKIEWQP---------------------------------------------------EPVKLHF-------------------------------------------------------CDG---S-----TMSADHVIVTVSLGVLKAGICGD----------------------SGLFNPPLPS---FKTEAIS-RLGYGVVNKLFVQLSPSH-----DHEGK-------------------------------------------------KLNKFPFLQMVFHRSDSE------------------------------------------------------------------------------------------LRHQKIPWWMRRTASVC---------------------------------------------------------------------PIYNNSSVLLSWFAGKEALELEK----MKD------EEIL----NGVS-VTVTSLLSN--------------------------------EVKFIKVLKSKWGTDPLF-RGSYSY-VGVGS----------------S--GE-DLD---SMAKPLPESS--------------KSGANACPPLQILFAGEATHR--THYSTTHGAYFSGLREANRLLQHYNCVGVSELLQH-------------------------------------------------------------------------------------------------------------------------------------------------------------------------------------------------------------------------------------------------------------------------------------------------------------------------------------------------------------

>Pm_Ma7

-------------------------------------------------------------------------------------------------------------------------------------------------------------------------------MVAKKPRVVIV-------------------GAGMA---------GITAAHRLHTASS----GDHFD--------LCIVEAS-HRTGGRILTSEFAG---------------------------DRIEM-GATWIHGIR----------------GSPIHAL--A-SD---IGALAHD------------------------SCPWE------RMD--GFPS-D----PITVAEGGTLIDPS------------LVVAPITSLYRRLMDSARAGD----------------------------------APVD---------------------P---------------KRPGVGHTDWNLEELEEAVFTMHE-FTE-RTCTS--ADDLEELNL-------AAE--GE----Y----------------------------RDYP-GDHITIAQGYSRIVQYL--ASALPR------------------------------GMIRFGRL----LQRIEWRSGSDDDGG----------------------------GD--------DGCRDGDPVRLYF-------------------------------------------------------EG--EHS-----AMVADHVIVTVSLGVLKAGLGKR--------------G---EEGSGVAFSPALPV---FKREAIE-RLGFGVVNKLFMEMDAAE-----GGGP------------------------------------------------------FPFLQMAFAQEEEEEE-------------------------------------------------------------------------------------DRRRRVAEIPRWIRKTASIC---------------------------------------------------------------------PIYDSSRVLQAWFAGKEALELEA----LPD------EEVI----RSIH-LTLHAFLPGVAT--------------------------------RAAGSRSGWGKDPLF-LGSYSY-VAVGS----------------S--GD-DLD---LMAEPLPRRGRG-------GEPDDRDVVSPPPALQILFAGEATHR--THYSTTHGAYLSGIREANRLLQHYRYTSAAL-----------------------------------------------------------------------------------------------------------------------------------------------------------------------------------------------------------------------------------------------------------------------------------------------------------------------------------------------------------------

>Pm_OBp_Ah

-------------------------------------------------------------------------------------------------------------------------------------------------------------------------------MVAKKPRVVVV-------------------GAGIS---------GLAAAHRLCGA-G----GDRFE--------VAVVEAG-DRVGGRILTSEFAG---------------------------HRVEM-GATWVQGVV----------------GSPVYAL--A-RD---AGALGEEE---------------------GRGLPYE------RMD--GFPD-R----VLTVAEGGEVVDAD------------TVAGPIEELYRGMMEAARAGE----------------------------------AG-GGGGVEEYLRRGLRAYQAARSA------------------GGGGGGGKELEEVDEALLAMHI-NRE-RTDTS--ADDLGDLDL-------TAE--GE----Y----------------------------RDFP-GEHVTIPGGYSRVVERL--AAALPP------------------------------GTVRLGLR----LRRLKWGGT---------------------------------------------------PVRLHF-------------------------------------------------------ADGA--P-----PLTADHVILTVSLGVLKASLGNK--------------DTAGVGAAAIAFDPPLPP---FKREAVA-RLGFGVVNKLFMEVEAVA-----PSEPEDVAG------VQ------------------------------------PAAAGFPFLHMA-----------------------------------------------------------------------------------------------FRGHVSKIPWWMRGTESIC---------------------------------------------------------------------PVHAGSTVALAWFAGREAAHLES----LPD------DDVI----RGAH-ATLDSFLPAA------------------------------PRWRVRRIKRSGWATDPLF-LGSYSY-VAVGS----------------S--GD-DLD---RMAEPLPRGPDA-------A------ADERPPSPRLLFAGEATHR--THYSTTHAAYLSGVREANRLLQHYRGGANHTT----------------------------------------------------------------------------------------------------------------------------------------------------------------------------------------------------------------------------------------------------------------------------------------------------------------------------------------------------------------

>Pm_Si5

-------------------------------------------------------------------------------------------------------------------------------------------------------------------------------MVTRKPRVVIV-------------------GAGVA---------GLTAARRLGAAGG----GDRFE--------VAVVEAG-ARAGGRVLTSEFAG---------------------------HRIEM-GATWVQGVD----------------GSPVYAL--A-RD---AGALGSRSG-NKDEDG-----------DAAGSLPYE------RMD--GFPD-R----VLTVAEGGEVVDAD------------RVARPVEELYRGMMEAARAGE----------------------------------AR-GGGGVEEYLRRGLRAYQAARPAA---------------AAAAAGGENKELEEVEEALLAMHI-NRE-RTDTS--ADDLGDLDL-------AAE--GE----Y----------------------------RDFP-GEHVTIPGGYSRVVDHL--VAALPP------------------------------GTVRLGLR----LRRLDWRGS---------------------------------------------------PVRLHF-------------------------------------------------------ADGA--P-----EITADHVILTVSLGVLKASLGGK--------------DAS--AAGAIAFDPPLPQ---FKREAVA-RLGFGAVNKLFMEVEPAE-----APGPEG--------------GG-------------------------------GQPPEFPFLHMA-----------------------------------------------------------------------------------------------FRGHVAKIPWWMRGTESIC---------------------------------------------------------------------PVHAGSRVVLAWFAGREAAHLES----LPD------DEVI----RGLQ-ATLDSFLPGP------------------------------PQWRVKRIKRSGWATDPLF-VGSYSY-VAVGS----------------N--GE-DLD---RMAEPLPRGSRG--------------DDGRASPPRVLFAGEATHR--THYSTTHAAYMSGVREAERLLQRYR-----------------------------------------------------------------------------------------------------------------------------------------------------------------------------------------------------------------------------------------------------------------------------------------------------------------------------------------------------------------------

>Pm_Sb5

-------------------------------------------------------------------------------------------------------------------------------------------------------------------------------MVAKKPRVVVV-------------------GAGVA---------GLTAAHRLCAG-G----GDRFE--------VTVVEAG-ARAGGRVLTSEFAG---------------------------HRVEM-GATWIQGID----------------GSPVYAL--A-RD---AGALACD----------------------RDAPPYE------RMD--GSPD-R----VLTVAEGGEVVDAD------------RVARPVEELYRGMMEAARAGE----------------------------------SS-GGGSVEEYLRRGLRAYQAARQ-----------------------G-GKELEGIEDALLAMHI-NRE-RTDTS--ADELGDLDL-------AAE--GE----Y----------------------------RDFP-GEHVTIPGGYSRVVDRL--VAALPP------------------------------GTVRLGLR----LRRLDWSDT---------------------------------------------------PVRLHF-------------------------------------------------------AEDGATT-----AITADHVILTVSLGVLKASIGK---------------DAH--AAGGVAFDPPLPQ---FKRDAVA-RLGFGVVNKLFMELEAVP-----AAKPEGDGG------GGGGGGSEHPLAM-------------------------SAPPEFPFLHMA-----------------------------------------------------------------------------------------------FRGHVSEIPWWMRGTESIC---------------------------------------------------------------------PVHGGSSVALAWFAGREAEYLES----LPD------DEVI----RGVQ-ATMDSFLPAPDPRD---------------------GAKATSRWRVKRIKRSGWATDPLF-LGSYSY-VAVGS----------------S--GE-DLE---RMAEPLPRGS----------------NVGGAPPLRVLFAGEATHR--THYSTTHAAYLSGVREAERLLQHYGN----------------------------------------------------------------------------------------------------------------------------------------------------------------------------------------------------------------------------------------------------------------------------------------------------------------------------------------------------------------------

>Pm_Zm4

-------------------------------------------------------------------------------------------------------------------------------------------------------------------------------MVATKPRVAVV-------------------GAGVA---------GLTAAHRLCAA-G----GERFE--------VTVVEAG-ARAGGRVLTSEFAG---------------------------HRVEM-GATWVQGVD----------------GSPVYAL--A-RD---AGALACG----------------------KDLPPYE------RMD--GFPG-R----VLTVAEGGEEVDAD------------RVASPIEELYRGMMEAARAGE----------------------------------AG-GGGGVEEYLSRGLRAFQAARP-----------------------GGGKELEEVEDALLAMHI-NRE-RADTS--ADDLGDLDL-------ATE--GE----Y----------------------------RDFP-GEHVTIPAGYSRVVDHL--VAALPP------------------------------DTVRLGLR----LRRLDWSET---------------------------------------------------PLRLHF-------------------------------------------------------DDGA--T-----AISADHVILTVSLGVLKASLGK---------------DAH--AAGGIAFDPPLPQ---FKREAVA-RLGFGVVNKMFMELEAVP-----AARPE----------GDRGGGGEHPLAA-------------------------SAPPEFPFLHMA-----------------------------------------------------------------------------------------------FRGHVSEIPWWMRGTESIC---------------------------------------------------------------------PVHAGSSVALAWFAGREAEHLES----LPD------DEVI----RGVH-STLDSFLPAP------------------------------SRWRVKRIKRSGWATDQLF-LGSYTY-VPVGS----------------S--GE-DLD---RMAEPLPRRLDA-------DV-----DVARAPPPRVLFAGEATHR--THYSTTHAAYLSGVREAERLLQHYA-----------------------------------------------------------------------------------------------------------------------------------------------------------------------------------------------------------------------------------------------------------------------------------------------------------------------------------------------------------------------

>Pm_Bd2

-------------------------------------------------------------------------------------------------------------------------------------------------------------------------------MVANKPRIVIV-------------------GAGIA---------GLSAAQQLCRAGQ----GDKFD--------VVVVEAA-PRAGGRVFTSEFAG---------------------------HRVEM-GATWVQGIT----------------GSPVYAL--A-HD---AGALTEDA---------------------GGHLPYE------RMDGPFPDD-R----VLTVAEGGDVVDAD------------KVAKPVEELYRGMMDAARAGE----------------------------------AGGGGGGVEEYLRRGLRAYQAARTD----------------------GSGSKVKEVEEALLAMHI-NRE-RTDTS--ADALGDLDL-------AAE--GE----Y----------------------------RDFP-GDHVTIPGGYTRVVEHL--VAALPP------------------------------GTVRLGLR----LRRLDWGET---------------------------------------------------PVRLHF-------------------------------------------------------ADDG-AA-----ALIADHVILTVSLGVLKASLGK---------------DA-ASAAGAIAFDPPLPQ---FKRDAVS-RLGFGVVNKLFVELEAVE-----PEGGGE---------------EQQLAGA-------------------------AAPPDFPFLHMA-----------------------------------------------------------------------------------------------FDGHVAKIPWWMRGTESIC---------------------------------------------------------------------PVHAGSSVALAWFAGREAKHLEF----LPD------DDVV----RGVQ-ATLDSFLPATSS------------------------SGATSRWRVKRIERSRWAGDPLF-VGSYSY-VAVGS----------------S--GG-DLD---RMAEPLPRGGVP-------E------ADRTPPPLRVLFAGEATHR--THYSTTHAAYLSGVREADRLLQHYP-----------------------------------------------------------------------------------------------------------------------------------------------------------------------------------------------------------------------------------------------------------------------------------------------------------------------------------------------------------------------

>Pm_Hv5

-------------------------------------------------------------------------------------------------------------------------------------------------------------------------------MVEKKPRIVIV-------------------GAGIA---------GLSAAQQLCGAGR-----EKFE--------VVVVEAG-CRAGGRVFTSEFAD---------------------------HRLEM-GATWVQGIL----------------GSPVYAL--A-RE---AGALREEA---------------------A-DLPYE------RMD--GFPD-G----VLTVAEGGGVVDAN------------TVAKPIEELYRGMMEAARAGE----------------------------------AVGEGGGVEEYLRRGLRAYQARRPG-----------------------GRKELEEVEEALLGMHI-NRE-RTDTS--ADDLGDLDL-------PAE--GE----Y----------------------------RDFP-GDHVTIPGGYTRVVEHL--VAALPP------------------------------GTVRLGLR----LRRLDWGET---------------------------------------------------PVRLHF-------------------------------------------------------AGEA-TT-----TLTADHVILTVSLGVLKASIGK---------------DVSATATGAIAFDPPLPQ---FKREAVE-RLGFGVVDKLFIEVEAVE-----TPEPDG---------------GYAQLAR-------------------------TAQPAFPFLHMA-----------------------------------------------------------------------------------------------FLGDAAKIPWWMRGTESVC---------------------------------------------------------------------PVHAGSTVALAWFAGREAAHLES----LPD------DEVI----SALQ-STLESFLPAQPRRC------------------SWAGAGATPRWRVKRIKRSGWAADPLF-LGSYSY-VAVGS----------------S--GE-DLD---RMAEPLPRGPE-----------------ADRTPLRVLFAGEATHR--THYSTTHAAYLSGVREADRLLQHYC-----------------------------------------------------------------------------------------------------------------------------------------------------------------------------------------------------------------------------------------------------------------------------------------------------------------------------------------------------------------------

>Ct-Cs2

----------------------------------------------------------------------------------------------------------------------------------------------------------------------------------------------------------------MS---------GIALARNLTDAG-------Y-R--------VLVLEGR-DRVGGRLNSTTTVA-------------------------PGSHVDL-GAMWIHDGI--------------AGRNPLYDL--V-VS---LGLKVSPRQ-DYGSI-------------------------------------------ATFSYTGQRTNR-------------SSFGRM---FSSWYSQLQPALARLKG-----------------------A-----TPADNRSFGDVYGEWLAANTQFT-------------------------A-----ADRGQANM-MM-NTN-YQSLL--NGNVTQLSV-------ARL--GD----A----------------------------KSIP-AVDVMLKDGFPALVDAL--VA-KGG------------------------------LDIRKNTV----VTSVQQDGDG---------------------------------------------------VIVAT-------------------------------------------------------ADG---T-----NYTAPYVVMTQTLGTLKAGD--------------------------IVFDPELPP---EKLQAIE-EMGFGVLDKALFVFD--K-----PFWDTS----------------------------------------------------VDF---LLRE-----------------------------------------------------------------------------------------------MPDWSGRWSIFLNYHKL-----------------------------------------------------------------------FGWPILAAIHVADTARELEK----LTD------EQVI----GEGM-AVLRQLYPG--------------------------------APQPVQTAFTRWAAEPLS-RGAYSY-FAVGN----------------P--KN-ITR---TLAQPHG---------------------------RVLFAGEATS---DKPATVLGAYLSGLREAERLRTLLGKPTGTSA----------------------------------------------------------------------------------------------------------------------------------------------------------------------------------------------------------------------------------------------------------------------------------------------------------------------------------------------------------------

>AMd-Va

------------------------------------------------------------------------------------------------------------------------------------------------------------------------------MERQAKLKVIVI-------------------GAGVS---------GLTTATELKKSG-------LVD--------VLVLEAQ-DRLGGRTWTVDLPESP------------------NGENKNPVPIDM-GAAWIHGV----------------TGNPLIAR--A-VE---AGASFHDFG-EENLI------------------------------------------PPIFDVDGNSITD-------------AQKVLPEKLFEKLNEYIVE-MKP-VL-----------------------K-----KEGRDFSLKEAVKKMCENDVQFS-------------------------DCWNSLGVEGQLLQ-WL-VGS-VESFY--ACPPSDLSV-------AYY--DD----W----------------------------KMLP-GGDYLMKSGYSTLVRHL--ASLLSP------------------------------FEWRLSSP----IKAIDWSSPSSSSSS-------------------------------------GRR--QRRQITVTT-------------------------------------------------------ERG---E-----EFVADVVVVTVSLGVLKSKT--------------------------IQFQPQLPP---FKLGAIE-KMGFGLMDKVVLRFP--R-----VFWHSC----------------------------------------------------AND---LYLG-----------------------------------------------------------------------------------------------TTDPTEQFRWWVNCNPI-----------------------------------------------------------------------CQEPILVCLVVSHFAYQIEDITLKSGD------QVVV----DRAM-KVLRACFEKENV-------------------------GLGGVPDPTNSYITKWSANPYT-RGSYSY-VSVGS----------------D--GPAAFD---DLAAPLA-------------------------DGHLRFAGEATYF--DYQGCVHAAYESGIRESSLILQNLGDLAQLSHKL--------------------------------------------------------------------------------------------------------------------------------------------------------------------------------------------------------------------------------------------------------------------------------------------------------------------------------------------------------------

>SD-Pt1

----------------------------------------------------------------------------------------------------------------------------------------------------------------------------------------------------------------MS---------GLSCARELQHRG-------Y-H--------VLVVEAR-QRVGGRLKGTALQLP-----------------------TGEQQVDL-GGALIHGI----------------DDNPVAEL--V-DQ---IGVRTRPV---SDT--------------------------------------------LLLDKTGWPLDL-------------REDERISHLFNECLEEAFERTRG---------------------------------KQSDTSFGDLFNTVCEGKAVNT-------------------------S--------A-ILR-WH-KAN-LEVSC--GTSFEKLGW-------QWN-EDE----A----------------------------YGFD-GDHVALQASWKPVVEAL--AE---P------------------------------LDIVYNAS----VELIHLTGPR------------------------------------------------NTVVQITL-------------------------------------------------------MDG---T-----VLEADSVVCTVPLGILKRKT--------------------------ISFDPPLPT---PKQQAIE-RLGIGLLNKCTLSFP--H-----VFWQDS----------------------------------------------------DFL------G-----------------------------------------------------------------------------------------------LAEDEHSYL-VLNGATF-----------------------------------------------------------------------TDNPVLLFMFGGEFAHEIEK----WTD------TEIV----TDCL-RILSRICGC-------------------------------QVPEPTDYHTTRWGREQYS-RMAFTF-IPPGV----------------D--GAAELR---AMGEPVLNSIG--------------------NVPALMFAGEHTTF--FHPSTIHGAFFSGIREAYRL----------------------------------------------------------------------------------------------------------------------------------------------------------------------------------------------------------------------------------------------------------------------------------------------------------------------------------------------------------------------------

>CR-Gt2

--------------------------------------------------------------------------------------------------------------------------------------------------------------------------APEPANRKTQPSVIVI-------------------GAGFA---------GLSAADELHALG-------C-K--------VVVLEGR-DRIGGRCWTDKSLD--------------------------GRTVDL-GAGWIHGI----------------VGNPLAEL--A-RR---KGVELCNIP-AD-T--------------------------------------------LIHDADGVVYSE-------------ETDRKIELLFNQFLQRAQKEVGT-G------------------------------SQKSDQSLGGLLDRMIASDDSLD-------------------------D-----ARELQLFN-WH-CAN-IEYST--ATDIHNLSA-------RNWALDD----E----------------------------NAFD-GDHCLLKSGYCALAEHL--AQ---G------------------------------LDIRLNSK----VKVIEHGKEGQ-----------------------------------------------QAACKVTL-------------------------------------------------------EDG---R-----TLSSDIVVLTVPLGVLKSKS--------------------------IAFYPQLPR---WKQAAID-KLGFGVLNKVVLAFS--K-----IFWQRA----------------------------------------------------TPI-GKYIGY-----------------------------------------------------------------------------------------------ASERKGQFYLFIDITDC-----------------------------------------------------------------------ASKPTLLALISGSMAKELEV----TPD------DEVV----REAM-KVLEKVVGE------------------------------GACEQPCGYKITRWGQDPFA-MGSYSY-VAIGC----------------T--PE-DMD---ALARPLD-------------------------HNRLFFAGEHTNS--EHPSTVHGAFISGRRVARELLVSWHGHGEVREGSRCV-EFPLR-----------------------------------------------------------------------------------------------------------------------------------------------------------------------------------------------------------------------------------------------------------------------------------------------------------------------------------------------------

>Cla_Cr

-------------------------------------------------------------------------------------------------------------------------------------------------------------------------------MRDEPLDVLVI-------------------GAGIS---------GLAAASALQRHG-------L-R--------VAVLESR-ARVGGRIHTVQIGP-------------------------HGPSVDL-GAAWIHGIG------------SAQAPNPLFAL--A-SR---AGLGAAPTD-YADA--------------------------------------------ATYTAGGTRLPP-------------SAVSEMEDIYNAFEQHLRSLLRS-PD-----------------------P------QPALQPLSVALDRYAACAGLSP-------------------------A-----QHVA-LSF-AA-SNH-MEHYW--AGDMHSMGV-------AAL--DE----E-----------------------------VLP-GGDVVLPGGYSGLVGTL--AA---G------------------------------LDVRLGHQ----VKHIRYGVGHNDGGG-------------------------------------GGASGTGAGVAVTVHIASPQ--------------PLVP--------------SEHPGHAQAAAGVAVDGGRL---V-----TLHARAAVVTLPLGVLRSGG--------------------------VAFSPPLGATDPAKAAAIG-ALGTAVYNKVIMYFDPAD-----VFWDNT----------------------------------------------------AFI---YRMP-----------------------------------------------------------------------------------------------RPHEAGRWSYFLNLHKV-----------------------------------------------------------------------TGAPVLIAFNLGEEAAALEA----LSD------EAAV----SGAL-AALAGVYGP------------------------------SRVRRPWAALVTRWGSDPHS-RMSYTY-IPAGV----------------T--TA-ALD---DLARPVA--------------------------GRLFFAGEATHR--AHYGTAHGAYDSGLRAAAALLQQLAAEAAQEVAGRAGQRLPLQPRLRLLPPPPPR--H---------QQRERMAAGKGSSWGKPQTVGVLGPDQW------LATSAAATGAVLQESGGGA----------PATMGVGSSPA---------------------------------AAAATAMVFAVPHNGGVVPAAASGTIGGSGEDSSCGLAGGGANEGQQEQ-GGSDRKQEDEEEDYGKVGQARVPPRPAARSRM--------------------------------------------------------------------------------------------------------------------------------

>Bcy_Ca1

-----------------------------------------------------------------------------------------------------------MINRRFFLIVSSVILAHVVASKNFA-------------------------------------SNSLKTTKKNKEKIIII-------------------GAGIA---------GLTAGKTLQNQG-------F-E--------VILLEAR-NRIGGRLWTSKKWD--------------------------NAFVDM-GASWIHGE----------------EGNPITKL--A-NT---INAQVFSTK-SEKS--------------------------------------------IIYDLNGKEIIE-------------DKEEKLDKLTN----KLKEIINK-IQ-----------------------N-----NYYYDISLQKALEKELKWQTLSD-------------------------V-----NKQY-LEY-LL-NSN-IEQEY--AADISQLSA-------FYF--DE----G----------------------------KAFD-GDDSLFIKGYNVISDYL--AQ---G------------------------------LNIKLNHT----VEAIGVAAPSVNASN-------------------------------------------SQGVNVIT--------------------------------------------------------NK---S-----NFQADRVIVTLPLGVLQKNI--------------------------VKFSPALPE---KKLEAIN-QLGMGVLNKLYVLFP--K-----RFWQNN----------------------------------------------------YDW---IG-K-----------------------------------------------------------------------------------------------ISEKKGQWSEWVNLESA-----------------------------------------------------------------------LKKPILLGFNAGKFGKEIES----WSD------EEII----ADAM-KTLRQIYGN-------------------------------SIPQPIDYQLTRWSQDPFT-FGSYSY-YATNS----------------T--PN-HRQ---ELAKPIN--------------------------KKVFFAGEATSI--DYPATVHGAYFSGLRVSQEIIALTN-----------------------------------------------------------------------------------------------------------------------------------------------------------------------------------------------------------------------------------------------------------------------------------------------------------------------------------------------------------------------

>Bcy_Sy

-----------------------------------------------------------------------------------------------------------MIRRRSFFKLSQLMFVSYLLSTSCG-------------------------------------KNNTPVTANDAPSILII-------------------GAGLA---------GLAAAQSLMKQG-------Y-T--------VRVLEAR-DRLGGRTWTSNYWD--------------------------DAPLDM-GASWIQGT----------------EGNPITEL--A-EK---IATPLVMTS-YDNA--------------------------------------------ITYEVGGQPFTA-------------KEDRIIEQLEK----KWQGAIAT-AQ-----------------------N------GDGDQSLQAVIENVFDLENQPL-------------------------E-----TKQI-IDW-YM-NST-IEHEY--AGSLKDTSI-------YWF--DG----D----------------------------GGFG-GDDAIFVEGYQAIVNYL--AK---D------------------------------ISIELNQI----VESIDYSEEI---------------------------------------------------PKIIT--------------------------------------------------------NQ---G-----AYTADQVIITLPLGVLKSGQ--------------------------VKFIPELPS---PKRKAIK-ALGMGILNKCYLRFP--K-----VFWPKK----------------------------------------------------VDW---IE-Q-----------------------------------------------------------------------------------------------VPTERGLWSEWVNIFRV-----------------------------------------------------------------------NQLPILLGFNAADEGKEIET----WTD------EEII----KSAM-KTLRHLFGD-------------------------------DIPDPTDYQITRWQSDSFS-RGSYSF-NALGS----------------H--PD-MRD---HLAKSLN--------------------------DQIFFAGEATER--DYFATAHGAYLSGLRVAEEINNL-------------------------------------------------------------------------------------------------------------------------------------------------------------------------------------------------------------------------------------------------------------------------------------------------------------------------------------------------------------------------

>Bcy_Ma

------------------------------------------------------------------------------------------------------------MYRRNFLKIFGLTLMGSAHSACSE-------------------------------------KSQGDIATSSKKRVVVI-------------------GAGLS---------GLAAAQELHRQG-------N-E--------VVVVEAR-DRIGGRIWTSSKWT--------------------------DMPLDF-GATWIHGT----------------EGNPLTDL--A-DQ---INAKRLTTS-YDRA--------------------------------------------VTYNTSGQLLSN-------------AEEVRLEKTRN----KVFGELKK-AQ-----------------------N------EDPDISLRQAIEPLIRQFDKSS-------------------------E-----SYRF-INF-IL-SGE-IEHEY--SGSAERLSA-------HWY--DS----D----------------------------KKFN-GNDDLFVQGFRVIPEFL--GQ---G------------------------------LRIELGQV----VKEIQWHQSP---------------------------------------------------IRVIT--------------------------------------------------------QN---T-----EFLADHVIVTLPLGVLQAGK--------------------------VRFTPELPQ---DKQTAIA-KLGMGTLNKCYLRFP--D-----VFWSAD----------------------------------------------------VDW---LE-Y-----------------------------------------------------------------------------------------------ISASHGEWTEWVSFNRA-----------------------------------------------------------------------ANMPILLGFNAADRGRAIET----WSD------EQIV----ASAM-QTLRTIYGV-------------------------------SIPEPIDYQITRWASDPFS-LGSYSY-NPVGA----------------V--PK-MRQ---ELAAPLE--------------------------KSVFFAGEASNE--DYFGTAHGAYLSGLRAAQEILEI-------------------------------------------------------------------------------------------------------------------------------------------------------------------------------------------------------------------------------------------------------------------------------------------------------------------------------------------------------------------------

>Bp_Et

------------------------------------------------------------------------------------------------------------MNRREMLRMMSAALATLW--LDAL-------------------------------------PTHAAERRPTRTKVLVI-------------------GAGLA---------GLACARTLQAQG-------F-A--------VQVVEAR-QRIGGRIWTSHAWP--------------------------EMPLDL-GATWIHGT----------------EKNPLTGI--A-EQ---IGARLLPTH-YEEA--------------------------------------------LVFAQDGRPLSA-------------KEERVLERLKS----VLFETLQE-GQ-----------------------S------APQDKSILATVADIVQDASPS--------------------------------ERLN-IWY-LL-NSN-LEQEL--SGALGEMST-------YYF--DD----D----------------------------WAFG-GEDALFPQGFSQITDHL--AQ---G------------------------------LTLALGQV----VSQIAYSTTG---------------------------------------------------VSVHT-------------------------------------------------------LQG---K-----VFQADRVVITLPLGVLQRGH--------------------------VTFAPALPA---DKLSAIQ-RLGMGTLNKCYLQFP--H-----IFWPDD----------------------------------------------------IDW---LE-Y-----------------------------------------------------------------------------------------------ISPQPGVWSEWVSFARA-----------------------------------------------------------------------AHWPVLLGFNAARQGVAMET----LSD------QQIV----ADAM-GVLQRLFGP-------------------------------TIPQPLRYQITRWSHDPYS-AGSYSY-YRTGS----------------T--PR-DRR---ALGKSVA--------------------------DRLYFAGEAVSR--RYYGTAHGALLSGLQAAQEIANH-------------------------------------------------------------------------------------------------------------------------------------------------------------------------------------------------------------------------------------------------------------------------------------------------------------------------------------------------------------------------

>Bg_Ha

----------------------------------------------------------------------------------------------------------MSYTRRDILKLAALVSLSPWLPACAPNDP----------NT-PTIKPTT--------------TSNSPVTSDSTPQVIVI-------------------GAGIA---------GLAAAAKLQANG-------Y-R--------VQIIEGR-DRIGGRIWTSRTWN--------------------------DMPVDL-GASWIHGV----------------TQNPLTDL--A-DT---ARIERTPTD-YENS--------------------------------------------LVYTMDGEELDD-------------AAVEQLEEQLVTLLDAVAEL-----------------------------V-----EDTDDMSLAAAMQQVLVEQAESI-------------------------------DQPR-LNF-SI-NST-IEHEY--AADVEELSA-------QYW--DN----D----------------------------GEVV-GGDVIFLDGYDQILDQL--TA---D------------------------------LTIHTGQP----VNAINYTAES---------------------------------------------------ITITT--------------------------------------------------------NT---T-----TFEAEHVIITVPLGVLKQGR--------------------------IQFTPPLDA---TKTDAIT-LLGSGLLNKTWLRFP--T-----AFWPKE----------------------------------------------------PEI---IN-Y-----------------------------------------------------------------------------------------------IDEQKGRWAEFLNIYHY-----------------------------------------------------------------------TDSPILLGFNAGSYARMLES----RSD------AEII----ADGM-QVLRTIYGQ-------------------------------EIPDPEAWQITRWGADPYA-FGSYSF-LGVGA----------------T--DA-LRD---DLAQPIA--------------------------GRLFFAGEATER--TYPSTVHGAYLSGLRAADEVMQA-------------------------------------------------------------------------------------------------------------------------------------------------------------------------------------------------------------------------------------------------------------------------------------------------------------------------------------------------------------------------

>Bcy_Oc

--------------------------------------------------------------------------------------------------------------------------------------------------------------------------------MSQSVDVLVI-------------------GAGIA---------GLAAASKLRAAG-------R-G--------VVVLEAR-DRIGGRIATDRTWN---------------------------VPIEL-GATWLHGT----------------EDNPLMAL--V-RQ---FNLKTQQTD-YDNY--------------------------------------------WLYDTKGKLVPD-------------NIQNELEDCLDDVLEELDALREH-LE-----------------------D-----GDEDDISLQDALEIVLSHWKLSL-------------------------S-----QRRE-LDY-AI-AAE-IEHEY--AADSCELSC-------YYW--DE----G----------------------------EQFE-GDDCLFPNGYDQLVEHL--AS---G------------------------------LDIRLQHI----VQQIAYSDVG---------------------------------------------------VEVQC--------------------------------------------------------DR---A-----TLQATHAVITLPLGVLKSDA--------------------------VTFSPALPT---RKQTAIR-RLGMGTLNKLVLLFP--S-----IFWQDE----------------------------------------------------AEV---LG-C-----------------------------------------------------------------------------------------------IPTTRGEWVEFYNLHPV-----------------------------------------------------------------------TGQPILVGFNAGNYARTVET----WTD------EETI----AAAM-QVLRRVYGA-------------------------------AVPAPLKALVTRWTADPFS-QGAYSF-IAKGA----------------S--PK-DIE---ALAKPVG--------------------------NRLFFAGEATSR--QYAATVHGALLSGWREADRINNLH------------------------------------------------------------------------------------------------------------------------------------------------------------------------------------------------------------------------------------------------------------------------------------------------------------------------------------------------------------------------

>Bg_Rc

-----------------------------------------------------------------------------------------------------------------MLKLLLSALTSASLPACA-GIP----------DP-PDALRPTDVPGSIHPSAPPAQSTADVNTDPAARDVIIV-------------------GAGIA---------GLRAAQTLQQHG-------R-R--------VLVLEGR-NRIGGRIWTDESTG---------------------------MPLDL-GASWIHGT----------------QGNPIATI--A-DQ---LNATLIATT-YDDV--------------------------------------------QRFDPTGNPLTN-------------NLNDR----IDALLERSFARARA-HA-----------------------E-----EQNSDISLQAALEAVLDQEPLDA-------------------------H-----DLRL-LNY-AI-NTV-FEHEY--AADSSQLSM-------RHF--DH----Q----------------------------KELN-GGDAIFGRGYRVIIDFL--AH---N------------------------------LDIRSGHI----VQRVAYADDG---------------------------------------------------VTVVT--------------------------------------------------------AH---G-----ALRAHAALITVPLGVLQRGG--------------------------IVFDPPLPS---SKQRAIE-RMGMGLLNKCYLIFP--E-----VFWGNT----------------------------------------------------T-L---LG-Y-----------------------------------------------------------------------------------------------VGERKGEWAEWLNLNTL-----------------------------------------------------------------------LGIPVLLGFNAATFARTIEA----QSD------ASII----QSAM-RTLRIIYGT-------------------------------DIPQPVDYRMTRWAADPFA-SGSYSF-LATGA----------------A--PN-DYD---TLAQPVG--------------------------KRLFFAGEHTHR--DYPATVHGAYLSGERAANEMLSTNDA----------------------------------------------------------------------------------------------------------------------------------------------------------------------------------------------------------------------------------------------------------------------------------------------------------------------------------------------------------------------

>Ct-Te

-----------------------------------------------------------------------------------------------------------MRCSRIAVGRINYLVRVQA---------------------------PIALP----TWQRSTRACTTVCANMQKKKVVVV-------------------GAGFA---------GITAARTLLAEA-----AAPLE--------VVVLEAS-SRIGGRAHTMELEGC--------------------------GKVEL-GATWFHGIV----------------GNPLYEH--A-IQ---LGLMTRHEA-EDS------------------KSPWG--------------------AMKYVRQNESALLGKED---------AAFITKARDMYGEAVEASTEAAL----------------------------------NGQAGVIGDSVRHAFAQAMQSM----------------------E-GASARSKEIFTEAWAWRE-QLQ-RAMDG--CHTTDDMSA-------NSL--AQ----Y----------------------------DEFE-GPNIPLPSGYQAYAMIF--IQRRVAVIMFSAARNTMHDAQVL----AEKLAEG--LPIRHGMT----VDGIQWNSR---------------------------------------------------GVIVQC-------------------------------------------------------QGG---Q-----QIEADAVIVTVSLGVLKAQH-------------------------KCMFQPELPT---ATVEAIE-RLGFGVVDKIYIDFG--A-----ASTAGPAAAQQHFSNYGPAGGSNADQSKPAQHDSQGSVKEHANNPVQDDSMHPLTAKDALSYYLLWHCNPEDFQPKLSADSRHEEEKQTVAANVSRDPLRDIKGMNNNGNGRQGDNSAGSPAAKGAGDKGPETHVITGPQAEGTDSTEAKSNAEADSIESTKGRQHDLPAWAHGAYTMRFAGSEFVADKPTQ--------------------------------------------------------HLAAANRCGVMWMTGEDAKKMEA----ASD------AELQ----QHIA-AVLQEFPAMALP-------------------------------SDFKVYRSCWGSDPLF-RGSYSY-GSASA----------------V--GG-ECS---VLAEPLCAPES--------------------SALRLLFAGEACHS--KYFGCTHGAYLTGQSQARALMKSLDICGAVSVAHSSNV-------------------------------------V--------------------------------------------------------------------------------------------------------------------------------------------------------------------------------------------------------------------------------------------------------------------------------------------------------------------

>Ct-Ap

-----------------------------------------------------------------------------------------------------------------------------------------------------------------------------------MSGVLIV-------------------GAGFA---------GLAAAQTLRRAG-------MSP--------VILLEAG-SHVGGRARTLRLDRG--------------------------LALEM-GATWIHGLEL----------GGGEGPNPVLRA--A-QR---AQLLGARP----R------------------LSVWE--------------------DSHFLVQGASGLLTQEQ---------AACIMHSIAAFEEGLDAAGGA-------------------------------------EEGATLGDVLQAAWDKLSSGQ----------------------KV---AQHADLARRVWAWRE-SLQ-RAIDG--TDCSGDLSA-------TAA--AR----Y----------------------------AAAVQNTNAPIPGGFQAVAEAM--AEG---------------------------------LDVRLHRR----VTGLDWGAE--------------------------------------------------GGACATC-------------------------------------------------------EDG---S-----RHAAAAAIVTVSLGVLKAQH-------------------------ETLFQPGLPA---RTRKAMA-QLRMGTVDKLFLEFY--D-----AE----DSALDREDGAAPAAAP-----------DLGN------------------GADVVTYALLWDPASTA------------------------------------------------------------------------------------------SADSALPDWARGVFSIGFGGAEVKQGAQEP---------------------------------------------------------GASPRPVGVVWLAGEAARAVEA----ASD------EEVL----GTLR-AVFRAFPGAVLPA--G------------------------ASWRRVRLHRSAWGSDPLF-RGSYSY-PGPSA----------------A--AD-AGA---VLGEALTPPGV--------------------ARPTLVLAGEACAV--EYFGTTHGAMRSGEAAAAKLLSSL------------------------------------------------------------------------------------------------------------------------------------------------------------------------------------------------------------------------------------------------------------------------------------------------------------------------------------------------------------------------

>Ct-Mc

--------------------------------------------------------------------------------------------------------------------------MSSR---------------------------GAPAT----AALRTAAAAAAAAAAPPRPRVAVI-------------------GAGFA---------GLEAALTLQTAG-------QCD--------VMLLEAG-ARPGGRAWTLPLPAG-----------------------SAAAALEL-GATWLHGLG------------SEGEPNPVFRH--A-VE---LGLIESNP----T------------------AERWW--------------------SSQFCLPGEARPLSLEE---------QAVITHALAAWGEAVEGLQQD--------------------------------------EAGTTADALRAAWAALLASG----------------------RLGDAGERLQLAGRSWRWRE-LLQ-RAMDG--CDSTSVQSA-------QGL--AL----Y----------------------------DEMPGGVHAAMPGGMQGVAEGL--AARV--------------------------------HDLRYGHA----VQCIRWGGAGH-----------------------------------------------AGPVSIAC-------------------------------------------------------ANG---A-----AVEADAVVVTVSLGVLKAQH-------------------------KALFEPALPL---TKQDAIQ-RLSIGTVDKLLLDLA--P-----GS----ASSSSGSGSGGGSGGGGSGDGGSVSGGRASG------------------SAEAVSFALLWSEPWQGFGGSTSA--------------------------------------------------------------------SASACVDSPPAAAPAPGEAQLPGWARGVFSIRFGGPEFKQRSAAS---------AAAGVAQQ-------AQQAAAGDAGRTGEAA---EGHQEEFNPRAQ-AQQPRTYQAVAWLTGAEAAAMEA----ASD------EEVL----GTLR-RLAEVFPALQLPP--G------------------------ASWDRVQLHRSRWGSDPLF-RGSYSY-LGPGS----------------S--PA-DVA---ALQAPVEGPDG--------------------GAPRVLFAGEACHV--KYIGTMHGAALTGRLAAQTLLQHWSEAPAGQQRERHEA-------------------------------------SPG------------------------------------------------------------------------------------------------------------------------------------------------------------------------------------------------------------------------------------------------------------------------------------------------------------------

>Ct-Cs1

---------------------------------------------------------------------------------------------------------------------MAAPLRTAV---------------------------APLRA----AATAATAATMSAAAAPARPRVAVI-------------------GAGFA---------GLAAALELQRSG-------RCD--------VTVLEAS-GRPGGRASTMQLPSG--------------------------TALEI-GATWFHGLG------------DEDEPNPVFQQ--A-LE---AGLIDANP----E------------------AEQWW--------------------SSQFLLPCATQPLSKEQ---------QVAVTHTLAAWGEAIDDLPPD--------------------------------------APGTTADHLRRAGEQLLASG----------------------KLGE--EHLELAERVWRWRE-QLQ-RAMDG--CHSTSEQSA-------QGH--AL----Y----------------------------DEMPGGVNCPLPAGMQAVAEHM--AAKV--------------------------------ADLRFQHA----VQRIEWGSAPSGTSNS-------------------------------SGGSGSGSGSCAARVRIAC-------------------------------------------------------SNG---D-----SLEADAAIVTVSLGVLQARH-------------------------HQLFSPALPP---QKVAAIE-RLRIGCVNKFFLDFG--A-----PE----DVAGSGS----SAGGGESPTS---------G------------------SSPAVSYSLLWSEAWEGASSSDTF-----------------------------------------------------------------------------TAAVPSAVEAQLPSWAKGIFSIRFGGPETKRPQLAGKSPDGHAAAAGAADTAP-------AAAGSSSGAPDNNEEE---EEEAQFIVPSAE-PEQPRCYQGVAWVSGEAAVEMEA----ASD------EEVL----CTLR-QLAGLFPQLQLPP--G------------------------ASWDRVRLYRSRWGTDPLF-RGSYSY-IPAGG----------------S--PA-DVA---ALAAPICLPAGPHSAAAVSAAGGEAAAPAAGSTPVLLFAGEACHV--KYIGTMHGAAITGQAAAAQLLAAWQHTE--------------------------------------------------------------------------------------------------------------------------------------------------------------------------------------------------------------------------------------------------------------------------------------------------------------------------------------------------------------------

>Ct-Cv3

------------------------------------------------------------------------------------------------------------------------------------------------------------MA----ATAAGPAAAPPLAGTARRPRVAVI-------------------GAGFA---------GLAAALTLERSR-------CCD--------VVVLEAS-GRTGGRACTQRLSPE--------------------------LALEL-GATWFHGLG------------SDGQPNPVFRH--A-VQ---QGLIGSAP----E------------------GEKWW--------------------SSQFLLPGSSQPLSKAQ---------QIAVTHAIAAWAEAVDELPRD--------------------------------------AAGTTADHLRVAWRRLLASG----------------------RAPE--ELEELAQRAWRWRE-HLQ-RAMDG--CNSTAEQSA-------QGL--AL----Y----------------------------EEMAGGVHAPLPAGMQAVAEGL--AAQL--------------------------------GDVRLGHA----VSRITWGRP--------------------------------------------------GGVTIAC-------------------------------------------------------RNG---A-----TVEADAAIVTVSLGVLKAQH-------------------------GTLFDPPLPP---AKQAALE-RLQIGTVDKLFLDFT--P-----PG----MPAGTSSTRQSSSSGSSSSGG---------S------------------EGPVVSYALLWAGPWDAAASGKEH--------------------------------------------------------------------A---AGSRAAAPATSEDEAQLPEWARGVFSLRFGGPEVKRCQQAADQQQPVG-AHGVGDGTE-------QQQQQQQQQHELQPEE---EPAESEFSPCSE-AAQPTCYQAVAWVTGAAAAAMEA----ASD------EQVL----AALR-QLATLFPQLQLPP--G------------------------ASWDAVELHRSRWGSDPLF-RGSYSY-LGAGS----------------T--PA-DVA---ALAAPLFAPAGGSGGAEA----SGGAEASGGAAPVLMFAGEACHV--KHIGTMHGAYLTGRHQAELLAAALAPEAVELPQQQQQP-------------------------------------PM-------------------------------------------------------------------------------------------------------------------------------------------------------------------------------------------------------------------------------------------------------------------------------------------------------------------

>Rp-Pop1

-----------------------------------------------------------------------------------------------------------------------------------------------------------------------------------MVRVCIV-------------------GAGVA---------GCTAASALHHALG---SSGALE--------LVVLEAS-ARVGGRVKHAGAGLR--------------------------CGGEL-GATWVHGK----------------RGNPIYPI--V-HA---ATNGST-----------------------LQQQSWQ---------------------MPVAIYHGRRVDP-------------QFVAHVLEDVEAIIDRAVSKGA---------------------------------GIQGVRSVGEFLDAEWKAQGQRD------------------DHEGEHESSETSHVLKDCIYQRRK-RLE-CSISA--CDSLNDLRL-------DQY--RE----Y----------------------------DRLD-DNHSRNSAPMETFLHGM--LAPLLP------------------------------ETVRLETR----VTQVQRCGAESQD---------------------------------------------GAPLLVKA-------------------------------------------------------WDA---RAGASVSYLADFCIFTASLGVMKAEG-------------------------ERIFEPPLPP---KKLQAIR-RLGFGTVDKVLIEFE--H-----ESDPVK-------------------------------------------------GELPQSAFLLGTPASAEEGH---------------------------------------------------------------------------------------APDTDKYAWLFDYAPLVYMDRPA------------------------------------------------------------------SGFLRGEMWLSGTTAREMEA----KDD------EHVF----RAVQ-AYLHALAPLAY-------------------------------PKVRSIARSRWFSDPNF-GGSYSY-NAIES----------------D--GS-DFE---TLAEPLPCLP-----------------PHDVNRPCILFAGEATHR--SFYSTMHGAFASGEREAKRLLGLLARTADVAN----------------------------------------------------------------------------------------------------------------------------------------------------------------------------------------------------------------------------------------------------------------------------------------------------------------------------------------------------------------

>Pl_Sm5

---------------------------------------------------------------------------------------------------------------------------------------------------------------------------MELKKLCGKPRVIVI-------------------GAGIS---------GLSAARRLYSSPA---AARDWQ--------ITVLEAS-DRIGGRIFTSQFDTG--------------------------EQIEI-GATWIHGV----------------EGSPIFDI--A-EK---SGALHGDVP-FECMDGFP-----------------E--------------------PPIVKAQGGVTVHS-------------TIAHDVASLYRQLVDDVNDRRGEPPE-----------------------VTAETLEHGDSGNLGSYLRRGFESFLGKQAATPA--GVNAAELLLKQDNPSIASSGWNLRALQEGVFTIQE-NWE-RCVTA--AESLHDLDL-------LAF--NE----Y----------------------------WEFP-GEQITIGKGFSSVVQAL--AKSLPP------------------------------DTIRFHKK----VDKVVWTDVARTSA------------------------------------------SSGYPVQLHC-------------------------------------------------------EDG---S-----TFEADHVIVTVSLGVLKAKALEE----------------------QQLFQPRLPD---WKLDSIE-KLGFGVVDKLFVLVE--P-----PPDGS----------------------------------------------------QHPNLQFIHKSQADADE-------------------------------------------------------------------------------------------DEVPRWMRKTHSLY---PIH------------------------------------------------------------------KKSNVLVAWFAGAEAKEMEK----LSD------EEIA----RGVQ-KTLAAFGDKRRVA--GLGSQRQHCCNGG--DAS---SNGGTHSGKVHVAHGCWNRNPLF-LGSYSY-VAVGS----------------N--GD-DID---HLAAPVPRLS------------------DSGPPLQLLFAGEATHR--DQYSTTHGAYFSGQREADRLIQHYKFAS--------------------------------------------------------------------------------------------------------------------------------------------------------------------------------------------------------------------------------------------------------------------------------------------------------------------------------------------------------------------

>Pl_Sm6

---------------------------------------------------------------------------------------------------------------------------------------------------------------------------MELKKLCGKPRVIVI-------------------GAGIS---------GLSAARRLYSSPA---AARDWQ--------ITVLEAS-DRIGGRIFTSQFDTG--------------------------EQIEI-GATWIHGV----------------EGSPIFDI--A-EK---SRALHGDVP-FECMDGFP-----------------E--------------------PPIVKAQGGVTVHS-------------TIAHDVASLYRQLVDDVNDRRGEPPE-----------------------VTAETLEHGDSGNLGSYLRRGFESFLAKQAATPA--GVNAAELLLKQDNPSIASSGWNLRALQEGVFTIQE-NWE-RCVTA--AESLHDLDL-------LAF--NE----Y----------------------------WEFP-GEQITIGKGFSSVVQAL--AKSLPP------------------------------DTIRFHKK----VDRVVWTDVARTSA------------------------------------------SSGYPVQLHC-------------------------------------------------------EDG---S-----TFEADHVIVTVSLGVLKAKALEE----------------------QQLFQPRLPD---WKLDSIE-KLGFGVVDKLFVLVE--P-----PPDGS----------------------------------------------------QHPNLQFIHKSQADADE-------------------------------------------------------------------------------------------DEVPRWMRKTHSLY---PIH------------------------------------------------------------------KKSNVLVAWFAGAEAKEMEK----LSD------EEIA----RGVQ-KTLAAFGDKRRVA--GLGSQRQHCCNGG--DAS---SNGGTHSGKVHVAHGCWNRNPLF-LGSYSY-VAVGS----------------N--GD-DID---HLAAPVPRLS------------------DSGPPLQLLFAGEATHR--DQYSTTHGAYFSGQREADRLIQHYKVAS--------------------------------------------------------------------------------------------------------------------------------------------------------------------------------------------------------------------------------------------------------------------------------------------------------------------------------------------------------------------

>Rc-Gs

-----------------------------------------------------------------------------------------------------------------------------------------------------------------------------------MPQVIVV-------------------GAGIA---------GITAASALHAA--------NVQ--------VCILEAS-HRIGGRVCTVSP------------------------------GMEL-GATWIHGT----------------VNNPIYDL--A-VV---RGLVEKYPS-PEDKAEPNEE--------ELTSWKLA--------------------ECPFIREGGTFVET-------------YVVKDALEKFGRYRNEIFHWPTLQV------------------------DA-----KQYNDSIEEYLSKRWKQDHLET--------------------------GMTPSEAQRLVFQWRK-RLE-CSISA--CSSLSELSL-------EYL--HE----Y----------------------------CELA-GENVEVLCGFSKIVESL--LAGFPS------------------------------ENILFGRE----VTRIRWGGSD-----------------------------------------------RNNRVSIEC-------------------------------------------------------SNS---E-----VFTAEYLIWTGSLGVLQERE-------------------------SNLFDPPLPR---KKKDAIH-RLALGTVDKVFVEFD--R-----QPLQHQ-------------------------------------------------GKQWDYVSLLWNESLEREE---------------------------------------------------------------------------------------------PSHWTKKIFSFRAV------------------------------------------------------------------------NNILSFWLTGASAKQMEQ----ESD------DAIL----QHTK-LLLSRFG--LV------------------------------EAEPIRVIRSSWYSNPLF-RGSYSF-VPVGA----------------S--GS-DFE---ILAEPVNLPEL----------GLETSDSHRIYNPCLFFAGEATHR--KFYSTTHGAYLSGCREAKRILELEGIKSQTLKTN--------------------------------------------------------------------------------------------------------------------------------------------------------------------------------------------------------------------------------------------------------------------------------------------------------------------------------------------------------------

>Mi_Am

----------------------------------------------------------------------------------------------------------------------------------------------------------------------MAESTKKTEDDKVKCKILIV-------------------GAGMA---------GLSAANHLLKN-H------ETD--------FLIVEAR-GRIGGRIVATKIG-N--------------------------EKVEL-GANWIHGV----------------LGNPMFEL--A-MA---NGLIDIIRV-PRPHK------------------------------------------VVAAMEDGKQLPF-------------PILQEIYEAYVCFLRRCEEYFLSTY------------------------SP-----PDGINSVGAHVALEAEIYLSTL--------------------------LPEERKIRQLLFDCLL-KRE-TCITG--CDSMENVDL-------LEM--GS----Y----------------------------AELQ-GGNISLPDGYSAILEPV--SKHIPK------------------------------SSILTKHV----VTKISSNTN--------------------------------------------------SSIEIQC-------------------------------------------------------ENG---K-----TILAEHVICTLPLGVLKEKA-------------------------NDIFEPPLPN---YKFEAIN-RLLFGTVDKIFLEYE--R-----PFLNP----------------------------------------------------GVSEVMLLWDDRGLSEEE----------------------------------------------------------------------------------------KQDISKTWFRKIYSFTKI-----------------------------------------------------------------------SETLLLGWISGKAAEYMEK----LSG------AEVA----EICT-SILRKFLNDPF------------------------------VPAPKNCLRTSWHSQPYT-RGSYTA-MAVGA----------------S--QL-DIK---YLSEPIVQED-------------------DPSKIIITFAGEHTHS--SFYSTVHGAYLTGRTAAQALLESRKNEKNSLSLSCEDT-------------------------------------SDLSSWIQGISLN--------------------------------------------------------------------------------------------------------------------------------------------------------------------------------------------------------------------------------------------------------------------------------------------------------

>Mi_Dm

------------------------------------------------------------------------------------------------------------------MGDKEEPVTPAP---------------------------SPTEEGMANIGSAGAGDQPQTSGNNTNVKIVII-------------------GAGMA---------GLSAANHLLQN-G------CDD--------FLILEAR-GRVGGRIVSIPLSNN--------------------------QKIEL-GANWIHGV----------------LGNPIFEL--A-VQ---HGLVSVVNV-PKPHK------------------------------------------VVATTEDGHQVPF-------------NILQEIYEAYVCFLRRCDEYFLCQY------------------------SP-----PPDIHSVGEHINYEIEIYLSGV-----------------------Q--DPKEKRLKQSIFNCLL-KRE-TCITG--CNNMDEVDL-------LEL--GS----Y----------------------------TELQ-GGNIVLPTGYSSILRPL--GAQIAK------------------------------QSILTKCP----VKKIHWKRKKTFTGLETVDENSEDEHSDDSERTVTEVPTGEIRGASVESNTSSNCDYPAGNVRIDC-------------------------------------------------------EDG---R-----VFHAAHVICTIPLGVLKNTH-------------------------RTLFDPVLPQ---YKQESIE-NLMFGTVDKIFLEYE--R-----PFLSA----------------------------------------------------DISEIMLLWDDDKRDMNSSEE---------------------------------------------------------------------------------ELASEAYLSKNWFKKIYSFAKV-----------------------------------------------------------------------TDTLLLGWVSGREAEYMEK----LDH------EAVA----EKCT-EILRNFLQDPY------------------------------VPKPKRCVCTSWKSQDFT-GGAYTS-IPVGA----------------T--QE-DIE---NLAQPLYATP-------------------QAMKPAIVFAGEHTHS--SFYSTVHGAYLSGRTAAQHLLASDEPDEIIM---ESDG-------------------------------------SDLSAWIQGIALD--------------------------------------------------------------------------------------------------------------------------------------------------------------------------------------------------------------------------------------------------------------------------------------------------------

>Mc_Bf

--------------------------------------------------------------------------------------------------------------------------------------------------------------------------MSAGLSSSLAPHVVVV-------------------GGGMA---------GVAAAQRLVQE-G------LTH--------VKILEAR-DRVGGRIWTQYLGSD--------------------------TTLEL-GANWIHGS----------------IGNPIYEL--A-KQ---HGLLRDEVK-PDDLDRPT-----------VG--ELK--------------------NGKFLMPGGKVMDE-------------AVVDSFLQNYNEMIEECCAVFEQGK-------------------------A-----SQPVDSIGEFLSREFGKQLVSS-----------------------NDTDASVKCTKMALLQHFL-KYE-TCDNG--CHDMREVSL-------KYF--GQ----Y----------------------------NELE-G-DHNNTSDFSAILDLV--LKTIPP------------------------------DCIAFNKK----VQCIRWKEEGQKRSDS-------------------------------------AHAYDTHGVEVEC-------------------------------------------------------EDG---Q-----VFSADHVIVTVPLGFLKKNS-------------------------RTLFQPPLPE---EKLASIE-RMGFGVVNKIFLTFQ--E-----PFWDT----------------------------------------------------EYDALHLVWDQDESN--------------------------------------------------------------------------------------------PKTPEEWYKKTYCFYID---S------------------------------------------------------------------KAPKTLMGFISGKEAEYMET----LSE------EEIS----NTFL-SLLKKFTGKDD------------------------------IPKPVRTMITRWGSDALT-CGSYSY-IHVGE----------------K--GD-DIS---TVAEPLYRDN--------------------TEVPAVQFAGEATHS--EFFSTVHGAYLSGQREANRLVNLYGNSENGTRKS--------------------------------------------------------------------------------------------------------------------------------------------------------------------------------------------------------------------------------------------------------------------------------------------------------------------------------------------------------------

>Mac_DrS

------------------------------------------------------------------------------------------------------------------------------------------------------------MQSCEISSDSTDDPLSSALHGHRQPRIVVI-------------------GAGLA---------GLAATKTLLEN-G------FTN--------VTVLEAS-DRIGR-VQSIQHGK---------------------------TTLEL-GATWIHGA----------------NGNPVYHL--A-ED---NGLLEHTTE-EERSVGRI-----------SLYAKNG--------------------V-HYQTNNGKRIPK-------------DLVEEFSDLYNEVYELTQEFFQNGKP-----------------------VG-----AESQNSVGIFTRDVVRKKIL-D-----------------------PYDSESIRKLKLSMLQQYL-KVE-SCESS--SPNMDEVSL-------SEF--GE----W----------------------------TEIP-GAHHVIPTGFIV-VEIL--AQDIPS------------------------------CVLHLSKP----VRRVHWNCSSQDAEEF----GDQVDHNQD---------Q----------------RPSPSPVCVEC-------------------------------------------------------EDG---R------LLADHVILTASLGVLKKAH-------------------------KTLFSPGLPQ---DKAQAIQ-KLGISTTDKIFLEFA--E-----PFWSP----------------------------------------------------E-NSIQFVWEDEAQLESQ-----------------------------------------------------------------------------------------AYPEELWYRKICSFDVLYPPE------------------------------------------------------------------RYGHMLSGWICGEEALRMER----CDE-------TVA----EICT-ELLRQFTGNQN------------------------------IPKPRRILRSSWGSNPYI-RGSYSF-TRVGS----------------S--GR-DVE---KLAEPLPYIK-----------------NTKAPPFQVLFAGEATHR--KYYSTTHGALLSGQREANRLMELYQYSCAETTKPNI------------------------------------------------------------------------------------------------------------------------------------------------------------------------------------------------------------------------------------------------------------------------------------------------------------------------------------------------------------

>Ma_XlS

------------------------------------------------------------------------------------------------------------------------------------------------------------MQSCEISSDGTDDPLSSGSRRHRQPRIVII-------------------GAGLA---------GLSAAKTLLEK-G------FTD--------VTILEAS-DRIGGRVQSIKLEN---------------------------STFEL-GATWIHGS----------------DGNPIYHL--A-ED---NGLLEETTD-GERSVGRI-----------SLYSKNG--------------------VAHYLTNGGHRIPK-------------DLVEEFSDVYNEVYNLTQEFFQNGKP-----------------------VN-----AESQNSVGVFTRDVVRKRIKED-----------------------PDDSENTKKLKLAMVQQFL-KVE-SCESS--SHSMDEVSL-------SEF--GE----W----------------------------TEIP-GAHHVIPCGFIRIVEIL--SSSVPA------------------------------SLIQLRKP----VKCVHWNRSVRKQ------IDQVADHNND---------QV---------------EDKGFPVFVEC-------------------------------------------------------EDY---E-----FIAADHVIVTASLGVMKKFH-------------------------ETLFHPSLPE---EKVTAIE-KLGISTTDKIFLEFE--E-----PFWSP----------------------------------------------------ECNSLQFVWEDEAESESL-----------------------------------------------------------------------------------------TYPEEMWYKKICSFDVLYPPE------------------------------------------------------------------RYGYVLSGWICGEEALIMEK----YDD------ETVA----ETCT-ELLRKFTGNPN------------------------------IPKPRRILRSSWGSNPYF-FGSYSY-TQVGS----------------S--GA-DVE---KLAKPLPYTE-----------------SSKTAPLQVMFSGEATHR--KYYSTTHGALLSGQREAERLSEMYQDLLQRQK----------------------------------------------------------------------------------------------------------------------------------------------------------------------------------------------------------------------------------------------------------------------------------------------------------------------------------------------------------------

>Mm_MeS

------------------------------------------------------------------------------------------------------------------------------------------------------------MQSCETSGHSADDPLSRGLPRRRQPRVVVI-------------------GAGLA---------GLAAAKALLEH-G------FTD--------VIVLEAS-DRIGGRVQSVKLEH---------------------------ATFEL-GATWIHGS----------------HGNPIYHL--A-EE---NGLLEETTD-AERSVGRI-----------SLYSKDG--------------------VAYYLTNRGQRIPK-------------DVVEEFSDLYNEVYNLTQEFFQRGKP-----------------------VN-----AESQNSVGVFTREEVRNRIKAD-----------------------PDDSETTKRLKLAMIQQYL-KVE-SCESS--SHSMDEVSL-------SAF--GE----W----------------------------TEIP-GAHHVIPCGFMRIVELL--AHDIPD------------------------------RVIQLGKA----VRCVHWDQASARREGPEIEQLANHN--ND-----------TSDEGGWGRQERRRGEGSRWPVNVEC-------------------------------------------------------EDC---E-----VIPADHVIVTMSLGVLKKHH-------------------------STLFRPPLPS---EKAGAIR-RLGISTTDKIFLEFE--E-----PFWGP----------------------------------------------------ECNRLQFVWEDEAESRSL-----------------------------------------------------------------------------------------TYPEELWYRKICGFDVLYPPE------------------------------------------------------------------RYGHVLSGWICGEEALVMER----CDD------EAVA----EICT-EMLRKFTGNPD------------------------------IPKPRRILRSSWGSNPFF-RGSYSY-TQVGS----------------S--GA-DVE---RLAKPLPYTE-----------------SSKTAPMQVLFSGEATHR--KYYSTTHGALLSGQREAARLIEMYQDLFQRGT----------------------------------------------------------------------------------------------------------------------------------------------------------------------------------------------------------------------------------------------------------------------------------------------------------------------------------------------------------------

>Mm_MmS

------------------------------------------------------------------------------------------------------------------------------------------------------------MQSCESSGDSADDPLSRGLRRRGQPRVVVI-------------------GAGLA---------GLAAARALLEQ-G------FTD--------VTVLEAS-SHIGGRVQSVRLGD---------------------------TTFEL-GATWIHGS----------------HGNPIYQL--A-EA---NGLLEETTD-GERSVGRI-----------SLYSKNG--------------------VACYLTNRGCRIPK-------------DVVEEFSDLYNEVYNMTQEFFRHGKP-----------------------VN-----AESQNSVGVFTREKVRNRIRDD-----------------------PDDTEATKRLKLAMIQQYL-KVE-SCESS--SHSIDEVSL-------SAF--GE----W----------------------------TEIP-GAHHIIPSGFMRVVELL--AEGIPP------------------------------HVIQLGKP----VRCIHWDQASAHPRGPEIEPRGEGDHNHD---------TGEGGQSGENPQQGRWDEDEPWPVVVEC-------------------------------------------------------EDC---E-----VIPADHVIVTVSLGVLKRQY-------------------------TSFFRPCLPT---EKVAAIH-RLGIGTTDKIFLEFE--E-----PFWGP----------------------------------------------------ECNSLQFVWEDEAESCTL-----------------------------------------------------------------------------------------TYPPELWYRKICGFDVLYPPE------------------------------------------------------------------RYGHVLSGWICGEEALVMER----CDD------EAVA----EICT-EMLRQFTGNPN------------------------------IPKPRRILRSAWGSNPYF-RGSYSY-TQVGS----------------S--GA-DVE---KLAKPLPYTE-----------------SSKTAPMQVLFSGEATHR--KYYSTTHGALLSGQREAARLIEMYRDLFQQGP----------------------------------------------------------------------------------------------------------------------------------------------------------------------------------------------------------------------------------------------------------------------------------------------------------------------------------------------------------------

>Mm_EqS

------------------------------------------------------------------------------------------------------------------------------------------------------------MQSCESSGDSADDPLSRGLRRRGQPRVVVI-------------------GAGLA---------GLAAAKALLEQ-G------FTD--------VTVLEAS-SRIGGRVQSVKLGH---------------------------STFEL-GATWIHGS----------------HGNPIYHL--A-EA---NGLLEETTD-GERSVGRI-----------SRYSKNG--------------------VACYLTNRGRRIPK-------------DVVEEFSDLYNEVYNLTQEFFRHGKP-----------------------VN-----AESQNSVGVFTREEVRNRIRDD-----------------------PDDPEATKRLKLAMIQQYL-KVE-SCESS--SHSIDEVSL-------SAF--GE----W----------------------------TEIP-GAHHIIPSGFMRVVELL--AEGIPA------------------------------HVIQLGKP----VRCVHWDQASARPRGPEIEPRGEGDHNHD---------TGEGSQGGEEPRGSGREEDEQWPVVVEC-------------------------------------------------------EDC---E-----VIPADHVIVTVSLGVLKRQH-------------------------ASFFRPGLPV---EKVAAIH-RLGIGTTDKIFLEFE--E-----PFWGP----------------------------------------------------ECNSLQFVWEDEAESRTL-----------------------------------------------------------------------------------------TYPPELWYRKICGFDVLYPPE------------------------------------------------------------------RYGHVLSGWICGEEALVMEK----CDD------EAVA----EICT-EMLRQFTGNPN------------------------------IPKPRRILRSAWGSDPYF-RGSYSY-TQVGS----------------S--GA-DVE---KLAKPLPYTE-----------------SSKTAPMQVLFSGEATHR--KYYSTTHGALLSGQREAARLIEMYRDLFQQGT----------------------------------------------------------------------------------------------------------------------------------------------------------------------------------------------------------------------------------------------------------------------------------------------------------------------------------------------------------------

>Mm_HsS

------------------------------------------------------------------------------------------------------------------------------------------------------------MQSCESSGDSADDPLSRGLRRRGQPRVVVI-------------------GAGLA---------GLAAAKALLEQ-G------FTD--------VTVLEAS-SHIGGRVQSVKLGH---------------------------ATFEL-GATWIHGS----------------HGNPIYHL--A-EA---NGLLEETTD-GERSVGRI-----------SLYSKNG--------------------VACYLTNHGRRIPK-------------DVVEEFSDLYNEVYNLTQEFFRHDKP-----------------------VN-----AESQNSVGVFTREEVRNRIRND-----------------------PDDPEATKRLKLAMIQQYL-KVE-SCESS--SHSMDEVSL-------SAF--GE----W----------------------------TEIP-GAHHIIPSGFMRVVELL--AEGIPA------------------------------HVIQLGKP----VRCIHWDQASARPRGPEIEPRGEGDHNHD---------TGEGGQGGEEPRGGRWDEDEQWSVVVEC-------------------------------------------------------EDR---E-----LIPADHVIVTVSLGVLKRQY-------------------------TSFFRPGLPT---EKVAAIH-RLGIGTTDKIFLEFE--E-----PFWGP----------------------------------------------------ECNSLQFVWEDEAESHTL-----------------------------------------------------------------------------------------TYPPELWYRKICGFDVLYPPE------------------------------------------------------------------RYGHVLSGWICGEEALVMEK----CDD------EAVA----EICT-EMLRQFTGNPN------------------------------IPKPRRILRSAWGSNPYF-RGSYSY-TQVGS----------------S--GA-DVE---KLAKPLPYTE-----------------SSKTAPMQVLFSGEATHR--KYYSTTHGALLSGQREAARLIEMYRDLFQQGT----------------------------------------------------------------------------------------------------------------------------------------------------------------------------------------------------------------------------------------------------------------------------------------------------------------------------------------------------------------

>Mac_DrA

-------------------------------------------------------------------------------------------------------------------------------------------------------------------------MDSMAQRPDRDSQIFII-------------------GCGIS---------GIGAAQKLIKH-G------FHN--------VRIIEAT-ARSGGRIRTGRLGD---------------------------NIIEI-GANWIHGPS---------------KENPVFRL--A-CD---YQLLDKESM-SEENQAID-----------IGGHPLF--------------------VPNWFTSSGRKLGP-------------ETMGPALEFFMTLLERSQQFHSTGG--------------------------------EPLPSVGEFIKAEAERLAPEE-----------------------WKEDRDNFAVRMAMINTLL-KLE-CCVSG--THTMDDVGL-------GAF--GM----Y----------------------------TTLP-GLDCTFPGGYEGLTDHM--MKELPR------------------------------DIVLYNKP----VKCIHWNYTKNGPNT----------------------------------------GGTSFPVTIEC-------------------------------------------------------VNG---E-----TFAADHVIVTVPLGYMKKHQ-------------------------NTFLSPSFPL---HKLHSIQ-RMGFGTNNKIFVEFE--Q-----PFWDE----------------------------------------------------DCELIYLVWEDETHLTDV----------------------------------------------------------------------------------------VSDLKMSWIRKLTGFTVLKPTE------------------------------------------------------------------RFGHVLCGWIAGQESEYMES----LSE------LEVL----QTVT-QLLRIFTGNPT-------------------------------IMPRKLLRSQWFHEPYS-CGSYSY-VAKGC----------------S--GY-DID---NLAEPLPLKG------------------SNSKPLQVLFAGEATHR--SFFSTVHGALLSGWREAERLISHHTSASGSFSSKL-------------------------------------------------------------------------------------------------------------------------------------------------------------------------------------------------------------------------------------------------------------------------------------------------------------------------------------------------------------

>Ma_XlA

-------------------------------------------------------------------------------------------------------------------------------------------------------------------------------MDPMGPVVLII-------------------GAGIS---------GLAAAQKLYKH-G------FKN--------LRILEAT-GRSGGRIRSQKYAK---------------------------GLVEI-GAQWIHGPS---------------PSNPVFQL--S-TQ---YDLLSPEAL-SEENQLVE-----------LEGHPMF---------------------SVIYSSSGKQIST-------------EIGENVVEMFSSWFQKSREFTKGGC--------------------------------NPEDSVGSFLRQEISCSYSNW-----------------------DKD---SLELKMALLNCLF-KLE-CCISG--THSMDCVAL-------GPY--GE----Y----------------------------KILP-GLDCTFPRGYESLVSHI--KASFPS------------------------------DMVLLNKP----VKTIHWKGSFHGSD------------------------------------------SHMYPVQVEC-------------------------------------------------------ENG---E-----TFIADHVIITVPLGFLKEKA-------------------------TDLLSPPLPS---YKLQAIQ-NLGFGTNNKILLEFE--K-----PFWEP----------------------------------------------------ECYAIQLIWEGESPLTEP----------------------------------------------------------------------------------------KTNLQQDWVKKIPGFVVLQPPE------------------------------------------------------------------QLGHVLCAFIAGKESEFMES----LSE------DEIL----STMT-SLLRKCTGTPN------------------------------LPPPISILRTRWHSEPYT-CGSYSY-VAVGS----------------S--GR-DID---MLAQPLPEER------------------ECAKPLQVLFAGEATHR--NFYSTTHGALLSGWREAERLIDQYPALHSVFSKSKL------------------------------------------------------------------------------------------------------------------------------------------------------------------------------------------------------------------------------------------------------------------------------------------------------------------------------------------------------------

>Mm_MeA

------------------------------------------------------------------------------------------------------------------------------------------------------------------------MEKGGGRGALAEPLVVVI-------------------GGGIA---------GLGAAQRLCRHSS------FRN--------LLLLEAT-DRCGGRIRSQAAFG---------------------------SVIEI-GAHWIHGPS---------------KSNPVFQL--A-LE---YGLLGEKEM-SEENQLIE-----------VGGHPGL--------------------PSLSLSSSGNNVNL-------------KLVEEVSNLFYTLLDQTREFLHMSE--------------------------------TPVPSVGEYLKKEIARHMVDW-----------------------TED-EATKRLKLSIMNTFF-NLE-CCVSG--SHSMDLVAL-------GSF--GE----Y----------------------------AMLP-GLDCTFSEGYEGLTNCI--MTSLPK------------------------------NVILLNKP----VKTIHWNGSYRQEKY----------------------------------------PGEKFPVLLEC-------------------------------------------------------EGG---E-----KFPAHHVIVTIPLXXLKEQM-------------------------NTLFSPPLPR---RKAEVIR-RLGFGTNNKIFLEFE--E-----PFWEP----------------------------------------------------DCQQIQVVWEDTSPLADV----------------------------------------------------------------------------------------RAELRDIWFKKLIGFLVL-PPL------------------------------------------------------------------ESTYVLCGFIAGQESEFMET----LSD------EEIL----SSLT-EVLRRITGNPQ------------------------------LSRPRSMLRSRWRSAPYT-RGSYSY-VAVGS----------------S--GE-DID---TLAQPLPAEF------------------S-VPQFQILFAGEATHR--TYYSTTHGALLSGWREADRLISCCDSEAQHLKPKP-------------------------------------------------------------------------------------------------------------------------------------------------------------------------------------------------------------------------------------------------------------------------------------------------------------------------------------------------------------

>Mm_MmA

-------------------------------------------------------------------------------------------------------------------------------------------------------------------------------MAFPGPRVLVV-------------------GSGIA---------GLGAAQKLCSHRA------APH--------LRVLEAT-ASAGGRIRSERCFG---------------------------GVVEL-GAHWIHGPS---------------QDNPVFQL--A-AE---FGLLGEKEL-SEENQLVD-----------TGGHVAL--------------------PSMIWSSSGTSVSL-------------ELMTEMARLFYGLIERTREFLNESE--------------------------------TPMASVGEFLKKEISQQVASW-----------------------TEDDEDTRKRKLAILNTFF-NIK-CCVSG--THSMDLVAL-------APF--GE----Y----------------------------TVLP-GLDCILAGGYQGLTDRI--LASLPK------------------------------DTVAFDKP----VKTIHWNGSFQEAAF----------------------------------------PGETFPVLVEC-------------------------------------------------------EDG---A-----RLPAHHVIVTVPLGFSKEHQ-------------------------DTFFEPPLPA---KKAEAIK-KLGFGTNNKIFLEFE--E-----PFWEP----------------------------------------------------DCQFIQVVWEDTSPLQDT----------------------------------------------------------------------------------------ALSLQDTWFKKLIGFLVQ-PSF------------------------------------------------------------------ESSHVLCGFIAGLESEFMET----LSD------EEVL----LSLT-QVLRRVTGNPQ------------------------------LPAAKSVLSSRWHSAPYT-RGSYSY-VAVGS----------------T--GD-DLD---LMAQPLPADG------------------T-GTQLQVLFAGEATHR--TFYSTTHGALLSGWREADRLIGLWDSQAEQSRPRL-------------------------------------------------------------------------------------------------------------------------------------------------------------------------------------------------------------------------------------------------------------------------------------------------------------------------------------------------------------

>Mm_EqA

----------------------------------------------------------------------------------------------------------------------------------------------------------------------------------------MV-------------------GGGIA---------GLGAVQRLCRHPA------SPH--------LRVLEAT-ARAGGRIRSERSFG---------------------------GVVEV-GAHWIHGPS---------------QGNPVFQL--A-AK---YGLLGEKEL-SEENQLVE-----------TGGHVGL--------------------PSVSFASSGRTVSL-------------ELVVELATLFHGLIDRAREFLHAAE--------------------------------TPVPSVGEYLKQEVSRHMARW-----------------------AED-EETKKLKLAVLNSFF-NVE-CCVSG--THSMDLVAL-------APF--GE----Y----------------------------TVLP-GLDCTFPGGYQGLTNHI--MASLPR------------------------------DTMVFNKP----VKTIHWSGSFQEAAS----------------------------------------PGETFPVLVEC-------------------------------------------------------EDG---A-----RFPAHHVLVTVPLGFLKEHL-------------------------DTFFEPPLPA---EKAEAIR-KIGFGTSNKIFLEFE--E-----PFWEP----------------------------------------------------DCQHIQVVWEDTSPLEDT----------------------------------------------------------------------------------------APELPATWFKKLIGFFVL-PSF------------------------------------------------------------------GSSHVLCGFIAGLESEFMET----LSD------EELL----RSLT-QVLRRVTGNPQ------------------------------LPAPRSVLRSCWHSAPYT-RGSYSY-VAVGS----------------T--GD-DID---LLAQPLPMDG------------------K-EAQLQILFAGEATHR--TFYSTTHGALLSGWREADRLIALWDPQVQPPGTKL-------------------------------------------------------------------------------------------------------------------------------------------------------------------------------------------------------------------------------------------------------------------------------------------------------------------------------------------------------------

>Mm_HsA

-----------------------------------------------------------------------------------------------------------------------------------------------------------------------MESTGSVGEAPGGPRVLVV-------------------GGGIA---------GLGAAQRLCGHSA------FPH--------LRVLEAT-ARAGGRIRSERCFG---------------------------GVVEV-GAHWIHGPS---------------RGNPVFQL--A-AE---YGLLGEKEL-SQENQLVE-----------TGGHVGL--------------------PSVSYASSGASVSL-------------QLVAEMATLFYGLIDQTREFLHAAE--------------------------------TPVPSVGEYLKKEIGQHVAGW-----------------------TED-EETRKLKLAVLNSFF-NLE-CCVSG--THSMDLVAL-------APF--GE----Y----------------------------TVLP-GLDCTFSKGYQGLTNCM--MAALPE------------------------------DTVVFEKP----VKTIHWNGSFQEAAF----------------------------------------PGETFPVSVEC-------------------------------------------------------EDG---D-----RFPAHHVIVTVPLGFLREHL-------------------------DTFFDPPLPA---EKAEAIR-KIGFGTNNKIFLEFE--E-----PFWEP----------------------------------------------------DCQLIQLVWEDTSPLEDA----------------------------------------------------------------------------------------APELQDAWFRKLIGFVVL-PAF------------------------------------------------------------------ASVHVLCGFIAGLESEFMET----LSD------EEVL----LCLT-QVLRRVTGNPR------------------------------LPAPKSVLRSRWHSAPYT-RGSYSY-VAVGS----------------T--GG-DLD---LLAQPLPADG------------------A-GAQLQILFAGEATHR--TFYSTTHGALLSGWREADRLLSLWAPQVQQPRPRL-------------------------------------------------------------------------------------------------------------------------------------------------------------------------------------------------------------------------------------------------------------------------------------------------------------------------------------------------------------

>SD_To3

----------------------------------------------------------MR---------------------------------------------------------------LVL---VLANLP-LLSTSVVASDA-ENI---G----HTPTGRPPATVSSTSEPDEVFVEAVIV-------------------GAGWA---------GISAAIDLQNSGY-------SS--------LLILEAN-DYVGGRSKSMNSDGTLN--------------TPPAELPSNNVPIEM-GSEWLYQSG-----------------STDVSQYSYLRD---GGYLSKVNT-NRYSNESLAL--------------------GRCCA----------NLLTSFYWQTGSSPGQSQLLN--NTEVKSLERNTWR---SYNSFKS---------------------------------------TCSSSHEQCKQAYFNSRSLS--------------------------------SLERQYLNLVI-DS---------CGGMDTSAR-------IDE--LP----ANKTFT------PD-Y------------YIYNT-GYMSPQGVGFGNT-AA-AVAEQL-----------------------------K--DKIRLNSK----VVEINTSTIPR-------------------------------------------------KVIVTYEV--------------------------------------------------ANSGSQ---V-----RVIANSVAVTVSLNVLKANN--------------------------INFVPQLPS---WKQNLIN-GMGMGVLNKCVFVWD--DGAV-AQLFPKKLF----------------------------------------------------WIELISN--QDS----------------------------------------------------------------------------------------------TSGRWTTFLNPSA-----------------------------------------------------------------------QKGKPTLVGWVAGEDAMRME-D---QTD------DEVK----AEMM-SNLKLMFP-D-------------------------------IPEPDRVVITRWGKEPNV-LGAYSH-HVVGRD---------------F--RD-DSS---ALGNPVG---------------------------RIIFAGEATAG--AWYATTKGAWLTGQRAAIEMKQYLTADIVLEASTSLATVA---ANYTMP-----------------MVAVS--ILYF---WRA----------------RRPRVGVIGGAQNVD----------LTGCELRATQHYRTSP----------------------------------------------------------------------------------------------------------------------------------------------------------------------------------------------------------------------------------------------------

>SD_To1

-------------------------------------------------------------------------------------------------------------------------------------------------------------------MPGEDTADTDEVDKTYRFDAIVI-------------------GAGWA---------GINAARHLKAYGV-------RS--------VLVVEAE-DYVGGRSRSFNEDGSVN--------------QPPTTLLAGNVPYDA-GSEWLYTDQ-----------------TL--TH--HLYQ---TGHLYHVDV-LDEND--D------------------------YLP----------LAKMQYYRQHKDWRGHVRTTAMEKSEATRLKSRVWD---TFTGFANHLWN--------------------------------IATEPDMSYHEAAERYRALIS----------------------------------DEDRRYFEAAL-KSV-GQIEY--TANLTDLSL-------TSD----------VFFD------G----------------SEDM-HYMSSTRVGFGNT-AA-AVAFGI-----------------------------G--CDFLVGSK----VTRVDYSRPEV-------------------------------------------------LVT---IE--------------------------------------------------MNGGTQ---A-----ELVSTVVAVTVPLGVLKANS--------------------------ISFVPPLPS---KKQQVID-KMKVGVSNKCIMIWD--SPG--SLVWPKDEI----------------------------------------------------WFTFMPLEDTSG----------------------------------------------------------------------------------------------QVPRWTTFSNLSK-----------------------------------------------------------------------YKGKPVLVGWIGGDDARHIE-S---LTD------DEVL----DEVM-ISLREMFP-T-------------------------------ITRPDRVIVTRWASEPNF-LGAYSY-KSVGRS---------------F--SS-DSA---TLAKPVG--------------------------DRLFFAGEATAG--AWYATTTGAWTSGYDAAVLMIKALLKSNENNAVD--------------------------------------------------------------------------------------------------------------------------------------------------------------------------------------------------------------------------------------------------------------------------------------------------------------------------------------------------------------

>SD_To2

------------------------------------------------------------------------------------------------------------------------------------MLT-RVLGLGVVGVA-SA---------LLMLTANVEKALVAGTRPEDRYDAVII-------------------GAGWA---------GINAARELKASGV--------S--------MIILEAN-DYIGGRSKSINSDGTLN--------------APPAELPSNNVPMDM-GSEYLYTAN-----------------EL--KN--YLRR---NGFLENIDL-DDAED--SPP--------------------HVLSG----------DRSIGYFRQERYIDGTTRTIGLIPNDLRSMYSAMWR---PFVEYIQELYQ----------------------------------SEGEMSYADALERYTAARQIS--------------------------------NTDRQYLNLML-DAG-LEIEY--GGESGRMSI-------WYH--DL----G-AILN------N----------------DSPI-HLMSKIGVGYGNT-AA-AVAESN-----------------------------D--LPIQLNSK----VTR--------------------------------------------------------------------------------------------------------------------HEGEV---A-----TVRAKVVSVTVSLGVLKSNI--------------------------IEFTPDLPA---QKKDAIE-NMEVGIFNKCAMTWN--DRG--ALVWPEEQL----------------------------------------------------AFELITPT--DE----------------------------------------------------------------------------------------------TSGRWTTFNNPTL----------------------------------------------------------------------YKGGKPTLVGWIAGDEAVRME-S---QSD------EEVL----DEVM-VNLEAMFP-D-------------------------------ITRPDEVHITRWGSDPSF-MGSYAH-MAIGRD---------------H--EQ-DAM---NLGARVG---------------------------RISFAGEATDA--TWYGTTVGPWKSGGRVAEEMMAILAVEVPTPSPSFSPTE----LNTGTP-----------------SKSPT--VSLSEAPSMTPSDVDV---------NENVVEPVNEVLVSND----------PVETNLPAPEQADTDGENSS-----------------------------TAISVSILVFLIIAFIN--------------------------------------------------------------------------------------------------------------------------------------------------------------------------------------------------

>SD-Fc1

------------------------MTEFNGCILDYDIHYSPLLLFQESEKKFSNPMRSSN---------------------------------------------------------------PMR---CLKNLP-GIEELITDYSP-HSL---TIVYISIFLSLGVLCSTGSASTNDDILDVVII-------------------GAGWS---------GLAAANYLIEAGI------TEN--------ILILEAR-DRIGGRSYTNDG------------------------LFESGHPVEL-GSNWIYPDT-----------------NV--FD--LVNE---LGIAHDTTV-FDFDT-------------------------------------------LGLYDSGGELL--------EEEKSILVDETFLK---DFVKYADK------------------------------------MANDETSWADIKQSYFTERPDLD-------------------------------SSKRQAINALV-HTG-ISIEF--GSPLNETNS-------GTT--KE----Y-LERG------DW-R------------NIEVM-SVSGGEGGGYTGALSR-GLAKSF-----------------------------E--EKIKTNTP----VVKIDQSGDV---------------------------------------------------VEVYTTN--------------------------------------------------G--------Q-----VIYARSVIVTVPLGVLKKSS--------------------------IEFVPPLND---AKLEAID-LIGMGNMNKVLMYWD--MTTQDVSWWPEGKV----------------------------------------------------DMQLITDQ--DS----------------------------------------------------------------------------------------------DSEDWTYFYNDHS-------------------------------------------------------------------H-VGNEDYHAMTSWNAGDAADRLE-K---NTD------EETM----DIVL-GNLRKMFGND-------------------------------VPSPSKYIITRWRSEEFS-GGAYSF-DTVGSD---------------L--TD-YRK---TLGEPM---------------------------NNVFFAGEATDID-GWFGTAVAAYTTGVKAAGEIDDSGILDM----------PLPDFQPTCTR-----------------MHGRCGEEFDEACCSGMSCVLDD---------RMAPIPTFTSLEGSIN----------TITTTMIWICSPSQKKETER------------------------------NRFGSISRIS----------RTGHRFSGSGSD----------------------------------------------------------------------------------------------------------------------------------------------------------------------------------

>SD-Fc2

------------------------------------------------------MIRDHD---------------------------------------------------------------TTR---LPSLVP-LLLGLIITLSI-SLT---LFTGV--TGETFSDDVDNDNNNNTVVYDVLII-------------------GAGWS---------GLAAANALRDKGI-------TN--------VKILEAR-DYIGGRSRTLQD------------------------YFVDGLATEV-GSSWVYRHS-----------------AI--DA--LYNK---LGLQWDVSR-FNFGT-------------------------------------------IKLYNEWGALS--------KTD-SKKLKSDYID---GFVAYAKNQYA-------------------------------NKDDDIDLSVEELMASYFASSDGRNL-----------------------------PNMRRQAVHAFV-T-P-VSTNL--GQIDDEADA-------SNI--IY----G-LT---------W-E------------D-EEL-DFTAVPHGGFTPV-VN-EYSKPI-----------------------------A--HWITRNSV----VTKINYTRNH--------------------------------------------------IVEVTTNN--------------------------------------------------GVNSGN---T-----LYHTRTVICTVPIGVLQHRD--------------------------IEFVPDLPE---KKWNAID-SIGNGSVNKCIMYWD--RNTKNTSWWPEGEL----------------------------------------------------DLQLITNA--DT----------------------------------------------------------------------------------------------GSDAWTYFINDQS-------------------------------------------------------------------H-ATNHDHYVLTAWSAGDIVKELE-A---ETD------EQTL----ARVL-INLRTMFGEE-------------------------------VPEPTKILVTRWLSDPYS-RGVHTF-RETGVD---------------N--ER-AKR---ELKRSLD--------------------------DQVFFAGEAV----DWGSNTVAAYNSGIAVANDVVKSLYIDDDDNSNNDVQEDQPTFQPTLYN-----------------TFNPTSLDTSSEYSVGNVCADD-------------------NNYRYYK----------DAWKDCTWIVENDRCNRSDN------------------------------KSGKSVTEYYCPVLCGYCDGSDSDDGNGSGNDNDSAGDTTDLDKQNVVGEIGTGTNSSTEDSSYASAVTPSESPITVKPTDKPTEQQNENEIQTENEIITDVAVDLQQNEEIKEVDQKQQEEIFEALLIEISASLEEEEREEIKEEDQQDQNEKIKLEDQQQQEDHSGFEKVEGFMVSRNSRLRNRIKIVSERDGYRLRSSTKQEVQGGD

>Eh-Ng

--------------------------------------------------------------------------------------------------------------------------------------------------------------------------------MSQHKNVVII-------------------GGGVA---------GLYAAKTLSDNQV-------SN--------FIILEAS-SRLGGRVCSDSE-----------------------------RGIEL-GAEYVHGEE-----------------TILWDI--LVNQ---LNVELEFNH-DMT-----------------------------------------------KLTPKECGLGLVVELD--EILEK------------LIN-DEE-----------------------------------LKFAKDMTLKEWLI----WR-----------------------------------EIPEKYH-LLV-DST-IAQEN--ATCIDVYSA-------KGA--QE----DLIA--------EFNS------------EFGDG-NYFLKGKNTLENLIIPYLVKSIQ-----------------------------Q--EQIRLDYA----VDSIDYTSNQI------------------------------------------------------KIN--------------------------------------------------G--------G-----DIICDKIILTVPLTTLQHNL--------------------------IQFTPDLSP---IKKHLIQNQFNMNGGTKIILNYK--K-----PFWREQFP----------------------------------------------------NVNVVCVGDDDPL---------------------------------------------------------------------------------------------ISQFWFSSTS--E-------------------------------------------------------------------E-QSEKFGHVITGFSMSNRANYGI-V---MGE------EKVK----DHFR-KMIQNICGLT-----------------------------SENDGFIGGMVRNWQDVEHI-SGAYAS-VNTSEESRQNY----------I--GN-PRL---ALSQPIE--------------------------SKIYFAGELFCT--HSPATIHGAMETGRDAALRVIAEM------------------------------------------------------------------------------------------------------------------------------------------------------------------------------------------------------------------------------------------------------------------------------------------------------------------------------------------------------------------------

>ALc-Tt1

-------------------------------------------------------------------------------------------------------------------------------------------------------------------------------MSTKRKSVLII-------------------GAGIS---------GLAAAHSLHENG--------FD--------VQIFEAR-KEFGGRIRKDDS--------------------------FAGFTLEV-GGEEIHKVN-----------------SPYYHL--ALK----MGADLKPDD-TLNHY----------------------------FE-----------DIEK---------EELIDRE--EFLNKYNDQYFYN---EVV--QNR-----------------------------------DIQDDSQSLQNFFT----KK-----------------------------------GLKSQFY-QWY-EAF-WGIEN--GGSLNEISV-------KAY--GD----YESG---------R-K------------SDHDL-NFILMNTSHYEIIEKAF---ESV-----------------------------L--PFIHYSTP----ITEINYFGEKEHPLQR-----DEDDEDED------------------NDEDDCKG-KDFNRVIIFDKQ--------------------------------------------------G--------N-----RYEGDYIIVTVPISQLQNKT--------------------------IRFNPELPP---QKQDAIR-RMKLGRGGKIHFKFK--N-----RFWPDNAR----------------------------------------------------TIFLR------SK---------------------------------------------------------------------------------------------ISFLWNQYHE-QK-------------------------------------------------------------------D-TDEIQTNVLAGLLAGDIMD----E---MQD------PEKRQALIDEVL-EKMTRVFKYP-----------------------------NAKEELLDVMWNDFTNFEYI-QGNYSM-PTLN-----------------I--GS-SRY---IYQQPVD--------------------------NILFFAGEASHT--TDSMTIHGAYETGLRDAQRIIDLQKEKK--------------------------------------------------------------------------------------------------------------------------------------------------------------------------------------------------------------------------------------------------------------------------------------------------------------------------------------------------------------------

>ALc-Tt2

-------------------------------------------------------------------------------------------------------------------------------------------------------------------------------MERIQKTVIVV-------------------GSGIS---------GLSCAYELLKNG--------FT--------VQILEAR-HIHGGRISKNST--------------------------FADFPIET-GAEEIHLPT------------------KYYKI--AKE----VGAKCESDS-DFNSY----------------------------IE-----------DLPKKGEDLSMGSGILIDEE--DFYDKYKIEKFYK---SILKEEEK-----------------------------------KFLKDDMSILEYFK----FK-----------------------------------QIDDRLI-QFY-ETV-LANEY--GSTLQEMSI-------KGY--AE----HELN---------W-E------------YE-EK-RYVITNMSHFDVVDRAF---STV-----------------------------L--PFVKYNTP----INYIAIQTNQL-------------------------------------------Q-NQSNGVTLVDAY--------------------------------------------------G--------N-----EYKADHVVVTVPVSQLKNGS--------------------------INFVPPLSQ---EKQRAIQ-LLQMGKGGKLHMKFK--E-----KFWPSDYY----------------------------------------------------AVVLR------TQ---------------------------------------------------------------------------------------------IGLVWNCSYH-R------------------------------------------------------------------------SKKSLVLCALISGQASI----D---MND------PNKRKQLMSELF-VKLQQVFKLKK----------------------------NVEELLEDYIWTDFNTMKYI-EGTYTY-PSLN-----------------L--GL-FRN---ILAQPVN--------------------------NQIFFAGEATEP--LYYATINGALDSGVREAQKIISLYKK----------------------------------------------------------------------------------------------------------------------------------------------------------------------------------------------------------------------------------------------------------------------------------------------------------------------------------------------------------------------

>ALc-Tt4

-------------------------------------------------------------------------------------------------------------------------------------------------------------------------------MNKIIKDVIVI-------------------GAGIS---------GLSAAHALVQKG--------LN--------VAILEAK-STFGGRISKNSQ--------------------------FADFPIET-GAEEIHLKE-----------------SAYFQL--AES----VGAIIQSDD-QVNNY----------------------------IE-----------SPDE---------EVLLERD--HFFENSGREEFYQ---MVM--KNR-----------------------------------KQLNENMSVLEYLE----QQ-----------------------------------KVEQKYF-KFY-ENF-WGAEN--GTSIKNISI-------KGL--AD----YESG---------W-K------------SDHDV-NYLITNMSHFDVIEKAY---ASI-----------------------------L--HLIQYNTP----VKSIHYGSDIQSL-------------------------------------DKQNN-SENYSVQITDKN--------------------------------------------------G--------R-----IFYSKYALITVPVTQLKQGK--------------------------IEFYPPLPE---KKQHAIQ-SLQLGKGGKLHLSFK--E-----KFWPNKFG----------------------------------------------------SMILQ------SS---------------------------------------------------------------------------------------------IGMVWSCSDL-R------------------------------------------------------------------------SEQSHVLCCLITEPVAL----D---MND------PIKQKQLIAELL-QKLSRIFKRD-----------------------------DIESLLNNTHWIEYSQIEYI-EGNYTY-PSLN-----------------M--GN-SKE---ILSQSVD--------------------------NKLFFAGESTNP--RYSSTIHGALETGLREAAKIIDIQEEQIDQLQIQN-------------------------------------------------------------------------------------------------------------------------------------------------------------------------------------------------------------------------------------------------------------------------------------------------------------------------------------------------------------

>Fa_Ff1

-------------------------------------------------------------------------------------------------MLIKSDN--AS-------------------------------------QY-H-----ER-LDMISKHLSAMAVDGFGAGTPAHYDTIVI-------------------GAGIS---------GLAFASRILQSNDTSGGCTPRR--------LRILEAR-DRIGGRIASVDVQ---------------------------GQRLDT-GANWIHGIG------T------PARPNPLMSI--V-PQ---KRYRAMQGS-VLFQTPAEEQNSE-----TCKANQQVSSEGTVCPAATT--------MPKTTCTNGLVVPTC--------------IGQLIA---SCHEKAIAQVQDLA----------------------------AGLPEKAAKYVSILQSLVSRNAFQNAFEVV-------------PREYHHTLGSLFQGIESMEAAPLL-ART-TEPLR--SESKPGVGL-------LEY--AV----D-----------------------------DFD-GEHIVLQDGYIEITNEI--AKPLL--------------------------E-A--GSISFNTV----VTGIAWGSN--PIS-------------------------------------------------IETTR-----------------------------------------------------------G-----NFTAREVVCTMPLGVLKDTA--K----------------------TEMFTPSLPA---DKQEAIE-SLGFGTLDKVFAVYS--K-----PWWNDEPYRS------------------------------------------------IITSGFIRGDTQSDDDMPD--------------------------------------------------------------------------------------------SFLGFTSEL--------------------------------------------SGISI--SSDGSVTPDVYRLPVMNL-DSLTGQPVLCAFVSCNTSITVE-E---KED------EDVG----QIFH-RALTQWFGVE-------------------------------PPEIESVHVTRWGQDAYS-KGSYSH-MITGVS---------------E--IR-HRE---LLQVPMI----------------------NEDGGVLRFAGEHCSR--DHFAMAHGALLDGWRAAEESSQYLKINSMN------------------------------------------------------------------------------------------------------------------------------------------------------------------------------------------------------------------------------------------------------------------------------------------------------------------------------------------------------------------

>Fb_Ml2

-------------------------------------------------------------------------------------------------MASSNTN--NKSNSNHTI------------------------------RN-P-----FNSILKA-----HELNQFEISELEGTHQVVII-------------------GAGMA---------GLSAALKLAKLN--------YK--------VIIVEAR-DRVGGRIETREFQTSTKSNDSV---------------KEDPSRIDL-GASFLHGI----------------EGNPLIDL--M-KE---YKQPVHFEN-EESPMKIYSFDGP-----ALPDK---------------------------------STKKL--------------IDHAYL---TFFESARNDAQASET------------------------------PDSAASLGSYLYDPQS-PLFNVA-----------------------------SGPEDRSVLAHL-VGG-LESWT--GAALEQVSL-------RWW--GF----E--------------------------R--EFN-GKDGVVTHGYGVLVNLM--AQEFI--------------------------RLG--GKIILGYE----CLGLEYDLDAGLVK-------------------------------------------------TLIRP---TLSESLEDNAHAERIPRPAEEA---------------------GSKSIQEGAV---I-----RLSSDYTVCTLPLGVLKSILVKD----------------------HLFFNPPLPA---RRCQAIE-RIGFGLLNKVILRYD--H-----AWWPI--DAP--------------------------------------------------CSGSTSSDSSSGAS----------------------------------------------------------------------------------TPSSVSPFHGHLPNHASLLES----------------------------------------------------------TIFATSVKVQNY-VPITGEAALVFFFGASAGEAIE-E---LSD------QSVS----EMMH-AKLVAHLDDAE-------------------EDD---RHLEIPEGPSECIVTRWRKDRFS-LGSYAF-IPPFSKQ------ASNLDEPAT--PL-DIM---EMNRPLW-------------------------NGRLGWAGEHCQV--DHYACVHGPHLSGLEEAERIHVAIQANTTGSPSSSV------------------------------------------------------------------------------------------------------------------------------------------------------------------------------------------------------------------------------------------------------------------------------------------------------------------------------------------------------------

>Fb_Pg

-------------------------------------------------------------------------------------------------MASCNQT--NQVCNNGAT------------------------------NR-A-----NCLGVREETSTMGPTNPQQNHTNHSPIDVLVI-------------------GAGIS---------GLTAALQLTRAG--------HP--------VTIVEAR-DRVGGRIDSHDWAD---------------------------GSIDL-GASFLHGV----------------DGNPLVDL--L-KQ---FDEPLYFEN-ETDPIKIYPYQAE-----RLSDQ---------------------------------TTKEL--------------YDHANK---TFFSTARTFSQSMLL-----------------PHPHPHTSSGLPYNPPPKSLYDFLLDSPTSPLYKNH-----------------------------HTPAERNVLQEI-VNS-LDSWT--GASSEQVSL-------KWW--GF----E--------------------------K--DYT-GEDGVLPNTYSSLIRKM--ASEFE--------------------------RLG--GRILLDSE----CERIQLQIPTGRIR-------------------------------------------------------------------------------------------------------VRVAGKP---E-----EIEAGCCVCTLPLGVLQAK--------------------------ADIFDPPLPP---RRLLAIS-RTGFGLLNKVVVRYP--T-----CWWSG--GVR--------------------------------------------------WFVLLPAEAESETDSESEG-----------------------------------------------------------------------SHPSADSSITSARSSSPENHWPSPTMSG--------------------------------------------TSKSN--SRPEYSVLFSKGVKVQNY-VPITGEPVLVFYLGAEAGEAVE-H---FSN------EYVA----ELIH-EKLLSQ---VP-------------------VEE---RSVEEPDLPSECLVTRWRSDPYA-RGSYSF-MKTKTSPKFNDHGDLEDHEDSN--PL-DLI---EMSKPLW-------------------------DGKLGFAGEHCSV--DHYACVHGPYMTGLEEAQRIQSNYHSFNPHTEQDQKDISRLT----T-----------SF----QK---------LI-------------------------------------------------------------------------------------------------------------------------------------------------------------------------------------------------------------------------------------------------------------------------------------------------------------------

>Fb_Ml1

----------------------------------------------------------------------------------------------------------------------------------------------------------------------------MDKIDDQEYDCIII-------------------GGGFS---------GLISAIELQKRSE-----SNLN--------ILILESQ-SRLGGRSLTDLN--------------------------RFPLPIDL-GCSLIHGYH---------------EGNPMSQI--A-KE---FNVEV-VVT-PDQDTLVLGHDG--------------------------------------------LLDLN--------------ESKSIL---ESLDKCINEVKQ--------------------NLK-------ESIPPETESLEDSLRNHIT-THYSN----------------------------------QSNLLSKL-IQT-IEVGA--GIPLNQISS-------KHF--GF----H--------------------------R--SFS-GSDGLPTGGYQEIVNQI--EKKIN--------------------------QLG--LQLKMNSE----VTKLVYDKENSKVK-------------------------------------------------LEVCN---K---------------------------------------------SDSSSTT---Q-----SYQSKYCISTIPLGVLKTN--------------------------PPKFEPPLEL---LTRLSIE-NTSVGLLNKIVLNYE--Y-----AWWPNSKTIG--------------------------------------------------RYILTSNRNTKLTEKT-------------------------------------------------------------------------------------------NSLTDILAMTT--------------------------------------------FW----------VDNLAVEN-------CNQSYPILIIPIGALAAKEIE-K---FSD------EDII----QTLH-KYLTQRFQIPD----------------------------QMLNLPKSSTITRWESNLYS-RGATSS-PIRIK--------DD-KISSTS--PL-DLI---LLSRSNW-------------------------DGHLGFAGEHTEV--DHRGSVAGAILSGKREAKRVIQLLDLTAKH------------------------------------------------------------------------------------------------------------------------------------------------------------------------------------------------------------------------------------------------------------------------------------------------------------------------------------------------------------------

>Fb_Um1

------------------------------------------------------------------------------------------------------------------------------------------------------------------MAKAAAATSSATTAASHMLDVLII-------------------GAGWS---------GLSAALKLSQAG--------RK--------VAILEAR-ERIGGRAFTHTWNDKTDLNDTS---------RTLTAPSAADYWCDL-GCSWIHGYL---------------EGTPLKAL--T-DK---YSIPV-TLA-SERETVVVAEQG--------------------------------------------PLPQA--------------LSHKLI---ANLATAQQAAKTAAL-----------------DHT-------TTPPDANTSLADFLYSDHS-PLFANL-----------------------------ASETEKSVARHV-ARM-LHIPL--GIELEKASL-------KWH--GF----E--------------------------H--AFA-GTDAAPKGGFTTMINKM--VNEIT--------------------------SLG--ASIYTGQE----VQSVQDG---DNVK-------------------------------------------------VTTKQ---G-------------------------------------------------------E-----QYTAHTALVTIPLAVLKNTA-------------------------GRLFEPALPE---RRLETIK-RVSVGNLNKVLLHYH--Q-----PWWNA--TTG--------------------------------------------------TFLVVPCSLAVPSSVKSEA---------------------------------------------------------------------------------------QKELWHLYSSTT--------------------------------------------LI----------VASLSSEQRASEA-GGSGASNSLLVMIGADSARQLE-A---YER------LDAG----NTLH-TYLVARIVGSD---------------------------HASTQPPKHIFYSRWANHAFT-GGATTS-PVSTA-------------SGSS--PL-DFE---MLSRPLW-------------------------NGRLGFAGEHTEI--NRESRSFRSHHDAHRSNEPINKLPISFFFFIPTVNRSRLGSR----RLR---------VW----PARS-----ESSLGVSGQAVSSNSQQALNESDEAGRRFYFDFVDTLRYQES-----------------------------------------------------------------------------------------------------------------------------------------------------------------------------------------------------------------------------------------------------------------------------

>Fb_Sr

--------------------------------------------------------------------------------------------------------------------------------------------------------------------MAKAAATTTSNAANNMLDVLII-------------------GAGWS---------GLSAALKLSQAG--------RK--------VAVLEAR-ERIGGRAFTHTWSDKTDVNDKS---------RTVAAASASDYWCDF-GCSWMHGYL---------------EGSPLKAL--T-DR---YGIAV-TIP-AARETVVVGEQG--------------------------------------------VVPRE--------------LAQKLT---ANLGKAQEAAKGVAH-----------------DQS-------ASPPDARTSLADFLYSDQS-PPFAGL-----------------------------ESEGEKKAARDL-ARM-LHIPL--GIELEKVSL-------KWH--GF----E--------------------------H--AFA-GTDAAPKGGFTSIINKL--VDEIT--------------------------ALG--TAIHTSQQ----VHSVKDQHASSNVK-------------------------------------------------VTTTQ---G-------------------------------------------------------H-----EYVARAALVTIPLAVLKKNA-------------------------GALFEPALPE---RRLATIG-RVSVGNLNKVLLHYA--Q-----PWD-A--NTG--------------------------------------------------TFVVLPSTAVPAPPSVTGE---------------------------------------------------------------------------------------QKKLWELYASTT--------------------------------------------LI----------VSSLAGDA----E-VGKGASSSLLVMVGADAAKQLE-A---FER------LDAG----NALH-AYLTARITGP------------------------------DAPRPKHVFYLRWAKQPFT-GGATTS-PVSTA-------------SGTS--PL-DFE---ALARPLW-------------------------NGRLGFAGEHTEL--NHRGSAAGAYVSGEREASRLVAYLDKLHPREQGKL-------------------------------------------------------------------------------------------------------------------------------------------------------------------------------------------------------------------------------------------------------------------------------------------------------------------------------------------------------------

>Fb_Uh

--------------------------------------------------------------------------------------------------------------------------------------------------------------------MAAATTTTSAAAAGNILDVLII-------------------GAGWS---------GLSAALKLSQAG--------RK--------VAILEAR-ERVGGRAFTHTWSDKRDVDDNS---------RTVSTASAKDQWCDL-GCSWMHGYL---------------EGSPLKQL--T-DK---YDISV-TIP-GPRDTVVVGEQG--------------------------------------------PLPQA--------------LSQKLT---ENLGKAQDAAKHIAH-----------------EKD-------VSPPDANTSLADFLFGESS-PLFAGL-----------------------------ESGGEKKAAGDL-ARM-LHIPL--GIELEKASL-------KWY--GF----E--------------------------Q--AFV-GTDAAPIGGFATIINKL--VDEIT--------------------------LLG--TSIHTSQE----VQCVRDELQSSKVK-------------------------------------------------IITKQ---G-------------------------------------------------------Q-----EYVARTALVTIPVAVLKKTA-------------------------GGLFEPALPE---RRLDRIK-RVSVGNLNKVLLNYD--Q-----PWWSD--KTG--------------------------------------------------TLLALPCSAPAPASIKSDA---------------------------------------------------------------------------------------EKKLWELYSSTT--------------------------------------------LI----------VSSLAGGR--CDA-AGKGASNSLLAMVGAEAGKKLE-A---FER------LDAG----NALH-AYLTARIGAG-----------------------------EDVKAPKHIFYSRWGEQPFT-GGATTS-PVSTA-------------SGNS--PL-DFE---ALSRPLW-------------------------NGRLGFAGEHTEI--NHRGSAAGAYVSGEREAKRLVAYLDKFYPCTANKL-------------------------------------------------------------------------------------------------------------------------------------------------------------------------------------------------------------------------------------------------------------------------------------------------------------------------------------------------------------

>Fby_Rg

---------------------------------------------------------------------------------------------------------------------------------------------------------------MAALAPLAAPSTPLSGEPEPAYDVVVV-------------------GCGMA---------GAVAARQLA--G--------HR--------VALLEAR-NRVGGRIYTAGEV------------------------EGVPQPVDL-GGSMIHGFR---------------EGVPTAKL--ITHE---LGMDV-HVP-QGAKGLVYGLNG--------------------------------------------PLAEA--------------EATSLF---ATS------AQN-A------------------FSP-------PSGVAADASIASLLIPT----------------------------------------LKSDPRLVAL-ART-AEIGA--GVELEGMSA-------KYA--GF----E--------------------------Q--GFK-GTDGFPEGGYGEVMKNL--VADIK--------------------------AAG--GEVHLGVE----VTKIEDLGADKGVK-------------------------------------------------LETKD---G-------------------------------------------------------R-----TFTAKAVISTIPLAVLQQS--------------------------PPTFQPPLSS---LYTSAIE-RMRTGSLEKIVLSYP--S-----AWWPSPDENG--------------------------------------------------SFLLLPLHDPSVPLDDA-K---------------------------------------------------------------------------------------PASLRDLFSRIV--------------------------------------------IP----------VSS-FQRI-------ASAPHPTLLAYIGATAARYIA-A---YPA------DDVT----SAFH-DYLVSRLSPS-----------------------------ALPPAPTVKLVTEWQRDPFS-RGATST-PVPLT--------QSKDGERAS--PL-DFI---IVSRPIW-------------------------DGRLGFAGEHTDL--DNHGSVAGAAISGQREGLRVKELLERLAEQEANEQGKALL--------------------------------------------------------------------------------------------------------------------------------------------------------------------------------------------------------------------------------------------------------------------------------------------------------------------------------------------------------

>Fby_Rt

---------------------------------------------------------------------------------------------------------------------------------------------------------------MVVPASRAAPSTPLSGEPDPAYDVVVI-------------------GCGMA---------GAVAARQLA--G--------HR--------VALLEAR-NRVGGRIYTAGEV------------------------EGLPQPIDL-GGSMIHGFR---------------EGVPTAKL--ITHE---LGMDV-HVP-QGAKGLVYGPNG--------------------------------------------PLAEA--------------EATSLF---ATS------AQN-A------------------FTP-------PSGVPADASIASLLFPT----------------------------------------LKSDPRLVAL-ART-AEIGA--GVELEGMSA-------KYA--GF----E--------------------------Q--GFK-GTDGFPEGGYGEVMKNL--VADIK--------------------------AAG--GEVRLSVE----VTEIEDLGAGKGVR-------------------------------------------------VETKD---G-------------------------------------------------------R-----TFTAKAVISTIPLAVLQHS--------------------------PPTFQPPLSP---LYTAAIE-RMRTGSLEKIVLSYP--S-----AWWPSPDENG--------------------------------------------------SFLLLPLHDPSVPLEEA-K---------------------------------------------------------------------------------------PASLHDLFSRTV--------------------------------------------IP----------VSS-FQRI-------ASTPHPTLLAYIGPAAARYIA-S---YPA------DEVA----SAFH-DYLVTRLSPS-----------------------------AAPPAPTVKLVTEWQRDPFS-RGATST-PVPLT--------QSKDGERAS--PL-DFV---IVSRPTW-------------------------DGRLGYAGEHTDL--DNHGSVAGAAISGQREGTRVKELLERLAEQEANEQGKALL--------------------------------------------------------------------------------------------------------------------------------------------------------------------------------------------------------------------------------------------------------------------------------------------------------------------------------------------------------

>Fb_Gt1

--------------------------------------------------------------------------------------------------------------------------------------------------------------------------------MSRLYDTIVI-------------------GAGWS---------GIVAARDLSKAG--------HS--------VLILEAR-DRIGGRARTY--T------------------------DGMHVPVDL-GCSFVHGYK---------------EGNPARDI--A-KE---FGVKTNVCQ-L-TERQVWRSSK--------------------------------------------PEPPS--------------VEHKLV---ANLGAARSSAQALA------------------QSA-------SPPPSPLTPLSEALFSQQS-PLFAD--------------------------------IQDKELAVSF-ARS-LEVPL--GVVLEKASL-------RWD--GW----E--------------------------N--NFA-GSDAAPEGGFQRFLEKI--VDESK--------------------------KDG--VEVRLGEA----VKKVEKS--QEGVK-------------------------------------------------ILTAK---D-------------------------------------------------------G-----EYTAKTVICTIPLGVLKTNA-------------------------KTLFEPPLPT---RRLETIA-GTHVGVLEKLVLAYP--S-----AWWPDAAKAS--------------------------------------------------PYIHLPSETRQSEAK-D--------------------------------------------------------------------------------------------AKSVFRQNT--------------------------------------------FI----------LASFAAPV-------LPQQHPTVLFYISETPALALE-K---FSA------EEVA----TAGH-EFLVERFAVG------------------------------SAPKPTGHVLTNWRTDPLA-LGATTT-PSIVG--------E-----GRS--PL-DFV---ELGKPLW-------------------------GGSLGFAGEHTDA--NHRGSVAGAVVSGAREAERVAKYLNKLKET------------------------------------------------------------------------------------------------------------------------------------------------------------------------------------------------------------------------------------------------------------------------------------------------------------------------------------------------------------------

>Fby_Ta

--------------------------------------------------------------------------------------------------------------------------------------------------------------------------------MPQVFDTIII-------------------GAGWA---------GAVAARDLAHAG--------RS--------VLVLEAR-DRIGGRARTW--A------------------------NG-DARIDL-GCSWIHGYN---------------EGNPARWI--A-KD---MGVQTTHLP-KPTPSAIYGPSG--------------------------------------------PLETP--------------VAAQLG---SALG----AAQAAF------------------RTP-------HPAPGPRESLADALFSSAS-PLGK---------------------------------VEDKSLAEGF-ART-LEVPL--GLKLERASL-------RWA--GW----E--------------------------GATNFA-GSDAAPEGGYEALVGKV--FQDAE--------------------------KHG--AVVKTGEP----VTGVRDM--ESGVA-------------------------------------------------VQT-D---K-------------------------------------------------------G-----SYQARTVLCTIPVAVLRQQ--------------------------LGIFHPPLPE---RYQDIVR-GVNVGVLEKMLLNYD--K-----PWWPRANEVA--------------------------------------------------SYIFLPTKAPGGQ---S--------------------------------------------------------------------------------------------LMDVLESST--------------------------------------------II----------TANLAGPA-------LPGATPTLLSYLSDTPARAAL-S---VSP------AEVA----QTFH-AFLKKRLDVP------------------------------DAPEPRASEITNWLTDPLS-LGATTT-PTPVS---------D---GERS--PM-DFK---ELSRPTW-------------------------DGKLGFAGEHTEM--ENRGSVAGAVVSGMREADRVKRYLSKHDAKKQ----------------------------------------------------------------------------------------------------------------------------------------------------------------------------------------------------------------------------------------------------------------------------------------------------------------------------------------------------------------

>Fb_Cf

--------------------------------------------------------------------------------------------------------------------------------------------------------------------------------MSNAYDSIIL-------------------GAGWA---------GSVAAKELTSKG--------HR--------VLVLEAR-DRVGGRARTW--T------------------------GG-GAKIDI-GCSWIHGYK---------------EGNPARNI--A-KS---LGVEA-RLP-AAAEGVIYGPNG--------------------------------------------PLSAE--------------EADALR---ASLG----TAVASS------------------KLP-------HPSPPPTTSLASALFSPNS-ALFSTA-----------------------------S---DQSLAKAL-ARS-LEVPL--GLKLEKASL-------KWA--GW----E--------------------------TTTSYA-GSDAAPEGGYQSLVTKV--LESS-----------------------------K--AEVKLNSP----VTSIKET--SSGVE-------------------------------------------------VTTRS---G-------------------------------------------------------E-----TYSAASVLSTIPLGVLKSLP-------------------------EDFFTPALPA---HLRETIA-GTHVGVLEKLLVQYP--T-----AWWPNAEKVG--------------------------------------------------SYTFLPTGPEPSASS-T--------------------------------------------------------------------------------------------LEQVFEGST--------------------------------------------LI----------TANFAAPT-------LPGPTPTLLTYLSETPAKILL-Q---HPT------EKVA----EAFH-SFLVKRFSPS-----------------------------SPPPAPSASALTTWLTDPLS-RGATTT-PSIIS---------T---GERS--PM-DFK---ELSRPVW-------------------------GGKLGFAGEHTEM--ENRGSVAGAVISGFREADRIDKWLAVRKE-------------------------------------------------------------------------------------------------------------------------------------------------------------------------------------------------------------------------------------------------------------------------------------------------------------------------------------------------------------------

>Fb_Cn1
[truncated: 753,961 more chars]
