# Supplementary material for: The tree of life of polyamine oxidases
Source: Sci Rep. 2020 Oct 20;10:17858. doi: 10.1038/s41598-020-74708-3 (PMC7576179; doi:10.1038/s41598-020-74708-3)
Supplement: Supplementary file 2 — Supplementary Figure S1 [file 41598_2020_74708_MOESM2_ESM.pdf]

Scientific Reports

The Tree of Life of Polyamine Oxidases

Daniele Salvi[1] and Paraskevi Tavladoraki[2,*]

1 Department of Health, Life & Environmental Sciences, University of L'Aquila, 67100 L'Aquila, Italy.
2 Department of Science, University 'Roma Tre', 00146 Rome, Italy.
*Corresponding author: Paraskevi Tavladoraki, paraskevi.tavladoraki@uniroma3.it

ZmPAO1-like

```
Pb-Pp2     MVLDLYHNDYEYAEPPRVTSLKSTQPLPTITNFGEAFQFVHD
Pb-Pp3     IVLDYYDNDYEIAEPPRVTSLKNTQPLPTITNFGESFQFVHE
Pd-Rc4     MVIDFFYNDFEDAEPPKVTSLKHTYPRNEMVDHGEDEYFVAD
Pd-Pt1     MVIDYFYNDYEDAEPPRVTSLKNTIPRYEFLDFGDQTYFLAD
Pd-Cc1     MAIDYFFNDYEDAEPPRITSLKTTYPRNQLVDFGEDSYFVAD
Pm-Si4     MALDYYKFDYEFAEPPRVTSLQNTEPTPTNADFGEDSNFVAD
Pm-Si2     MALDYYKFDYEFAEPPRVTSLQNTEPTPTNADFGEDSNFVAD
Pm-Bd1     MALDYFRYDYEFAEPPRATSLQNTEPLPTAADFGEDNHFVAD
Pm-Hv2     MALDYYKYDYEFAEPPRVTSLQGTEPTATFADFGDDANFVAD
Pm-Os6     MAVDYFTYDYEFAEPPRVTSLQNTVPLPTFTDFGDDTYFVAD
Pm-Sb1     MVVDYYKYDYEFAEPPRVTSLQNVVPLPTFSDFGDDVYFVAD
Pm-Zm1     MVVDYYKFDYEFAEPPRVTSLQNTVPLATFSDFGDDVYFVAD
Pd-Vv1     MVIDYYLCDYESAEPPRATSLLNSEPSSTYSNFGEDSYFVSD
Pa-Amt2    MAIDYYTYDYEFAEPPRITSLQNTEPLPTFANFGEDLNFVAD
Pm-Ma1     MIVDYYSYDYEFAEPPRVTSLQNTVPLPTFDDFGDNVYFVAD
Pd-Vv2     MAIDYIAYDYEFSEPPRVTSLKNSIPLHTFSKFGEDAYFVAD

Pl-Sm2     MCLEYQNYDFEFAEPPRVTSLENTHPNPTFRDFGDDEYFVAD
Pl-Sm1     MVLDYYNYDYEFAEPPRVTSLKNTQPNPTFHNFGDSNFLVAD
Pa-Amt3    IAVDYLSYDAEIAEPPRITSLKNVEPISTMSYYGEDEQFVAD
```

AtPAO1-like

```
P-Amt1     LAIDYILHDFEMAE---------VEPIATYTEFGEREVLVAD
Pd-At1     LAIDFILHDFEMAE---------VEPISTYVDFGEREFLVAD
Pd-St1     LAIDFFLHDFEMAE---------VEPISTYVDFGEREFLVAD
Pd-Gm3     LAIDFILHDFEMAE---------VEPISTYVDFGEREFLVAD
Pd-Mt1     LAIDFILHDFEMAE---------VEPISTYVDFGEREFLVAD
Pd-Vv4     LAIDFILHDFEMAE---------VEPISTFLEFGEREYLVAD
Pd-Cc2     LAIDFILHDFEMAE---------VEPISTYVDFGEREFLVAD
Pd-Tc1     LAIDFILHDFEMAE---------VEPISTYVDFGEREFLVAD
Pd-Cs5     LAIDFILHDFEMPE---------VEPISTYLDFGEREFLVAD
Pd-Rc1     LAIDFILHDFEMAE---------VEPISTYVDFGEREFLVAD
```

Supplementary Figure S1. Sequence alignment in the region of peptide A of representative AtPAO1-like and ZmPAO1-like proteins from various plant species. Amino acids of peptide A are shown in red. Arrow indicates the position of the intron in respect to the amino acid sequence. E170 of ZmPAO1 (numbering of ZmPAO1 mature protein) is shown in green.
