# Supplementary material for: The tree of life of polyamine oxidases
Source: Sci Rep. 2020 Oct 20;10:17858. doi: 10.1038/s41598-020-74708-3 (PMC7576179; doi:10.1038/s41598-020-74708-3)
Supplement: Supplementary file 4 — Supplementary Table S2 [file 41598_2020_74708_MOESM4_ESM.pdf]

Scientific Reports

The Tree of Life of Polyamine Oxidases

Daniele Salvi[1] and Paraskevi Tavladoraki[2,*]

1 Department of Health, Life & Environmental Sciences, University of L'Aquila, 67100 L'Aquila, Italy.
2 Department of Science, University 'Roma Tre', 00146 Rome, Italy.
*Corresponding author: Paraskevi Tavladoraki, paraskevi.tavladoraki@uniroma3.it

Supplementary Table S2. Summary of results from BLAST searches of PAO-like proteins targeting, for each eukaryotic supergroup, selected species with available genomic resources ('PAO query result = No' means that the PAO query returned no BLAST hits).

Domain	Supergroups	Species	PAO query
Archaea	Asgard group	Asgard group	No
Archaea	Euryarchaeota	*Euryarchaeota archaeon*	Yes
Archaea	Euryarchaeota	*Halorubrum vacuolatum*	Yes
Archaea	Euryarchaeota	*Natronolimnobius innermongolicus*	Yes
Archaea	Euryarchaeota	*Natronolimnobius* sp.	Yes
Archaea	Thaumarchaeota	*Candidatus Nitrosotalea sinensis*	Yes
Eukaryotes	Alveolates, ciliates	*Tetrahymena thermophila* SB210	Yes
Eukaryotes	Alveolates, dinoflagellates	*Symbiodinium microadriaticum*	Yes
Eukaryotes	Amoebozoans	*Acanthamoeba astronyxis*	No
Eukaryotes	Amoebozoans	*Acanthamoeba castellanii*	No
Eukaryotes	Amoebozoans	*Acanthamoeba comandoni*	No
Eukaryotes	Amoebozoans	*Acanthamoeba lenticulata*	No
Eukaryotes	Amoebozoans	*Acanthamoeba lugdunensis*	No
Eukaryotes	Amoebozoans	*Acanthamoeba quina*	No
Eukaryotes	Amoebozoans	*Acanthamoeba rhysodes*	No
Eukaryotes	Amoebozoans	*Acanthamoeba royreba*	No
Eukaryotes	Amoebozoans	*Acytostelium leptosomum*	No
Eukaryotes	Amoebozoans	*Acytostelium subglobosum*	No
Eukaryotes	Amoebozoans	*Balamuthia mandrillaris*	No
Eukaryotes	Amoebozoans	*Cavenderia deminutiva*	No
Eukaryotes	Amoebozoans	*Cavenderia fasciculata*	No
Eukaryotes	Amoebozoans	*Cavenderia parvispora*	Yes
Eukaryotes	Amoebozoans	*Coremiostelium polycephalum*	No
Eukaryotes	Amoebozoans	*Dictyostelium citrinum*	No
Eukaryotes	Amoebozoans	*Dictyostelium discoideum*	No
Eukaryotes	Amoebozoans	*Dictyostelium firmibasis*	No
Eukaryotes	Amoebozoans	*Dictyostelium intermedium*	No
Eukaryotes	Amoebozoans	*Dictyostelium purpureum*	No
Eukaryotes	Amoebozoans	*Entamoeba dispar*	No
Eukaryotes	Amoebozoans	*Entamoeba histolytica*	No
Eukaryotes	Amoebozoans	*Entamoeba histolytica*	No
Eukaryotes	Amoebozoans	*Entamoeba invadens*	No
Eukaryotes	Amoebozoans	*Entamoeba moshkovskii*	No
Eukaryotes	Amoebozoans	*Entamoeba nuttalli*	No
Eukaryotes	Amoebozoans	*Heterostelium album*	No
Eukaryotes	Amoebozoans	*Heterostelium multicystogenum*	No
Eukaryotes	Amoebozoans	*Mastigamoeba balamuthi*	No
Eukaryotes	Amoebozoans	*Paramoeba pemaquidensis*	No
Eukaryotes	Amoebozoans	*Physarum polycephalum*	No
Eukaryotes	Amoebozoans	*Planoprotostelium fungivorum*	No
Eukaryotes	Amoebozoans	*Polysphondylium violaceum*	No
Eukaryotes	Amoebozoans	*Protostelium mycophagum*	No
Eukaryotes	Amoebozoans	*Rostrostelium ellipticum*	No
Eukaryotes	Amoebozoans	*Speleostelium caveatum*	No
Eukaryotes	Amoebozoans	*Synstelium polycarpum*	No
Eukaryotes	Amoebozoans	*Tieghemostelium lacteum*	No
Eukaryotes	Amoebozoans	*Vannella* sp.	Yes
Eukaryotes	Centrohelids	*Raphidiophrys*	No
Eukaryotes	Centrohelids	*Raphidiophrys ambigua*	No
Eukaryotes	Centrohelids	*Raphidiophrys contractilis*	No
Eukaryotes	Cryptista	*Guillardia theta* CCMP2712	Yes
Eukaryotes	Cryptista	*Palpitomonas bilix*	No
Eukaryotes	Excavates, euglenids	*Angomonas deanei*	No
Eukaryotes	Excavates, euglenids	*Crithidia acanthocephali*	No
Eukaryotes	Excavates, euglenids	*Crithidia fasciculata*	No
Eukaryotes	Excavates, euglenids	*Crithidia mellificae*	No
Eukaryotes	Excavates, euglenids	*Diplonema papillatum*	No
Eukaryotes	Excavates, euglenids	*Endotrypanum monterogeii*	No
Eukaryotes	Excavates, euglenids	*Euglena gracilis*	No
Eukaryotes	Excavates, euglenids	*Leishmania*	No
Eukaryotes	Excavates, euglenids	*Leishmania amazonensis*	No
Eukaryotes	Excavates, euglenids	*Leishmania donovani*	No
Eukaryotes	Excavates, euglenids	*Leishmania donovani*	No
Eukaryotes	Excavates, euglenids	*Leishmania major*	No
Eukaryotes	Excavates, euglenids	*Phytomonas*	No
Eukaryotes	Excavates, euglenids	*Strigomonas oncopelti*	No
Eukaryotes	Excavates, euglenids	*Trypanosoma brucei*	No
Eukaryotes	Excavates, euglenids	*Trypanosoma cruzi*	No
Eukaryotes	Excavates, heteroloboseans	*Naegleria gruberi* strain NEG-M	Yes
Eukaryotes	Glaucophytes	*Cyanophora paradoxa*	No
Eukaryotes	Glaucophytes	*Paulinella chromatophora*	No
Eukaryotes	Green algae	*Auxenochlorella protothecoides*	Yes
Eukaryotes	Green algae	*Chlamydomonas eustigma*	Yes
Eukaryotes	Green algae	*Chlorella sorokiniana*	Yes
Eukaryotes	Green algae	*Chlorella variabilis*	Yes
Eukaryotes	Green algae	*Coccomyxa subellipsoidea*	Yes
Eukaryotes	Green algae	*Micractinium conductrix*	Yes
Eukaryotes	Green algae	*Tetrabaena socialis*	Yes
Eukaryotes	Green algae	*Trebouxia* sp. A1-2	Yes
Eukaryotes	Haptophytes	*Chrysochromulina parva*	No
Eukaryotes	Haptophytes	*Chrysochromulina tobinii*	Yes
Eukaryotes	Haptophytes	*Emiliania huxleyi*	No
Eukaryotes	Metamonads	*Giardia intestinalis*	No
Eukaryotes	Metamonads	*Giardia muris*	No
Eukaryotes	Metamonads	*Kipferlia bialata*	No
Eukaryotes	Metamonads	*Monocercomonoides*	No
Eukaryotes	Metamonads	*Spironucleus salmonicida*	No
Eukaryotes	Metamonads	*Streblomastix strix*	No
Eukaryotes	Metamonads	*Trichomonas gallinae*	No
Eukaryotes	Metamonads	*Trichomonas tenax*	No
Eukaryotes	Metamonads	*Trichomonas vaginalis*	No
Eukaryotes	Metamonads	*Tritrichomonas foetus*	No
Eukaryotes	Red algae	*Chondrus crispus*	Yes
Eukaryotes	Red algae	*Cyanidioschyzon merolae*	No
Eukaryotes	Red algae	*Cyanidioschyzon merolae*	No
Eukaryotes	Red algae	*Cyanidioschyzon merolae* strain 10D	No
Eukaryotes	Red algae	*Digenea simplex*	No
Eukaryotes	Red algae	*Galdieria phlegrea*	No
Eukaryotes	Red algae	*Galdieria sulphuraria*	Yes
Eukaryotes	Red algae	*Gracilariopsis lemaneiformis*	No
Eukaryotes	Red algae	*Kappaphycus alvarezii*	Yes
Eukaryotes	Red algae	*Porphyra umbilicalis*	Yes
Eukaryotes	Red algae	*Porphyridium purpureum*	Yes
Eukaryotes	Red algae	*Porphyridium purpureum*	Yes
Eukaryotes	Red algae	*Pyropia haitanensis*	Yes
Eukaryotes	Red algae	*Pyropia yezoensis*	No
Eukaryotes	Rhizarians	*Astrammina rara*	No
Eukaryotes	Rhizarians	*Bigelowiella natans*	No
Eukaryotes	Rhizarians	*Globobulimina*	No
Eukaryotes	Rhizarians	*Lotharella oceanica*	No
Eukaryotes	Rhizarians	*Plasmodiophora brassicae*	Yes
Eukaryotes	Rhizarians	*Plasmodiophora brassicae*	No
Eukaryotes	Rhizarians	*Polymyxa betae*	No
Eukaryotes	Rhizarians	*Reticulomyxa filosa*	No
Eukaryotes	Rhizarians	*Spongospora subterranea*	No
Eukaryotes	Stramenopiles, diatoms	*Fragilariopsis cylindrus* CCMP1102	Yes
Eukaryotes	Stramenopiles, diatoms	*Phaeodactylum tricornutum* CCAP 1055/1	Yes
Eukaryotes	Stramenopiles, oomycetes	*Phytophthora nicotianae*	Yes
