# Supplementary material for: The tree of life of polyamine oxidases
Source: Sci Rep. 2020 Oct 20;10:17858. doi: 10.1038/s41598-020-74708-3 (PMC7576179; doi:10.1038/s41598-020-74708-3)
Supplement: Supplementary file 5 — Supplementary Table S3 [file 41598_2020_74708_MOESM5_ESM.pdf]

Scientific Reports

The Tree of Life of Polyamine Oxidases

Daniele Salvi[1] and Paraskevi Tavladoraki[2,*]

1 Department of Health, Life & Environmental Sciences, University of L'Aquila, 67100 L'Aquila, Italy.

2 Department of Science, University 'Roma Tre', 00146 Rome, Italy.

*Corresponding author: Paraskevi Tavladoraki, paraskevi.tavladoraki@uniroma3.it

Supplementary Table S3. Details on numbers of sequences, sites, and informative sites for each dataset analysed in this study.

Dataset	Number of sequences	Number of sites	Number of informative sites
Total	300	1738	1032
Eukaryote clade	253	1661	939
AtPAO1-like (Clade I)	81	749	478
AtPAO5-like (Clade II)	59	713	497
AtPAO2,3,4-like (Clade IV)	100	619	356
