# Supplementary material for: The tree of life of polyamine oxidases
Source: Sci Rep. 2020 Oct 20;10:17858. doi: 10.1038/s41598-020-74708-3 (PMC7576179; doi:10.1038/s41598-020-74708-3)
Supplement: Supplementary file 3 — Supplementary Table S1 [file 41598_2020_74708_MOESM3_ESM.pdf]

Scientific Reports

The Tree of Life of Polyamine Oxidases

Daniele Salvi[1] and Paraskevi Tavladoraki[2,*]

1 Department of Health, Life & Environmental Sciences, University of L'Aquila, 67100 L'Aquila, Italy.
2 Department of Science, University 'Roma Tre', 00146 Rome, Italy.
*Corresponding author: Paraskevi Tavladoraki, paraskevi.tavladoraki@uniroma3.it

Supplementary Table S1. Polyamine oxidase protein sequences used in this study (ordered alphabetically by taxonomy). Codes and sequences used in phylogenetic trees (Figs. 1-5) are reported.

CODE	Taxonomy	Species	Clade	Genbank accession number	Phylogeny Three-domains (Fig. 1)	Phylogeny Eukaryota clade (Fig. 2)	Phylogeny Land Plants Clade I (Fig. 3)	Phylogeny Land Plants Clade II (Fig. 4)	Phylogeny Land Plants Clade IV (Fig. 5)
ARe-Ea1	Archaea — Euryarchaeota	*Euryarchaeota archaeon*	Eukaryota — Clade IV	OGS63392	Yes	Yes	—	—	—
ARe-Ea2	Archaea — Euryarchaeota	*Euryarchaeota archaeon*	Eukaryota — Clade IV	OGS47722	Yes	Yes	—	—	—
ARe-Ea3	Archaea — Euryarchaeota	*Euryarchaeota archaeon*	Eukaryota — Clade IV	TLZ75006	Yes	Yes	—	—	—
ARe-Hv	Archaea — Euryarchaeota	*Halorubrum vacuolatum*	Archaea	WP089384808	Yes	—	—	—	—
ARe-Ni3	Archaea — Euryarchaeota	*Natronolimnobius innermongolicus*	Archaea	WP049889138	Yes	—	—	—	—
ARe-Ns1	Archaea — Euryarchaeota	*Natronolimnobius* sp.	Archaea	WP165163700	Yes	—	—	—	—
ARe-Ns2	Archaea — Euryarchaeota	*Natronolimnobius* sp.	Archaea	WP165162801	Yes	—	—	—	—
ARe-Ha	Archaea — Euryarchaeota	halophilic archaeon DL31	Archaea	WP014053004.1	Yes	—	—	—	—
ARe-Hn	Archaea — Euryarchaeota	*Halobiforma nitratireducens*	Archaea	WP006672637.1	Yes	—	—	—	—
ARe-Na	Archaea — Euryarchaeota	*Natronococcus amylolyticus*	Archaea	WP005553718.1	Yes	—	—	—	—
ARe-Nc	Archaea — Euryarchaeota	*Natrialba chahannaoensis*	Archaea	WP006169598.1	Yes	—	—	—	—
ARe-Ng	Archaea — Euryarchaeota	*Natronobacterium gregoryi*	Archaea	WP005581379.1	Yes	—	—	—	—
ARe-Ni1	Archaea — Euryarchaeota	*Natronolimnobius innermongolicus*	Archaea	ELY59648.1	Yes	—	—	—	—
ARe-Ni2	Archaea — Euryarchaeota	*Natronolimnobius innermongolicus*	Archaea	WP007258153.1	Yes	—	—	—	—
ARe-Nm	Archaea — Euryarchaeota	*Natrialba magadii*	Archaea	WP004267159.1	Yes	—	—	—	—
ARe-No	Archaea — Euryarchaeota	*Natronococcus occultus*	Archaea	WP015319894.1	Yes	—	—	—	—
Bac-Kv	Bacteria — Acidobacteria	*Koribacter versatilis*	Bacteria	ABF40635.1	Yes	—	—	—	—
Bat-Re[1]	Bacteria — Actinobacteria	*Rhodococcus erythropolis*	Archaea	WP019744737.1	Yes	—	—	—	—
Bat-Sr	Bacteria — Actinobacteria	*Streptosporangium roseum*	Bacteria	WP012887142.1	Yes	—	—	—	—
Bg-Ha	Bacteria — Chloroflexi	*Herpetosiphon aurantiacus*	Eukaryota — Clade IV	ABX03967.1	Yes	Yes	—	—	—
Bg-Rc	Bacteria — Chloroflexi	*Roseiflexus castenholzii*	Eukaryota — Clade IV	ABU58160.1	Yes	Yes	—	—	—
Bcy-Ca1	Bacteria — Cyanobacteria	*Cyanobacterium aponinum*	Eukaryota — Clade IV	WP015218588.1	Yes	Yes	—	—	—
Bcy-Ma	Bacteria — Cyanobacteria	*Microcystis aeruginosa*	Eukaryota — Clade IV	WP002773540.1	Yes	Yes	—	—	—
Bcy-Oc	Bacteria — Cyanobacteria	*Oscillatoriales cyanobacterium*	Eukaryota — Clade IV	WP009768362.1	Yes	Yes	—	—	—
Bcy-Sy	Bacteria — Cyanobacteria	*Synechocystis*	Eukaryota — Clade IV	WP011153630.1	Yes	Yes	—	—	—
Bd-Ms	Bacteria — Deinococcus-Thermus	*Meiothermus silvanus*	Bacteria	WP013159446.1	Yes	—	—	—	—
Bp-Ah	Bacteria — Proteobacteria	*Aeromonas hydrophila*	Bacteria	WP005302938.1	Yes	—	—	—	—
Bp-Al	Bacteria — Proteobacteria	*Alcanivorax* sp.	Bacteria	WP008734800.1	Yes	—	—	—	—
Bp-As	Bacteria — Proteobacteria	*Aeromonas salmonicida*	Bacteria	WP005312693.1	Yes	—	—	—	—
Bp-Cl	Bacteria — Proteobacteria	*Congregibacter litoralis*	Bacteria	WP008295487.1	Yes	—	—	—	—
Bp-Cml	Bacteria — Proteobacteria	*Campylobacter lari*	Bacteria	WP012661782.1	Yes	—	—	—	—
Bp-Cn	Bacteria — Proteobacteria	*Cupriavidus necator*	Bacteria	WP011615168.1	Yes	—	—	—	—
Bp-Cp	Bacteria — Proteobacteria	*Cupriavidus pinatubonensis*	Bacteria	WP011297623.1	Yes	—	—	—	—
Bp-Cs	Bacteria — Proteobacteria	*Cronobacter sakazakii*	Bacteria	WP014728939.1	Yes	—	—	—	—
Bp-Cv	Bacteria — Proteobacteria	*Chromobacterium violaceum*	Bacteria	WP011136602.1	Yes	—	—	—	—
Bp-Eb	Bacteria — Proteobacteria	*Erwinia billingiae*	Bacteria	WP013202347.1	Yes	—	—	—	—
Bp-En	Bacteria — Proteobacteria	*Enterobacter sp.*	Bacteria	WP008459642.1	Yes	—	—	—	—
Bp-Et	Bacteria — Proteobacteria	*Edwardsiella tarda*	Eukaryota — Clade IV	WP005286802.1	Yes	Yes	—	—	—
Bp-Ha	Bacteria — Proteobacteria	*Hafnia alvei*	Bacteria	WP004092198.1	Yes	—	—	—	—
Bp-HdN1	Bacteria — Proteobacteria	gamma proteobacterium HdN1	Bacteria	WP013262386.1	Yes	—	—	—	—
Bp-HTCC	Bacteria — Proteobacteria	marine gamma proteobact HTCC2080	Bacteria	WP007235554.1	Yes	—	—	—	—
Bp-Ls	Bacteria — Proteobacteria	*Luminiphilus syltensis* NOR5-1B	Bacteria	WP009020723.1	Yes	—	—	—	—
Bp-Nn	Bacteria — Proteobacteria	*Novosphingobium nitrogenifigens*	Bacteria	WP008069558.1	Yes	—	—	—	—
Bp-NOR5	Bacteria — Proteobacteria	*gamma proteobacterium* NOR5-3	Bacteria	WP009022399.1	Yes	—	—	—	—
Bp-Pf	Bacteria — Proteobacteria	*Pseudomonas fluorescens*	Bacteria	WP003209555.1	Yes	—	—	—	—
Bp-Pp	Bacteria — Proteobacteria	*Pseudomonas putida*	Bacteria	WP012273323.1	Yes	—	—	—	—
Bp-Rg	Bacteria — Proteobacteria	*Rubrivivax gelatinosus*	Bacteria	WP014426774.1	Yes	—	—	—	—
Bp-Sf	Bacteria — Proteobacteria	*Shigella flexneri*	Bacteria	EIQ79912.1	Yes	—	—	—	—
Bp-So	Bacteria — Proteobacteria	*Serratia plymuthica*	Bacteria	WP004952114.1	Yes	—	—	—	—
Bp-Sp	Bacteria — Proteobacteria	*Serratia proteamaculans*	Bacteria	WP012146348.1	Yes	—	—	—	—
Bp-SpdH	Bacteria — Proteobacteria	*Pseudomonas aeruginosa*	Bacteria	WP003113842.1	Yes	—	—	—	—
Bp-Yer	Bacteria — Proteobacteria	*Yersinia ruckeri*	Bacteria	WP004721820.1	Yes	—	—	—	—
Bp-Yor	Bacteria — Proteobacteria	*Yokenella regensburgei*	Bacteria	WP006816923.1	Yes	—	—	—	—
ALc-Tt1	Eukaryotes — Alveolates, ciliates	*Tetrahymena thermophila* SB210	Eukaryota — Clade II	XP001032538	Yes	Yes	—	—	—
ALc-Tt2	Eukaryotes — Alveolates, ciliates	*Tetrahymena thermophila* SB210	Eukaryota — Clade II	XP001017978	Yes	Yes	—	—	—
ALc-Tt4	Eukaryotes — Alveolates, ciliates	*Tetrahymena thermophila* SB210	Eukaryota — Clade II	XP001013248	Yes	Yes	—	—	—
ALd-Sm	Eukaryotes — Alveolates, dinoflagellates	*Symbiodinium microadriaticum*	Eukaryota — Clade II	OLQ06107	Yes	Yes	—	—	—
AMd-Va	Eukaryotes — Amoebozoans, discosea	*Vannella* sp.	Eukaryota — Clade IV	IACZ01008817	Yes	Yes	—	—	—
AMm-Cp	Eukaryotes — Amoebozoans, mycetozoa	*Cavenderia parvispora*	Eukaryota — Clade IV	GIOM01030114	Yes	Yes	—	—	—
CR-Gt2	Eukaryotes — Cryptista, cryptophytes	*Guillardia theta* CCMP2712	Eukaryota — Clade IV	XP005834025	Yes	Yes	—	—	—
CR-Gt3	Eukaryotes — Cryptista, cryptophytes	*Guillardia theta* CCMP2712	Eukaryota	XP005830446	Yes	—	—	—	—
Eh-Ng	Eukaryotes — Excavates, heteroloboseans	*Naegleria gruberi strain NEG-M*	Eukaryota — Clade II	XP002681036	Yes	Yes	—	—	—
Fa-Ac1	Eukaryotes — Fungi, ascomycetes	*Aspergillus clavatus*	Eukaryota — Clade III	XP001271211.1	Yes	Yes	—	—	—
Fa-Ac2	Eukaryotes — Fungi, ascomycetes	*Aspergillus clavatus*	Eukaryota — Clade I	XP001274297.1	Yes	Yes	—	—	—
Fa-Af1	Eukaryotes — Fungi, ascomycetes	*Aspergillus flavus*	Eukaryota — Clade I	XP002384541.1	Yes	Yes	—	—	—
Fa-Af2	Eukaryotes — Fungi, ascomycetes	*Aspergillus flavus*	Eukaryota — Clade III	XP002374550.1	Yes	Yes	—	—	—
Fa-Ag	Eukaryotes — Fungi, ascomycetes	*Microsporum gypseum*	Eukaryota — Clade I	XP003176013.1	Yes	Yes	—	—	—
Fa-An1	Eukaryotes — Fungi, ascomycetes	*Aspergillus niger*	Eukaryota — Clade I	CAK39871.1	Yes	Yes	—	—	—
Fa-An2	Eukaryotes — Fungi, ascomycetes	*Aspergillus niger*	Eukaryota — Clade I	XP001396385.1	Yes	Yes	—	—	—
Fa-An3	Eukaryotes — Fungi, ascomycetes	*Aspergillus niger*	Eukaryota — Clade I	XP001393570.2	Yes	Yes	—	—	—
Fa-An4	Eukaryotes — Fungi, ascomycetes	*Aspergillus niger*	Eukaryota — Clade III	XP001399959.2	Yes	Yes	—	—	—
Fa-Ao	Eukaryotes — Fungi, ascomycetes	*Aspergillus oryzae*	Eukaryota — Clade III	EIT76752.1	Yes	Yes	—	—	—
Fa-Aro	Eukaryotes — Fungi, ascomycetes	*Arthroderma otae*	Eukaryota — Clade I	XP002847427.1	Yes	Yes	—	—	—
Fa-Aso	Eukaryotes — Fungi, ascomycetes	*Aspergillus oryzae*	Eukaryota — Clade I	Q2TX03; XP001827353.1	Yes	Yes	—	—	—
Fa-At	Eukaryotes — Fungi, ascomycetes	*Aspergillus terreus*	Eukaryota — Clade I	XP001209815.1	Yes	Yes	—	—	—
Fa-Bf1	Eukaryotes — Fungi, ascomycetes	*Botrytis cinerea* T4	Eukaryota — Clade III	CCD54241.1	Yes	Yes	—	—	—
Fa-Cg	Eukaryotes — Fungi, ascomycetes	*Colletotrichum gloeosporioides*	Eukaryota — Clade III	XP007285234.1	Yes	Yes	—	—	—
Fa-Ch	Eukaryotes — Fungi, ascomycetes	*Colletotrichum higginsianum*	Eukaryota — Clade III	CCF46224.1	Yes	Yes	—	—	—
Fa-Co	Eukaryotes — Fungi, ascomycetes	*Colletotrichum orbiculare*	Eukaryota — Clade III	ENH86630.1	Yes	Yes	—	—	—
Fa-Ff1	Eukaryotes — Fungi, ascomycetes	*Fusarium fujikuroi*	Eukaryota — Clade III	CCT76192.1	Yes	Yes	—	—	—
Fa-Ff2	Eukaryotes — Fungi, ascomycetes	*Fusarium fujikuroi*	Eukaryota — Clade I	CCT75728.1	Yes	Yes	—	—	—
Fa-Fo1	Eukaryotes — Fungi, ascomycetes	*Fusarium oxysporum*	Eukaryota — Clade I	EMT74311.1	Yes	Yes	—	—	—
Fa-Fo2	Eukaryotes — Fungi, ascomycetes	*Fusarium oxysporum*	Eukaryota — Clade I	EMT68980.1	Yes	Yes	—	—	—
Fa-Fo3	Eukaryotes — Fungi, ascomycetes	*Fusarium oxysporum*	Eukaryota — Clade I	EMT69436.1	Yes	Yes	—	—	—
Fa-Ma	Eukaryotes — Fungi, ascomycetes	*Metarhizium anisopliae*	Eukaryota — Clade I	KFG79001.1	Yes	Yes	—	—	—
Fa-Nf1	Eukaryotes — Fungi, ascomycetes	*Neosartorya fischeri*	Eukaryota — Clade I	XP001259716.1	Yes	Yes	—	—	—
Fa-Nf2	Eukaryotes — Fungi, ascomycetes	*Neosartorya fischeri*	Eukaryota — Clade III	XP001257555.1	Yes	Yes	—	—	—
Fa-Nf3	Eukaryotes — Fungi, ascomycetes	*Neosartorya fischeri*	Eukaryota — Clade III	XP001263250.1	Yes	Yes	—	—	—
Fa-Nfu1	Eukaryotes — Fungi, ascomycetes	*Neosartorya fumigata*	Eukaryota — Clade I	EDP47631.1	Yes	Yes	—	—	—
Fa-Nfu2	Eukaryotes — Fungi, ascomycetes	*Neosartorya fumigata*	Eukaryota — Clade III	EDP52537.1	Yes	Yes	—	—	—
Fa-Nh1	Eukaryotes — Fungi, ascomycetes	*Nectria haematococca*	Eukaryota — Clade I	XP003041352.1	Yes	Yes	—	—	—
Fa-Pc	Eukaryotes — Fungi, ascomycetes	*Penicillium chrysogenum*	Eukaryota — Clade I	XP002562069.1	Yes	Yes	—	—	—
Fa-Pd	Eukaryotes — Fungi, ascomycetes	*Penicillium digitatum*	Eukaryota — Clade I	XP014531555.1	Yes	Yes	—	—	—
Fa-Pn	Eukaryotes — Fungi, ascomycetes	*Phaeosphaeria nodorum* SN15	Eukaryota — Clade I	EAT91993.2; XP001791182.1	Yes	Yes	—	—	—
Fa-Pt	Eukaryotes — Fungi, ascomycetes	*Pyrenophora tritici/repentis*	Eukaryota — Clade I	XP001940957.1	Yes	Yes	—	—	—
Fa-Sc	Eukaryotes — Fungi, ascomycetes	*Sclerotinia sclerotiorum* 1980	Eukaryota — Clade III	XP001591821.1	Yes	Yes	—	—	—
Fa-Te	Eukaryotes — Fungi, ascomycetes	*Trichophyton equinum*	Eukaryota — Clade I	EGD95686.1	Yes	Yes	—	—	—
Fa-Tr	Eukaryotes — Fungi, ascomycetes	*Trichophyton rubrum*	Eukaryota — Clade I	XP003239450.1	Yes	Yes	—	—	—
Fa-Tt	Eukaryotes — Fungi, ascomycetes	*Trichophyton tonsurans*	Eukaryota — Clade I	EGD95686.1	Yes	Yes	—	—	—
Fa-Vd	Eukaryotes — Fungi, ascomycetes	*Verticillium dahliae*	Eukaryota — Clade I	XP009651749.1	Yes	Yes	—	—	—
Fa-Zt	Eukaryotes — Fungi, ascomycetes	*Zymoseptoria tritici isolate* IPO323	Eukaryota — Clade III	XP003851688.1	Yes	Yes	—	—	—
Fay-Cb1	Eukaryotes — Fungi, ascomycetes yeast	*Candida dubliniensis*	Eukaryota — Clade III	XP002419858.1	Yes	Yes	—	—	—
Fay-Cb2	Eukaryotes — Fungi, ascomycetes yeast	*Candida albicans*	Eukaryota — Clade III	XP716457.1	Yes	Yes	—	—	—
Fay-Cp	Eukaryotes — Fungi, ascomycetes yeast	*Candida parapsilosis*	Eukaryota — Clade III	CCE45127.1	Yes	Yes	—	—	—
Fay-Ct	Eukaryotes — Fungi, ascomycetes yeast	*Candida tenuis*	Eukaryota — Clade III	XP006684827.1	Yes	Yes	—	—	—
Fay-Dh1	Eukaryotes — Fungi, ascomycetes yeast	*Debaryomyces hansenii*	Eukaryota — Clade III	BAF93947.2	Yes	Yes	—	—	—
Fay-Dh2	Eukaryotes — Fungi, ascomycetes yeast	*Debaryomyces hansenii*	Eukaryota — Clade III	XP456450.2; CAG84402.2	Yes	Yes	—	—	—
Fay-FMS1	Eukaryotes — Fungi, ascomycetes yeast	*Saccharomyces cerevisiae*	Eukaryota — Clade III	NP013733.1	Yes	Yes	—	—	—
Fay-Pa	Eukaryotes — Fungi, ascomycetes yeast	*Pichia angusta*	Eukaryota — Clade III	XP013933775.1	Yes	Yes	—	—	—
Fay-Ps	Eukaryotes — Fungi, ascomycetes yeast	*Pichia farinosa*	Eukaryota — Clade III	XP004198801.1	Yes	Yes	—	—	—
Fay-Ss	Eukaryotes — Fungi, ascomycetes yeast	*Scheffersomyces stipitis*	Eukaryota — Clade III	XP001387500.2	Yes	Yes	—	—	—
Fb-Cf	Eukaryotes — Fungi, basidomycetes	*Cryptococcus neoformans var. grubii*	Eukaryota — Clade III	XP012052396.1	Yes	Yes	—	—	—
Fb-Cn1	Eukaryotes — Fungi, basidomycetes	*Cryptococcus neoformans var. grubii*	Eukaryota — Clade III	XP012052396.1	Yes	Yes	—	—	—
Fb-Cn2	Eukaryotes — Fungi, basidomycetes	*Cryptococcus neoformans*	Eukaryota — Clade III	XP776369.1	Yes	Yes	—	—	—
Fb-Gt1	Eukaryotes — Fungi, basidomycetes	*Gloeophyllum trabeum*	Eukaryota — Clade III	XP007861232.1	Yes	Yes	—	—	—
Fb-Hi	Eukaryotes — Fungi, basidomycetes	*Heterobasidion irregulare* TC32-1	Eukaryota — Clade I	ETW85406.1; XP009541770.1	Yes	Yes	—	—	—
Fb-Ml1	Eukaryotes — Fungi, basidomycetes	*Melampsora larici/populina*	Eukaryota — Clade III	XP007413802.1	Yes	Yes	—	—	—
Fb-Ml2	Eukaryotes — Fungi, basidomycetes	*Melampsora larici/populina*	Eukaryota — Clade III	XP007405934.1	Yes	Yes	—	—	—
Fb-Pg	Eukaryotes — Fungi, basidomycetes	*Puccinia graminis* f. sp. *tritici*	Eukaryota — Clade III	XP003332763.2	Yes	Yes	—	—	—
Fb-Sr	Eukaryotes — Fungi, basidomycetes	*Sporisorium reilianum*	Eukaryota — Clade III	CBQ70895.1	Yes	Yes	—	—	—
Fb-Uh	Eukaryotes — Fungi, basidomycetes	*Ustilago hordei*	Eukaryota — Clade III	CCF51619.1	Yes	Yes	—	—	—
Fb-Um1	Eukaryotes — Fungi, basidomycetes	*Ustilago maydis*	Eukaryota — Clade III	CBL29058.1	Yes	Yes	—	—	—
Fby-Rg	Eukaryotes — Fungi, basidomycetes yeast	*Rhodotorula glutinis*	Eukaryota — Clade III	EGU13497.1	Yes	Yes	—	—	—
Fby-Rt	Eukaryotes — Fungi, basidomycetes yeast	*Rhodosporidium toruloides*	Eukaryota — Clade III	EMS19074.1	Yes	Yes	—	—	—
Fby-Ta	Eukaryotes — Fungi, basidomycetes yeast	*Trichosporon asahii*	Eukaryota — Clade III	EKD03658.1	Yes	Yes	—	—	—
Fz-Mc1	Eukaryotes — Fungi, zygomycetes	*Mucor circinelloides*	Eukaryota — Clade I	EPB87491.1	Yes	Yes	—	—	—
Fz-Rd	Eukaryotes — Fungi, zygomycetes	*Rhizopus delemar*	Eukaryota — Clade I	EIE89983.1	Yes	Yes	—	—	—
Clo-Cr	Eukaryotes — Green algae, Chlorophyceae	*Chlamydomonas reinhardtii*	Eukaryota — Clade IV	PNW77697.1	Yes	Yes	—	—	—
Clo-Ts1	Eukaryotes — Green algae, chlorophyceae	*Tetrabaena socialis*	Eukaryota — Clade IV	PNH12967	Yes	Yes	—	—	—
Clo-Vc	Eukaryotes — Green algae, Chlorophyceae	*Volvox carteri*	Eukaryota — Clade IV	XP002957050.1	Yes	Yes	—	—	—
Ct-Ap	Eukaryotes — Green algae, trebouxiophyceae	*Auxenochlorella protothecoides*	Eukaryota — Clade II	XP011398363	Yes	Yes	—	—	—
Ct-Cos	Eukaryotes — Green algae, trebouxiophyceae	*Coccomyxa subellipsoidea*	Eukaryota — Clade II	XP005650275	Yes	Yes	—	—	—
Ct-Cs1	Eukaryotes — Green algae, trebouxiophyceae	*Chlorella sorokiniana*	Eukaryota — Clade II	PRW56804	Yes	Yes	—	—	—
Ct-Cs2	Eukaryotes — Green algae, trebouxiophyceae	*Chlorella sorokiniana*	Eukaryota — Clade IV	PRW58489	Yes	Yes	—	—	—
Ct-Cv1	Eukaryotes — Green algae, trebouxiophyceae	*Chlorella variabilis*	Eukaryota — Clade IV	XP005851294	Yes	Yes	—	—	—
Ct-Cv2	Eukaryotes — Green algae, trebouxiophyceae	*Chlorella variabilis*	Eukaryotes	XP005843020	Yes	—	—	—	—
Ct-Cv3	Eukaryotes — Green algae, trebouxiophyceae	*Chlorella variabilis*	Eukaryota — Clade II	XP005850098	Yes	Yes	—	—	—
Ct-Mc	Eukaryotes — Green algae, trebouxiophyceae	*Micractinium conductrix*	Eukaryota — Clade II	PSC70494	Yes	Yes	—	—	—
Ct-Te	Eukaryotes — Green algae, trebouxiophyceae	*Trebouxia* sp. A1-2	Eukaryota — Clade II	KAA6422138	Yes	Yes	—	—	—
Hh-Ct2	Eukaryotes — Haptista, haptophyta	*Chrysochromulina tobinii*	Eukaryota — Clade IV	KOO28044	Yes	Yes	—	—	—
Hh-Ct5	Eukaryotes — Haptista, haptophyta	*Chrysochromulina tobinii*	Eukaryota — Clade IV	KOO22211	Yes	Yes	—	—	—
Hh-Ct6	Eukaryotes — Haptista, haptophyta	*Chrysochromulina tobinii*	Eukaryota — Clade IV	KOO25806	Yes	Yes	—	—	—
Pa-Amt1	Eukaryotes — Land Plant, amborellales	*Amborella trichopoda*	Eukaryota — Clade I	XP006851065.1	—	—	Yes	—	—
Pa-Amt2	Eukaryotes — Land Plant, amborellales	*Amborella trichopoda*	Eukaryota — Clade I	XP006844936.1	—	—	Yes	—	—
Pa-Amt3	Eukaryotes — Land Plant, amborellales	*Amborella trichopoda*	Eukaryota — Clade I	XP006856901.2	—	—	Yes	—	—
Pa-Amt4	Eukaryotes — Land Plant, amborellales	*Amborella trichopoda*	Eukaryota — Clade IV	XP006848004.1	—	—	—	—	Yes
Pa-Amt5	Eukaryotes — Land Plant, amborellales	*Amborella trichopoda*	Eukaryota — Clade IV	XP006836334.1	—	—	—	—	Yes
Pa-Amt6	Eukaryotes — Land Plant, amborellales	*Amborella trichopoda*	Eukaryota — Clade II	XP006858310.1	—	—	—	Yes	0
Pb-Pp1	Eukaryotes — Land Plant, bryophytes	*Physcomitrella patens*	Eukaryota — Clade IV	XP001756864.1; XP024384528.1	Yes	Yes	—	—	Yes
Pb-Pp2	Eukaryotes — Land Plant, bryophytes	*Physcomitrella patens*	Eukaryota — Clade I	PNR34409.1	—	—	Yes	—	—
Pb-Pp3	Eukaryotes — Land Plant, bryophytes	*Physcomitrella patens*	Eukaryota — Clade I	PNR40749.1	—	—	Yes	—	—
Pb-Pp4	Eukaryotes — Land Plant, bryophytes	*Physcomitrella patens*	Eukaryota — Clade IV	XP024397670.1	—	—	—	—	Yes
Ph-Mp1	Eukaryotes — Land Plant, bryophytes hepatic	*Marchantia polymorpha*	Eukaryota — Clade I	BAS31067.1	—	—	Yes	—	—
Ph-Mp2	Eukaryotes — Land Plant, bryophytes hepatic	*Marchantia polymorpha*	Eukaryota — Clade I	BAS31068.1	—	—	Yes	—	—
Ph-Mp3	Eukaryotes — Land Plant, bryophytes hepatic	*Marchantia polymorpha*	Eukaryota — Clade I	BAS31069.1	—	—	Yes	—	—
Ph-Mp4	Eukaryotes — Land Plant, bryophytes hepatic	*Marchantia polymorpha*	Eukaryota — Clade IV	BAS31070.1	—	—	—	—	Yes
Ph-Mp5	Eukaryotes — Land Plant, bryophytes hepatic	*Marchantia polymorpha*	Eukaryota — Clade II	OAE35179.1	—	—	—	Yes	—
Pd-Ah1	Eukaryotes — Land Plant, eudicots	*Amaranthus hypochondriacus*	Eukaryota — Clade I	AAM43922.1	Yes	Yes	Yes	—	—
Pd-At1	Eukaryotes — Land Plant, eudicots	*Arabidopsis thaliana*	Eukaryota — Clade I	NP196874.1; AT5G13700	Yes	Yes	Yes	—	—
Pd-At2	Eukaryotes — Land Plant, eudicots	*Arabidopsis thaliana*	Eukaryota — Clade IV	AAL75899; ABI93934.1; At2g43020	Yes	Yes	—	—	Yes
Pd-At3	Eukaryotes — Land Plant, eudicots	*Arabidopsis thaliana*	Eukaryota — Clade IV	NP191464.1; AT3G59050	Yes	Yes	—	—	Yes
Pd-At4	Eukaryotes — Land Plant, eudicots	*Arabidopsis thaliana*	Eukaryota — Clade IV	NP176759.1; AT1G65840	Yes	Yes	—	—	Yes
Pd-At5	Eukaryotes — Land Plant, eudicots	*Arabidopsis thaliana*	Eukaryota — Clade II	NP194701.1; AT4G29720	Yes	Yes	—	Yes	—
Pd-Bj1	Eukaryotes — Land Plant, eudicots	*Brassica juncea*	Eukaryota — Clade II	AAO16558.1	Yes	Yes	—	Yes	—
Pd-Bn1	Eukaryotes — Land Plant, eudicots	*Brassica napus*	Eukaryota — Clade I	XP013727301.1	—	—	Yes	—	—
Pd-Bn2	Eukaryotes — Land Plant, eudicots	*Brassica napus*	Eukaryota — Clade I	XP013738149.1; XP013738149.2	—	—	Yes	—	—
Pd-Br1	Eukaryotes — Land Plant, eudicots	*Brassica rapa*	Eukaryota — Clade I	XP009131355.1	Yes	Yes	Yes	—	—
Pd-Br2	Eukaryotes — Land Plant, eudicots	*Brassica rapa*	Eukaryota — Clade IV	XP009142900.1	Yes	Yes	—	—	Yes
Pd-Br3	Eukaryotes — Land Plant, eudicots	*Brassica rapa*	Eukaryota — Clade IV	XP009142156.1	Yes	Yes	—	—	Yes
Pd-Br4	Eukaryotes — Land Plant, eudicots	*Brassica rapa*	Eukaryota — Clade IV	XP009133564.1	Yes	Yes	—	—	Yes
Pd-Br5	Eukaryotes — Land Plant, eudicots	*Brassica rapa*	Eukaryota — Clade IV	XP009104181.1	Yes	Yes	—	—	Yes
Pd-Br6	Eukaryotes — Land Plant, eudicots	*Brassica rapa*	Eukaryota — Clade IV	XP009127456.1	Yes	Yes	—	—	Yes
Pd-Br7	Eukaryotes — Land Plant, eudicots	*Brassica rapa*	Eukaryota — Clade II	XP009128530.1	Yes	Yes	—	Yes	—
Pd-Br8	Eukaryotes — Land Plant, eudicots	*Brassica rapa*	Eukaryota — Clade II	XP009137858.1	Yes	Yes	—	Yes	—
Pd-Bv1	Eukaryotes — Land Plant, eudicots	*Beta vulgaris vulgaris*	Eukaryota — Clade I	XP010696142.1	—	—	Yes	—	—
Pd-Ca1	Eukaryotes — Land Plant, eudicots	*Cicer arietinum*	Eukaryota — Clade II	XP004488041.1	Yes	Yes	—	Yes	—
Pd-Ca2	Eukaryotes — Land Plant, eudicots	*Cicer arietinum*	Eukaryota — Clade IV	XP004499541.1	Yes	Yes	—	—	Yes
Pd-Ca3	Eukaryotes — Land Plant, eudicots	*Cicer arietinum*	Eukaryota — Clade IV	XP004491274.1	Yes	Yes	—	—	Yes
Pd-Ca4	Eukaryotes — Land Plant, eudicots	*Cicer arietinum*	Eukaryota — Clade IV	XP004503087.1	Yes	Yes	—	—	Yes
Pd-Ca5	Eukaryotes — Land Plant, eudicots	*Cicer arietinum*	Eukaryota — Clade I	XP004499921.1	Yes	Yes	Yes	—	—
Pd-Cc1	Eukaryotes — Land Plant, eudicots	*Citrus clementina*	Eukaryota — Clade I	XP006445810.1	Yes	Yes	Yes	—	—
Pd-Cc2	Eukaryotes — Land Plant, eudicots	*Citrus clementina*	Eukaryota — Clade I	XM006438904.1	Yes	Yes	Yes	—	—
Pd-Cc3	Eukaryotes — Land Plant, eudicots	*Citrus clementina*	Eukaryota — Clade IV	XP006437065.1	Yes	Yes	—	—	Yes
Pd-Cc4	Eukaryotes — Land Plant, eudicots	*Citrus clementina*	Eukaryota — Clade IV	XP006430357.1	Yes	Yes	—	—	Yes
Pd-Cc5	Eukaryotes — Land Plant, eudicots	*Citrus clementina*	Eukaryota — Clade II	XP006451470.1	Yes	Yes	—	Yes	—
Pd-Cfc1	Eukaryotes — Land Plant, eudicots	*Coffea canephora*	Eukaryota — Clade I	CDP17949.1	—	—	Yes	—	—
Pd-Cfc2	Eukaryotes — Land Plant, eudicots	*Coffea canephora*	Eukaryota — Clade II	CDP03163.1	—	—	—	Yes	—
Pd-Cm1	Eukaryotes — Land Plant, eudicots	*Cucumis melo*	Eukaryota — Clade I	XP008461322.1	—	—	Yes	—	—
Pd-Cm2	Eukaryotes — Land Plant, eudicots	*Cucumis melo*	Eukaryota — Clade IV	XP008464648.1	—	—	—	—	Yes
Pd-Cms1	Eukaryotes — Land Plant, eudicots	*Camelina sativa*	Eukaryota — Clade I	XP010419967.1	—	—	Yes	—	—
Pd-Cms2	Eukaryotes — Land Plant, eudicots	*Camelina sativa*	Eukaryota — Clade I	XP010492143.1	—	—	Yes	—	—
Pd-Cms3	Eukaryotes — Land Plant, eudicots	*Camelina sativa*	Eukaryota — Clade I	XP010456521.1; XP010453464.2	—	—	Yes	—	—
Pd-Cms4	Eukaryotes — Land Plant, eudicots	*Camelina sativa*	Eukaryota — Clade IV	XP010508481.1	—	—	—	—	Yes
Pd-Cms5	Eukaryotes — Land Plant, eudicots	*Camelina sativa*	Eukaryota — Clade IV	XP010511508.1	—	—	—	—	Yes
Pd-Cms6	Eukaryotes — Land Plant, eudicots	*Camelina sativa*	Eukaryota — Clade IV	XP010415130.1	—	—	—	—	Yes
Pd-Cms7	Eukaryotes — Land Plant, eudicots	*Camelina sativa*	Eukaryota — Clade II	XP010438275.1	—	—	—	Yes	—
Pd-Cms8	Eukaryotes — Land Plant, eudicots	*Camelina sativa*	Eukaryota — Clade II	XP010433074.1	—	—	—	Yes	—
Pd-Cms9	Eukaryotes — Land Plant, eudicots	*Camelina sativa*	Eukaryota — Clade II	XP010447817.1	—	—	—	Yes	—
Pd-Cpa1	Eukaryotes — Land Plant, eudicots	*Capsicum annuum*	Eukaryota — Clade I	AKJ71425.1	—	—	Yes	—	—
Pd-Cpa2	Eukaryotes — Land Plant, eudicots	*Capsicum annuum*	Eukaryota — Clade IV	XP016580438.1	—	—	—	—	Yes
Pd-Cpa3	Eukaryotes — Land Plant, eudicots	*Capsicum annuum*	Eukaryota — Clade IV	XP016560216.1	—	—	—	—	Yes
Pd-Cpa4	Eukaryotes — Land Plant, eudicots	*Capsicum annuum*	Eukaryota — Clade II	XP016572934.1	—	—	—	Yes	—
Pd-Cr1	Eukaryotes — Land Plant, eudicots	*Capsella rubella*	Eukaryota — Clade II	XP006283498.1	—	—	—	Yes	—
Pd-Cs1	Eukaryotes — Land Plant, eudicots	*Cucumis sativus*	Eukaryota — Clade II	XP004141056.1	Yes	Yes	—	Yes	—
Pd-Cs2	Eukaryotes — Land Plant, eudicots	*Cucumis sativus*	Eukaryota — Clade II	XP004141056.1	Yes	Yes	—	—	—
Pd-Cs3	Eukaryotes — Land Plant, eudicots	*Cucumis sativus*	Eukaryota — Clade IV	XP004149519.1	Yes	Yes	—	—	Yes
Pd-Cs4	Eukaryotes — Land Plant, eudicots	*Cucumis sativus*	Eukaryota — Clade IV	XP004147745.1	Yes	Yes	—	—	Yes
Pd-Cs5	Eukaryotes — Land Plant, eudicots	*Cucumis sativus*	Eukaryota — Clade I	XP004158184.1; XP004135942.1	Yes	Yes	Yes	—	—
Pd-Cts1	Eukaryotes — Land Plant, eudicots	*Citrus sinensis*	Eukaryota — Clade I	KDO48663.1	—	—	Yes	—	—
Pd-Cts2	Eukaryotes — Land Plant, eudicots	*Citrus sinensis*	Eukaryota — Clade II	KDO57776.1	—	—	—	Yes	—
Pd-Eg1	Eukaryotes — Land Plant, eudicots	*Eucalyptus grandis*	Eukaryota — Clade I	XP010067844.1	—	—	Yes	—	—
Pd-Eg2	Eukaryotes — Land Plant, eudicots	*Eucalyptus grandis*	Eukaryota — Clade I	XP010068969.1	—	—	Yes	—	—
Pd-Eg3	Eukaryotes — Land Plant, eudicots	*Eucalyptus grandis*	Eukaryota — Clade IV	XP010032762.1	—	—	—	—	Yes
Pd-Eg4	Eukaryotes — Land Plant, eudicots	*Eucalyptus grandis*	Eukaryota — Clade II	XP010047573.1	—	—	—	Yes	—
Pd-Es1	Eukaryotes — Land Plant, eudicots	*Eutrema salsugineum*	Eukaryota — Clade I	XP006399866.1	—	—	Yes	—	—
Pd-Es2	Eukaryotes — Land Plant, eudicots	*Eutrema salsugineum*	Eukaryota — Clade IV	XP006411576.1	—	—	—	—	Yes
Pd-Es3	Eukaryotes — Land Plant, eudicots	*Eutrema salsugineum*	Eukaryota — Clade IV	XP006402729.1;	—	—	—	—	Yes
Pd-Es4	Eukaryotes — Land Plant, eudicots	*Eutrema salsugineum*	Eukaryota — Clade IV	XP006391313.1	—	—	—	—	Yes
Pd-Es5	Eukaryotes — Land Plant, eudicots	*Eutrema salsugineum*	Eukaryota — Clade II	XP006412816.1	—	—	—	Yes	—
Pd-Ga1	Eukaryotes — Land Plant, eudicots	*Genlisea aurea*	Eukaryota — Clade I	EPS72582.1	—	—	Yes	—	—
Pd-Gb1	Eukaryotes — Land Plant, eudicots	*Gossypium barbadense*	Eukaryota — Clade I	AGO02007.1	—	—	Yes	—	—
Pd-Gh1	Eukaryotes — Land Plant, eudicots	*Gossypium hirsutum*	Eukaryota — Clade I	AGO02008.1	—	—	Yes	—	0
Pd-Gh2	Eukaryotes — Land Plant, eudicots	*Gossypium hirsutum*	Eukaryota — Clade I	AGC67035.1	—	—	Yes	—	—
Pd-Gh3	Eukaryotes — Land Plant, eudicots	*Gossypium hirsutum*	Eukaryota — Clade IV	XP016712212.1	—	—	—	—	Yes
Pd-Gh4	Eukaryotes — Land Plant, eudicots	*Gossypium hirsutum*	Eukaryota — Clade IV	XP016701742.1	—	—	—	—	Yes
Pd-Gm1	Eukaryotes — Land Plant, eudicots	*Glycine max*	Eukaryota — Clade I	XP003534392.1	Yes	Yes	Yes	—	—
Pd-Gm2	Eukaryotes — Land Plant, eudicots	*Glycine max*	Eukaryota — Clade I	XP003535841.2	Yes	Yes	Yes	—	—
Pd-Gm3	Eukaryotes — Land Plant, eudicots	*Glycine max*	Eukaryota — Clade IV	XP003519523.1	Yes	Yes	—	—	Yes
Pd-Gm4	Eukaryotes — Land Plant, eudicots	*Glycine max*	Eukaryota — Clade IV	XP003551948.1	Yes	Yes	—	—	Yes
Pd-Gm5	Eukaryotes — Land Plant, eudicots	*Glycine max*	Eukaryota — Clade IV	XP003545339.1	Yes	Yes	—	—	Yes
Pd-Gm6	Eukaryotes — Land Plant, eudicots	*Glycine max*	Eukaryota — Clade IV	XP003538368.1	Yes	Yes	—	—	Yes
Pd-Gm7	Eukaryotes — Land Plant, eudicots	*Glycine max*	Eukaryota — Clade IV	XP003553017.1	Yes	Yes	—	—	Yes
Pd-Gm8	Eukaryotes — Land Plant, eudicots	*Glycine max*	Eukaryota — Clade II	XP014624869.1	Yes	Yes	—	Yes	—
Pd-Gm9	Eukaryotes — Land Plant, eudicots	*Glycine max*	Eukaryota — Clade II	XP006587008.1	Yes	Yes	—	Yes	—
Pd-Jc1	Eukaryotes — Land Plant, eudicots	*Jatropha curcas*	Eukaryota — Clade I	XP012065601.1	—	—	Yes	—	—
Pd-Jc2	Eukaryotes — Land Plant, eudicots	*Jatropha curcas*	Eukaryota — Clade I	XP012080084.1	—	—	Yes	—	—
Pd-Jc3	Eukaryotes — Land Plant, eudicots	*Jatropha curcas*	Eukaryota — Clade I	XP012080085.1	—	—	Yes	—	—
Pd-Jc4	Eukaryotes — Land Plant, eudicots	*Jatropha curcas*	Eukaryota — Clade IV	XP012074697.1	—	—	—	—	Yes
Pd-Jc5	Eukaryotes — Land Plant, eudicots	*Jatropha curcas*	Eukaryota — Clade IV	XP012072709.1	—	—	—	—	Yes
Pd-Jc6	Eukaryotes — Land Plant, eudicots	*Jatropha curcas*	Eukaryota — Clade II	XP012076928.1	—	—	—	Yes	—
Pd-Md1	Eukaryotes — Land Plant, eudicots	*Malus domestica*	Eukaryota — Clade I	BAF02915.1	Yes	Yes	Yes	—	—
Pd-Md2	Eukaryotes — Land Plant, eudicots	*Malus domestica*	Eukaryota — Clade IV	XP008342321.1	—	—	—	—	Yes
Pd-Md3	Eukaryotes — Land Plant, eudicots	*Malus domestica*	Eukaryota — Clade IV	XP008380158.1	—	—	—	—	Yes
Pd-Md4	Eukaryotes — Land Plant, eudicots	*Malus domestica*	Eukaryota — Clade IV	XP008381525.1; XP028964486.1	—	—	—	—	Yes
Pd-Md5	Eukaryotes — Land Plant, eudicots	*Malus domestica*	Eukaryota — Clade IV	XP008343150.2	—	—	—	—	Yes
Pd-Md6	Eukaryotes — Land Plant, eudicots	*Malus domestica*	Eukaryota — Clade II	XP008367921.1	—	—	—	Yes	—
Pd-Md7	Eukaryotes — Land Plant, eudicots	*Malus domestica*	Eukaryota — Clade II	XP008344020.1	—	—	—	Yes	—
Pd-Mn1	Eukaryotes — Land Plant, eudicots	*Morus notabilis*	Eukaryota — Clade I	XP010097441.1	—	—	Yes	—	—
Pd-Mn2	Eukaryotes — Land Plant, eudicots	*Morus notabilis*	Eukaryota — Clade II	XP010090043.1	—	—	—	Yes	—
Pd-Mt1	Eukaryotes — Land Plant, eudicots	*Medicago truncatula*	Eukaryota — Clade I	XP003599417.1	Yes	Yes	Yes	—	—
Pd-Mt2	Eukaryotes — Land Plant, eudicots	*Medicago truncatula*	Eukaryota — Clade IV	XP003617318.1	Yes	Yes	—	—	Yes
Pd-Mt3	Eukaryotes — Land Plant, eudicots	*Medicago truncatula*	Eukaryota — Clade IV	XP003600621.1	Yes	Yes	—	—	Yes
Pd-Mt4	Eukaryotes — Land Plant, eudicots	*Medicago truncatula*	Eukaryota — Clade II	XP003595165.1	Yes	Yes	—	Yes	—
Pd-Mt5	Eukaryotes — Land Plant, eudicots	*Medicago truncatula*	Eukaryota — Clade I	AFK47076.1	—	—	Yes	—	—
Pd-Nn1	Eukaryotes — Land Plant, eudicots	*Nelumbo nucifera*	Eukaryota — Clade I	XP010257143.1	—	—	Yes	—	—
Pd-Ns1	Eukaryotes — Land Plant, eudicots	*Nicotiana sylvestris*	Eukaryota — Clade I	XP009758563.1	—	—	Yes	—	—
Pd-Ns2	Eukaryotes — Land Plant, eudicots	*Nicotiana sylvestris*	Eukaryota — Clade II	XP009795930.1	—	—	—	Yes	—
Pd-Nt1	Eukaryotes — Land Plant, eudicots	*Nicotiana tabacum*	Eukaryota — Clade I	NP001313211.1; XP016515915	Yes	Yes	Yes	—	—
Pd-Nt2	Eukaryotes — Land Plant, eudicots	*Nicotiana tabacum*	Eukaryota — Clade IV	XP016436167.1	—	—	—	—	Yes
Pd-Nt3	Eukaryotes — Land Plant, eudicots	*Nicotiana tabacum*	Eukaryota — Clade IV	XP016450958.1	—	—	—	—	Yes
Pd-Nt4	Eukaryotes — Land Plant, eudicots	*Nicotiana tabacum*	Eukaryota — Clade II	XP016442430.1	—	—	—	Yes	—
Pd-Nt5	Eukaryotes — Land Plant, eudicots	*Nicotiana tabacum*	Eukaryota — Clade II	XP016493240.1	—	—	—	Yes	—
Pd-Nt6	Eukaryotes — Land Plant, eudicots	*Nicotiana tabacum*	Eukaryota — Clade II	XP016494589.1	—	—	—	Yes	—
Pd-Pm1	Eukaryotes — Land Plant, eudicots	*Prunus mume*	Eukaryota — Clade I	XP008231466.1	—	—	Yes	—	—
Pd-Pr1	Eukaryotes — Land Plant, eudicots	*Prunus persica*	Eukaryota — Clade II	XP007201933.1	Yes	Yes	—	Yes	—
Pd-Pr2	Eukaryotes — Land Plant, eudicots	*Prunus persica*	Eukaryota — Clade I	XP007219032.1	Yes	Yes	Yes	—	—
Pd-Pr3	Eukaryotes — Land Plant, eudicots	*Prunus persica*	Eukaryota — Clade IV	XP007217227.1;	Yes	Yes	—	—	Yes
Pd-Pr4	Eukaryotes — Land Plant, eudicots	*Prunus persica*	Eukaryota — Clade IV	XP007215363.1;	Yes	Yes	—	—	Yes
Pd-Pt1	Eukaryotes — Land Plant, eudicots	*Populus trichocarpa*	Eukaryota — Clade I	XP002318037.2	Yes	Yes	Yes	—	—
Pd-Pt2	Eukaryotes — Land Plant, eudicots	*Populus trichocarpa*	Eukaryota — Clade I	XP002299932.1; XP002299932.2	Yes	Yes	Yes	—	—
Pd-Pt3	Eukaryotes — Land Plant, eudicots	*Populus trichocarpa*	Eukaryota — Clade IV	XP002302123.1;	Yes	Yes	—	—	Yes
Pd-Pt4	Eukaryotes — Land Plant, eudicots	*Populus trichocarpa*	Eukaryota — Clade IV	XP002306765.2; XP002306765.2	Yes	Yes	—	—	Yes
Pd-Pt5	Eukaryotes — Land Plant, eudicots	*Populus trichocarpa*	Eukaryota — Clade IV	XP002305924.1	Yes	Yes	—	—	Yes
Pd-Pt6	Eukaryotes — Land Plant, eudicots	*Populus trichocarpa*	Eukaryota — Clade II	XP002309226.1	Yes	Yes	—	Yes	—
Pd-Pv1	Eukaryotes — Land Plant, eudicots	*Phaseolus vulgaris*	Eukaryota — Clade I	XP007148899.1	—	—	Yes	—	—
Pd-Pv2	Eukaryotes — Land Plant, eudicots	*Phaseolus vulgaris*	Eukaryota — Clade IV	XP007141453.1	—	—	—	—	Yes
Pd-Pv3	Eukaryotes — Land Plant, eudicots	*Phaseolus vulgaris*	Eukaryota — Clade IV	XP007146970.1	—	—	—	—	Yes
Pd-Pv4	Eukaryotes — Land Plant, eudicots	*Phaseolus vulgaris*	Eukaryota — Clade II	XP007138729.1	—	—	—	Yes	—
Pd-Rc1	Eukaryotes — Land Plant, eudicots	*Ricinus communis*	Eukaryota — Clade I	XP002517783.1	Yes	Yes	Yes	—	—
Pd-Rc2	Eukaryotes — Land Plant, eudicots	*Ricinus communis*	Eukaryota — Clade IV	XP002521588.1	Yes	Yes	—	—	Yes
Pd-Rc3	Eukaryotes — Land Plant, eudicots	*Ricinus communis*	Eukaryota — Clade IV	EEF36188.1	Yes	Yes	—	—	Yes
Pd-Rc4	Eukaryotes — Land Plant, eudicots	*Ricinus communis*	Eukaryota — Clade I	XP002511416.1	Yes	Yes	Yes	—	—
Pd-Rc5	Eukaryotes — Land Plant, eudicots	*Ricinus communis*	Eukaryota — Clade II	XP002514111.1	Yes	Yes	—	Yes	—
Pd-Rs1	Eukaryotes — Land Plant, eudicots	*Raphanus sativus*	Eukaryota — Clade IV	XP018439969.1	—	—	—	—	Yes
Pd-Rs2	Eukaryotes — Land Plant, eudicots	*Raphanus sativus*	Eukaryota — Clade IV	XP018460415.1	—	—	—	—	Yes
Pd-Rs3	Eukaryotes — Land Plant, eudicots	*Raphanus sativus*	Eukaryota — Clade IV	XP018462513.1	—	—	—	—	Yes
Pd-Rs4	Eukaryotes — Land Plant, eudicots	*Raphanus sativus*	Eukaryota — Clade II	XP018477297.1	—	—	—	Yes	—
Pd-Rs5	Eukaryotes — Land Plant, eudicots	*Raphanus sativus*	Eukaryota — Clade II	XP018443814.1	—	—	—	Yes	—
Pd-Sei1	Eukaryotes — Land Plant, eudicots	*Sesamum indicum*	Eukaryota — Clade I	XP011087975.1	—	—	Yes	—	—
Pd-Sei2	Eukaryotes — Land Plant, eudicots	*Sesamum indicum*	Eukaryota — Clade IV	XP011100368.1	—	—	—	—	Yes
Pd-Sei3	Eukaryotes — Land Plant, eudicots	*Sesamum indicum*	Eukaryota — Clade IV	XP011085441.1	—	—	—	—	Yes
Pd-Sei4	Eukaryotes — Land Plant, eudicots	*Sesamum indicum*	Eukaryota — Clade IV	XP011096924.1	—	—	—	—	Yes
Pd-Sei5	Eukaryotes — Land Plant, eudicots	*Sesamum indicum*	Eukaryota — Clade II	XP011078201.1	—	—	—	Yes	—
Pd-Sei6	Eukaryotes — Land Plant, eudicots	*Sesamum indicum*	Eukaryota — Clade II	XP011093505.1	—	—	—	Yes	—
Pd-Sl1	Eukaryotes — Land Plant, eudicots	*Solanum lycopersicum*	Eukaryota — Clade I	XP004229651.1	Yes	Yes	Yes	—	—
Pd-Sl2	Eukaryotes — Land Plant, eudicots	*Solanum lycopersicum*	Eukaryota — Clade II	XP004239292.1	Yes	Yes	—	Yes	—
Pd-Sl3	Eukaryotes — Land Plant, eudicots	*Solanum lycopersicum*	Eukaryota — Clade II	XP004243758.1	Yes	Yes	—	Yes	—
Pd-Sl4	Eukaryotes — Land Plant, eudicots	*Solanum lycopersicum*	Eukaryota — Clade II	XP010314061.1	Yes	Yes	—	Yes	—
Pd-Sl5	Eukaryotes — Land Plant, eudicots	*Solanum lycopersicum*	Eukaryota — Clade IV	XP004232664.1	—	—	—	—	Yes
Pd-Sl6	Eukaryotes — Land Plant, eudicots	*Solanum lycopersicum*	Eukaryota — Clade IV	XP004234492.1	—	—	—	—	Yes
Pd-Sl7	Eukaryotes — Land Plant, eudicots	*Solanum lycopersicum*	Eukaryota — Clade IV	XP004243630.1	—	—	—	—	Yes
Pd-Sl8	Eukaryotes — Land Plant, eudicots	*Solanum lycopersicum*	Eukaryota — Clade IV	XP004251556.1	—	—	—	—	Yes
Pd-St1	Eukaryotes — Land Plant, eudicots	*Solanum tuberosum*	Eukaryota — Clade I	XP006345421.1	Yes	Yes	Yes	—	—
Pd-St2	Eukaryotes — Land Plant, eudicots	*Solanum tuberosum*	Eukaryota — Clade IV	XP006357889.1	—	—	—	—	Yes
Pd-St3	Eukaryotes — Land Plant, eudicots	*Solanum tuberosum*	Eukaryota — Clade IV	XP006351988.1	—	—	—	—	Yes
Pd-St4	Eukaryotes — Land Plant, eudicots	*Solanum tuberosum*	Eukaryota — Clade IV	XP006348188.1	—	—	—	—	Yes
Pd-St5	Eukaryotes — Land Plant, eudicots	*Solanum tuberosum*	Eukaryota — Clade IV	XP006343304.1	—	—	—	—	Yes
Pd-St6	Eukaryotes — Land Plant, eudicots	*Solanum tuberosum*	Eukaryota — Clade II	XP006353583.1	—	—	—	Yes	—
Pd-St7	Eukaryotes — Land Plant, eudicots	*Solanum tuberosum*	Eukaryota — Clade II	XP006348065.1	—	—	—	Yes	—
Pd-St8	Eukaryotes — Land Plant, eudicots	*Solanum tuberosum*	Eukaryota — Clade II	XP006346568.1	—	—	—	Yes	—
Pd-St9	Eukaryotes — Land Plant, eudicots	*Solanum tuberosum*	Eukaryota — Clade II	XP006338189.1	—	—	—	Yes	—
Pd-Tc1	Eukaryotes — Land Plant, eudicots	*Theobroma cacao*	Eukaryota — Clade I	XP007052480.1; XP007052480.2	—	—	Yes	—	—
Pd-Tc2	Eukaryotes — Land Plant, eudicots	*Theobroma cacao*	Eukaryota — Clade IV	XP007048902.2	—	—	—	—	Yes
Pd-Tc3	Eukaryotes — Land Plant, eudicots	*Theobroma cacao*	Eukaryota — Clade II	XP007012841.1	—	—	—	Yes	—
Pd-Th1	Eukaryotes — Land Plant, eudicots	*Tarenaya hassleriana*	Eukaryota — Clade I	XP010547285.1	—	—	Yes	—	—
Pd-Th2	Eukaryotes — Land Plant, eudicots	*Tarenaya hassleriana*	Eukaryota — Clade I	XP010557801.1	—	—	Yes	—	—
Pd-Th3	Eukaryotes — Land Plant, eudicots	*Tarenaya hassleriana*	Eukaryota — Clade IV	XP010525644.1	—	—	—	—	Yes
Pd-Th4	Eukaryotes — Land Plant, eudicots	*Tarenaya hassleriana*	Eukaryota — Clade IV	XP010550699.1	—	—	—	—	Yes
Pd-Th5	Eukaryotes — Land Plant, eudicots	*Tarenaya hassleriana*	Eukaryota — Clade IV	XP010551566.1	—	—	—	—	Yes
Pd-Th6	Eukaryotes — Land Plant, eudicots	*Tarenaya hassleriana*	Eukaryota — Clade II	XP010541064.1	—	—	—	Yes	—
Pd-Th7	Eukaryotes — Land Plant, eudicots	*Tarenaya hassleriana*	Eukaryota — Clade II	XP010526397.1	—	—	—	Yes	—
Pd-Va1	Eukaryotes — Land Plant, eudicots	*Vigna angularis*	Eukaryota — Clade I	KOM42459.1	—	—	Yes	—	—
Pd-Va2	Eukaryotes — Land Plant, eudicots	*Vigna angularis*	Eukaryota — Clade II	KOM39894.1	—	—	—	Yes	—
Pd-Vv1	Eukaryotes — Land Plant, eudicots	*Vitis vinifera*	Eukaryota — Clade I	XP002264412.1	Yes	Yes	Yes	—	—
Pd-Vv2	Eukaryotes — Land Plant, eudicots	*Vitis vinifera*	Eukaryota — Clade I	XP010651220.1	Yes	Yes	Yes	—	—
Pd-Vv3	Eukaryotes — Land Plant, eudicots	*Vitis vinifera*	Eukaryota — Clade I	XP002267667.1	Yes	Yes	Yes	—	—
Pd-Vv4	Eukaryotes — Land Plant, eudicots	*Vitis vinifera*	Eukaryota — Clade I	XP010659441.1	Yes	Yes	Yes	—	—
Pd-Vv5	Eukaryotes — Land Plant, eudicots	*Vitis vinifera*	Eukaryota — Clade IV	XP002282970.1	Yes	Yes	—	—	Yes
Pd-Vv6	Eukaryotes — Land Plant, eudicots	*Vitis vinifera*	Eukaryota — Clade IV	XP002264892.1	Yes	Yes	—	—	Yes
Pd-Vv7	Eukaryotes — Land Plant, eudicots	*Vitis vinifera*	Eukaryota — Clade II	CBI27910.3; XP010656196.1	Yes	Yes	—	Yes	—
Pl-Sl1	Eukaryotes — Land Plant, lycopodiophytes	*Selaginella lepidophylla*	Eukaryota — Clade II	BAS29669.1	—	—	—	Yes	—
Pl-Sm1	Eukaryotes — Land Plant, lycopodiophytes	*Selaginella moellendorffii*	Eukaryota — Clade I	XP002965265.1.	Yes	Yes	Yes	—	—
Pl-Sm2	Eukaryotes — Land Plant, lycopodiophytes	*Selaginella moellendorffii*	Eukaryota — Clade I	XP002969966.1; EFJ29090.1	Yes	Yes	Yes	—	—
Pl-Sm3	Eukaryotes — Land Plant, lycopodiophytes	*Selaginella moellendorffii*	Eukaryota — Clade IV	XP002960711.1	Yes	Yes	—	—	Yes
Pl-Sm4	Eukaryotes — Land Plant, lycopodiophytes	*Selaginella moellendorffii*	Eukaryota — Clade IV	XP002965599.1; EFJ33019.1	Yes	Yes	—	—	Yes
Pl-Sm5	Eukaryotes — Land Plant, lycopodiophytes	*Selaginella moellendorffii*	Eukaryota — Clade II	XP002984796.1	Yes	Yes	—	Yes	—
Pl-Sm6	Eukaryotes — Land Plant, lycopodiophytes	*Selaginella moellendorffii*	Eukaryota — Clade II	XP002985859.1	Yes	Yes	—	Yes	—
Pm-Ae1	Eukaryotes — Land Plant, monocots	*Aegilops tauschii*	Eukaryota — Clade I	EMT31301.1; XP020188563.1	—	—	Yes	—	—
Pm-Bd1	Eukaryotes — Land Plant, monocots	*Brachypodium distachyon*	Eukaryota — Clade I	KQJ96059.1	Yes	Yes	Yes	—	—
Pm-Bd2	Eukaryotes — Land Plant, monocots	*Brachypodium distachyon*	Eukaryota — Clade II	XP003567045.1	Yes	Yes	—	Yes	—
Pm-Bd3	Eukaryotes — Land Plant, monocots	*Brachypodium distachyon*	Eukaryota — Clade IV	XP010240449.1	—	—	—	—	Yes
Pm-Bd4	Eukaryotes — Land Plant, monocots	*Brachypodium distachyon*	Eukaryota — Clade IV	XP003580795.1	—	—	—	—	Yes
Pm-Hv1	Eukaryotes — Land Plant, monocots	*Hordeum vulgare*	Eukaryota — Clade I	CAC42080.1	Yes	Yes	Yes	—	—
Pm-Hv2	Eukaryotes — Land Plant, monocots	*Hordeum vulgare*	Eukaryota — Clade I	CAC42081.1	Yes	Yes	Yes	—	—
Pm-Hv3	Eukaryotes — Land Plant, monocots	*Hordeum vulgare*	Eukaryota — Clade I	BAJ98538.1	Yes	Yes	Yes	—	—
Pm-Hv4	Eukaryotes — Land Plant, monocots	*Hordeum vulgare*	Eukaryota — Clade IV	BAJ93995.1	Yes	Yes	—	—	Yes
Pm-Hv5	Eukaryotes — Land Plant, monocots	*Hordeum vulgare*	Eukaryota — Clade II	BAK01686.1	Yes	Yes	—	Yes	—
Pm-Ma1	Eukaryotes — Land Plant, monocots	*Musa acuminata*	Eukaryota — Clade I	XP009400016.1	Yes	Yes	Yes	—	—
Pm-Ma2	Eukaryotes — Land Plant, monocots	*Musa acuminata*	Eukaryota — Clade IV	XP009399229.1	Yes	Yes	—	—	Yes
Pm-Ma3	Eukaryotes — Land Plant, monocots	*Musa acuminata*	Eukaryota — Clade IV	ABF69974.1	Yes	Yes	—	—	Yes
Pm-Ma4	Eukaryotes — Land Plant, monocots	*Musa acuminata*	Eukaryota — Clade IV	XP009384199.1	Yes	Yes	—	—	Yes
Pm-Ma5	Eukaryotes — Land Plant, monocots	*Musa acuminata*	Eukaryota — Clade IV	XP009386432.1	Yes	Yes	—	—	Yes
Pm-Ma6	Eukaryotes — Land Plant, monocots	*Musa acuminata*	Eukaryota — Clade IV	XP009418290.1	Yes	Yes	—	—	Yes
Pm-Ma7	Eukaryotes — Land Plant, monocots	*Musa acuminata*	Eukaryota — Clade II	XP009394109.1	Yes	Yes	—	Yes	—
Pm-Ma8	Eukaryotes — Land Plant, monocots	*Musa acuminata*	Eukaryota — Clade I	EMT31301.1; XP009406028.1	—	—	Yes	—	—
Pm-Os1	Eukaryotes — Land Plant, monocots	*Oryza sativa*	Eukaryota — Clade II	XP015630114.1	Yes	Yes	—	Yes	—
Pm-Os2	Eukaryotes — Land Plant, monocots	*Oryza sativa*	Eukaryota — Clade I	BAF11161.2	Yes	Yes	Yes	—	—
Pm-Os3	Eukaryotes — Land Plant, monocots	*Oryza sativa*	Eukaryota — Clade IV	XP015636130.1	Yes	Yes	—	—	Yes
Pm-Os4	Eukaryotes — Land Plant, monocots	*Oryza sativa*	Eukaryota — Clade IV	XP015637015.1	Yes	Yes	—	—	Yes
Pm-Os5	Eukaryotes — Land Plant, monocots	*Oryza sativa*	Eukaryota — Clade IV	XP015636452.1	Yes	Yes	—	—	Yes
Pm-Os6	Eukaryotes — Land Plant, monocots	*Oryza sativa*	Eukaryota — Clade I	BAF24924.2	Yes	Yes	Yes	—	—
Pm-Os7	Eukaryotes — Land Plant, monocots	*Oryza sativa*	Eukaryota — Clade I	BAF24925.1	Yes	Yes	Yes	—	—
Pm-Pd1	Eukaryotes — Land Plant, monocots	*Phoenix dactylifera*	Eukaryota — Clade I	XP008798475.1	—	—	Yes	—	—
Pm-Sb1	Eukaryotes — Land Plant, monocots	*Sorgum bicolor*	Eukaryota — Clade I	XP002445199.1	Yes	Yes	Yes	—	—
Pm-Sb2	Eukaryotes — Land Plant, monocots	*Sorgum bicolor*	Eukaryota — Clade IV	XP002448555.1	Yes	Yes	—	—	Yes
Pm-Sb3	Eukaryotes — Land Plant, monocots	*Sorgum bicolor*	Eukaryota — Clade IV	XP002448744.1	Yes	Yes	—	—	Yes
Pm-Sb4	Eukaryotes — Land Plant, monocots	*Sorgum bicolor*	Eukaryota — Clade IV	XP002448742.1	Yes	Yes	—	—	Yes
Pm-Sb5	Eukaryotes — Land Plant, monocots	*Sorgum bicolor*	Eukaryota — Clade II	XP002458387.1	Yes	Yes	—	Yes	—
Pm-Si1	Eukaryotes — Land Plant, monocots	*Setaria italica*	Eukaryota — Clade I	XP004985372.1	Yes	Yes	Yes	—	—
Pm-Si2	Eukaryotes — Land Plant, monocots	*Setaria italica*	Eukaryota — Clade I	XP004985369.1	Yes	Yes	Yes	—	—
Pm-Si3	Eukaryotes — Land Plant, monocots	*Setaria italica*	Eukaryota — Clade I	KQK92160.1	Yes	Yes	—	—	—
Pm-Si4	Eukaryotes — Land Plant, monocots	*Setaria italica*	Eukaryota — Clade I	KQK92161.1	Yes	Yes	—	—	—
Pm-Si5	Eukaryotes — Land Plant, monocots	*Setaria italica*	Eukaryota — Clade II	XP004969720.1	Yes	Yes	—	Yes	—
Pm-Si6	Eukaryotes — Land Plant, monocots	*Setaria italica*	Eukaryota — Clade IV	XP004960122.1	—	—	—	—	Yes
Pm-Si7	Eukaryotes — Land Plant, monocots	*Setaria italica*	Eukaryota — Clade IV	XP004960123.1	—	—	—	—	Yes
Pm-Si8	Eukaryotes — Land Plant, monocots	*Setaria italica*	Eukaryota — Clade IV	XP004976853.1	—	—	—	—	Yes
Pm-Zm1	Eukaryotes — Land Plant, monocots	*Zea mays*	Eukaryota — Clade I	NP001105106.1	Yes	Yes	Yes	—	—
Pm-Zm2	Eukaryotes — Land Plant, monocots	*Zea mays*	Eukaryota — Clade IV	ACN36050.1	Yes	Yes	—	—	Yes
Pm-Zm3	Eukaryotes — Land Plant, monocots	*Zea mays*	Eukaryota — Clade IV	ACF78749.1	Yes	Yes	—	—	Yes
Pm-Zm4	Eukaryotes — Land Plant, monocots	*Zea mays*	Eukaryota — Clade II	NP001169333.1	Yes	Yes	—	Yes	—
Pp-Ac1	Eukaryotes — Land Plant, pinophytes	*Araucaria cunninghamii*	Eukaryota — Clade IV	A0A0D6R4D6	—	—	—	—	Yes
Pp-Pg1	Eukaryotes — Land Plant, pinophytes	*Picea glauca*	Eukaryota — Clade I	GQ03710J21	—	—	Yes	—	—
Pp-Pp1	Eukaryotes — Land Plant, pinophytes	*Pinus pinaster*	Eukaryota — Clade IV	BX254044.1	—	—	—	—	Yes
Pp-Ps1	Eukaryotes — Land Plant, pinophytes	*Pinus sylvestris*	Eukaryota — Clade I	ADQ37306.1	Yes	Yes	Yes	—	—
Pp-Wn1	Eukaryotes — Land Plant, pinophytes	*Wollemia nobilis*	Eukaryotes — Clade IV	A0A0C9QS89	—	—	—	—	Yes
Mac-DrA	Eukaryotes — Metazoa, actinopterygii	*Danio rerio*	Eukaryota — Clade II	NP001314981.1	Yes	Yes	—	—	—
Mac-DrS	Eukaryotes — Metazoa, actinopterygii	*Danio rerio*	Eukaryota — Clade II	AAH66413	Yes	Yes	—	—	—
Ma-XlA	Eukaryotes — Metazoa, amphibians	*Xenopus laevis*	Eukaryota — Clade II	NP001088588.1	Yes	Yes	—	—	—
Ma-XlS	Eukaryotes — Metazoa, amphibians	*Xenopus laevis*	Eukaryota — Clade II	AAH72220; XP018098470.1	Yes	Yes	—	—	—
Mc-Bf	Eukaryotes — Metazoa, cephalochordates	*Branchiostoma floridae*	Eukaryota — Clade II	XP002608290.1; XP002225568.1	Yes	Yes	—	—	—
Mi-Am	Eukaryotes — Metazoa, insects	*Apis mellifera*	Eukaryota — Clade II	XP001122522	Yes	Yes	—	—	—
Mi-Dm	Eukaryotes — Metazoa, insects	*Drosophila melanogaster*	Eukaryota — Clade II	AAL39756	Yes	Yes	—	—	—
Mi-Phc	Eukaryotes — Metazoa, insects	*Pediculus humanus corporis*	Eukaryota — Clade II	EEB13427	Yes	Yes	—	—	—
Mm-EqA	Eukaryotes — Metazoa, mammals	*Equus caballus*	Eukaryota — Clade II	XP014588897.1	Yes	Yes	—	—	—
Mm-EqS	Eukaryotes — Metazoa, mammals	*Equus caballus*	Eukaryota — Clade II	XP014590530.1	Yes	Yes	—	—	—
Mm-HsA	Eukaryotes — Metazoa, mammals	*Homo sapiens*	Eukaryota — Clade II	NP690875.1	Yes	Yes	—	—	—
Mm-HsS	Eukaryotes — Metazoa, mammals	*Homo sapiens*	Eukaryota — Clade II	AAK55763.1; Q99K82	Yes	Yes	—	—	—
Mm-MeA	Eukaryotes — Metazoa, mammals	*Macropus eugenii*	Eukaryota — Clade II	ENSMEUP00000004459	Yes	Yes	—	—	—
Mm-MeS	Eukaryotes — Metazoa, mammals	*Macropus eugenii*	Eukaryota — Clade II	ENSMEUP00000000797	Yes	Yes	—	—	—
Mm-MmA	Eukaryotes — Metazoa, mammals	*Mus musculus*	Eukaryota — Clade II	NP722478.2	Yes	Yes	—	—	—
Mm-MmMAO-A[2]	Eukaryotes — Metazoa, mammals	*Mus musculus*	Archaea	NP776101.3	Yes	—	—	—	—
Mm-MmMAO-B[2]	Eukaryotes — Metazoa, mammals	*Mus musculus*	Archaea	NP766366.2	Yes	—	—	—	—
Mm-MmS	Eukaryotes — Metazoa, mammals	*Mus musculus*	Eukaryota — Clade II	NP663508.1	Yes	Yes	—	—	—
Rb-Ph	Eukaryotes — Red algae, bangiophytes	*Pyropia haitanensis*	Eukaryota — Clade I	GADD01000777.1	Yes	Yes	—	—	—
Rb-Pu	Eukaryotes — Red algae, bangiophytes	*Porphyra umbilicalis*	Eukaryota — Clade I	OSX76648	Yes	Yes	—	—	—
Rc-Cc	Eukaryotes — Red algae, cyanidophytes	*Chondrus crispus*	Eukaryota — Clade I	XP005714872	Yes	Yes	—	—	—
Rc-Gs	Eukaryotes — Red algae, cyanidophytes	*Galdieria sulphuraria*	Eukaryota — Clade II	EME29606	Yes	Yes	—	—	—
Rf-ka	Eukaryotes — Red algae, florideophyceae	*Kappaphycus alvarezii*	Eukaryota — Clade I	IHJY-2054821	Yes	Yes	—	—	—
Rp-Pop1	Eukaryotes — Red algae, porphyridiophytes	*Porphyridium purpureum*	Eukaryota — Clade II	KAA8499925	Yes	Yes	—	—	—
Rp-Pop2	Eukaryotes — Red algae, porphyridiophytes	*Porphyridium purpureum*	Eukaryota	KAA8499002	Yes	Yes	—	—	—
RH-Pb1	Eukaryotes — Rhizaria, phytomyxea	*Plasmodiophora brassicae*	Eukaryota — Clade IV	SPQ93354	Yes	Yes	—	—	—
RH-Pb2	Eukaryotes — Rhizaria, phytomyxea	*Plasmodiophora brassicae*	Eukaryota	CEP02494	Yes	Yes	—	—	—
SD-Fc1	Eukaryotes — Stramenopiles, diatoms	*Fragilariopsis cylindrus* CCMP1102	Eukaryota	OEU15358	Yes	Yes	—	—	—
SD-Fc2	Eukaryotes — Stramenopiles, diatoms	*Fragilariopsis cylindrus* CCMP1103	Eukaryota	OEU17981	Yes	Yes	—	—	—
SD-Fc4	Eukaryotes — Stramenopiles, diatoms	*Fragilariopsis cylindrus* CCMP1104	Eukaryota	OEU10590	Yes	Yes	—	—	—
SD-Pt1	Eukaryotes — Stramenopiles, diatoms	*Phaeodactylum tricornutum* CCAP 1055/1	Eukaryota — Clade IV	XP002178648	Yes	Yes	—	—	—
SD-Pt2	Eukaryotes — Stramenopiles, diatoms	*Phaeodactylum tricornutum* CCAP 1055/2	Eukaryota	XP002186444	Yes	Yes	—	—	—
SD-Pt4	Eukaryotes — Stramenopiles, diatoms	*Phaeodactylum tricornutum* CCAP 1055/3	Eukaryota — Clade II	XP002176436	Yes	Yes	—	—	—
SD-To1	Eukaryotes — Stramenopiles, diatoms	*Thalassiosira oceanica*	Eukaryota	EJK49320.1	Yes	Yes	—	—	—
SD-To2	Eukaryotes — Stramenopiles, diatoms	*Thalassiosira oceanica*	Eukaryota	EJK71405.1	Yes	Yes	—	—	—
SD-To3	Eukaryotes — Stramenopiles, diatoms	*Thalassiosira oceanica*	Eukaryota	EJK62008.1	Yes	Yes	—	—	—
SO-Pi	Eukaryotes — Stramenopiles, oomycetes	*Phytophthora infestans* T30-4	Eukaryota	XP002905580.1	Yes	Yes	—	—	—
SO-Pr1	Eukaryotes — Stramenopiles, oomycetes	*Phytophthora ramorum*	Eukaryota	H3GS50	Yes	Yes	—	—	—
SO-Pr2	Eukaryotes — Stramenopiles, oomycetes	*Phytophthora ramorum*	Eukaryota	H3GHR9	Yes	Yes	—	—	—
SO-Pr3	Eukaryotes — Stramenopiles, oomycetes	*Phytophthora ramorum*	Eukaryota	H3GS49	Yes	Yes	—	—	—
SO-Ps1	Eukaryotes — Stramenopiles, oomycetes	*Phytophthora sojae*	Eukaryota	XP009539477.1	Yes	Yes	—	—	—
SO-Ps2	Eukaryotes — Stramenopiles, oomycetes	*Phytophthora sojae*	Eukaryota	XP009526364.1	Yes	Yes	—	—	—
SO-Pu	Eukaryotes — Stramenopiles, oomycetes	*Pythium ultimum*	Eukaryota	K3X946	Yes	Yes	—	—	—

[1]: putrescine oxidase

[2]: monoamine oxidase
